# Dynamic Enantioconvergent Desaturation of 4,5-Disubstituted γ‑Lactones in Whole Cells of *Rhodococcus erythropolis*

**DOI:** 10.1021/jacs.5c19136

**Published:** 2025-12-29

**Authors:** Maria C. Cancellieri, Filip Boratyński, Stefano Serra, Dawid Hernik, Francesco G. Gatti

**Affiliations:** † Department of Chemistry, Materials and Chemical Engineering “G. Natta”, 18981 Politecnico di Milano, P.zza L. da Vinci 32, Milano 20133, Italy; ‡ Department of Food Chemistry and Biocatalysis, Wrocław University of Environmental and Life Sciences, Norwida 25, 50-375 Wrocław, Poland; § Consiglio Nazionale delle Ricerche, Istituto di Scienze e Tecnologie Chimiche, Via Mancinelli 7, Milan 20131, Italy

## Abstract

The α,β-desaturation of esters is one of the most challenging transformations in organic synthesis. While transition-metal-catalyzed methods have significantly advanced in the last decades, they often require high catalyst loading and nongreen solvents and rarely enable stereoselective transformations. Here we show that whole cells of *Rhodococcus erythropolis* enable a dynamic enantioconvergent desaturation of 4,5-disubstituted γ-butyrolactones (*Quercus*-like lactones), funneling all four initial stereoisomers into a single (*R*)-configured product with excellent enantioselectivity (up to *ee* 99%). Mechanistic investigations with deuterium-labeled substrates reveal a highly complex pathway involving multiple transformations catalyzed by a network of enzymes. The process includes lactone ring-opening by esterases, stereoselective oxidation of secondary alcohols by alcohol dehydrogenases (ADHs), desaturation of carboxylates by ene-reductases (ERs), and stereoselective reductive steps mediated by ERs and ketoreductases (KRs). The final stereochemical outcome arises from the combination of two stereoediting transformations: (*i*) a site-selective de-epimerization catalyzed by ADH/KR, and (*ii*) an ER-mediated *E* → Z isomerization coupled to the irreversible lactonization (thermodynamic sink). Finally, this dynamic enantioconvergent biocatalytic desaturation was applied to the preparation of a key intermediate used in the total synthesis of forskolin, showcasing the potential of biocatalytic desaturations as a greener and more stereoselective alternative to traditional chemical methods with broad implications for asymmetric synthesis and sustainable chemistry.

## Graphical abstract

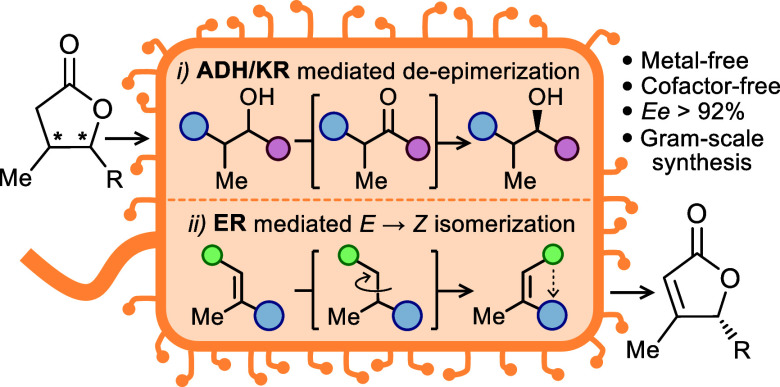

## Introduction

Catalytic desaturation of carbonyl compounds is a highly attractive transformation, as it provides the most atom-economical route to α,β-unsaturated carbonyl compounds. Due to the strategic importance of these structural motifs, present in many bioactive molecules (such as drugs, fragrances, and flavors) and synthetic intermediates, significant efforts have been dedicated to discovering new and efficient catalytic methods to perform this transformation over the last decades.^1^

Ever since the pioneering works of Saegusa and Tsuji,^2^ in which Pd-based catalysis was for the first time applied to the dehydrosilylation of silyl enol ethers, impressive advancements have been made, especially if compared to the early methodologies based on strong oxidants such as SeO_2_ and periodic acid.^1a,3^ For instance, the recent development of Cu- and Pt-based catalytic systems has enabled the direct dehydrogenation of ketones without the need to first prepare silyl enol ether derivatives; in addition, the substrate scope was expanded to other important carbonyl derivatives, such as lactones and lactams.^4,5^ Furthermore, the electrochemical implementation of Tsuji’s procedure^2b^ seems to be particularly promising, because it avoids the use of co-oxidants like the diallyl carbonate, significantly decreasing the wastes.^6^ However, these methodologies are not yet fully aligned with the principles of green chemistry.^7^ Specifically, they often require excessively high catalyst loadings, rarely utilize oxygen as the oxidant,^8^ and are mainly conducted in nongreen solvents.^9^

The biocatalytic counterpart to the chemical dehydrogenations likely drew inspiration from the oxidation of succinic acid to fumaric acid, an essential transformation in the Krebs cycle. The reaction is catalyzed by the enzyme succinate dehydrogenases, where the flavoprotein subunit facilitates the hydride abstraction from the succinate enolate to the flavin cofactor.^10^ Massey demonstrated that also flavoproteins belonging to the class of ene-reductase (ER) enzymes can desaturate carbonyl compounds (aldehydes or ketones) simply by substituting the natural flavin mononucleotide (FMN) cofactor with a synthetic analogue having a higher redox potential.^11^ After the desaturation step, the reduced form of flavin is oxidized by O_2_, resulting in the formation of H_2_O_2_ and the regeneration of the oxidized cofactor required for catalytic turnover (Figure 1A). ERs are redox enzymes largely employed in the stereospecific reduction of C=C double bonds conjugated with electron-withdrawing groups (typical EWGs are −CO of ketones, aldehydes and methyl esters, −CN, and −NO_2_).^12^

**Figure 1:**
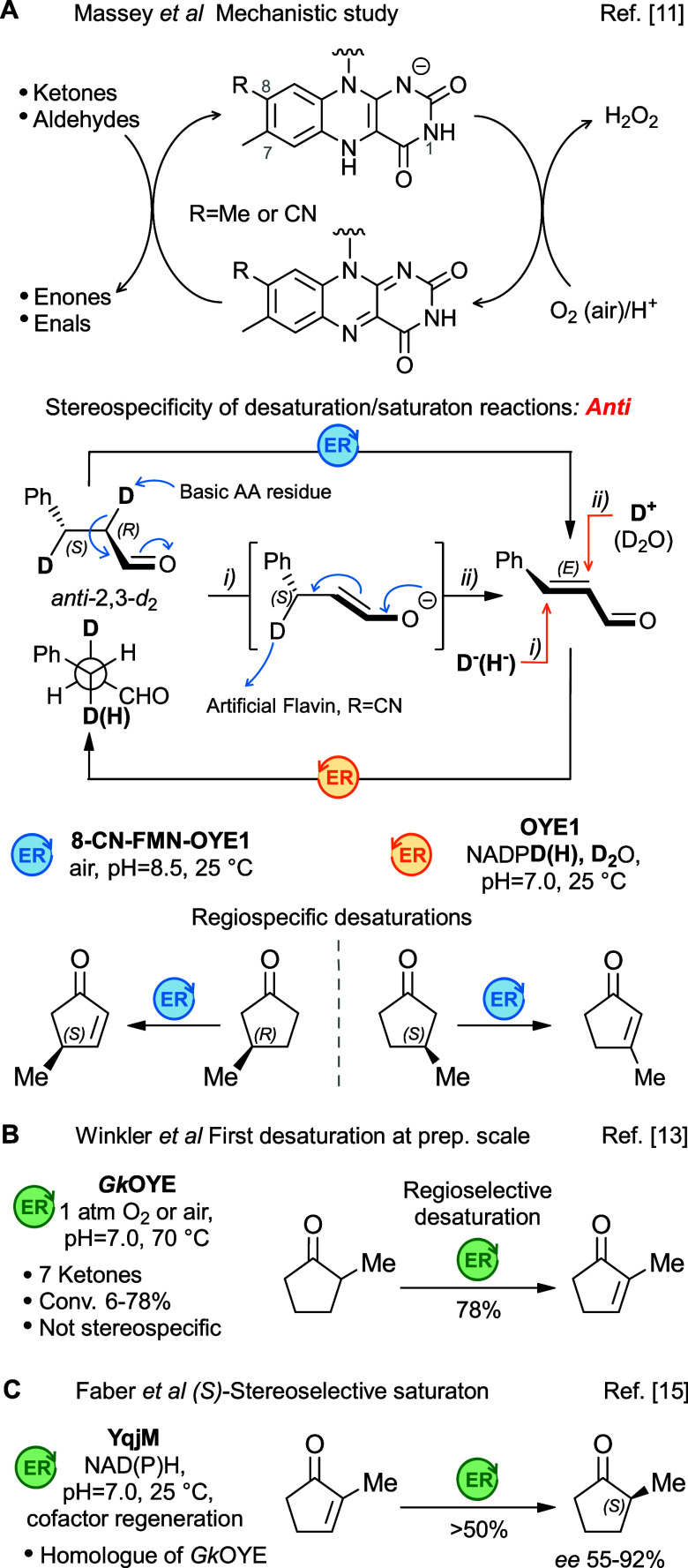
Flavoprotein-catalyzed desaturations/saturations.

Deuterium labeling experiments have shown that the desaturation catalyzed by OYE1 (an ER belonging to the Old Yellow Enzyme family) occurs through a two-step reaction mechanism with *anti*-stereospecificity, and consequently it is regiospecific. Indeed, the desaturation of (3*S*)-methylcyclopentanone yielded exclusively the most substituted enone, whereas its enantiomer gave the disubstituted regioisomer (Figure 1A). But only with the use of *Gk*OYE (an OYE homologue from the thermophilic bacterium *Geobacillus kaustophilus*) was it possible to dehydrogenate several cyclic ketones in moderate to good yields on a preparative scale. In addition, this enzyme does not need any modification of FMN cofactor, which significantly improves the reaction’s practicality and scalability.^13^ However, in this case, the reaction was not regiospecific but rather regioselective, favoring the formation of the most substituted enones. For example, the desaturation of the racemic mixture of 2-methylcyclopentanone gave exclusively the 2-methylcyclopent-2-en-one regioisomer (Figure 1B). Interestingly, the C=C double-bond reduction of the same enone catalyzed by YqjM from *Bacillus subtilis* (homologue of *Gk*OYE,^13^ both enzymes belong to the same cluster^14^), afforded the (*S*) enantiomer of the saturated ketone with variable enantiomeric excess (*ee* 55–92%), depending on the cofactor used (Figure 1C).^15^ More recently, enzymatic desaturations have been exploited for the stereoselective desymmetrization of cyclohexanones^16,17^ and for the preparation of coumarin derivatives,^18^ demonstrating a higher synthetic potential of biobased approaches compared to the chemical procedures.^19^

In the following we report on the first example of dynamic enantioconvergent desaturation of 4,5-disubstituted γ-lactones catalyzed by whole cells of *Rhodococcus* *erythropolis* affording enantioenriched α,β-unsaturated γ-lactones.

## Results and Discussion

### Stereoselective Desaturation and Substrate Scope

Recently we reported on the stereoselective synthesis of γ-lactone **1a** (one of the key aroma components of whisky) starting from its *syn* or *anti* diol precursors **2a** with good to excellent *ee* (Figure 2). The reaction was biocatalyzed by the alcohol dehydrogenases (ADHs) of *Rhodococcus* bacteria.^20^

**Figure 2:**
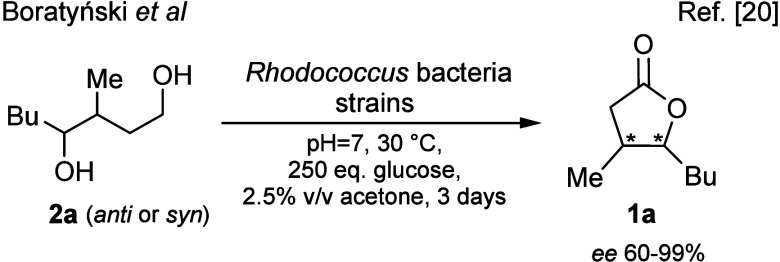
*Rhodococcus*-catalyzed stereoselective synthesis of whisky lactone.

While repeating the biotransformation of *anti*-**1a** with whole cells of *R. erythropolis* (strain DSM 44534), we discovered a previously unreported enzymatic activity for this microorganism. In addition to the enantioenriched whisky lactone we detected the presence of a very small amount of dehydrogenated lactone (<2% by GC-MS), later identified as **3a** (entry 1 of Table 1).

**Table 1:**
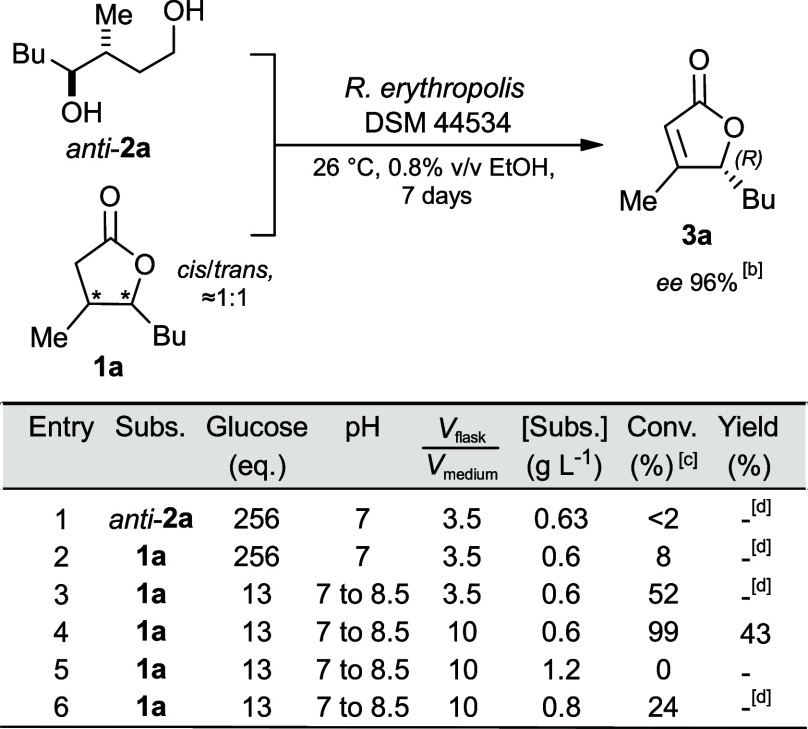
Reaction Optimization^a^

a Reaction conditions: growth medium (100 mL, *V*_medium_): demineralized H_2_O, glucose (1–19.6 g), NaCl (0.5 g), casein peptone (0.4 g), meat peptone (1 g), yeast extract (0.3 g), NaOAc (0.2 g), riboflavin (1 mg). After 24 h of incubation, substrate **2a** or **1a** (0.34 mmol) in EtOH (0.8 mL) was added at OD_600_ ≈ 1.3; the reaction mixture was shaken (130 rpm) under aerobic and sterile conditions in an Erlenmeyer flask (different sizes of flasks were tested, *V*_flask_).

b By chiral GC.

c Conversion of substrate into **3a**, by GC-MS relative to an internal standard (γ-decalactone).

d Product not isolated.

Over the past decades, *Rhodococcus* bacteria have proven to be a virtually endless source of enzymatic activities exploited in numerous organic transformations,^21,22^ including the reduction of C=C double bonds conjugated to different EWGs, but not of the α,β-unsaturated esters.^23^ In addition, the reverse reaction—̵the desaturation—had never been reported for this biocatalyst. Intrigued by the unexpected finding of **3a**, we hypothesized that enzymes with ER activity might be capable of directly desaturating whisky lactone **1a**.

To our delight, simply by favoring the oxidative reaction conditions typically applied in the ER-catalyzed desaturations (slightly basic medium and stronger aerobic conditions,^16–18^ entries 3, 4), decreasing substantially the equivalents of glucose (13 vs 256), and using a commercially available diastereomeric mixture of whisky lactone as substrate (**1a**, *cis*/*trans* = 46:54 by GC-MS) instead of a single diastereoisomer of diol **2a**, we were able to isolate **3a** in a yield of 43%, after column chromatography (entry 4). Then, with the optimized reaction conditions in our hands, we attempted to increase the substrate concentration (from 0.6 g L^–1^ to 1.2 g L^–1^, entries 4–6), but the formation of **3a** decreased significantly, indicating that the substrate concentration must be kept lower than 0.6 g L^–1^, very likely because of the toxicity of lactone-based substrates on the used *Rhodococcus erythropolis* strain. In addition, control experiments with heat-inactivated cells and spent medium showed no formation of **3a** (see Supporting Information).

Noteworthy, in the biotransformation under optimized conditions, only a trace amount of starting whisky lactone was left, mainly the *trans* diastereoisomer (Figure 3 and Figure 5). Then, the structural identity of **3a** was unambiguously confirmed by comparing ^1^H and ^13^C NMR spectra with those of an authentic sample of dehydrogenated lactone, chemically prepared by oxidation of the α-phenyl selenide derivative of **1a** (for more details see the Supporting Information, Scheme S6).

**Figure 3:**
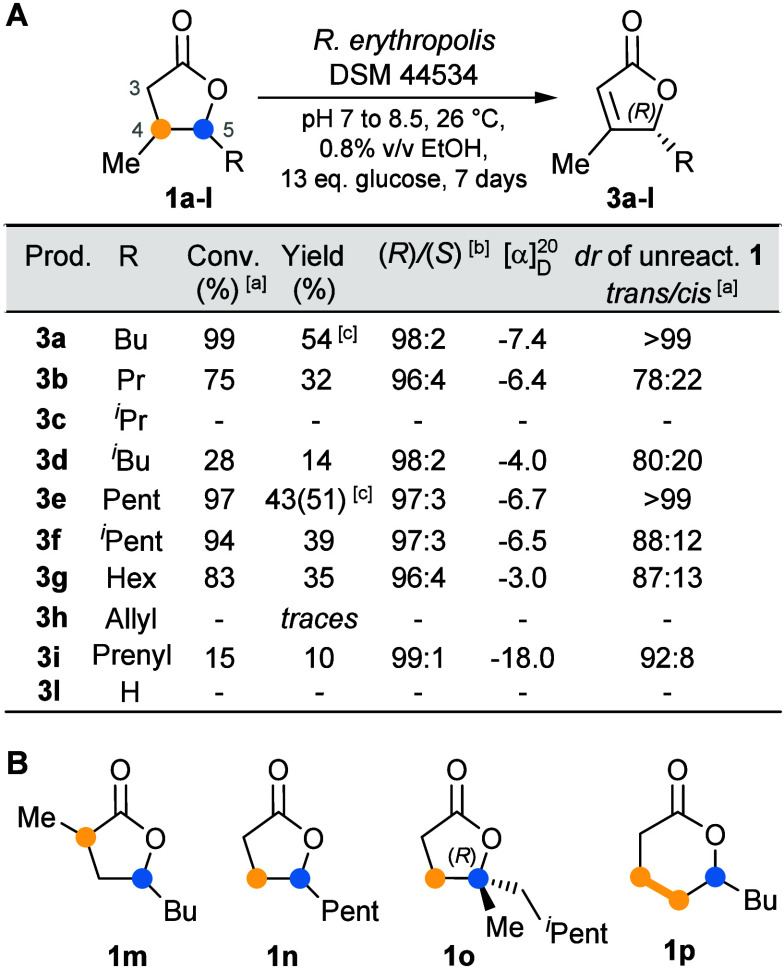
Substrate scope: (A) Biodesaturation of oak-like lactones; (B) substrates not desaturated. ^a^By GC-MS using γ-decalactone as internal standard. ^b^By chiral GC. ^c^Gram-scale preparation in a 2 L bioreactor (0.6 g L^–1^) using the optimized reaction conditions (entry 4, Table 1).

**Figure 4:**
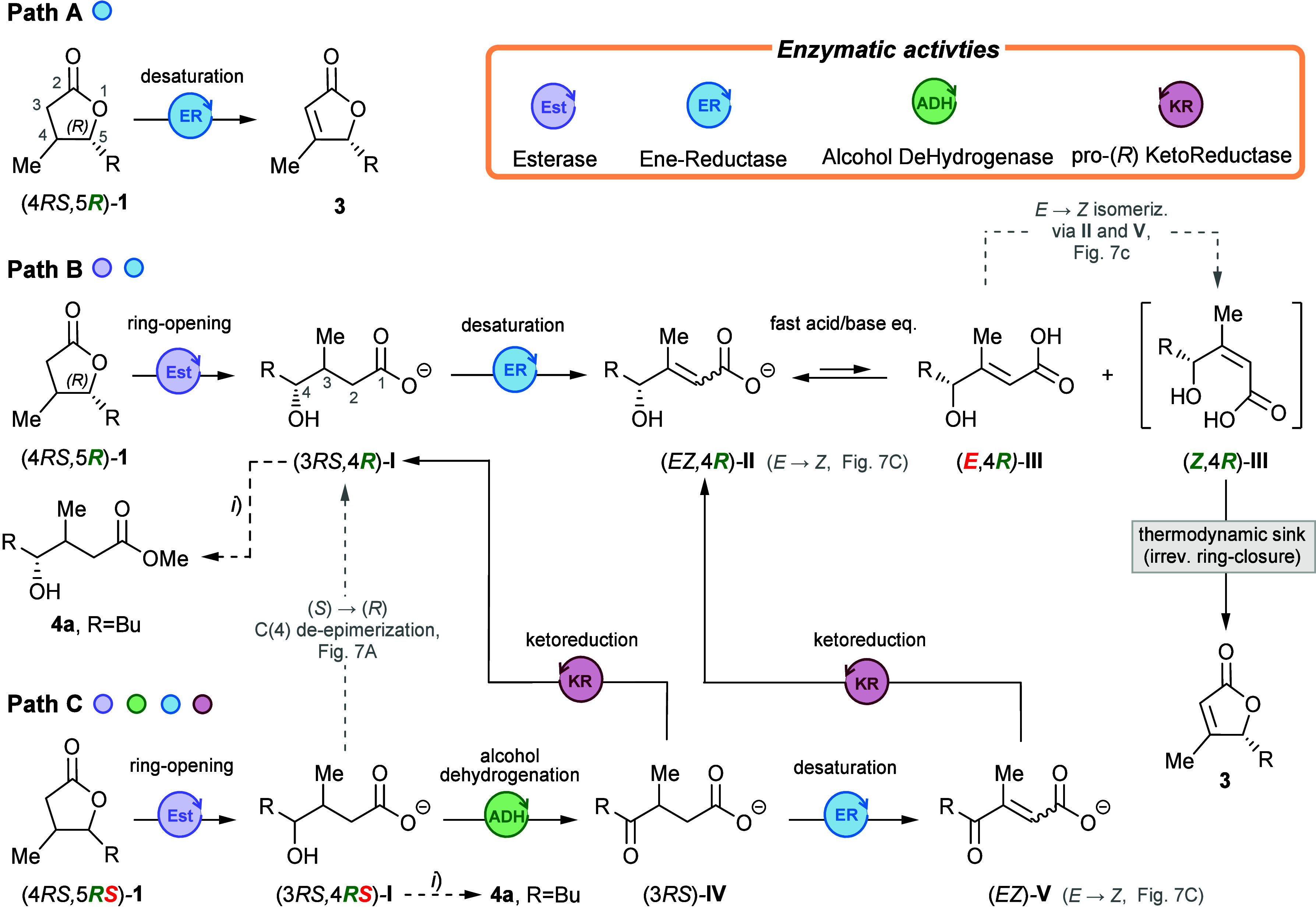
Proposed biochemical pathways for the stereoselective desaturation of 4,5-disubstituted γ-lactones. The three paths differ in the number of enzymes involved. In paths A and B only the (4*RS*,5*R*) stereoisomers of **1** are transformed, whereas in path C all stereoisomers of **1** are converted. For clarity, the enzymatic reverse reactions are not shown. (*i*) Derivatization with CH_2_N_2_ for the GC-MS identification of **I**.

**Figure 5:**
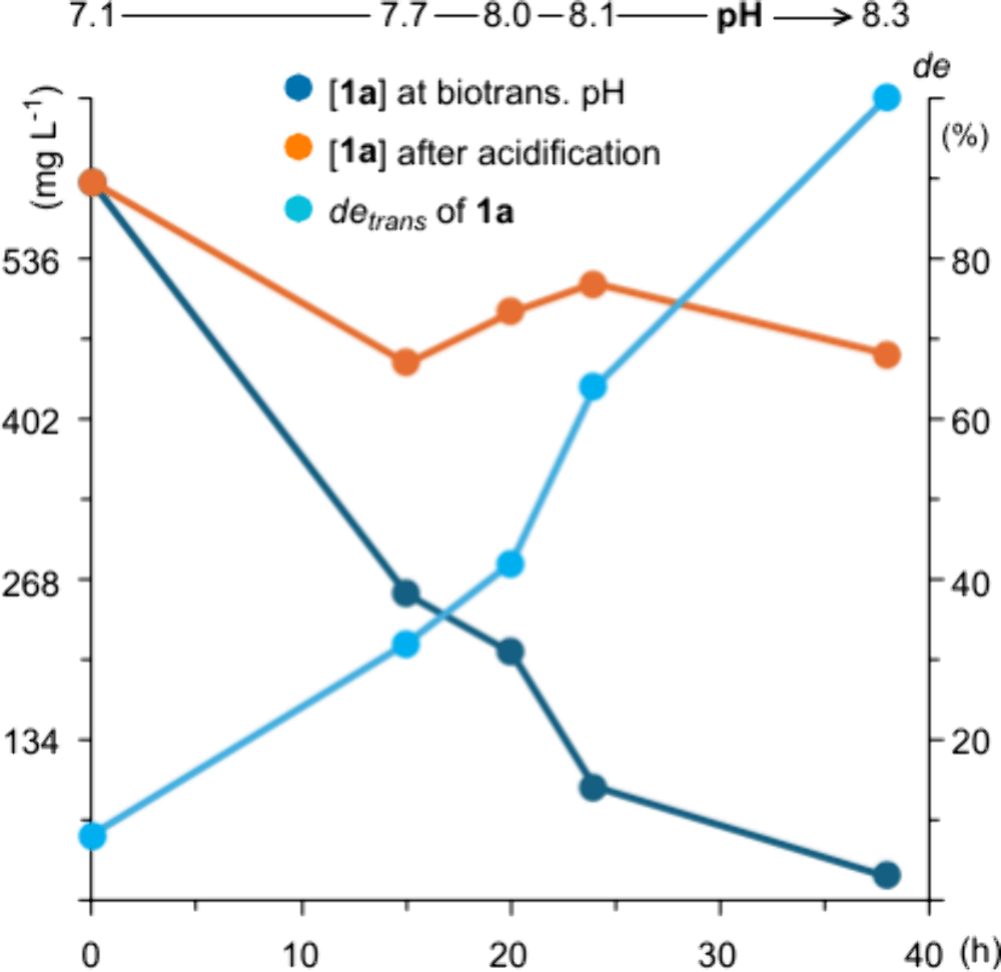
Reaction monitoring of the hydrolysis of whisky lactone (**1a**), using γ-decalactone as internal standard (GC-MS).

However, our great surprise came when we discovered that the formation of **3a** was highly enantioselective (*ee* 96% by chiral GC, see the Supporting Information) with stereopreference for the (*R*) absolute configuration ([α]_D_^20^ = −7.4, *c* 1.1, CHCl_3_, vs lit. [α]_D_ = +2.7, *c* 1.07, CHCl_3_, for (*S*) enantiomer, 94% *ee*).^24^

Encouraged by these results, we explored the substrate scope of this biotransformation, focusing our interest on the dehydrogenation of structurally similar disubstituted γ-lactones (**1b**-**i**, Figure 3A). This important class of natural compounds, also known as oak or *Quercus* lactones, is characterized by the methyl substituent at C(4) and an alkyl or alkenyl substituent at C(5) (for their preparation see the Supporting Information, Schemes S1 and S2).

Remarkably, seven out of ten substrates were converted. The desaturation of cognac lactone **1e** afforded **3e** with a yield and selectivity comparable to those achieved for **3a**. The (*R*) absolute configuration was assigned by reconverting the optically enriched **3e** (*ee* 94% by chiral GC) into the *cis* diastereoisomer of cognac lactone **1e** (*cis*/*trans* = 86:14, [α]_D_^25^ = +46.0, *c* 1.5, CHCl_3_, vs lit. [α]_D_^25^ = +63.3, *c* 1.12, CHCl_3_, for (4*R*,5*R*) enantiomer),^25^ through Rh/Al_2_O_3_-catalyzed hydrogenation of the C=C double bond (Scheme S7 of the Supporting Information). The yield of substrates with linear substituents (**1b** and **1g**) was comparable to those of cognac and whisky lactones, while for substrates with nonlinear substituents (**1c**,**d** and **1f**), the conversion was strongly influenced by the branching position: the closer the branching carbon was to C(5), the lower the yield. Substrates with alkenyl substituents were desaturated with a conversion that correlated with the distance between the C=C double-bond unsaturation and C(5). For instance, the substrate with the allyl chain (**1h**) was not dehydrogenated, whereas the eldanolide (**1i**), bearing the prenyl substituent, was transformed into **3i** in a 10% yield. Finally, (*R*) stereochemical configuration was assigned to all products, based on the chiral GC elution order and on the sign of the optical rotation value, which was consistent with those of lactones **3a** and **3e**.^26^

Testing substrates **1m** (Supporting Information, Scheme S3) and **1n**, both regioisomers of whisky lactone, revealed the crucial role of the methyl substituent at C(4) (Figure 3B). When this group was shifted from C(4) to C(3) or simply replaced by hydrogen (**1m** and **1n**, respectively), no formation of unsaturated lactones was detected. Likewise, β-methyl butyrolactone (**1l**) was not desaturated (Figure 3A), indicating that the methyl substituent at C(5) is also required to activate the desaturation pathway. Interestingly, lactone **1o** (Supporting Information, Scheme S4), bearing a quaternary carbon at the γ-position, was not dehydrogenated, most likely due to an increased steric hindrance at C(5) and/or the absence of the methyl substituent at C(4). Finally, δ-lactones such as **1p** proved to be unsuitable substrates for *R. erythropolis*-catalyzed desaturation (Figure 3B).

### Searching for Biosynthetic Pathways

When the desaturation was carried out in a bioreactor at gram-scale and under controlled aerobic conditions, both yield and reaction time were significantly improved (54% in 4 days vs 43% in 7 days for substrate **1a**; similar improvements were achieved for desaturating cognac lactone **1e**, Figure 3A). Given that the recovery of volatile and polar compounds from microbial broths is often nonquantitative, especially at low substrate concentrations (<1 g L^–1^), these yields are quite respectable. However, to gain deeper insight into the chemical pathway of this biotransformation, several questions must be addressed:

i) Is the unsaturated lactone formed by direct dehydrogenation^18^ of **1** (path A, Figure 4) or does it follow more complex synthetic routes that require an initial ring-opening step (paths B and/or C)?
ii) Does the observed enantioselectivity arise from a simple kinetic asymmetric transformation in which only the (4*RS*,5*R*)-stereoisomers of **1** react (thus limiting the maximum conversion to 50%, paths A and B), or is it the result of a dynamic enantioconvergent catalytic process^27,28^ in which all four initial stereoisomers are funneled to the final product with (*R*)-defined configuration (thus overcoming the 50% maximum conversion limit, path C)?
iii) If paths B and/or C are operative, are the desaturations entirely *Z* diastereoselective? And if not, how are the *E* isomers transformed into the final product?

To answer these fundamental questions, we designed a series of experiments based on the biodesaturation of whisky lactone and structurally related analogues.

At first, monitoring of the reaction time course (by GC-MS, Figure 5, Table S2 of the Supporting Information) showed that during the first phase of biotransformation (usually within 38 h), when the pH of the microbial broth under aerobic conditions naturally increases from neutral to basic (pH = 8.3–8.5), almost no dehydrogenated lactone was formed. Nonetheless, the concentration of the starting material decreased progressively, almost reaching its complete consumption (from 0.6 g L^–1^ to 0.01 g L^–1^). This outcome suggested that **1a** might be first hydrolyzed to the hydroxy carboxylate intermediate **I**, which only after the end of hydrolysis is transformed into **3a**. The slight acidification of a biotransformation sample (pH = 6), followed by treatment with CH_2_N_2_ after liquid–liquid extraction with EtOAc, showed the presence of **I** as a methyl ester derivative (**4a**), confirming our hypothesis on the hydrolase activity of *Rhodococcus erythropolis* (paths B and C, Figure 4).

Control experiments without the microorganism showed that at reaction conditions very similar to those of biotransformation (D_2_O, 50 mM Tris buffer pH = 8.5, 26 °C, 5 days), chemical hydrolysis of **1a** is negligible (by ^1^H NMR, Figure S1 of the Supporting Information), and it occurs only at high temperatures (*T* > 60 °C), but conversely to that observed in the biotransformation, it is not diastereoselective. Indeed, the reaction monitoring of biotransformation confirmed that during the hydrolysis *cis*-**1a** was consumed more rapidly than *trans*-**1a** (Figure 5 and Table S2 of the Supporting Information). Overall, these results suggest that direct α,β-dehydrogenation of **1a** is unlikely (path A). Thus, the desaturation likely proceeds through the more complex routes B and/or C, which might operate in a complementary rather than mutually exclusive fashion. To validate these two possible pathways, we investigated the desaturation with different deuterium-labeled substrates (**1a**-5-*d*, **2a**-1,1,4-*d*_3_, and *cis*-**1a**-3,4-*d*_2_; their preparation is described in the Supporting Information, Schemes S8 and S9) or in D_2_O-enriched medium (Figure 6A).

**Figure 6:**
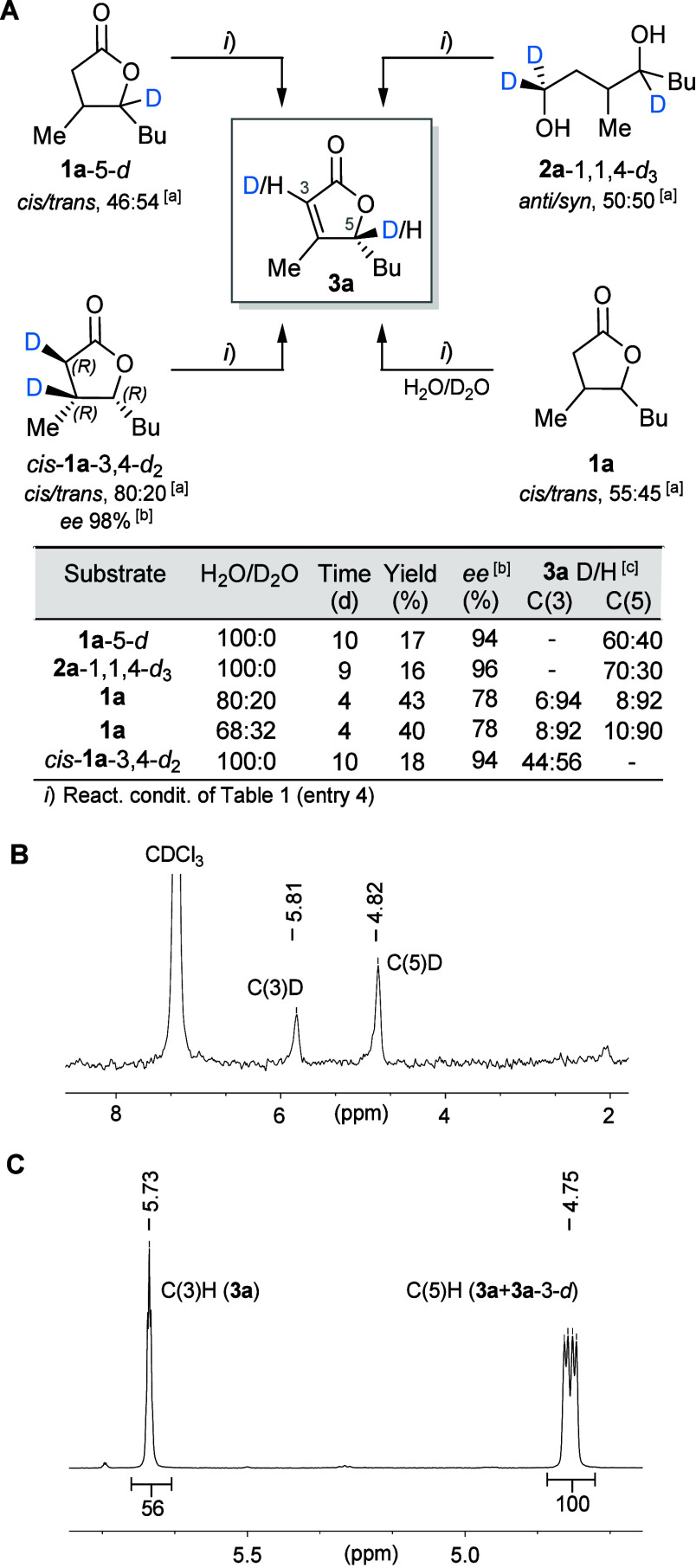
(A) Biotransformations with deuterium-labeled substrates or in D_2_O-enriched medium. (B) ^2^H NMR spectrum of **3a**-3,5-*d*_2_ in CHCl_3_ (61.4 MHz), obtained from biotransformation in H_2_O/D_2_O (68:32). (C) ^1^H NMR spectrum of a **3a**/**3a**-3-*d* mixture in CDCl_3_ (400 MHz), obtained from the biotransformation of **1a**-3,4-*d*_2_; the integration is calibrated with respect to the C(5)H signal. ^a^By GC-MS. ^b^By chiral GC. ^c^By ^1^H NMR.

First, all biotransformations of deuterated substrates gave **3a** with *ee* values very similar to that achieved in the biotransformation of unlabeled substrate **1a**, while the conversions were always lower (18–43%). It is important to emphasize that the deuterium-retention experiments provide qualitative, but not quantitative, information on the relative contribution of the competing pathways, likely due to kinetic isotopic effects. These effects operate on alcohol oxidation, ER-mediated desaturation/saturation, and KR-mediated reduction; hence the overall conversion of deuterated substrates is not proportional to the individual fluxes through path B or C.

The C(5)-deuterated whisky lactone (**1a**-5-*d*) gave a mixture of labeled and unlabeled products (**3a**-5-*d*/**3a** = 60:40, quantified by ^1^H NMR, see Supporting Information). Since only the direct desaturation of **I** to **II** intermediates would preserve the initial D-labeling in the final product, this result suggests that a non-negligible fraction of product has been formed through path B, in which no ADH-catalyzed oxidations occur. While the partial loss of deuterium at C(5) can be rationalized by a redox-based site-selective de-epimerization: the (3*RS*,4*S*) stereoisomers of **I**-4-*d* are first oxidized to the keto carboxylates (3*RS*)-**IV** by ADH and then reduced back to the (4*R*)-configured hydroxy acids by a keto reductases (KR).^27d^ Then, the resulting (3*RS*,4*R*)-**I** epimers re-enter path B, accounting for the formation of a mixture of unlabeled and D-labeled products (Figures 4 and 7A).

**Figure 7:**
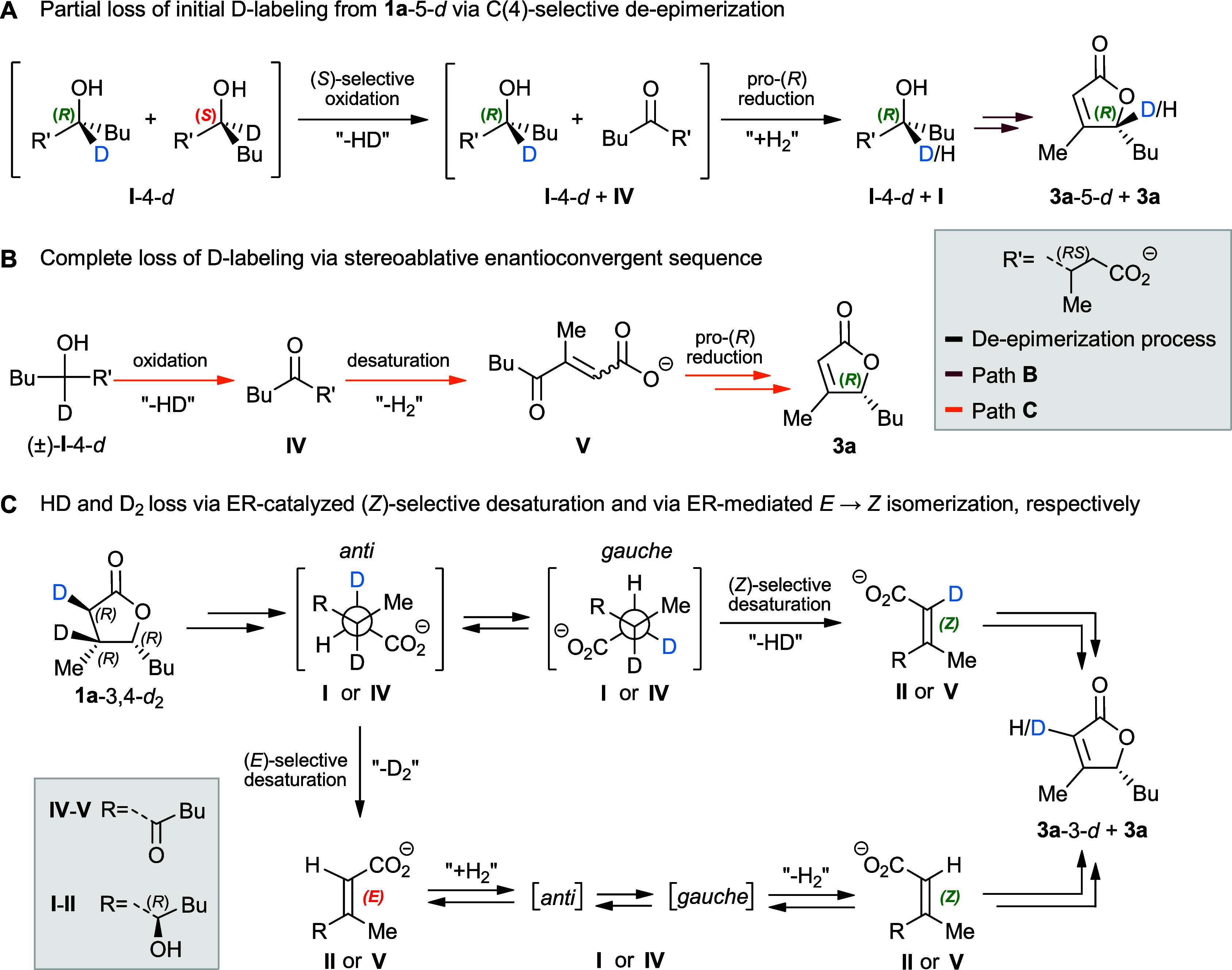
Proposed reaction pathways for the desaturation of the D-labeled substrates.

A complete loss of D-labeling can occur only if all four stereoisomers of **I**-4-*d* are oxidized to **IV**, irrespective of their C(4) configuration (path C). Then, the intermediate (3*RS*)-**IV** is first desaturated by an ER to the prochiral keto enoate **V** (full stereoablation) and subsequently is reduced by a KR, giving the unlabeled hydroxy enoate (4*R*)-**II** (reinstallation of chirality), common intermediate with path B (Figures 4 and 7B).

In principle, efficient de-epimerization of **I**, coupled with a faster desaturation of **I** relative to **IV**, could yield the unlabeled product exclusively via path B. Thus, the biotransformation of **1a**-5-*d* did not allow us to exclude the contribution of the second half of path C (**IV** → **V** → **II**), i.e., the stereoablative enantioconvergent sequence.^28^ In addition, it was not possible to assess whether the ER-catalyzed desaturations are intrinsically *Z*-selective; however after protonation of carboxylates **II** (*E*/*Z* diastereoisomers) to the corresponding hydroxy enoic acids **III**, only the diastereoisomer with *Z* configuration undergoes spontaneous ring-closure, forming the final product (Figure 4).

Similarly to **1a**-5-*d*, the trideuterated diol **2a**-1,1,4-*d*_3_ yielded a mixture of C(5) deuterium labeled and unlabeled lactones (**3a**-5-*d*/**3a** = 70:30, by ^1^H NMR). When the biotransformation was conducted in a mixed H_2_O/D_2_O medium (80:20 and 68:32, Figure 6A), the product was partially deuterated at C(5) (8–10% D-incorporation determined by ^1^H NMR, detected by ^2^H NMR, C(5)­D δ = 4.82 ppm, Figure 6B). All together these results further support our hypothesis regarding the involvement of KR and ADH activities either in the C(4)-selective de-epimerization process of **I** and/or in the stereoconvergent sequence of path C (Figure 4).

Quite unexpectedly, however, deuterium incorporation (6–8% by ^1^H NMR) also occurred at C(3) (detected by ^2^H NMR, C(3)­D δ = 5.81 ppm, Figure 6B). Initially, we suspected that this could be ascribable to the OH^–^-catalyzed α-deprotonation of **1a** or **I** species, but control experiments in D_2_O showed that these compounds are not chemically enolizable at the pH of biotransformation (by ^1^H NMR, Figure S1). Thus, we concluded that the pairs of intermediates **I**/**II** and/or **IV/V** must be involved in a desaturation/saturation equilibrium mediated very likely by enzymes with ER activity, in which D^+^ incorporation at the α position, i*.*e., C(2), occurs by quenching the enolate intermediate with D_2_O (second step of the C=C double-bond reduction mechanism, Figure 1A).

The dehydrogenation of the optically pure whisky lactone labeled at C(3) and C(4) (*cis*-**1a**-3,4-*d*_2_, 60% *de*, 98% *ee*) unexpectedly afforded an almost equimolar mixture of deuterated and nondeuterated products (**3a**-3-*d*/**3a** = 44:56, by ^1^H NMR, Figure 6C). The formation of nondeuterated **3a** could in principle arise from a *syn* elimination of D_2_, but such a stereochemical outcome would contradict the widely accepted mechanism of ER-catalyzed desaturations, which typically proceed through an *anti*-stereospecific elimination pathway (Figure 1A).^11,12^ Thus, if the ERs present in *R. erythropolis* desaturate carbonyl compounds with such a mechanism, the monodeuterated *Z* isomers of **II** and **V** intermediates (HD elimination) can be obtained only from the C(2)–C(3) *gauche* conformers of **I** and **IV** intermediates. Both monolabeled *Z* isomers can subsequently be transformed into the monodeuterated lactone **3a**-3-*d* (Figure 7C).

Conversely, the *E* isomers are obtained from intermediates **I** and **IV** adopting the *anti* conformation, but in this case the desaturation leads to the complete loss of the initial labeling (−D_2_).^29^ Since both *E* diastereoisomers (**II** and **V**) cannot undergo ring-closure and are not accumulated during the biotransformation, they must necessarily isomerize to *Z* through a new saturation/desaturation cycle (Figure 7C). This finding indicates that the formation of the unlabeled lactone **3a** from the optically pure *cis*-**1a**-3,4-*d*_2_ is possible only if the α,β-unsaturated intermediates (**II** and/or **V**) with *E* configuration isomerize to the corresponding *Z* isomers, unless we contemplate a desaturation with a reaction mechanism based on an unlike *syn* elimination.^11b^

### Validation of Proposed Intermediates

It is a fact that during the biotransformation we could not isolate or detect any of the proposed intermediates except for **I**, likely due to their low concentration, which made the liquid–liquid extraction from the microbial broth inefficient. Hence, to further validate the two proposed pathways, we decided to generate *in situ* the intermediates via hydrolysis of their ester precursors (**5a**–**7a**, for their preparation see Schemes S1 and S10 of the Supporting Information), relying on the well-known high hydrolase activity of *Rhodococcus erythropolis* toward esters (Figure 8).^21^

**Figure 8:**
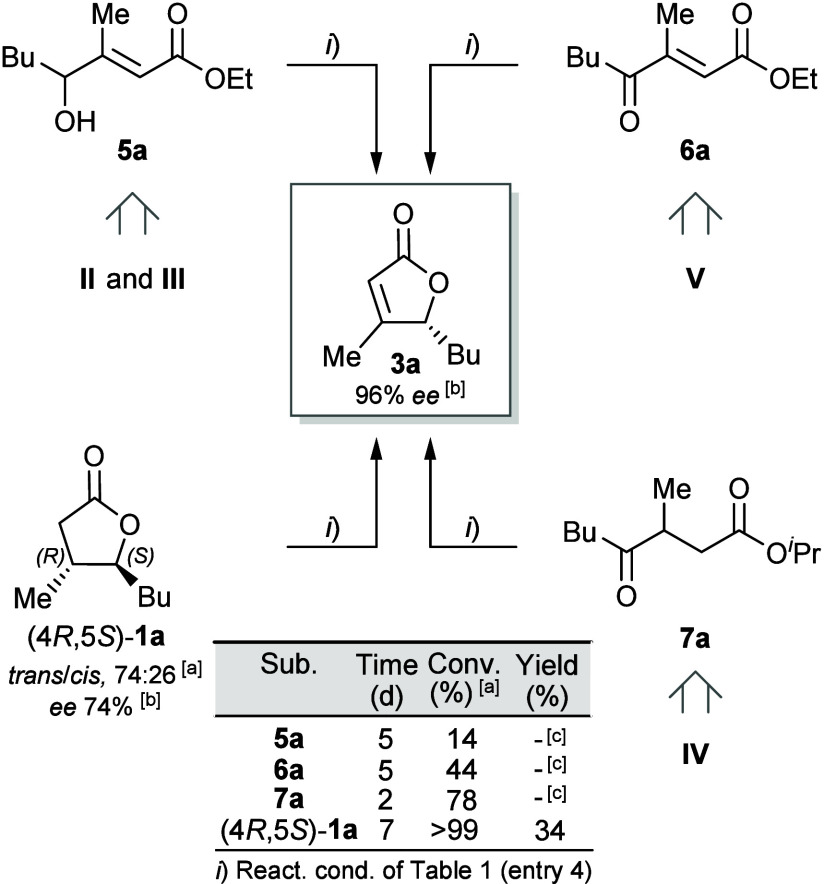
Biotransformations of ester derivatives of the proposed intermediates and of (5*S*) enantiomerically enriched **1a**. ^a^By GC-MS. ^b^By chiral GC. ^c^Not isolated.

The ethyl ester of (*E*)-**III**, i*.*e., **5a** (*E >* 99%, by ^1^H NMR), was biotransformed to **3a** with a conversion of 14%. This result confirms that **II** and **III** are indeed intermediates of both paths and that the *E* → *Z* isomerization must occur, as **5a** is clearly not geometrically suitable for lactonization. Similarly, the biotransformation of **6a** (ethyl ester of intermediate (*E*)-**V**) provided further evidence for path C. In this case, we observed a significantly higher conversion than that achieved with **5a**, likely because substrates like **6a**, with double activating groups, are usually well accepted by ERs.^30^ To test the existence of intermediate **IV**, the keto *i*-propyl ester **7a** was subjected to the same biotransformation conditions,^31^ and after only 2 days almost 80% of product was detected in the reaction mixture (Figure 8).

Lastly, by submitting a sample of whisky lactone enantiomerically enriched in the (4*RS*,5*S*)-epimers (75:25, for its preparation see Supporting Information) we furnished an additional proof for the C(4) de-epimerization process, since the dehydrogenated lactone was isolated in a yield quite similar to that obtained using the racemic mixture (34% vs 43%) and with the same *ee* (Figure 8). Indeed, only if the (3*RS*,4*S*) epimers of **I** (enantiomerically enriched *syn* and *anti* diastereoisomers) are converted into the (3*RS*,4*R*)-epimers is it possible to achieve similar yields; otherwise, in the absence of (3*RS*,4*S*) → (3*RS*,4*R*) de-epimerization, we should obtain **3a** in a much lower yield or with a much lower *ee* value. In this regard, the reaction monitored by chiral GC shows clearly a progressive increase in the (5*R*) stereoisomers (Figure 9). For example, after 5 days and a conversion of 50%, the combined ratio of **1a** and **3a** (5*R*) stereoisomers more than doubled, shifting from the initial 25:75 to 58:42.

**Figure 9:**
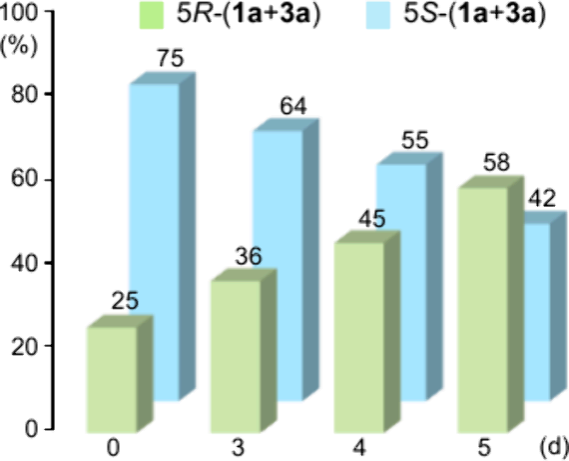
Time-dependent (5*R*)/(5*S*) ratio of **1a** + **3a** species during the biodesaturation of optically enriched (4*R*,5*S*)-**1a**.

### Computational Approach to the Thermodynamics of the *E/Z* Isomerization

In order to obtain more information on the thermodynamic parameters of the *gauche*/*anti* conformational equilibrium along the C(2)–C(3) bond and of the *E/Z* isomerization, a computational study was carried out: geometries and solvation energies were computed on the density functional theory (DFT) level (model chemistry: B3LYP/6-311+g­(d,p); SCRF = SMD, solvent = water), whereas single-point electronic energies were computed with the post-Hartree–Fock method (model chemistry: MP2/6-311+g­(d,p); more details are available in the Supporting Information, Tables S4–S6). In Figure 10 the energy diagrams are shown along with the computed structures of the intermediates.

**Figure 10:**
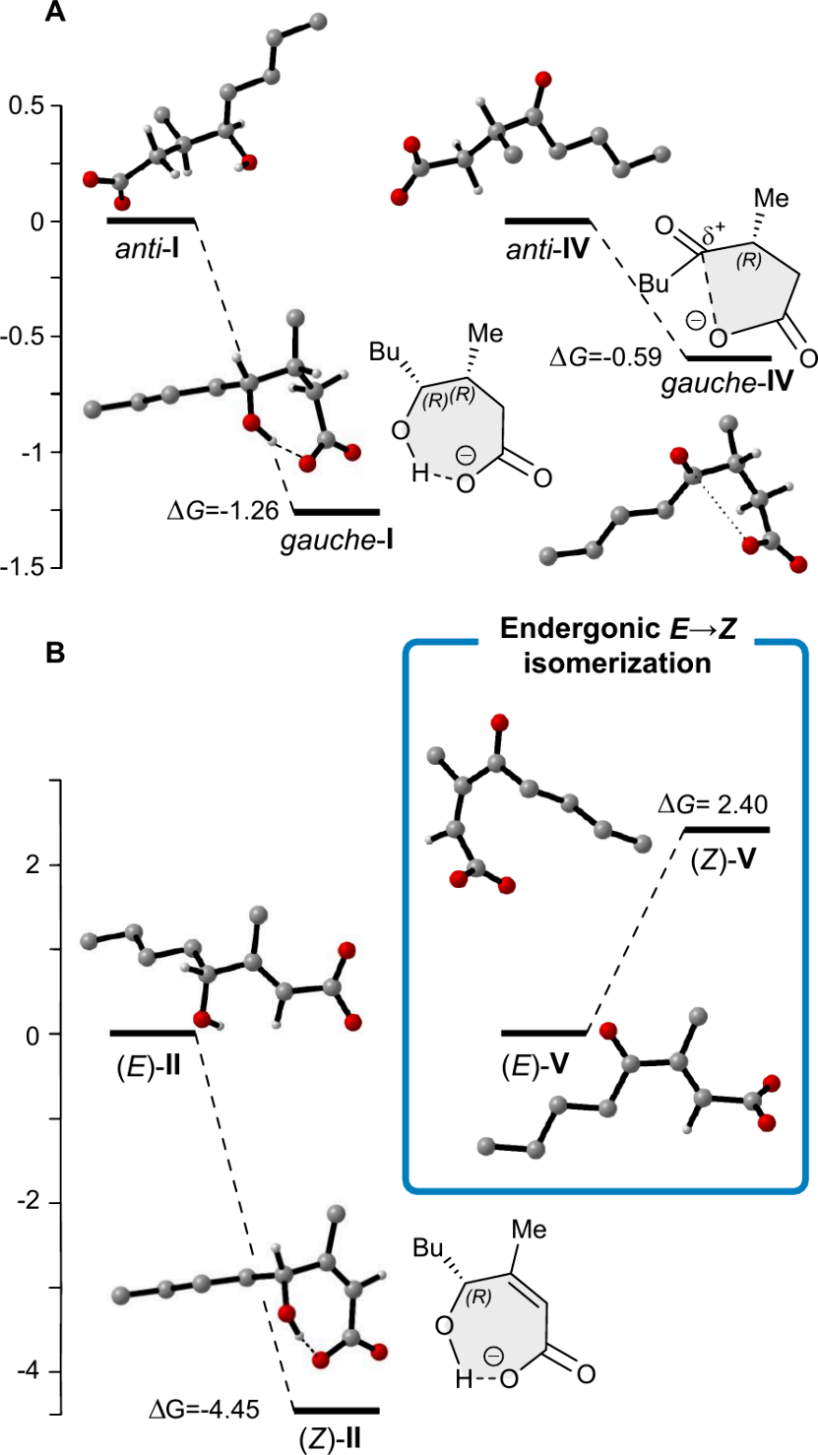
Optimized structures and energies (kcal mol^–1^) in water at 298 K. For clarity, some hydrogen atoms are omitted. (A) C(2)–C(3) *gauche/anti* equilibrium of **I** and **IV** intermediates. (B) *E → Z* isomerization of **II** and **V** intermediates.

Generally *anti* conformations are thermodynamically more stable than the *gauche* conformations since they adopt a geometry that minimizes the steric repulsions between the substituents. However, our computations indicate that both conformational equilibriums are slightly shifted toward the *gauche* forms (Δ*G* = −1.26 kcal mol^–1^ for **I** and Δ*G* = −0.59 kcal mol^–1^ for **IV**). Very likely, the *gauche* conformation of **I** is more stable than the *anti*, due to the intramolecular H-bond between the carboxylate and the hydroxyl group (CO_2_^–^···HO, Figure 10A). According to the Grabowsky classification, H-bonds with donor···acceptor distances between 2.45 and 2.55 Å and a bond angle of 180° are defined as being very strong and have energies exceeding 15 kcal mol^–1^.^32^ In this case, the H-bond distance of 2.60 Å with an angle of 168° allowed us to classify it as moderate strength, but still strong to offset the negative steric repulsions, typically occurring in the *gauche* rotamers. Similarly, the *gauche* conformer of **IV** was more stable than the *anti*, but in this case likely due to an electrostatic interaction between the electrophilic sp^2^ carbon of ketone and the negatively charged oxygen of carboxylate (Figure 10A).

The *E* configuration of trisubstituted olefins is usually the most stable, but for the hydroxy carboxylate **II**, the *Z* diastereoisomer is significantly more stable than the *E* isomer (Δ*G*_*E*→*Z*_ =– 4.45 kcal mol^–1^); once again this was likely due to the strong intramolecular H-bond (H-bond distance 2.56 Å, Figure 10B). In contrast, for keto intermediate **V**, the *E*/*Z* stability order is reversed (Δ x*G*_*E*→*Z*_ = 2.40 kcal mol^–1^). Indeed, the absence of an internal H-bond allows steric effects to be predominant, outweighing other possible stabilizing electronic interactions (Figure 10B).

This last result highlights another key feature of the very complex stereochemical dynamic process occurring in the desaturation of **1a**: the thermodynamically unfavorable *E → Z* isomerization of intermediate **V** catalyzed by ERs, which was observed also in the biotransformation of substrates **5a** and **6a**. These kinds of transformations, also known as contra-thermodynamic isomerizations,^33^ can take place if they are coupled with transient intermediates triggered by an external stimulus (usually light), an additive or a template that shifts the equilibrium. In our case, endergonic isomerization occurs because only the *Z* isomer of **III** (transient intermediate) can undergo ring-closure to form the unsaturated lactone, making the last step the true driving force of the entire process (thermodynamic sink via irreversible lactonization).

### Established Biosynthetic Pathways

Although most intermediates could not be directly observed, the combined isotopic and kinetic evidence consistently supports that *R. erythropolis* whole cells catalyze the desaturation of 4,5-disubstituted γ-lactones through a multistep process (paths B and C) rather than a single enzymatic step (path A). Initially, the lactone is enzymatically hydrolyzed to the hydroxy carboxylate **II**, which then is transformed through a sequence of redox steps, plausibly catalyzed by ERs, KRs, and ADHs, into the transient intermediate *Z*-hydroxy carboxylic acid **III**. The latter plays a crucial role, since as soon as it is formed, it spontaneously undergoes ring-closure to produce the very stable unsaturated γ-lactone **3** driving the entire multistep process. Remarkably, regardless of the type of substrate supplied to the microorganism (γ-lactone, 1,4-diol, keto ester, α,β-unsaturated γ-hydroxyester, and enone with *E* configuration, and even whisky lactone with the stereochemical configuration at C(5) opposite that of the final product), the biotransformation chemo- and enantioconverges invariably to the formation of the unsaturated lactone with (*R*) absolute stereochemical configuration.

The high *ee* values of products cannot be explained by a simple kinetic asymmetric transformation alone; therefore, the C(4) de-epimerization of **II** must necessarily be involved. Therefore, the desaturation of all intermediates seems to be not completely *Z*-diastereoselective, meaning that the *E* intermediates undergo C=C double-bond isomerization, which is very likely catalyzed by ERs.

Lastly, concerning the desaturation step, to the knowledge of the authors, no enzymes with such specific activity have been isolated or characterized in whole cells of *Rhodococcus erythropolis*. However, this clarification does not affect the validity of the proposed biosynthetic paths.

### Formal Synthesis of Forskolin

In the final stage of this work, we show the synthetic utility of the dynamic enantioconvergent desaturation of 4,5-disubstituted γ-lactones in the preparation of key intermediate **8** (Figure 11A). This aldehyde has previously been reported by Ikegami et al. in the total synthesis of racemic forskolin.^34^

**Figure 11:**
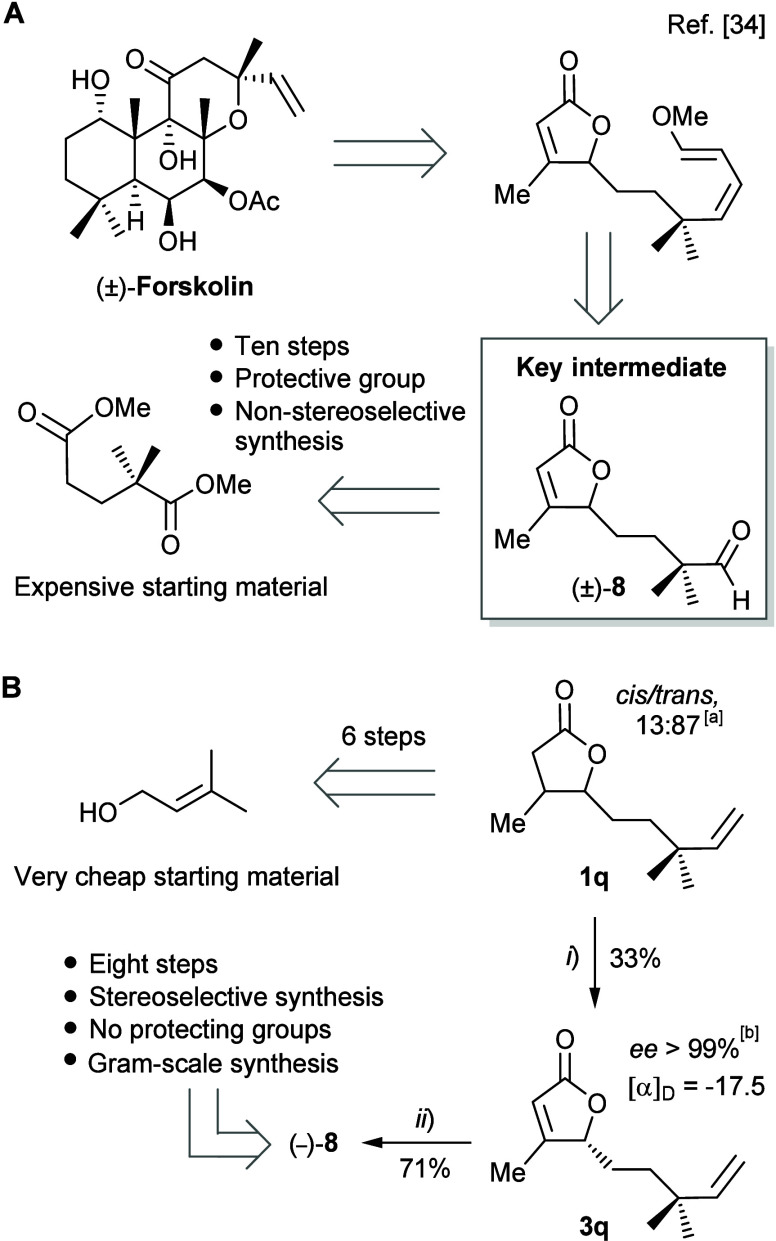
(A) Partial retrosynthesis of (±)-forskolin according to Ikegami. (B) Synthesis of key intermediate **8**. Reaction conditions: (*i*) *R. erythropolis* DSM 44534, [**1q**] = 0.33 g L^–1^, 26 °C; (*ii*) (a) K_2_OsO_4_·2H_2_O cat., NMO, H_2_O/acetone, rt; (b) NaIO_4_, THF, rt. ^a^By GC-MS. ^b^By chiral GC.

Forskolin is a complex natural product that has long attracted interest as a synthetic target^34,35^ due to its unique and biologically promising profile, especially in anticancer therapy.^36^

According to our retrosynthetic analysis (Scheme S5), substrate **1q** was obtained in six steps, starting from the commercially available prenyl alcohol. Biodesaturation of **1q** in a bioreactor gave the dehydrogenated lactone **3q** in 33% yield after chromatographic purification, with a high enantioselectivity (*ee* > 99%), consistent with that achieved in the biotransformation of similar *Quercus*-like lactones (Figure 11B). A subsequent two-step sequence converted **3q** into aldehyde (−)-**8** in 71% overall yield. The sequence comprised a regioselective dihydroxylation of the terminal alkene of **3q** using the Upjohn protocol,^37^ followed by oxidative cleavage of the diol intermediate with sodium periodate (Figure 11B).

This new synthesis of the forskolin key intermediate **8** offers several advantages over the previous route: it employs a significantly shorter sequence (eight vs ten steps), it avoids the use of protecting groups, the starting material is very cheap (prenyl alcohol vs 2,2-dimethylglutaric acid), and it delivers the product with a high enantioselectivity.

## Conclusions

The α,β dehydrogenation of esters is one of the most challenging transformations of organic chemistry, which often requires nontrivial reaction conditions and catalysts. In this regard the *R. erythropolis*-catalyzed desaturation of the naturally occurring *Quercus* lactones opens an appealing perspective for the development of more sustainable methodologies, with the setup of this biotransformation being very simple, implementable at gram-scale, and not requiring the use of expensive nicotinamide-based cofactors and their recycling systems. However, beyond its green chemistry implications, *R. erythropolis* provides two unique features that would be very hard to replicate in a catalytic system using traditional methodologies: *i*) the site-selective de-epimerization of secondary alcohols by a concurrent tandem oxidation and reduction catalyzed by ADHs and KRs, respectively; *ii*) the dynamic *E* → *Z* isomerization by a concurrent tandem saturation and desaturation process catalyzed by ER-like enzymes. These capabilities available within whole cells of *R. erythropolis* enabled the dynamic enantioconvergent desaturation of 4,5-disubstituted γ-lactones affording unsaturated lactones with a very high level of enantioselectivity, thus establishing a rare example of biocatalytic late-stage stereochemical editing^38^ applied to a complex terpenoid scaffold, as showcased by the dynamic desymmetrization of the forskolin precursor **1q**.

## Experimental Section

All experimental procedures, spectroscopic data, chromatograms, and computational details are provided in the Supporting Information.

## Supplementary Material



## Notes

Preparation and characterization of products; copies of chiral GC chromatograms and ^1^H, ^2^H, and ^13^C NMR spectra together with energies and geometries of computed structures (PDF)
