# Supplementary material for: Dynamic Enantioconvergent Desaturation of 4,5-Disubstituted γ‑Lactones in Whole Cells of Rhodococcus erythropolis
Source: J Am Chem Soc. 2025 Dec 29;148(1):1779–89. doi: 10.1021/jacs.5c19136 (PMC12814362; doi:10.1021/jacs.5c19136)
Supplement: Supplementary file 1 [file ja5c19136_si_001.pdf]

Dynamic Enantioconvergent Desaturation of 4,5-Disubstituted γ-Lactones in Whole Cells of *Rhodococcus erythropolis*

Maria C. Cancellieri,[a] Filip Boratyński,[b] Stefano Serra,[c,*] Dawid Hernik,[b] and Francesco G. Gatti[a,*]

a) Department of Chemistry, Materials and Chemical Engineering "Giulio Natta", Politecnico di Milano, P.zza L. da Vinci 32, Milano, 20133, Italy

b) Department of Food Chemistry and Biocatalysis, Wrocław University of Environmental and Life Sciences, Norwida 25, 50-375 Wrocław, Poland

c) Consiglio Nazionale delle Ricerche, Istituto di Scienze e Tecnologie Chimiche, Via Mancinelli 7, 20131 Milan, Italy

* Corresponding authors:

Dr. Stefano Serra

Telephone: +39 02 23993076, E-mail: stefano.serra@cnr.it

Prof. Francesco Gilberto Gatti

Telephone: +39 02 23993070-72, E-mail: francesco.gatti@polimi.it

Table of Contents

General information 5

Microorganism and growth medium 5

Synthesis of γ-hydroxyesters (**5a** and **5l**) and 4,5-disubstituted γ-lactones (**1a-h** and **1l**) 6

Scheme S1 6

Synthesis of aldehyde **9** and allylic alcohol **5l** 7

Ethyl 3-methyl-4-oxobutanoate (**9**) 7

Ethyl (*E*)-4-hydroxy-3-methylbut-2-enoate (**5l**) 7

General procedures for the synthesis of substrates **5a**, **1a-h** and **1l** 7

Ethyl (*E*)-4-hydroxy-3-methyloct-2-enoate (**5a**) 8

5-Butyl-4-methyldihydrofuran-2(3*H*)-one (**1a**) 8

Ethyl (*E*)-4-hydroxy-3-methylhept-2-enoate (**5b**) 8

4-Methyl-5-propyldihydrofuran-2(3*H*)-one (**1b**) 8

Ethyl (*E*)-4-hydroxy-3,5-dimethylhex-2-enoate (**5c**) 9

5-Isopropyl-4-methyldihydrofuran-2(3*H*)-one (**1c**) 9

Ethyl (*E*)-4-hydroxy-3,6-dimethylhept-2-enoate (**5d**) 9

5-Isobutyl-4-methyldihydrofuran-2(3*H*)-one (**1d**) 9

Ethyl (*E*)-4-hydroxy-3-methylnon-2-enoate (**5e**) 10

4-Methyl-5-pentyldihydrofuran-2(3*H*)-one (**1e**) 10

Ethyl (*E*)-4-hydroxy-3,7-dimethyloct-2-enoate (**5f**) 10

5-Isopentyl-4-methyldihydrofuran-2(3*H*)-one (**1f**) 10

Ethyl (*E*)-4-hydroxy-3-dimethyldec-2-enoate (**5g**) 10

5-Hexyl-4-methyldihydrofuran-2(3*H*)-one (**1g**) 11

Ethyl (*E*)-4-hydroxy-3-methylhepta-2,6-dienoate (**5h**) 11

5-Allyl-4-methyldihydrofuran-2(3*H*)-one (**1h**) 11

4-Methyldihydrofuran-2(3H)-one (**1l**) 11

Synthesis of Eldanolide (**1i**) 12

Scheme S2 12

Ethyl 3-methylpent-4-enoate (**10**) 12

Ethyl 3-(oxiran-2-yl)butanoate (**11**) 12

4-Methyl-5-(3-methylbut-2-en-1-yl)dihydrofuran-2(3*H*)-one (**1i**) 13

Synthesis of 5-butyl-3-methyldihydrofuran-2(3*H*)-one (**1m**) 14

Scheme S3 14

Synthesis of (*R*)-5-methyl-5-(4-methylpentyl)dihydrofuran-2(3H)-one (**1o**) 15

Scheme S4. 15

(*R*)-5-Methyl-5-(4-methylpent-3-en-1-yl)furan-2(5H)-one (**12**) 15

(*R*)-5-Methyl-5-(4-methylpentyl)dihydrofuran-2(3H)-one (**1o**) 15

Synthesis of substrate precursor of forskolin (**1q**) 17

Scheme S5. 17
Ethyl 3,3-dimethylpent-4-enoate (**13**) 18
3,3-Dimethylpent-4-en-1-ol (**14**) 18
5-Bromo-3,3-dimethylpent-1-ene (**15**) 18
3,3,8,8-Tetramethyldeca-1,9-diene (**16**) 18
5-Iodo-3,3-dimethylpent-1-ene (**17**) 19
Ethyl (*E*)-4-hydroxy-3,7,7-trimethylnona-2,8-dienoate (**5q**) 19
5-(3,3-Dimethylpent-4-en-1-yl)-4-methyldihydrofuran-2(3*H*)-one (**1q**) 19
Synthesis of 3,4-dehydrogenated γ-lactones as reference for chiral GC analysis (**3a-q**) 21
Scheme S6 21
General procedure for synthetic route A 21
5-Butyl-4-methylfuran-2(5*H*)-one (**3a**) 21
General procedure for synthetic route B 22
4-Methyl-5-propylfuran-2(5H)-one (**3b**) 22
5-Isopropyl-4-methylfuran-2(5*H*)-one (**3c**) 22
5-Isobutyl-4-methylfuran-2(5H)-one (**3d**) 22
4-Methyl-5-pentylfuran-2(5H)-one (**3e**) 22
5-Isopentyl-4-methylfuran-2(5*H*)-one (**3f**) 23
5-Hexyl-4-methylfuran-2(5*H*)-one (**3g**) 23
5-Allyl-4-methylfuran-2(5*H*)-one (**3h**) 23
4-Methylfuran-2(5*H*)-one (**3l**) 23
5-(3,3-Dimethylpent-4-en-1-yl)-4-methylfuran-2(5*H*)-one (**3q**) 24
General procedure for biodesaturation in an Erlenmeyer flask (optimized conditions) 25
Table S1 25
Growth inhibition profile as a function of lactone concentration 25
Control biotransformations: absence of microorganisms or inactivated (autoclaved) cells 26
(*R*)-5-Butyl-4-methylfuran-2(5*H*)-one (**3a**) 27
(*R*)-4-Methyl-5-propylfuran-2(5*H*)-one (**3b**) 27
(*R*)-5-Isobutyl-4-methylfuran-2(5*H*)-one (**3d**) 27
(*R*)-4-Methyl-5-pentylfuran-2(5*H*)-one (**3e**) 27
(*R*)-5-Isopentyl-4-methylfuran-2(5*H*)-one (**3f**) 27
(*R*)-5-Hexyl-4-methylfuran-2(5*H*)-one (**3g**) 28
(*R*)-4-Methyl-5-(3-methylbut-2-en-1-yl)furan-2(5*H*)-one (**3i**) 28
(*R*)-5-(3,3-dimethylpent-4-en-1-yl)-4-methylfuran-2(5*H*)-one (**3q**) 28
General procedure for biodesaturation in a bioreactor 29
(*R*)-5-Butyl-4-methylfuran-2(5*H*)-one (**3a**) 29
(*R*)-4-Methyl-5-pentylfuran-2(5*H*)-one (**3e**) 29
(*R*)-4-Methyl-5-pentylfuran-2(5*H*)-one (**3q**) 29

Hydrogenation of (5*R*)-**3a** and (5*R*)-**3e** for the determination of absolute stereochemical configuration....... 30
Scheme S7 30
General procedure 30
(+)-*cis*-Whisky lactone: (4*R*,5*R*)-5-Butyl-4-methyldihydrofuran-2(3*H*)-one ((4*R*,5*R*)-**1a**) 30
(+)-*cis*-Cognac lactone: (4*R*,5*R*)-5-Pentyl-4-methyldihydrofuran-2(3*H*)-one ((4*R*,5*R*)-**1e**) 30
Monitoring enzymatic hydrolysis 31
Table S2 31
Derivatization of intermediate I as methylester (**4a**) 31
Synthesis of deuterated substrates 32
Synthesis of **2a**-1,1,4-d_3 and **1a**-5-*d* 32
Scheme S8 32
Isopropyl 3-methyl-4-oxooctanoate (**7a**) 32
3-Methyloctane-1,1,4-d_3-1,4-diol (**2a**-1,1,4-d_3) 32
5-Butyl-4-methyldihydrofuran-2(3*H*)-one-5-*d* (**1a**-5-*d*) 33
Synthesis of 5-butyl-4-methyldihydrofuran-2(3*H*)-one-3,4-d_2 (**1a**-3,4-d_2) 33
Scheme S9 33
(3*S*,4*S*,5*R*)-5-Butyl-4-methyldihydrofuran-2(3*H*)-one-3,4-d_2 (*trans*-**1a**-3,4-d_2) 34
(3*R*,4*R*,5*R*)-5-Butyl-4-methyldihydrofuran-2(3*H*)-one-3,4-d_2 (*cis*-**1a**-3,4-d_2) 34
Biodesaturation of deuterium labeled substrates or of **3a** in D_2O/water medium 35
Table S3 35
Synthesis of intermediate **6a** 37
Scheme S10 37
Ethyl (*E*)-3-methyl-4-oxooct-2-enoate (**6a**) 37
Synthesis of enantiomerically enriched (4*R*,5*S*)-**1a** 37
Chemical hydrolysis of **1a** and H/D exchange monitoring at α-position by ^{1}H-NMR 38
Figure S1 38
(*R*)-2,2-Dimethyl-4-(3-methyl-5-oxo-2,5-dihydrofuran-2-yl)butanal (**8**) 39
Computational study 40
Table S4 40
Table S5 40
Table S6 41
Coordinates of computed species 41
Copies of Chiral GC chromatograms 46
Chiral GC chromatograms of biotransformation of (5*S*)-**1a** enantiomerically enriched stereoisomers 61
Copies of ^{1}H and ^{13}C NMR spectra 62
References 115

General information

Chemicals (including substrates **1n** and **1p**) and solvents were purchased from suppliers and used without further purification, while, where required, the solvents were dried over molecular sieves (4 Å) for 1 day before their use. Reductions of C=C double bonds with D_2 (*in situ* generated by electrolysis of D_2O, purity>99%) were carried on a high-pressure continuous flow hydrogenation-reactor (H-Cube from ThalesNano) equipped with a Pd/C cartridge (5% wt. loading). Classical catalytic hydrogenations were carried out with Pd/C (10% wt. loading) or Rh/Al_2O_3 (0.5% wt. loading). Photoisomerization was carried out on a photoreactor equipped with 12 UV lamps (8 W each). ^1H, ^{13}C and ^2H NMR spectra were recorded on a 400 MHz spectrometer at 302 K using $CDCl_3$ or $CHCl_3$ + 20 µL $CDCl_3$ as solvent for the ^2H-spectra. Chemical shifts (δ) are expressed in ppm relative to the tetramethylsilane (TMS) signal or to the residual $CHCl_3$ signal (δ = 7.26 ppm for ^1H, δ = 77.16 ppm for ^{13}C and δ =7 .26 ppm for ^2H). $^{13}C\{^1H\}$-NMR spectra were acquired under ^1H-decoupling conditions, and an Attached Proton Test (APT) ^{13}C NMR experiment was performed to differentiate between CH, CH_3, CH_2, and C_q quaternary carbon signals. ^2H-NMR spectra were measured on deuterium lock channel using a standard 90° pulse, the magnetic field homogeneity was optimized on FID (free induction decay) area. High-resolution MS spectra were recorded with a Q-TOF mass spectrometer, equipped with an ESI source. The GC-MS analyses of all compounds were performed on a column with a low polarity stationary phase (30 m x 0.25 mm x 0.25 µm). Program temperature: 60 °C (1 min)/6 °C min^{-1}/150 °C (1 min)/12 °C min^{-1}/280 °C (5 min). Chiral GC analyses for compounds **1a-b**, **1d**, **1e-g**, **1l, 3a-b**, **3d**, **3e-g**, **3l** were performed on a CP7502 Chirasil-DEX CB column (25 m x 0.25 mm x 0.25 µm); program temperature: 80 °C (1 min)/5 °C min^{-1}/150 °C (1 min)/60 °C min^{-1}/180 °C (2 min). Chiral GC analyses for compounds **1q**, **3q** were performed on the same column but with a slightly different program: 80 °C (1 min)/5 °C min^{-1}/170 °C/40 °C min^{-1}/200 °C (1 min). Optical rotation values $[\alpha]_D$ were measured on a digital automatic polarimeter at λ=589 nm (sodium *D* line) and are given in ° cm^3 g^{-1} dm^{-1}, the measures were carried out at 20 °C temperature. The optical density (OD_{600}) was measured on a UV-Vis spectrophotometer λ=600 nm. TLC analyses were performed on precoated silica gel 60 F_{254} plates, and spots were visualized either by UV light (254 nm) or by spraying with phosphomolybdic acid reagent or by spraying with potassium permanganate reagent. All gravimetric chromatographic separations were carried out on silica gel columns (230-400 mesh). The distillations were performed on a bulb-to-bulb Kugelrohr like apparatus connected to a high *vacuum* pump (0.1-0.2 mmHg).

Microorganism and growth medium

Casein peptone, peptone from meat, yeast extract, glucose and NaOAc were of commercial quality and were purchased from suppliers and used without further purification. Riboflavin (98% purity) and marine salt (purchased from a local market) were used as received. *Rhodococcus erythropolis* (DSM 44534). *Rhodococcus erythropolis* medium: glucose (10 g/L), marine salt (5 g/L), casein peptone (4 g/L), peptone from meat (10 g/L), yeast extract (3 g/L), NaOAc (2 g/L), trace elements solution (10 mL/L), riboflavin (10 mg/L). Trace elements solution: $FeCl_3$ (50 mM), $CaCl_2$ (20 mM), $MnCl_2$ (10 mM), $ZnSO_4$ (10 mM), $CoCl_2$ (2 mM), $CuCl_2$ (2 mM), $NiCl_2$ (2 mM), Na_2MoO_4 (2 mM), Na_2SeO_3 (2 mM), H_3BO_3 (2 mM). Then, the medium was autoclaved at 121 °C for 15 min; final pH 6.5. The biodesaturation at mg-scale was carried out on an Erlenmeyer flask shaker equipped with temperature and speed controller. The biodesaturation at g-scale was carried out on a 5 L bioreactor-fermenter equipped with pH, temperature and air controller.

Synthesis of γ-hydroxyesters (5a and 5l) and 4,5-disubstituted γ-lactones (1a-h and 1l)

The 4,5-disubstituted γ-lactones (**1a-g**) were synthesized from the commercially available ethyl *trans* 3-methyl-4-oxocrotonate (*E*>99%) through a three-step telescopic reaction sequence (Scheme S1). In the first step a freshly prepared Grignard reagent was added to the carbonyl aldehyde of *trans*-formyl crotonate yielding the α,β-unsaturated γ-hydroxyester intermediate (**5a-g**) of sufficient purity for the next step and without the loss of the initial *de*. In the second step the C=C double bond was reduced by catalytic hydrogenation using Pd/C as catalyst. In the last step, the hydroxyester intermediate was ring-closed to the corresponding lactone **1a-g** by treatment with a catalytic amount of trifluoroacetic acid (TFA). Then, the lactone was purified by column chromatography, followed by bulb-to-bulb distillation to obtain the corresponding lactone. Lactone **1h** was prepared following a similar synthetic route. First, the hydrogenation of ethyl *trans* 3-methyl-4-oxocrotonate catalyzed by Pd/C at low temperature (-10 °C) in EtOAc gave a mixture of aldehyde **9** and allylic alcohol **5l** with a moderate chemoselectivity (3:1 by GC-MS). The two products were separated by distillation under reduced pressure. Subsequent addition of allyl magnesium bromide to aldehyde **9** afforded the corresponding intermediate alcohol, which was cyclized to lactone **1h** by treatment with TFA (Scheme S1). Lactone **1l** was prepared by TFA catalyzed lactonization of hydroxyester **5l**. Both lactones were purified using the same procedure as for lactones **1a-g**.

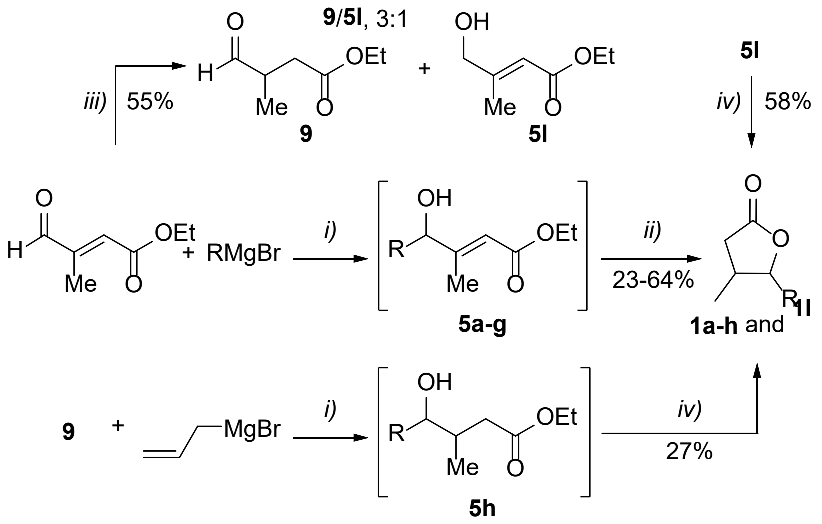

Alcohol	Lactone	R
**5a**[a]	**1a**	Bu
**5b**[b]	**1b**	Pr
**5c**[b]	**1c**	^{i}Pr
**5d**[b]	**1d**	^{i}Bu
**5e**[b]	**1e**	Pent
**5f**[b]	**1f**	^{i}Pent
**5g**[b]	**1g**	Hex
**5h**[b]	**1h**	All
**5l**[a]	**1l**	H

(a) purified and NMR characterized; (b) not purified

Scheme S1. Reaction conditions: *i*) in THF, at -78 °C. *ii*): a) H_2, Pd/C (substrate/catalyst 10% w/w), EtOAc, rt; b) TFA cat., CH_2Cl_2, 0 °C to rt. *iii*) H_2, Pd/C (substrate/catalyst 10% w/w), -10 °C. *iv)* TFA cat., CH_2Cl_2, 0 °C to rt.

Synthesis of aldehyde 9 and allylic alcohol 5l

To a solution of ethyl *trans*-3-methyl-4-oxocrotonate (0.2 mol, 28.8 g) in EtOAc (200 mL), a sub-stoichiometric amount of Pd/C (5% w/w relative to the substrate) was added. The reaction mixture was cooled to -10 °C, mechanically stirred, and maintained under a hydrogen atmosphere until complete consumption of the starting material, as monitored by GC-MS. The mixture was then filtered through a celite pad, which was subsequently washed with pentane (3 x 30 mL). The combined filtrates were concentrated under reduced pressure, and the residue was subjected to distillation under reduced pressure (14 mbar) through a Vigreux-column (15 cm) to afford, in sequence, aldehyde **9** (T= 65-67 °C) and alcohol **5l** (T=112°C).

Ethyl 3-methyl-4-oxobutanoate (9)

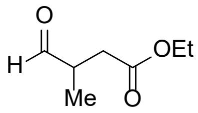

9

Yield 41% (11.8 g) as a colorless liquid; 95% purity by GC-MS, t_R=7.3 min; **^{1}H-NMR** ($CDCl_3$, 400 MHz): δ 9.69 (d, J=0.7 Hz, 1H), 4.14 (q, J=7.1 Hz, 2H), 2.84 (m, 1H), 2.71 (dd, J=16.4, 7.0 Hz, 1H), 2.36 (dd, J=16.4, 6.4 Hz, 1H), 1.25 (t, J=7.1 Hz, 3H), 1.18 (d, J=7.3 Hz, 3H); **^{13}C{^{1}H}-NMR** ($CDCl_3$, 101 MHz): δ 202.8, 171.9, 60.9, 42.7, 35.1, 14.3, 13.5; GC-MS: m/z (%) 116 (M^+-28, 70), 98(100), 88(32), 73(100). The spectroscopic data were consistent with those reported in literature.[1]

Ethyl (*E*)-4-hydroxy-3-methylbut-2-enoate (5l)

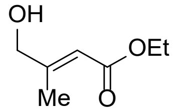

5l

Yield 14% (4.0 g) as a colorless liquid; 94% purity by GC-MS, t_R=11.1 min, *E*>99% by ^{1}H-NMR; **^{1}H-NMR** ($CDCl_3$, 400 MHz): δ 5.98 (q, J=1.5 Hz, 1H), 4.13-4.21 (m, 2H+2H), 2.13 (dt, J=1.3 and 0.7 Hz, 3H), 1.29 (t, J=7.1 Hz, 3H); **^{13}C{^{1}H}-NMR** ($CDCl_3$, 101 MHz): δ 167.0, 157.3, 113.9, 67.2, 59.9, 15.7, 14.4; GC-MS: m/z (%) 144(M^+,10), 126(15), 115(23), 98(100). The spectroscopic data were consistent with those reported in literature.[2]

General procedures for the synthesis of substrates 5a, 1a-h and 1l

Grignard addition. To a well stirred solution of aldehyde (ethyl *trans* 3-methyl-4-oxocrotonate or **9**; 10 mmol) in THF (40 mL) was dropwise added a solution of RMgBr (12 mmol) in Et_2O (60 mL) at -78 °C (prepared following typical preparation procedures), usually over 30 minutes, and let to stir for 1 hour. Then the reaction mixture was left to reach -50 °C, and after 2 hours it was quenched with a solution of NH_4Cl (sat., 50 mL) under vigorous stirring. After 1 hour the reaction mixture was extracted with EtOAc (3 x 50 mL). The combined organic phase was extracted with brine (sat., 50 mL), dried over anhydrous Na_2SO_4. The solvent was removed under *vacuum* affording crude material as pale-yellow oil. The latter was submitted to bulb-to-bulb distillation to eliminate the starting material and other volatile side-products affording the hydroxyester intermediate (**5a-h**), which was of sufficient purity to be submitted to the next step, except for hydroxyester **5a**. Since hydroxyester **5a** was used as substrate in the biotransformation, it was purified by column chromatography using *n*-hexane/EtOAc (70:30) as eluent.[3]

Hydrogenation. To a solution of α,β-unsaturated hydroxyester (**5a-g**) in EtOAc (30 mL) was added a sub-stoichiometric amount of Pd/C (substrate/catalyst 10% w/w). Then, the reaction mixture was well stirred and kept under an H_2 atmosphere until the complete conversion of starting material (checked by GC-MS). Subsequently, the mixture was filtered on a celite pad, which was washed with EtOAc (3 x 10 mL). The organic phase was dried over anhydrous Na_2SO_4 and concentrated under reduced pressure. The crude hydrogenation product was subjected directly to acid-catalyzed ring closure without further purification.

Acid catalyzed ring-closure. To an ice-cooled solution of hydroxyester (product of hydrogenation or **5h)** in CH_2Cl_2 (10 mL) was added TFA (100 μL) and left to stir at room temperature. After 12 hours, the mixture was ice-cooled and quenched with a solution of $NaHCO_3$ (sat., 30 mL) and left to stir over 30 minutes at room temperature. The aqueous phase was extracted with CH_2Cl_2 (2 x 30 mL). The combined organic phase was extracted with brine (sat., 30 mL), dried over anhydrous Na_2SO_4 and concentrated under *vacuum* affording the lactone (**1a-h** and **1l**). The latter was purified by silica gel column chromatography using *n*-hexane/EtOAc (80:20) as eluent, except for substrate **1l** which was chromatographed using *n*-pentane/Et_2O (4:6) as eluent. Then the lactone was distilled under *vacuum* (0.2-0.3 bar) by bulb-to-bulb apparatus.

Ethyl (*E*)-4-hydroxy-3-methyloct-2-enoate (5a)

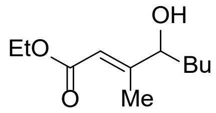

5a

Yield 75% (1.52 g) as a colorless oil; 96% purity by GC-MS, t_R=18.3 min, *E*>99% by ^{1}H-NMR; **^{1}H-NMR** ($CDCl_3$, 400 MHz): δ 5.88 (m, 1H), 4.15 (q, J=7.2 Hz, 2H), 4.06 (t, J=6.3 Hz, 1H), 2.09 (d, J=1.4 Hz, 3H), 1.62 (m, 1H), 1.50 (m, 1H), 1.38-1.30 (m, 1H+2H), 1.29-1.24 (m, 1H+3H), 0.88 (m, 3H); **^{13}C{^{1}H}-NMR** ($CDCl_3$, 101 MHz): δ 167.0, 160.4, 115.2, 76.8, 59.9, 34.7, 27.7, 22.7, 14.9, 14.4, 14.1; GC-MS: m/z (%) 200(M^+,1), 143(80), 115(100), 87(63). The spectroscopic data were consistent with those reported in literature.[4]

5-Butyl-4-methyldihydrofuran-2(3*H*)-one (1a)

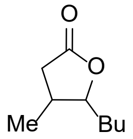

1a

Yield 64% (1.02 g) as a colorless liquid; >99% purity by GC-MS, t_R=13.7 min (*trans*) and 14.4 min (*cis*); *trans*/*cis*=54:46 by GC-MS and by ^{1}H-NMR; t_R=10.5 min (4*S*,5*R*), t_R=10.7 min (4*R*,5*S*), t_R=11.7 min (4*S*,5*S*) and t_R=11.9 min (4*R*,5*R*) by chiral GC; **^{1}H-NMR** ($CDCl_3$, 400 MHz): δ 4.42 (m, 0.46H), 4.00 (m, 0.54H), 2.69 (m, 1H), 2.57 (m, 0.46H), 2.23-2.12 (m, 0.54H+1H), 1.70-1.59 (m, 0.54H+1H), 1.55-1.45 (m, 0.46H+1H), 1.40-1.28 (m, 1H+2H), 1.13 (m, 1.62H), 1.00 (m, 1.38H), 0.91 (m, 3H); **^{13}C{^{1}H}-NMR** ($CDCl_3$, 101 MHz) (*trans*): δ 176.5, 87.4, 37.1, 36.0, 33.0, 27.8, 22.44, 17.4, 13.78; (*cis*): δ 176.9, 83.64, 37.5, 33.7, 29.5, 28.0, 22.48, 13.9, 13.84; GC-MS (*trans*): m/z (%) 156(M^+,2), 99(100), 87(17), 71(26); GC-MS (*cis*): m/z (%) 156(M^+,1), 99(100), 87(26), 69(33). The spectroscopic data were consistent with those reported in literature.[2]

Ethyl (*E*)-4-hydroxy-3-methylhept-2-enoate (5b)

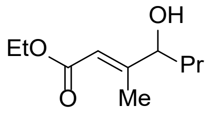

5b

Yield 64% (1.21 g) as a yellow liquid; 88% purity and *E*>99% by GC-MS, t_R=16.3 min; GC-MS: m/z (%) 168(M^+-18,1), 157(5), 143(78), 115(100).

4-Methyl-5-propyldihydrofuran-2(3*H*)-one (1b)

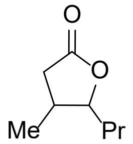

1b

Yield 58% (0.84 g) as a colorless liquid; >99% purity by GC-MS, t_R=11.3 min (*trans*) and 12.1 min (*cis*),; *trans*/*cis*=59:41 by GC-MS and by ^{1}H-NMR; t_R=8.1 min (4*S*,5*R*), t_R=8.5 min (4*R*,5*S*), t_R=9.4 min (4*S*,5*S*) and t_R=9.5 min (4*R*,5*R*) by chiral GC; **^{1}H-NMR** ($CDCl_3$, 400 MHz): δ 4.43 (m, 0.41H), 4.00 (td, J=7.6, 4.1 Hz, 0.59H), 2.74-2.50 (m, 0.41H+1H), 2.25-2.11 (m, 0.59H+1H), 1.69-1.34 (m, 2H+2H), 1.13 (m, 1.77H), 1.00 (d, J=7.0 Hz, 1.23H), 0.95 (m, 3H); **^{13}C{^{1}H}-NMR** ($CDCl_3$, 101 MHz) (*trans*): δ 176.7, 87.3, 37.2, 36.3, 36.2, 19.2, 17.57, 13.96; (*cis*): δ 177.0, 83.5, 37.7, 33.1, 32.1, 19.3, 17.57, 14.00; GC-MS (*trans*): m/z (%) 142(M^+,2), 99(100), 71(30), 55(23); GC-MS (*cis*): m/z (%) 142(M^+,1), 99(100), 71(43), 55(34). The spectroscopic data of *trans* diastereoisomer were consistent with those reported in literature.[5]

Ethyl (*E*)-4-hydroxy-3,5-dimethylhex-2-enoate (5c)

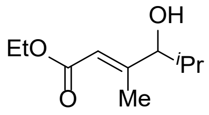

5c

Yield 76% (1.40 g) as a yellow liquid; 91% purity and *E*>99% by GC-MS, t_R=15.3 min; GC-MS: m/z (%) 186(M^+,1), 153(5), 143(100).

5-Isopropyl-4-methyldihydrofuran-2(3*H*)-one (1c)

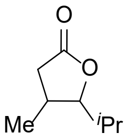

1c

Yield 64% (0.91 g) as a colorless liquid; 97% purity by GC-MS, t_R=11.4min (*trans*) and 11.9 min (*cis*); *trans*/*cis*=63:37 by GC-MS and by ^{1}H-NMR; t_R=7.4 min (4*S*,5*R*), t_R=7.7 min (4*R*,5*S*), t_R=8.3 min (4*S*,5*S*) and t_R=9.1 min (4*R*,5*R*) by chiral GC; **^{1}H-NMR** ($CDCl_3$, 400 MHz): δ 3.93 (dd, J=10.1, 4.7 Hz, 0.37H), 3.84 (dd, J=6.5, 5.6 Hz, 0.63H), 2.71 (m, 1H), 2.59-2.48 (m, 0.37H), 2.38-2.29 (m, 0.63H), 2.22-2.13 (m, 1H), 1.92-1.80 (m, 1H), 1.14 (d, J=6.7 Hz, 1.89H), 1.07 (d, J=6.4 Hz, 1.11H), 1.00-0.96 (m, 1.89H+3H), 0.89 (d, J=6.7 Hz, 1.11H); **^{13}C{^{1}H}-NMR** ($CDCl_3$, 101 MHz) (*trans*): δ 177.0, 92.3, 37.4, 32.6, 31.7, 19.3, 18.8, 17.4; (*cis*): δ 177.3, 89.3, 39.1, 32.4, 28.2, 20.2, 17.8, 13.5 GC-MS (*trans*): m/z (%) 142(M^+,2), 114(25), 99(100), 71(43); GC-MS (*cis*): m/z (%) 142(M^+,1), 114(24), 99(100), 71(41). The spectroscopic data of *trans* diastereoisomer were consistent with those reported in literature.[3] The spectroscopic data of *cis* diastereoisomer were consistent with those reported in literature.[6]

Ethyl (*E*)-4-hydroxy-3,6-dimethylhept-2-enoate (5d)

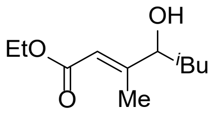

5d

Yield 42% (0.84 g) as a yellow liquid; 90% purity and *E*>99% by GC-MS, t_R=14.8 min; GC-MS: m/z (%) 200 (M^+,2), 143 (68), 115 (100).

5-Isobutyl-4-methyldihydrofuran-2(3*H*)-one (1d)

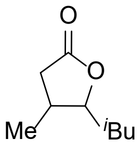

1d

Yield 35% (0.55 g) as a colorless liquid; 98% purity by GC-MS, t_R=13.3 min (*trans*) and 14.1 min (*cis*); *trans*/*cis*=56:44 by GC-MS and by ^{1}H-NMR; t_R=8.8 min (4*S*,5*R*), t_R=9.0 min (4*R*,5*S*), t_R=9.8 min (4*S*,5*S*) and t_R=10.0 min (4*R*,5*R*) by chiral GC; **^{1}H-NMR** ($CDCl_3$, 400 MHz): δ 4.56 (m, 0.44H), 4.01 (m, 0.56H), 2.73-2.51 (m, 0.44H+1H), 2.24-2.09 (m, 0.56H+1H), 1.83 (m, 1H), 1.58 (m, 1H), 1.43 (m, 0.56H), 1.28 (m, 0.44H), 1.12 (d, J=6.2 Hz, 1.68H), 1.00 (d, J=7.1 Hz, 1.32H), 0.95 (m, 3H+3H); **^{13}C{^{1}H}-NMR** ($CDCl_3$, 101 MHz) (*trans*): δ 176.6, 85.7, 43.2, 37.6, 36.7, 25.2, 23.27, 21.8, 17.2; (*cis*): δ 176.9, 81.8, 38.7, 37.0, 33.3, 25.0, 23.32, 21.9, 13.9; GC-MS (*trans*): m/z (%) 156(M^+,2), 99(100), 87(15), 71(31); GC-MS (*cis*): m/z (%) 156(M^+,1), 99(100), 87(20), 71(38). ESI-HRMS (m/z): calcd. for $C_9H_{16}NaO_2^+$ $[M+Na]^+$ 179.1043, found 179.1040.

Ethyl (*E*)-4-hydroxy-3-methylnon-2-enoate (5e)

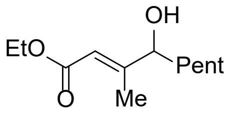

5e

Yield 54% (1.20 g) as a yellow liquid; 83% purity and *E*>99% by GC-MS, t_R=19.8 min; GC-MS: m/z (%) 196 (M^+-18,1), 185(5), 169(12), 143(70), 115 (100).

4-Methyl-5-pentyldihydrofuran-2(3*H*)-one (1e)

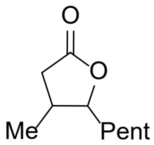

1e

Yield 25% (0.43 g) as a colorless liquid; t_R=15.9 min (*trans*) and 16.7 min (*cis*), >99% purity by GC-MS; *trans*/*cis*=58:42 by GC-MS and by ^{1}H-NMR; t_R=12.6 min (4*S*,5*R*), t_R=12.8 min (4*R*,5*S*), t_R=13.7 min (4*S*,5*S*) and t_R=13.9 min (4*R*,5*R*) by chiral GC; **^{1}H-NMR** ($CDCl_3$, 400 MHz): δ 4.43 (m, 0.42H), 4.00 (td, J=7.7, 4.1 Hz, 0.58H), 2.76-2.50 (m, 0.42H+1H), 2.29-2.12 (m, 0.58H+1H), 1.68-1.48 (m, 1H+2H), 1.38-1.24 (m, 1H+ 2H+2H), 1.45 (m, 1.74H), 1.01 (d, J=7.0 Hz, 1.26H), 0.90 (m, 3H); **^{13}C{^{1}H}-NMR** ($CDCl_3$, 101 MHz) (*trans*): δ 176.6, 87.5, 37.1, 36.0, 34.0, 31.5, 25.4, 22.4, 17.4, 13.9; (*cis*): δ 176.9, 83.7, 37.5, 33.0, 31.6, 29.8, 25.5, 22.5, 17.4, 13.8; GC-MS (*trans*): m/z (%) 170(M^+,2), 99(100), 71(29), 55(13); GC-MS (*cis*): m/z (%) 170(M^+,1), 99(100), 83(20), 71(23). The spectroscopic data were consistent with those reported in literature.[2]

Ethyl (*E*)-4-hydroxy-3,7-dimethyloct-2-enoate (5f)

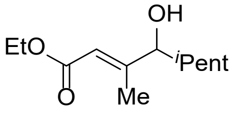

5f

Yield 33% (0.71 g) as a yellow liquid; 91% purity and *E*/Z 98:2 by GC-MS, t_R=19.2 min; GC-MS: m/z (%) 196(M^+-18,1), 185(2), 169(5), 143(82), 115 (100).

5-Isopentyl-4-methyldihydrofuran-2(3*H*)-one (1f)

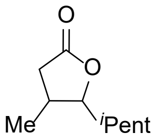

1f

Yield 24% (0.41 g) as colorless oil; 99% purity by GC-MS, t_R=15.1 min (*trans*) and 15.8 min (*cis*); *trans*/*cis*=45:55 by GC-MS and by ^{1}H-NMR; t_R=11.3 min (4*S*,5*R*), t_R=11.5 min (4*R*,5*S*), t_R=12.5 min (4*S*,5*S*) and t_R=12.6 min (4*R*,5*R*) by chiral GC; **^{1}H-NMR** ($CDCl_3$, 400 MHz): δ 4.40 (m, 0.55H), 4.97 (m, 0.45H), 2.76-2.62 (m, 1H), 2.58 (m, 0.55H), 2.25-2.13 (m, 0.45H+1H), 1.67-1.51 (m, 1H+2H), 1.39 (m, 1H), 1.29 (m, 1H), 1.13 (d, J=6.4 Hz, 1.35H), 1.01 (d, J=7.0 Hz, 1.65H), 0.90 (m, 3H+3H); **^{13}C{^{1}H}-NMR** ($CDCl_3$, 101 MHz) (*trans*): δ 176.7, 87.9, 37.3, 36.2, 34.8, 32.0, 28.0, 22.63, 22.5, 17.7; (*cis*): δ 177.0, 84.1, 37.8, 35.0, 33.2, 28.1, 27.9, 22.58, 22.5, 14.0; GC-MS (*trans*): m/z (%) 170(M^+,2), 99(100), 83(20), 71(30); GC-MS (*cis*): m/z (%) 170(M^+,1), 99(100), 83(22), 71(24). The spectroscopic data were consistent with those reported in literature.[7]

Ethyl (*E*)-4-hydroxy-3-dimethyldec-2-enoate (5g)

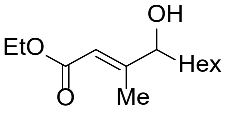

5g

Yield 46% (1.05 g) as a yellow oil; 87% purity and *E*/*Z* 97:3 by GC-MS, t_R=21.3 min; GC-MS: m/z (%) 210 (M^+-18,1), 199(5), 183(15), 143(76), 115(100).

5-Hexyl-4-methyldihydrofuran-2(3*H*)-one (1g)

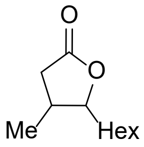

1g

Yield 33% (0.6 g) as a colorless oil; 95% purity by GC-MS, t_R=18.3 min (*trans*) and 19.0 min (*cis*); *trans*/*cis*=46:54 by GC-MS and by ^{1}H-NMR; t_R=14.6 min (4*S*,5*R*), t_R=14.8 min (4*R*,5*S*), t_R=15.6 min (4*S*,5*S*) and t_R=15.8 min (4*R*,5*R*) by chiral GC; **^{1}H-NMR** ($CDCl_3$, 400 MHz): δ 4.42 (m, 0.54H), 4.00 (td, J=7.7, 4.1 Hz, 0.46H), 2.77-2.50 (0.54H+1H), 2.28-2.11 (m, 0.46H+1H), 1.74-1.56 (m, 2H), 1.54-1.04 (m, 2H), 1.37-1.23 (m, 2H+2H+2H), 1.12 (m, 1.62H), 1.00 (d, J=7.0 Hz, 1.38H), 0.88 (m, 3H); **^{13}C{^{1}H}-NMR** ($CDCl_3$, 101 MHz) (*trans*): δ 176.7, 83.8, 37.3, 33.2, 31.76, 30.0, 29.19, 25.8, 22.65, 17.6, 14.0; (*cis*): δ 177.0, 87.6, 37.7, 36.2, 34.2, 31.78, 29.23, 26.0, 22.67, 17.6, 14.2; GC-MS (*trans*): m/z (%) 184(M^+,2), 142(18), 99(100), 71(21); GC-MS (*cis*): m/z (%) 184(M^+,1), 99(100), 71(22), 55(19). The spectroscopic data were consistent with those reported in literature.[8]

Ethyl (*E*)-4-hydroxy-3-methylhepta-2,6-dienoate (5h)

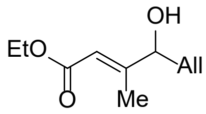

5h

Yield 35% (0.59 g) as a yellow liquid; 96% purity and *E*/*Z* 98:2 by GC-MS, t_R=15.7 min; GC-MS: m/z (%) 168 (M^+,1), 143 (100), 139 (10).

5-Allyl-4-methyldihydrofuran-2(3*H*)-one (1h)

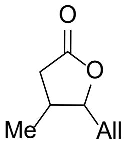

1h

Yield 27% (380 mg) as a colorless liquid; 96% purity by GC-MS; t_R=11.0 min (*trans*) and 11.8 min (*cis*), *trans*/*cis*=35:65 by GC-MS and ^{1}H-NMR; t_R=8.3 min (4*S*,5*R*), t_R=8.8 min (4*R*,5*S*), t_R=9.6 min (4*S*,5*S*) and t_R=9.7 min (4*R*,5*R*) by chiral GC; **^{1}H-NMR** ($CDCl_3$, 400 MHz): δ 5.82 (m, 1H), 5.21-5.12 (m, 2H), 4.48 (m, 0.65H), 4.12-4.04 (m, 0.35H), 2.69 (m, 1H), 2.60 (m, 0.65H), 2.51-2.28 (m, 2.35H), 2.19 (m, 1H), 1.14 (d, J=6.6 Hz, 1.05H), 1.04 (d, J=6.9 Hz, 1.95H); **^{13}C{^{1}H}-NMR** ($CDCl_3$, 101 MHz) (*trans*): δ 176.3, 132.6, 118.7, 86.2, 38.0, 37.0, 35.2, 17.6; (*cis*): δ 176.6, 133.0, 118.2, 82.6, 37.5, 34.4, 32.9, 14.0; GC-MS (*trans*): m/z (%) 140(M^+,2), 99(100), 71(43), 55(12); GC-MS (*cis*): m/z (%) 140(M^+,1), 99(100), 71(43), 55(12). The spectroscopic data were consistent with those reported in literature.[9]

4-Methyldihydrofuran-2(3H)-one (1l)

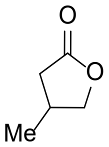

1l

Yield 58% (580 mg) as a colorless liquid; 97% purity by GC-MS, t_R=6.0 min; **^{1}H-NMR** ($CDCl_3$, 400 MHz): δ 4.41 (m, 1H), 3.87 (dd, J=8.8, 6.3 Hz, 1H), 2.70-2.60 (m, 1H+1H), 2.15 (m, 1H), 1.17 (d, J=6.6 Hz, 3H); **^{13}C{^{1}H}-NMR** ($CDCl_3$, 101 MHz) (*trans*): δ 177.3, 74.8, 36.3, 30.5, 18.1; GC-MS: m/z (%) 100(M^+,40), 70(30), 56(100). The spectroscopic data were consistent with those reported in literature.[10]

Synthesis of Eldanolide (1i)

The preparation of eldanolide **1i** was carried out following a literature procedure,[11] slightly modified (Scheme S2). The first step consisted of a Johnson-Claisen rearrangement starting from the commercially available but-2-en-1-ol, triethyl orthoacetate, and a catalytic amount of propionic acid, yielding ester **10** in a good yield. Compound **10** was treated with *m*-chloroperbenzoic acid (MCPBA) affording epoxide **11**. Then, the commercially available 2-methyl-1-propenylmagnesium bromide reacted with epoxide **11** in the presence of a sub-stoichiometric amount of CuBr affording the hydroxyester intermediate of sufficient purity for the next step.[12] In the final step, the hydroxyester was cyclized to yield the desired product **1i**, by treatment with a catalytic amount of TFA. The product was purified by silica gel column chromatography.

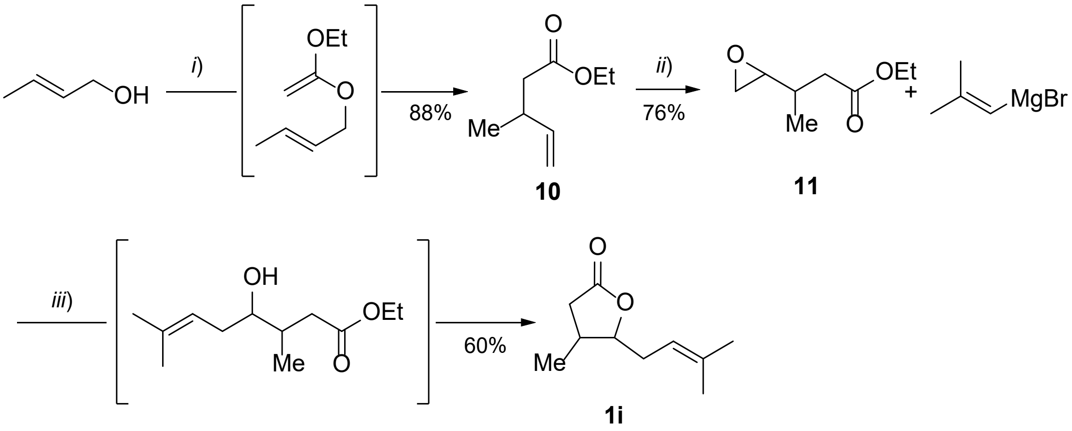

Scheme S2. Reaction conditions: *i*) $CH_3C(OEt)_3$, $CH_3CH_2CO_2H$ cat., 120 °C. *ii*) MCPBA, $NaHCO_3$, CH_2Cl_2, 5-10 °C. *iii*): a) THF, 0.1 eq. CuBr, -78 °C to -50 °C; b) TFA cat., CH_2Cl_2, 0 °C to rt.

Ethyl 3-methylpent-4-enoate (10)

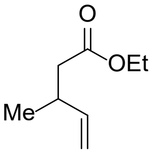

10

A solution of but-2-en-1-ol (11.0 g, 152.7 mmol), triethyl orthoacetate (60 mL) and propionic acid (0.5 g) was left to stir at 120 °C for 1h under a N_2 atmosphere. Then the triethyl orthoacetate left was removed under *vacuum* (60 °C, 30 mbar). The crude material was of sufficient purity for the next step. Yield 87% (19.0 g) as a colorless liquid; 89% purity by GC-MS; t_R=5.4 min, **^{1}H-NMR** ($CDCl_3$, 400 MHz): δ 5.76 (m, 1H), 5.08-4.88 (m, 2H), 4.22-4.04 (m, 2H), 2.63 (m, 1H), 2.38 (m, 1H), 2.24 (m, 1H), 1.24 (m, 3H), 1.05 (m, 3H); **^{13}C{^{1}H}-NMR** ($CDCl_3$, 101 MHz): δ 172.6, 142.7, 113.4, 60.3, 41.5, 34.6, 19.8, 14.4; GC-MS: m/z (%) 142(M^+,13), 96(67), 69(100), 55(53). The spectroscopic data were consistent with those reported in literature.[13]

Ethyl 3-(oxiran-2-yl)butanoate (11)

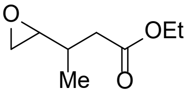

11

To an ice-cooled and mechanically stirred suspension of MCPBA (77% w/w, 22.0 g, 127.5 mmol) and $NaHCO_3$ (11.0 g, 131.0 mmol) in CH_2Cl_2 (250 mL) was added dropwise a solution of **10** (14.2 g, 100 mmol) in CH_2Cl_2 (20 mL) at a rate such that the temperature was kept between 5-10 °C (usually over 30 minutes), after addition the reaction mixture was left to reach room temperature. After 2 hours the reaction mixture was quenched with a solution of $NaHSO_3$ (1.0 M, 50 mL) and left to stir (30 minutes) at room temperature and then filtered. The white solid was washed with CH_2Cl_2 (3 x 25 mL). The organic phase was first extracted with H_2O (50 mL) and then with a solution of $NaHCO_3$ (sat., 50 mL). The combined aqueous phase was extracted with CH_2Cl_2 (2 x 25 mL). Then, the combined organic phase was dried over anhydrous Na_2SO_4 and concentrated under *vacuum* affording **11** as a pale-yellow liquid which was purified by Vigreux (30 cm length) distillation (90 °C, 2-3 mbar).

Yield 76% (11.5 g) as a colorless liquid; 92% purity, t_R=9.9 min and 10.0 min, (diastereomeric ratio=51:49) by GC-MS; **^{1}H-NMR** ($CDCl_3$, 400 MHz): δ 4.16-4.10 (m, 2H), 2.81 (m, 1H), 2.74 (m, 1H), 2.53 (m, 1H), 2.41- 2.19 (m, 2H), 1.93 (m, 1H), 1.25 (t, J=7.1 Hz, 3H), 1.04 (m, J=16.9, 6.9 Hz, 3H); **^{13}C{^{1}H}-NMR** ($CDCl_3$, 101 MHz) δ 172.4, 172.3, 60.6, 60.5, 55.91, 55.87, 46.5, 46.4, 38.7, 38.2, 33.5, 32.9, 16.8, 15.9, 14.4; GC-MS (first diastereoisomer): m/z (%) 158(M^+,2), 85(96), 69(100), 55(75); GC-MS (second diastereoisomer): m/z (%) 158(M^+,1), 85(88), 69(100), 55(80).

4-Methyl-5-(3-methylbut-2-en-1-yl)dihydrofuran-2(3*H*)-one (1i)

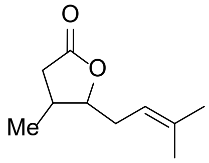

1i

To a well stirred mixture of **11** (2.4 g, 15.0 mmol) and CuBr (0.2 g, 1.5 mmol) in dry THF (40 mL) at -78 °C under N_2 atmosphere, was added dropwise a solution of 2-methyl-1-propenylmagnesium bromide in THF (60 mL, 0.5 M, over 30 minutes). The reaction mixture was left to stir for 1 hour. Then it was left to reach -50 °C and after 4 hours was quenched with a solution of NH_4Cl (sat., 50 mL). The mixture was left to reach room temperature, and after 1 hour, it was extracted with EtOAc (3 x 50 mL). The combined organic phase was extracted with brine (sat., 50 mL), dried over anhydrous Na_2SO_4 and concentrated under *vacuum.* The crude material was ice-cooled, diluted in CH_2Cl_2 (30 mL) and TFA (100 µL) was added, then the reaction was left to stir at room temperature. After 12 hours, the reaction mixture was ice-cooled and quenched with a solution of $NaHCO_3$ (sat., 30 mL) and left to stir at room temperature. After 30 minutes, it was extracted with CH_2Cl_2 (3 x 30 mL). The combined organic phase was extracted with brine (sat., 30 mL), dried over anhydrous Na_2SO_4 and concentrated under *vacuum* affording **1i** as pale-yellow oil, which was purified through silica gel column chromatography using *n*-hexane/EtOAc (80:20) as eluent. Then, the product was further purified by bulb-to-bulb distillation (110 °C, 0.1mbar).

Yield 60% (1.5 g) as a slightly yellow oil; 86% purity by GC-MS; t_R=15.5 min (*trans*) and 16.5 min (*cis*); *trans*/*cis*=41:59 by GC-MS and ^{1}H-NMR; t_R=12.0 min (4*S*,5*R*), t_R=12.3 min (4*R*,5*S*), t_R=13.4 min (4*S*,5*S* and 4*R*,5*R*) by chiral GC; **^{1}H-NMR** ($CDCl_3$, 400 MHz): δ 5.13 (m, 1H), 3.92 (m, 0.59H), 4.13 (m, 0.41H), 2.68 (m, 1H), 2.52 (m, 1H), 2.41-2.24 (m, 2H), 2.16 (m, 1H), 1.70 (s, 3H), 1.62 (s, 3H), 1.12 (d, J=6.6 Hz, 1.23H), 1.02 (d, J=7.0 Hz, 1.77H); **^{13}C{^{1}H}-NMR** ($CDCl_3$, 101 MHz) (*trans*): δ 176.6, 135.5, 118.1, 87.2, 37.2, 35.2, 32.3, 25.9, 18.1, 14.0; (*cis*): δ 176.8, 135.2, 118.2, 83.5, 37.7, 33.0, 28.9, 25.9, 17.9, 14.0; GC-MS (*trans*): m/z (%) 168(M^+,38), 99(100), 71(52), 69(16); GC-MS (*cis*): m/z (%) 168(M^+,35), 99(100), 71(45), 69(15). The spectroscopic data were consistent with those reported in literature.[14]

Synthesis of 5-butyl-3-methyldihydrofuran-2(3*H*)-one (1m)

The synthesis of γ-lactone **1l** involves a one-pot two-step reaction sequence: by treatment of the commercially available γ-octanolactone with lithium diisopropylamide (LDA) is formed the enolate, the latter reacts with the electrophilic methyl iodide affording the α-methylated lactone (Scheme S3).

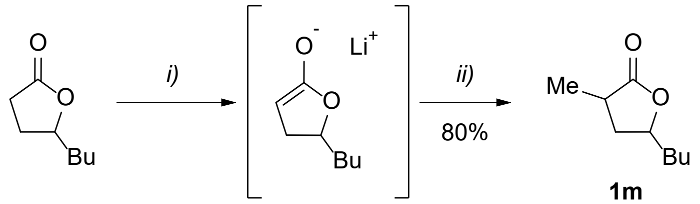

1m

Scheme S3. Reaction conditions: *i*): a) DIPA, BuLi, THF, -20 °C; b) γ-octalactone, THF, -78 °C. *ii*) MeI, THF/DMPU (1:1), -78 °C to -50 °C.

To a well-stirred solution of DIPA (1.7 mL) in dry THF (40 mL) under an N_2 atmosphere at -20 °C a solution of BuLi (1.6 M, 7.5 mL, 12 mmol) in *n*-hexane was dropwise added. After 2 hours, once LDA had formed, a solution of γ-octanolactone (1.42 g, 10 mmol) in dry THF (10 mL) was added at -78 °C, usually over 10 minutes, and left to stir. After 30 minutes, a solution of MeI (1.7 g, 12 mmol) in THF/DMPU (10 mL, 1:1) was dropwise added at -78 °C, usually over 10 minutes, and left to stir at -50 °C. After 1 hour, it was quenched with a solution of NH_4Cl (sat., 50 mL) and left to stir at room temperature. After 30 minutes, Et_2O (50 mL) was added and the organic phase was extracted with $NaHCO_3$ (sat., 50 mL), brine (sat., 50 mL), dried over anhydrous Na_2SO_4 and concentrated under *vacuum* affording **1m** as pale-yellow oil, which was purified through silica gel column chromatography with *n*-hexane/EtOAc (80:20).

Yield 80% (1.20 g) as a pale-yellow oil; 98% purity by GC-MS; t_R=17.5 min (*cis*) and 17.6 min (*trans*), *cis/trans*=15:85 by GC-MS and ^{1}H-NMR; **^{1}H-NMR** ($CDCl_3$, 400 MHz): δ 4.49 (m, 0.85H), 4.32 (ddt, J=10.7, 7.4, 5.5 Hz, 0.15H), 2.67 (m, 0.85H), 2.48 (m, 0.15H), 2.11 (m, 1H), 1.98 (m, 1H), 1.71 (m, 1H), 1.53 (m, 1H), 1.42-1.31 (m, 2H+2H), 1.27 (m, 3H), 0.91 (t, J=7.0 Hz, 3H); **^{13}C{^{1}H}-NMR** ($CDCl_3$, 101 MHz) (*trans*): δ 180.2, 78.6, 35.6, 35.3, 34.2, 27.6, 22.5, 16.0, 14.0; GC-MS (*cis*): m/z (%) 156(M^+,1), 99(100), 71(37), 55(35); GC-MS (*trans*): m/z (%) 156(M^+,2), 99(100), 71(42), 55(28). The spectroscopic data were consistent with those reported in literature.[15]

Synthesis of (*R*)-5-methyl-5-(4-methylpentyl)dihydrofuran-2(3H)-one (1o)

In Scheme S4 is shown the synthesis of γ-lactone **1o**: the vinyl lithium of (*R*)-linalool reacts with CO_2 affording the carboxylate intermediate, that during the acidic work-up undergoes to spontaneous cyclization forming lactone **12**. In the next step both C=C double bonds of **12** are reduced by catalytic hydrogenation in presence of Pd/C affording the substrate **1o**.

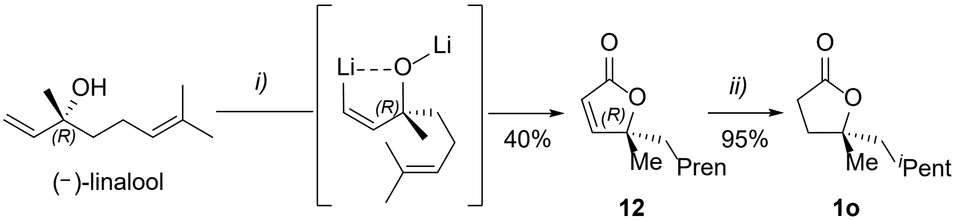

Scheme S4. Reaction conditions: *i*): a) TMEDA, BuLi, hexane, -20 °C (2 h) then rt (1 h); b) CO_2 at -78 °C; c) HCl. *ii*) H_2, Pd/C (substrate/catalyst 10% w/w), EtOAc, rt.

(*R*)-5-Methyl-5-(4-methylpent-3-en-1-yl)furan-2(5H)-one (12)

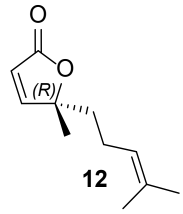

12

A solution of BuLi (2.5 M, 110 mL, 275 mmol) in *n*-hexane was added dropwise, at -20 °C, to a well-stirred solution of TMEDA (39 mL) and (*R*)-linalool (20 g, 130 mmol) in dry hexane (100 mL) under a N_2 atmosphere. After 2 hours, the cooling bath was removed, the reaction was left to reach rt and stirring was prolonged for further 10 hours. Hence, the reaction mixture was added portionwise to a mechanically stirred slurry of dry ice and hexane. After 30 minutes, the reaction was quenched with aqueous solution of HCl (1 M, 350 mL), the organic phase was separated, and the aqueous phase was extracted with Et_2O (2 x 60 mL). The combined organic phase was washed with brine (sat., 100 mL), dried over anhydrous Na_2SO_4 and concentrated under *vacuum*. The residue was purified through silica gel column chromatography (*n*-hexane/EtOAc, 8:2) following by bulb-to-bulb distillation, to afford lactone **12**.[16]

Yield 40% (9.40 g) as a pale-yellow oil; 95% purity by GC-MS; t_R=16.5 min; $[\alpha]_D$= +101.4 ($CHCl_3$, *c*=2.3) *vs* lit. -95.6 ($CHCl_3$, *c*=1.0);[17] **^{1}H-NMR** $CDCl_3$, 400 MHz): δ 7.34 (d, J=5.6 Hz, 1H), 5.98 (d, J=5.6 Hz, 1H), 5.00 (m, 1H), 1.65-2.05 (m, 4H), 1.65 (s, 3H), 1.55 (s, 3H), 1.45 (s, 3H); **^{13}C{^{1}H}-NMR** ($CDCl_3$, 101 MHz): δ 172.7, 160.4, 132.8, 123.0, 120.5, 89.0, 38.4, 25.7, 24.2, 22.6, 17.80; GC-MS: m/z (%) 180(M^+,16), 165(15), 135(2), 103(68), 98(100). The spectroscopic data were consistent with those reported in literature.[17]

(*R*)-5-Methyl-5-(4-methylpentyl)dihydrofuran-2(3H)-one (1o)

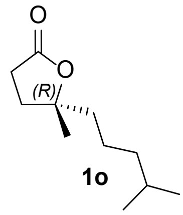

1o

To a solution of **12** (0.7 g, 3.9 mmol) in EtOAc (20 mL) was added a sub-stoichiometric amount of Pd/C substrate/catalyst 10% w/w). Then, the reaction mixture was well stirred and kept under an H_2 atmosphere until the complete conversion of starting material (checked by GC-MS). Subsequently, the mixture was filtered on a celite pad, which was washed with EtOAc (3 x 10 mL). The organic phase was dried over anhydrous Na_2SO_4, and the filtrate solution was concentrated under *vacuum*, the crude material was submitted to bulb-to-bulb distillation affording lactone **1o**.

Yield 95% (0.67 g) as a pale-yellow liquid; 98% purity by GC-MS; t_R=16.8 min; $[\alpha]_D$= +7.6 ($CHCl_3$, *c*=1.8) *vs* lit. +6.98 (isooctane);[18] **^{1}H-NMR** ($CDCl_3$, 400 MHz): δ 2.67-2.51 (m, 1H+1H), 2.08 (ddd, J=12.8, 9.3, 8.1 Hz, 1H), 1.97 (ddd, J=12.8, 9.3, 6.6 Hz, 1H), 1.49-1.60 (m, 2H+1H), 1.31-1.42 (m, 2H+3H), 1.13-1.22 (m, 2H), 0.86 (d, J=12.8 Hz, 6H); **^{13}C{^{1}H}-NMR** ($CDCl_3$, 101 MHz): δ 177.0, 87.1, 41.4, 39.2, 33.1, 29.3, 28.0, 25.8, 2.7, 22.6, 21.8; GC-MS: m/z (%)

185(M+1,1), 151(3), 114(3), 99(100), 69(10). The ^{1}H-NMR spectroscopic data were consistent with those reported in literature.[18]

Synthesis of substrate precursor of forskolin (1q)

The synthesis of substrate **1q**, precursor of the natural product Forskolin, and allylic alcohol **5q** are shown in Scheme S5. First, bromide derivative **15** was prepared through a three-step sequence: (*i*) Johnson-Claisen rearrangement affording ester **13**; (*ii*) reduction of ester **13** with $LiAlH_4$; and (*iii*) conversion of alcohol **14** into bromide **15** using NBS and PPh_3. Then, the bromide **15** was then employed for the preparation of the corresponding Grignard reagent, which, according to our synthetic plan, was added to the formyl crotonate aldehyde, following the same approach described in Scheme S1. However, under the reaction conditions reported by Studer *et al.*,[19] the formation of the Grignard reagent proved to be quite troublesome. Indeed, after addition of the aldehyde, the major isolated product was the hydrocarbon **16**, most likely arising from a Wurtz-type homocoupling, rather than the desired allylic alcohol **5q**.
As an alternative, the iodo derivative **17** was treated with *tert*-butyllithium to generate the corresponding organolithium reagent, which upon addition to the formyl crotonate aldehyde afforded the allylic alcohol **5q**. Whereas, the substrate **1q** was obtained by transmetalation of the organolithium specie with $MgBr_2$, followed by addition of the resulting Grignard reagent to the saturated aldehyde **9**. The resulting γ-hydroxy ester underwent spontaneous cyclization during the acidic work-up to furnish lactone **1q**.

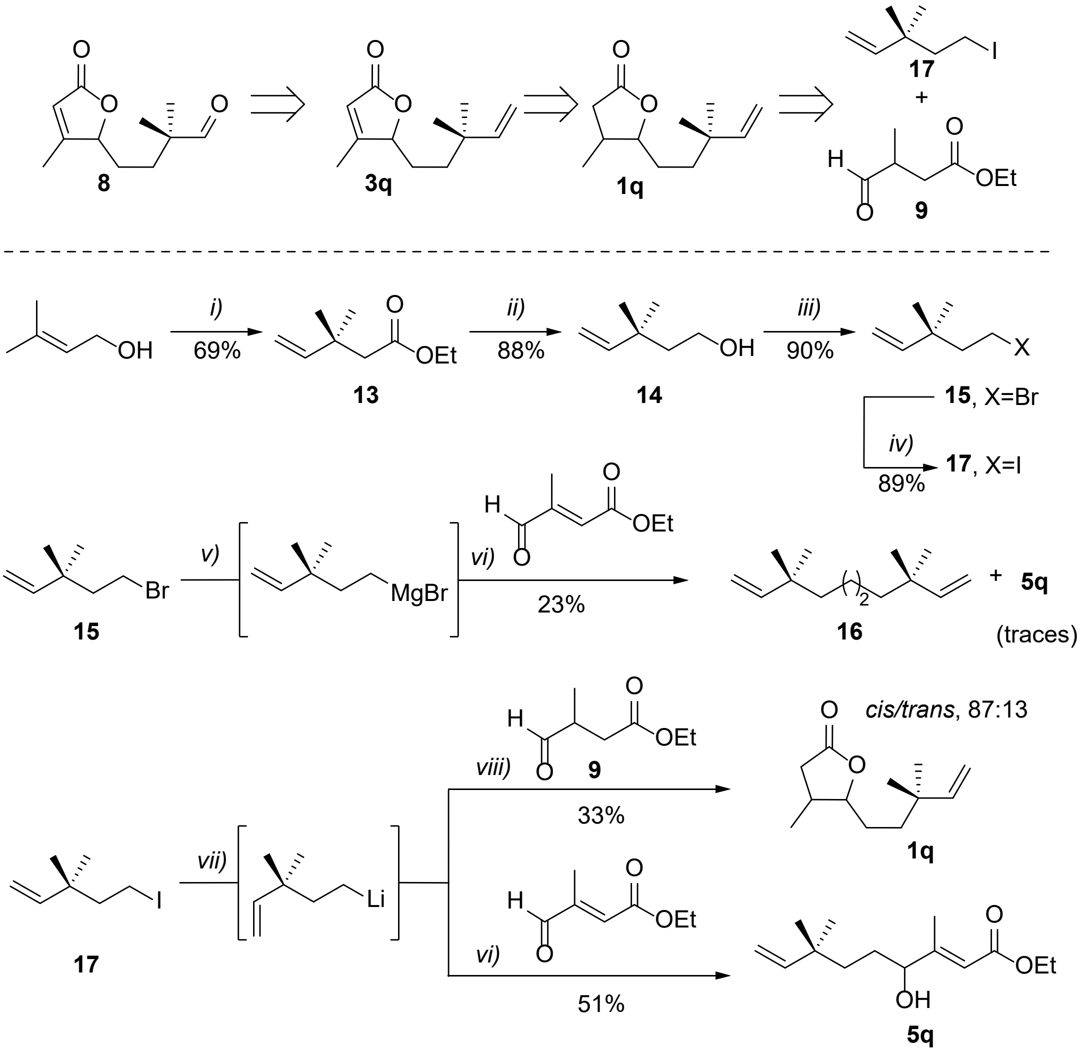

Scheme S5. Reaction conditions: *i*) $CH_3C(OEt)_3$, H_3PO_4 cat., 120 °C. *ii*) $LiAlH_4$, Et_2O, 0 °C to rt. *iii*) NBS, PPh_3, CH_2Cl_2, 0 °C to rt. *iv*) NaI, acetone, reflux. *v*) Mg, THF, reflux. *vi*) THF, -78 °C to rt. *vii*) *t*-BuLi, pentane/Et_2O, -78 °C to rt. *viii*): a) $MgBr_2$, Et_2O, 0 °C to rt; b) THF, -78 °C to -30 °C.

Ethyl 3,3-dimethylpent-4-enoate (13)

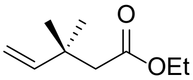

13

To a reaction vessel equipped with a Claisen apparatus and charged with a solution of prenyl alcohol (43.0 g, 0.50 mol) in triethyl orthoacetate (240.0 g) was added H_3PO_4 (0.4 g). The reaction mixture was heated at 120 °C, and the ethanol formed was distilled off. After the distillation was complete, the mixture was maintained at the same temperature for 12 hours, then concentrated to one third of the initial volume and cooled to room temperature. The residue was diluted with Et_2O (150 mL), washed with sat. brine (1 x 50 mL) and sat. Na_2CO_3 (1 x 50 mL), dried over anhydrous Na_2SO_4, and concentrated under reduced pressure. The crude product was purified by distillation under reduced pressure (48 mbar, 77-80 °C) to afford ester **13**.

Yield: 69% (53.9 g), as a colorless liquid; t_R=6.38 min, 92% purity by GC-MS; ^{1}**H-NMR** ($CDCl_3$, 400 MHz): δ 5.90 (dd, J=17.7, 10.4 Hz, 1H), 4.96 (m, 2H), 4.13 (q, J=7.1 Hz, 2H), 2.29 (s, 2H), 1.22 (t, J=7.1 Hz, 3H), 1.13 (s, 6H); ^{13}**C{^{1}H}-NMR** ($CDCl_3$, 101 MHz): δ 171.7, 146.9, 110.7, 60.0, 46.8, 36.1, 26.9, 14.3; GC-MS: m/z 156 (M^+,2), 141(8), 127(5), 110(58), 82(70), 68(100). The spectroscopic data are consistent with those reported in literature.[20]

3,3-Dimethylpent-4-en-1-ol (14)

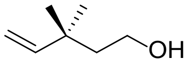

14

To an ice-cooled, well-stirred solution of **13** (27.5 g, 176 mmol) in dry Et_2O (350 mL) was added portionwise $LiAlH_4$ (6.0 g, 158 mmol) over 1 h. The reaction mixture was then stirred at room temperature for 3 h, until complete consumption of the starting material (monitored by TLC and/or GC-MS). The mixture was cooled to 0 °C, carefully quenched with EtOAc (5 mL), and subsequently treated with an aqueous solution of Rochelle's salt (sat., 400 mL). Stirring continued for 12 hours, and the phases were separated. The organic phase was dried over anhydrous Na_2SO_4 and concentrated under reduced pressure. The crude product was purified by distillation under reduced pressure (32 mbar, 80-81 °C) to give **14**.

Yield: 88% (17.9 g), as a colorless liquid; t_R=4.4, 96% purity by GC-MS; ^{1}**H-NMR** ($CDCl_3$, 400 MHz): δ 5.85 (dd, J=17.5, 10.8 Hz, 1H), 4.96 (m, 1H+1H), 3.65 (t, J=7.1 Hz, 2H), 1.62 (t, J=7.1 Hz, 2H), 1.03 (s, 6H); ^{13}**C{^{1}H}-NMR** ($CDCl_3$, 101 MHz): δ 148.4, 110.9, 60.3, 45.3, 35.9, 27.3; GC-MS: m/z 96 (M-18, 5), 81(100), 69(80). The spectroscopic data are consistent with those reported in literature.[19]

5-Bromo-3,3-dimethylpent-1-ene (15)

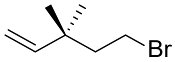

15

To a stirred solution of **14** (17.6 g, 153 mmol) and PPh_3 (48.3 g, 184 mmol) in CH_2Cl_2 (400 mL) at 0 °C was added portionwise NBS (32.7 g, 184 mmol). After complete consumption of the starting alcohol (typically after 2 hours, monitored by GC-MS), MeOH (5 mL) was added, and the reaction mixture was concentrated to one quarter of its initial volume. Pentane (200 mL) was then added, and the ice cooled mixture was filtered through a celite pad. The filtrate was concentrated under reduced pressure and purified by distillation (30 mbar, 68-70 °C) to give **15**.

Yield: 90% (24.4 g), as a colorless liquid; t_R=5.5 min, 98% purity by GC-MS; ^{1}**H-NMR** ($CDCl_3$, 400 MHz): δ 5.74 (dd, J=17.4, 10.8 Hz, 1H), 4.92-5.01 (m, 1H+1H)), 3.27-3.33 (m, 1H+1H), 1.90-1.95 (m, 1H+1H), 1.03 (s, 6H); ^{13}**C{^{1}H}-NMR** ($CDCl_3$, 101 MHz): δ 146.7, 111.8, 46.1, 40.0, 29.5, 26.7; GC-MS: m/z 178 (M^+, 1), 176 (M^+, 1), 97(20), 81(15), 68(100). The spectroscopic data are consistent with those reported in literature.[21]

3,3,8,8-Tetramethyldeca-1,9-diene (16)

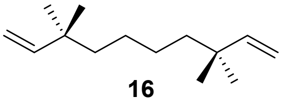

16

To a suspension of Mg powder (280 mg, 11.7 mmol) in dry THF (5 mL) was added 1,2-dibromoethane (0.2 mL). After clear evolution of ethylene was observed, a solution of bromide **15** (490 mg, 2.8 mmol) in THF (3 mL) was added. The reaction mixture was stirred for two hours, with occasional heating. The mixture was then filtered under N_2 through a pad of glass wool, and the filtrate was added dropwise to a solution of aldehyde **9** (253 mg, 1.8 mmol) in THF (10 mL) at -78

°C under N_2 atmosphere. The reaction was allowed to warm to -50 °C and stirred for an additional two hours, then quenched with NH_4Cl (sat., 10 mL) under vigorous stirring. After one hour, the reaction mixture was extracted with Et_2O (3 x 15 mL). The combined organic layers were washed with brine (sat., 10 mL), dried over anhydrous Na_2SO_4, and concentrated under reduced pressure. The crude yellow oil was purified by silica gel column chromatography (*n*-pentane/Et_2O, 90:10) to afford **16**.

Yield: 23% (125 mg), as a colorless liquid; t_R=11.7 min, 99% purity by GC-MS; **^{1}H-NMR** ($CDCl_3$, 400 MHz): δ 5.75 (m, 2H), 4.84-2.93 (m, 2H+2H)), 1.12-1.29 (m, 4H), 0.96 (s, 12H); **^{13}C{^{1}H}-NMR** ($CDCl_3$, 101 MHz): δ 148.9, 110.1, 43.0, 36.7, 26.9, 25.5; GC-MS: m/z 194 (M^+,1), 151(2), 123(10), 109(10), 95(20), 69(100).

5-Iodo-3,3-dimethylpent-1-ene (17)

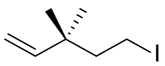

17

To a stirred solution of **15** (17.7 g, 100 mmol) in acetone (300 mL) was added NaI (54 g, 360 mmol). The reaction mixture was refluxed for 3 hours and then concentrated to one quarter of its initial volume. Pentane (200 mL) was added, and the ice-cooled mixture was filtered through a celite pad, the cake was washed with pentane (3 x 25 mL). The filtrate solution was washed with water (50 mL), dried over Na_2SO_4, and the organic phase was concentrated. The crude material was purified by distillation under reduced pressure (30 mbar, 88 °C) to give **17**.

Yield: 89% (19.9 g), as a slightly pink liquid; t_R=7.4 min, 98% purity by GC-MS; **^{1}H-NMR** ($CDCl_3$, 400 MHz): δ 5.66 (dd, J=17.4, 10.8 Hz, 1H), 4.93 (d, J=10.8, 1.2 Hz, 1H), 4.88 (d, J=17.4, 1.2 Hz, 1H), 2.98-3.04 (m, 1H+1H), 1.91 (m, 1H+1H), 0.95 (s, 6H); **^{13}C{^{1}H}-NMR** ($CDCl_3$, 101 MHz): δ 146.5, 111.9, 47.8, 39.4, 16.4, 1.1; GC-MS: m/z 224 (M^+, 23), 155(5), 127(3), 97(24), 69(100). The spectroscopic data are consistent with those reported in literature.[22]

Ethyl (*E*)-4-hydroxy-3,7,7-trimethylnona-2,8-dienoate (5q)

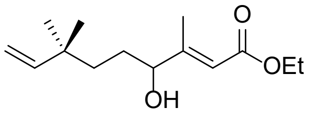

5q

The alkyl iodide **17** (5 g, 22.3 mmol) was dissolved in mixture of dry *n*-pentane/Et_2O (75 mL, 3:2). The solution was cooled at -78 °C, and a solution of *t*-BuLi in *n*-pentane (1.9 M, 25.6 mL) was added dropwise under a N_2 atmosphere. Stirring continued at -78 °C for an additional 5 min following the addition. Hence, the cooling bath was removed, and the mixture was allowed to warm and stand at room temperature for 1 hour, in such a way to consume the unreacted *t*-BuLi. The mixture was then cooled to -78°C, and an excess of ethyl *trans*-3-methyl-4-oxocrotonate (5 g, 35.2 mmol) in dry Et_2O (15 mL) was then added dropwise under vigorous stirring. The reaction mixture was left to reach -30 °C, and after 2 hours it was quenched with a solution of NH_4Cl (sat., 150 mL). The organic phase was separated, and the aqueous solution was extracted with Et_2O (2 x 60 mL). The combined organic phase was washed with brine (sat., 100 mL), dried over anhydrous Na_2SO_4 and concentrated under *vacuum*. The residue was purified through silica gel column chromatography (*n*-hexane/EtOAc, 7:3), following by bulb-to bulb distillation to afford **5q**.

Yield: 51% (2.73 g), as a slightly yellow liquid; t_R=21.0 min, 97% purity by GC-MS; **^{1}H-NMR** ($CDCl_3$, 400 MHz): δ 5.87 (m, 1H), 5.71 (dd, J=17.4, 10.8 Hz, 1H), 4.86-4.92 (m, 2H), 4.14 (t, J=7.1 Hz, 2H), 4.01 (m, 1H), 2.07 (s, 3H), 1.35-1.60 (m, 3H), 1.17-1.34 (m, 3H+2H), 0.97 (s, 6H); **^{13}C{^{1}H}-NMR** ($CDCl_3$, 101 MHz): δ 167.0, 160.2, 148.0, 115.4, 111.0, 77.2, 59.9, 38.1, 36.4, 30.2, 26.9, 26.7, 14.8, 14.4; GC-MS: m/z 240 (M^+,1), 197(3), 194(2), 179(2), 143(80), 115(100). ESI-HRMS (m/z): calcd. for $C_{14}H_{24}O_3Na^+$ $[M+Na]^+$ 263.1618, found 263.1615.

5-(3,3-Dimethylpent-4-en-1-yl)-4-methyldihydrofuran-2(3*H*)-one (1q)

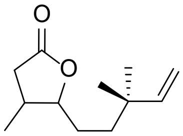

1q

The alkyl iodide **17** (5.0 g, 22.3 mmol) was dissolved in a mixture of dry *n*-pentane/Et_2O (75 mL, 3:2) and cooled at -78 °C, then a solution of *t*-BuLi in *n*-pentane (1.9 M, 25.6 mL) was added dropwise under a N_2 atmosphere. Stirring continued at -78 °C for an additional 5 min following the addition. Hence, the cooling bath was removed, and the mixture was allowed to warm and stand at room temperature for 1 hour, in such a way to consume the unreacted *t*-BuLi. The mixture was then cooled at -78°C and was treated with a solution of $MgBr_2$ in dry THF (0.4 M, 56 mL). Then, a solution of ethyl

trans-3-methyl-4-oxocrotonate (5 g, 35.2 mmol) in dry Et_2O (15 mL) was added dropwise under vigorous stirring. The reaction mixture was left to reach -50 °C, and after 2 hours it was quenched with NH_4Cl aq. (sat., 150 mL). The organic phase was separated, and the aqueous solution was extracted with Et_2O (2 x 60 mL). The combined organic phase was washed with brine (sat., 100 mL), dried over anhydrous Na_2SO_4 and concentrated under *vacuum*. The residue was purified through silica gel column chromatography (*n*-hexane/EtOAc, 9:1), following by bulb-to bulb distillation to afford **1q**.

Yield: 33 % (1.44 g), 97% purity by GC-MS; t_R=18.0 min (*trans*) and 18.6 min (*cis*); *trans*/*cis*=87:13 by GC-MS and by ^{13}C-NMR; t_R=16.6 min (4*S*,5*R*), t_R=16.7 min (4*R*,5*S*), t_R=17.5 min (4*S*,5*S*), t_R=17.6 min (4*R*,5*R*) by chiral GC;**^{1}H-NMR** ($CDCl_3$, 400 MHz): δ 5.87 (m, 1H), 4.87 (m, 1H), 4.36 (m, 0.13H), 3.95 (td, J=7.6, 7.2, 3.5 Hz, 0.87H), 2.65 (m, 1H), 2.10-2.26 (m, 2H), 1.43-1.65 (m, 3H), 1.32 (m, 1H), 1.13 (d, 3H), 1.00 (m, 3H); **^{13}C{^{1}H}-NMR** ($CDCl_3$, 101 MHz): (*trans*) δ 176.6, 147.8, 111.16, 88.1, 38.3, 37.2, 36.3, 36.1, 29.4, 26.88, 26.7, 17.7; (*cis*) δ 176.9, 147.7, 111.21, 84.4, 38.6, 37.7, 36.5, 33.1, 26.92, 26.6, 25.3, 14.0; GC-MS (*trans*): m/z (%) 196(M^+,1), 181(2), 136(10), 109(20), 82(100); (*cis*): m/z (%) 196(M^+,1), 163(2), 136(8), 109(12), 82(100). ESI-HRMS (m/z): calcd. for $C_{12}H_{20}O_2Na^+$ $[M+Na]^+$ 219.1356, found 219.1360.

Synthesis of 3,4-dehydrogenated γ-lactones as reference for chiral GC analysis (3a-q)

The synthesis of dehydrogenated γ-lactones (**3**) was carried out using two different synthetic routes, respectively A and B (Scheme S6):

i) Route A involves the dehydrogenation of lactone **1** through α-selenylation with lithium diisopropylamide (LDA) and diphenyl diselenide (Ph_2Se_2). The selenyl intermediate, by treatment with H_2O_2, was oxidized to selenoxide derivative, the latter undergoes spontaneous elimination yielding the 3,4-dehydrogenated lactone **3**.[23]

ii) Route B involves an *E* to *Z* photoisomerization of hydroxyester **5**, obtained in the synthesis of lactones **1** as described at page S6. The *E*-**5** hydroxyester was isomerized through UV irradiation (λ=254 nm) in the presence of phenanthrene as photosensitizer, yielding *Z*-**5**, which spontaneously undergoes cyclization affording the desired 3,4-dehydrogenated lactone **3**.[24]

Although both yield and selectivity of route A were more than satisfactory, its employment was limited to lactone **3a** for safety reasons. The synthesis of the other dehydrogenated γ-lactones (**3b-q**) was carried out employing route B, due to its simplicity and safety.

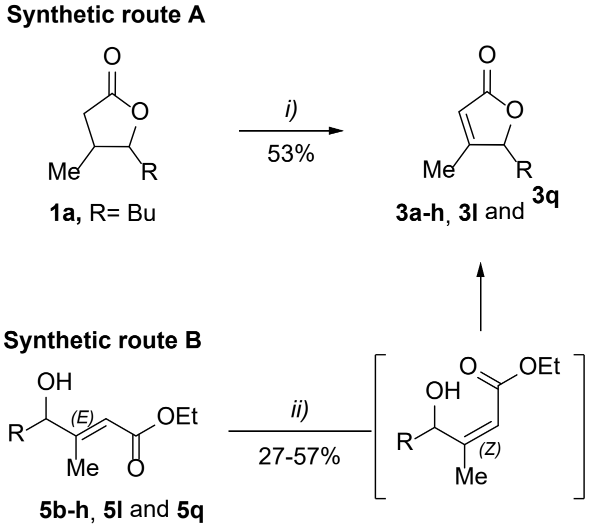

Lactone	R
**3a**	Butyl
**3b**	Propyl
**3c**	*i*-Propyl
**3d**	*i*-Butyl
**3e**	Pentyl
**3f**	*i*-Pentyl
**3g**	Hexyl
**3h**	Allyl
**3l**	Hydrogen
**3q**	3,3-Dimethylpent-4-en-1-yl

R= Bu, Pr, ^{i}Pr, ^{i}Bu, Pent, ^{i}Pent, Hex, All, H, 3,3-dimethylpent-4-en-1-yl

Scheme S6. Reaction conditions: *i*) a) DIPA, BuLi, THF, -20 °C; b) Ph_2Se_2, THF/DMPU (1:1), -78 °C to rt; c) H_2O_2, THF, 0 °C to rt; *ii*) UV irradiation (λ=254 nm), phenanthrene, CH_2Cl_2, rt.

General procedure for synthetic route A

To a well-stirred solution of DIPA (0.54 mL, 3.8 mmol) in dry THF (10 mL) at -20 °C under an N_2 atmosphere, a solution of BuLi (1.6 M, 2.4 mL, 3.8 mmol) was dropwise added. After 2 hours, once LDA had formed, a solution of lactone (3.2 mmol) in dry THF (10 mL) was added at -78 °C (usually over 10 minutes) and left to stir. After 30 minutes, a solution of Ph_2Se_2 (1.2 g, 3.8 mmol) in THF/DMPU (10 mL, 1:1) was dropwise added at -78 °C, (over 10 minutes) and let to stir at room temperature. After 2 hours it was ice-cooled and quenched with a solution of HCl (1 M, 20 mL) and left to stir at room temperature. After 30 minutes, Et_2O (20 mL) was added and the organic phase was extracted with $NaHCO_3$ (sat., 20 mL), brine (sat., 20 mL), dried over anhydrous Na_2SO_4 and concentrated under *vacuum*. Then, the crude material was ice-cooled, dissolved in THF (20 mL), a solution of H_2O_2 (35% w/v, 0.5 mL) was added dropwise, and left to stir at room temperature. After 2 hours, the mixture was ice-cooled and treated with a solution of $NaHCO_3$ (sat., 20 mL). After 30 minutes, Et_2O (20 mL) was added and the organic phase was extracted with brine (sat., 20 mL), dried over anhydrous Na_2SO_4 and concentrated under *vacuum*. The product was purified by silica gel column chromatography with *n*-hexane/EtOAc (80:20) as eluent and then by bulb-to-bulb distillation.

5-Butyl-4-methylfuran-2(5*H*)-one (3a)

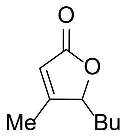

3a

Yield 53% (260 mg) as a colorless oil; 92% purity by GC-MS; t_R=12.3 min (5*R*) and t_R=12.9 min (5*S*) by chiral GC; b.p. 105 °C, 0.2-0.3 mbar; **^{1}H-NMR** ($CDCl_3$, 400 MHz): δ 5.79 (m, 1H), 4.82 (m, 1H), 2.04 (m, 3H), 1.89 (m, 1H), 1.51 (m,

1H), 1.42-1.34 (m, 2H+2H), 0.89 (t, J=6.8 Hz, 3H); **^{13}C{^{1}H}-NMR** (CDCl$_3$, 101 MHz): δ 173.5, 168.7, 117.1, 84.8, 31.8, 26.6, 22.5, 13.99, 13.95; GC-MS: m/z (%) 154 (M$^+$,16), 125(38), 97(100), 69(86). The spectroscopic data were consistent with those reported in literature.[25]

General procedure for synthetic route B

The air of a solution of hydroxyester **5b-h** (0.45 mmol) and phenanthrene (2 mg) in CH_2Cl_2 (60 mL) in a quartz tube was purged by bubbling Ar for 5 min. Then, the solution was irradiated in a photoreactor (λ=254 nm) for 24-48 hours. The solution was concentrated under *vacuum* to get the crude product, which was purified through silica gel column chromatography with *n*-hexane/EtOAc (80:20) affording lactone **3b-h**.

4-Methyl-5-propylfuran-2(5H)-one (3b)

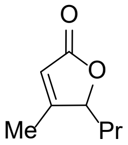

3b

Yield 57% (36 mg) as a pale-yellow oil; >99% purity by GC-MS, t_R=13.0 min; t_R=10.0 min (5*R*) and t_R=11.0 min (5*S*) by chiral GC; **^{1}H-NMR** (CDCl$_3$, 400 MHz): δ 5.76 (m, 1H), 4.80 (m, 1H), 2.05 (m, 3H), 1.85 (m, 1H), 1.53-1.37 (m, 1H+2H), 0.95 (t, J=7.2, 3H); **^{13}C{^{1}H}-NMR** (CDCl$_3$, 101 MHz): δ 173.4, 168.8, 117.0, 84.6, 34.2, 18.0, 14.0, 13.9; GC-MS: m/z (%) 140(M$^+$,44), 111(33), 97(100), 69(77). ESI-HRMS (m/z): calcd. for $C_8H_{12}O_2Na^+$ $[M+Na]^+$ 163.0730, found 163.0734.

5-Isopropyl-4-methylfuran-2(5*H*)-one (3c)

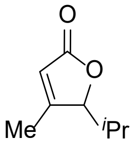

3c

Yield 54% (34 mg) as a pale-yellow oil; t_R=12.0 min, 98% purity by GC-MS; t_R=9.2 min (5*R*) and t_R=9.4 min (5*S*) by chiral GC; **^{1}H-NMR** (CDCl$_3$, 400 MHz): δ 5.81 (m, 1H), 4.75 (m, 1H), 2.10 (m, 1H), 2.02 (m, 3H), 1.15 (d, J=6.9 Hz, 3H), 0.71 (d, J=6.9, 3H); **^{13}C{^{1}H}-NMR** (CDCl$_3$, 101 MHz): δ 173.6, 167.9, 117.7, 88.8, 29.7, 19.8, 14.1, 13.7; GC-MS: m/z (%) 140(M$^+$,12), 111(8), 98(100), 69(18). ESI-HRMS (m/z): calcd. for $C_8H_{12}O_2Na^+$ $[M+Na]^+$ 163.0730, found 163.0728.

5-Isobutyl-4-methylfuran-2(5H)-one (3d)

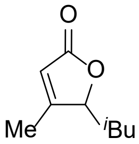

3d

Yield 43% (30 mg) as a pale-yellow oil; t_R=17.4 min, 98% purity by GC-MS; t_R=10.6 min (5*R*) and t_R=11.0 min (5*S*) by chiral GC; **^{1}H-NMR** (CDCl$_3$, 400 MHz): δ 5.74 (m, 1H), 4.80 (m, 1H), 2.04 (m, 3H), 1.95 (m, 1H), 1.57 (m, 1H), 1.38 (m, 1H), 0.99 (d, J=6.6 Hz, 3H), 0.96 (d, J=6.7 Hz, 3H); **^{13}C{^{1}H}-NMR** (CDCl$_3$, 101 MHz): δ 173.4, 169.5, 116.6, 83.5, 41.7, 25.3, 23.6, 21.7, 14.0; GC-MS: m/z (%) 154(M$^+$,22), 143(61), 115(100), 87(50). ESI-HRMS (m/z): calcd. for $C_9H_{14}O_2Na^+$ $[M+Na]^+$ 177.0886, found 177.0883.

4-Methyl-5-pentylfuran-2(5H)-one (3e)

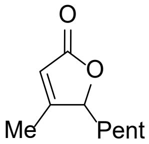

3e

Yield 27% (20 mg) as a pale-yellow oil; t_R=17.6 min, 96% purity by GC-MS; t_R=14.4 min (5*R*) and t_R=15.0 min (5*S*) by chiral GC; **^{1}H-NMR** (CDCl$_3$, 400 MHz): δ 5.77 (m, 1H), 4.81 (m, 1H), 2.04 (m, 3H), 1.89 (m, 1H), 1.49 (m, 1H), 1.44-1.37 (m, 2H), 1.32-1.28 (m, 4H), 0.88 (m, 3H); **^{13}C{^{1}H}-NMR** (CDCl$_3$, 101 MHz): δ 173.3, 168.6, 116.9, 84.6, 31.9, 31.5,

24.0, 22.4, 13.9, 13.8; GC-MS: m/z (%) 168(M^+,8), 139(45), 98(100), 69(93). The spectroscopic data were consistent with those reported in literature.[14]

5-Isopentyl-4-methylfuran-2(5*H*)-one (3f)

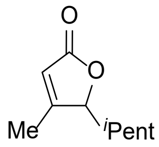

3f

Yield 28% (21 mg) as a pale-yellow oil; t_R=16.6 min, 98% purity by GC-MS; t_R=13.3 min (5*R*) and t_R=13.7 min (5*S*) by chiral GC; **^{1}H-NMR** ($CDCl_3$, 400 MHz): δ 4.82 (m, 1H), 2.04 (m, 3H), 1.92 (m, 1H), 1.64-1.45 (m, 1H+2H), 1.29-1.25 (m, 2H), 0.90 (d, J=4.5 Hz, 3H), 0.88 (d, J=4.5 Hz, 3H); **^{13}C{^{1}H}-NMR** ($CDCl_3$, 101 MHz): δ 173.4, 168.6, 117.2, 84.9, 33.2, 29.9, 28.0, 22.7, 22.4, 14.0; GC-MS: m/z (%) 168(M^+,17), 112(40), 98(100), 69(93). ESI-HRMS (m/z): calcd. for $C_{10}H_{16}O_2Na^+$ $[M+Na]^+$ 191.1043, found 191.1040.

5-Hexyl-4-methylfuran-2(5*H*)-one (3g)

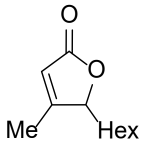

3g

Yield 54% (48 mg) as a pale-yellow oil; t_R=19.6 min, >99% purity by GC-MS; t_R=16.3 min (5*R*) and t_R=16.5 min (5*S*) by chiral GC; **^{1}H-NMR** ($CDCl_3$, 400 MHz): δ 5.78 (m, 1H), 4.81 (m, 1H), 2.03 (m, 3H), 1.91 (m, 1H), 1.50 (m, 1H), 1.43-1.35 (m, 2H), 1.33-1.20 (m, 6H), 0.87 (t, J=6.4 Hz, 3H); **^{13}C{^{1}H}-NMR** ($CDCl_3$, 101 MHz): δ 173.4, 168.7, 117.0, 84.7, 32.1, 31.7, 29.1, 24.4, 22.6, 14.1, 14.0; GC-MS: m/z (%) 182(M^+,13), 113(40), 98(100), 69(73). The spectroscopic data were consistent with those reported in literature.[26]

5-Allyl-4-methylfuran-2(5*H*)-one (3h)

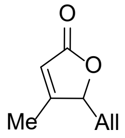

3h

Yield 41% (26 mg) as a pale-yellow oil; t_R=12.6 min, 98% purity by GC-MS; t_R=10.4 min (5*R*) and t_R=11.2 min (5*S*) by chiral GC; **^{1}H-NMR** ($CDCl_3$, 400 MHz): δ 5.82 (m, 1H), 5.71 (m, 1H), 5.24-5.10 (m, 2H), 4.91 (m, 1H), 2.70 (m, 1H), 2.36 (m, 1H), 2.05 (m, 3H); **^{13}C{^{1}H}-NMR** ($CDCl_3$, 101 MHz): δ 173.0, 167.9, 130.8, 119.5, 117.7, 83.7, 36.0, 14.1; GC-MS: m/z (%) 138(M^+,11),97 (100), 69(18), 51(3). ESI-HRMS (m/z): calcd. for $C_8H_{10}O_2Na^+$ $[M+Na]^+$ 161.0573, found 161.0574.

4-Methylfuran-2(5*H*)-one (3l)

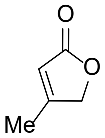

3l

Yield 49% (22 mg) as a colorless liquid; t_R=7.7 min, 98% purity by GC-MS; **^{1}H-NMR** ($CDCl_3$, 400 MHz): δ 5.85 (h, J=1.6 Hz, 1H), 4.71 (dd, J=1.8, 0.9 Hz, 2H), 2.13 (dt, J=1.8, 0.9 Hz, 3H); **^{13}C{^{1}H}-NMR** ($CDCl_3$, 101 MHz): δ 174.2, 166.1, 116.6, 74.0, 14.2; GC-MS: m/z (%) 98(M^+,40), 69 (100), 53(3). The spectroscopic data were consistent with those reported in literature.[27]

5-(3,3-Dimethylpent-4-en-1-yl)-4-methylfuran-2(5*H*)-one (3q)

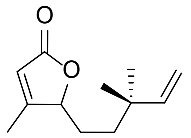

3q

Yield: 64% (56 mg), as a slightly yellow-brown liquid; t_R=19.2 min, 95% purity by GC-MS; t_R=18.4 min (5*R*) and t_R=18.5 min (5*S*) by chiral GC;**^{1}H-NMR** ($CDCl_3$, 400 MHz): δ 5.80 (p, J=1.6 Hz, 1H), 5.71 (dd J=17.4, 10.8 Hz, 1H), 4.88-4.97 (m, 2H), 4.83 (m, 1H), 2.03 (dd, J=1.6 and 0.7 Hz, 3H), 1.86 (m, 1H), 1.32-1.47 (m, 3H), 0.99 (m, 3H+3H); **^{13}C{^{1}H}-NMR** ($CDCl_3$, 101 MHz): δ 173.4, 168.5, 147.5, 117.3, 111.4, 84.9, 59.9, 38.1, 36.4, 36.2, 30.2, 27.2, 26.8, 14.0; GC-MS: m/z 194 (M^+,1), 170(10), 165(8), 151(20), 111(90), 98(100). ESI-HRMS (m/z): calcd. for $C_{12}H_{18}O_2Na^+$ $[M+Na]^+$ 217.1199, found 217.1122.

General procedure for biodesaturation in an Erlenmeyer flask (optimized conditions)

According to Table 1 (entry 4), 1 L conical Erlenmeyer flask was loaded with 100 mL of sterilized medium, which was inoculated with an active culture (5 mL) of *R. erythropolis* (DSM 44534). The flask was sealed with a cellulose plug, and it was shaken (130 rpm) at 26 °C. When the medium reached OD_{600} 1.3 (usually after 24 hours), the substrate (**1a-q**, 0.38 mmol) in EtOH (0.8 mL) was added at once. The reaction was monitored by GC-MS analysis, and after 3-7 days the mixture was filtered on a celite pad. The pad was washed several times with EtOAc (4 x 50 mL). The organic phase was separated from the aqueous phase, which was extracted again with EtOAc (4 x 50 mL). The combined organic phase was dried over Na_2SO_4, and the solvent was removed under reduced pressure. The crude material was submitted to silica gel column chromatography purification affording the dehydrogenated γ-lactone **3**. Yield and enantiomeric ratio are summarized in Table S1. Substrates **1m-p** were not desaturated.

Table S1. Biodesaturation of *Quercus*-like substrates (**1a-l**, **1q)** and substrates **5a**, **6** and **7**.

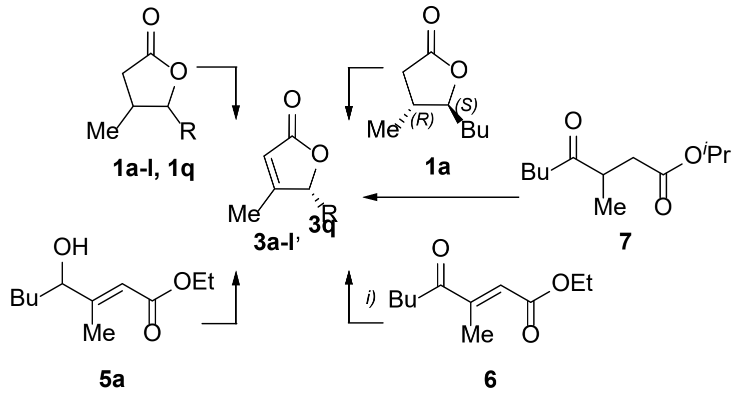

Substrate	R	Conv.[a] (%)	Yield (%)	(*R*)/(*S*)[b]	$[\alpha]_D$[c]	*dr* of unreacted 1 *trans/cis*[a]
**1a**	Bu	99	43	98:2	-7.4	>99
**1b**	Pr	75	32	96:4	-6.4	78:22
**1c**	^{i}Pr	-	-	-	-	-
**1d**	^{i}Bu	28	14	98:2	-4.0	80:20
**1e**	Pent	97	43d	97:3	-6.7	>99
**1f**	^{i}Pent	94	39	97:3	-6.5	88:12
**1g**	Hex	83	35	96:4	-3.0	87:13
**1h**	Allyl	-	traces	-	-	-
**1i**	Prenyl	15	10	99:1	-18.0	92:8
**1l**	H	-	-	-	-	-
**1q**	3,3-Dimethylpent-4-en-1-yl	48	22	>99	-14.3	87:13
(4*R*,5*S*)-**1a**[d]	Bu	99	34	98:2	-7.6	>99
**5a**	-	14	-[e]	98:2	-	-
**6a**	-	44	-[e]	98:2	-	-
**7a**	-	78	-[e]	98:2	-	-

Reaction conditions: Growth media: H_2O (100 mL), glucose (1 g), NaCl (0.5 g), casein peptone (0.4 g), meat peptone (1.0 g), yeast extract (0.3 g), NaOAc (0.2 g), riboflavin (1 mg); after 15 min *R. erythropolis* DSM 44534 (5 mL) was added. React. cond.: after reaching $OD_{600} \approx 1.3$ (usually after 24 h of incubation) the substrate (0.38 mmol) dissolved in EtOH (0.8 mL) was added. The reaction mixture was stirred under aerobic conditions in an Erlenmeyer flask (1 L) and the conversion was checked by GC. [a] GC-MS relative to an internal standard (γ-decalactone) see procedure at page S31. [b] Chiral GC. [c] In $CHCl_3$, λ=589 nm, 20 °C, $c \approx 1.0$. [d] Samples of this biotransformation were analyzed by chiral-GC, GC are reported at page S46. [e] Not isolated.

Growth inhibition profile as a function of lactone concentration

Whisky and cognac lactones demonstrated considerable toxicity to *R. erythropolis*. Experiments performed on increasing lactone concentration led to a significant reduction of microbial growth, which was measured by assessing the optical density (OD_{600}) values of the biotransformation broth. In particular, the lactone addition produced a growth interruption for all the concentrations tested (0.6, 0.8, 1.2 g/L). Noteworthy, only with a substrate concentration of 0.6 g/L the microbial growth started again within a few hours. In contrast, at a concentration of 0.8 g/L the growth interruption lasted some

days whereas with 1.2 g/L the fermentation was completely inhibited. Because of these experiments, we used a concentration of 0.6 g/L for all the substrates employed in our study.

Control biotransformations: absence of microorganisms or inactivated (autoclaved) cells

To exclude any catalytic contribution from the culture medium or from non-viable biomass, two independent control experiments were conducted under the same conditions used for the biotransformation. *Rhodococcus erythropolis* DSM 44534 was cultivated in 100 mL of rich medium for 7 days under the optimized growth conditions. The culture was centrifuged to separate the biomass from the spent medium. The cell pellet was resuspended in 100 mL of 25 mM phosphate buffer (pH 8.4) and subsequently autoclaved (121 °C, 10 min) to inactivate all enzymatic activities. Whisky lactone (60 mg in 0.8 mL EtOH) was added to this suspension, and the mixture was shaken at 130 rpm at 26 °C for 7 days. In parallel, the spent medium (pH 8.4) was filtered through a 0.21 μm membrane, transferred into a sterile Erlenmeyer flask, and supplemented with 60 mg of whisky lactone (in 0.6 mL EtOH). The mixture was shaken at 130 rpm at 26 °C for the same incubation time. After 7 days, both reactions were extracted following the general procedure for biotransformation described above. GC-MS analysis did not detect any formation of the corresponding α,β-unsaturated γ-lactone in either control experiment. These results demonstrate that neither medium components nor extracellular metabolites can promote the desaturation, which strictly requires living *R. erythropolis* cells.

(*R*)-5-Butyl-4-methylfuran-2(5*H*)-one (3a)

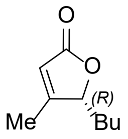

3a

Yield 43% (26 mg) as a pale-yellow oil; 98% purity by GC-MS; *ee* = 96% by chiral GC; $[\alpha]_D$= −7.4 ($CHCl_3$, *c*=1.1) *vs* lit. +2.7 ($CHCl_3$, *c*=1.1) [28]. ^{1}H and ^{13}C NMR spectra are consistent with those of racemic reference.

(*R*)-4-Methyl-5-propylfuran-2(5*H*)-one (3b)

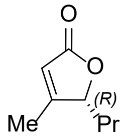

3b

Yield 32% (19 mg) as a pale-yellow; 97% purity by GC-MS; *ee* = 92% by chiral GC; $[\alpha]_D$= −6.4 ($CHCl_3$, *c*=0.8). ^{1}H and ^{13}C NMR spectra are consistent with those of racemic reference.

(*R*)-5-Isobutyl-4-methylfuran-2(5*H*)-one (3d)

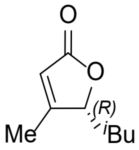

3d

Yield 14% (8 mg) as a pale-yellow oil; 96% purity by GC-MS; *ee* = 96% by chiral GC; $[\alpha]_D$= −4.0 ($CHCl_3$, *c*=0.8). ^{1}H and ^{13}C NMR spectra are consistent with those of racemic reference.

(*R*)-4-Methyl-5-pentylfuran-2(5*H*)-one (3e)

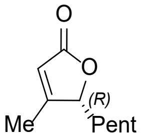

3e

Yield 43% (25 mg) as a pale-yellow oil; 98% purity by GC-MS; *ee* = 94% by chiral GC; $[\alpha]_D$= −6.7 ($CHCl_3$, *c*=1.1). ^{1}H and ^{13}C NMR spectra are consistent with those of racemic reference.

(*R*)-5-Isopentyl-4-methylfuran-2(5*H*)-one (3f)

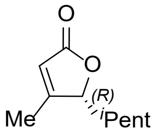

3f

Yield 39% (23 mg) as a pale-yellow oil; 97% purity by GC-MS; *ee* = 94% by chiral GC; $[\alpha]_D$= −6.5 ($CHCl_3$, *c*=1.1). ^{1}H and ^{13}C NMR spectra are consistent with those of racemic reference.

(*R*)-5-Hexyl-4-methylfuran-2(5*H*)-one (3g)

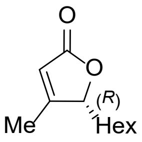

3g

Yield 35% (21 mg) as a pale-yellow oil; 97% purity by GC-MS; *ee* = 92% by chiral GC; $[\alpha]_D$= –3.0 ($CHCl_3$, *c*=1.0). ^1H and ^{13}C NMR spectra are consistent with those of racemic reference.

(*R*)-4-Methyl-5-(3-methylbut-2-en-1-yl)furan-2(5*H*)-one (3i)

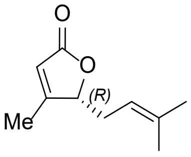

3i

Yield 10% (6 mg) as a pale-yellow oil; t_R=17.1 min, 88% purity by GC-MS; t_R=13.9 min (5*R*) and t_R=14.4 min (5*S*) by chiral GC; *ee* = 98% by chiral GC; $[\alpha]_D$= –18.0 ($CHCl_3$, *c*=0.8); **^{1}H-NMR** ($CDCl_3$, 400 MHz): δ 5.80 (m, 1H), 5.03 (m, 1H), 4.86 (t, J=5.5 Hz, 1H), 2.64 (m, 1H), 2.34 (m, 1H), 2.04 (d, J=1.5 Hz, 3H), 1.70 (s, 3H), 1.64 (s Hz, 3H); **^{13}C{^{1}H}-NMR** ($CDCl_3$, 101 MHz): δ 173.3, 168.3, 136.4, 117.5, 116.3, 84.4, 30.5, 25.9, 18.2, 14.1; GC-MS: m/z (%) 166(M^+,24), 98(63), 69(100), 53(13); the spectroscopic data were consistent with those reported in literature.[29]

(*R*)-5-(3,3-dimethylpent-4-en-1-yl)-4-methylfuran-2(5*H*)-one (3q)

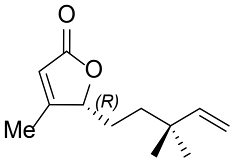

3q

Yield 22% (16 mg, carried out with substrate conc. of 0.33 g/L) as a pale-yellow oil; t_R=19.2 min, 97% purity by GC-MS; t_R=18.4 min (5*R*) and t_R=18.5 min (5*S*) by chiral GC; *ee* > 99% by chiral GC; $[\alpha]_D$= –17.5 ($CHCl_3$, *c*=0.8). ^1H and ^{13}C NMR spectra are consistent with those of racemic reference.

General procedure for biodesaturation in a bioreactor

A 5 L fermenter was loaded with 2 L of medium. The bioreactor was sterilized at 121 °C for 15 minutes, then the temperature and the stirring speed, were set to 26 °C and 230 rpm respectively, whilst aeration was secured by a continuous airflow (0.5 v/v/min.). Hence, the medium was inoculated with an active culture (50 mL) of *R. erythropolis* (DSM 44534) and after 18 hours lactone **1** (3.4-7.8 mmol) in EtOH (1.5-3 mL) was added at once. The pH slowly increased over 8.0 reaching 8.6-8.9 in the final phase of the fermentation. After 4-5 days since the addition of substrate, the biotransformation was stopped by addition of concentrated HCl to 4.0-4.5 pH, then of the broth was filtered through a celite pad. The pad was washed several times with EtOAc (4 x 200 mL). The organic phase was separated from the aqueous phase, which was extracted again with EtOAc (4 x 200 mL). The combined organic phase was dried over Na_2SO_4, and the solvent was removed under reduced pressure. The crude material was submitted to silica gel column chromatography purification affording the dehydrogenated lactone **3**. Yield and enantiomeric ratio are summarized in Figure 3 and Figure 10 of article.

(*R*)-5-Butyl-4-methylfuran-2(5*H*)-one (3a)

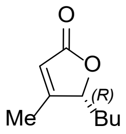

3a

Yield 54% (0.64 g, carried out on 7.8 mmol) as a pale-yellow oil; 99% purity by GC-MS; *ee* = 96% by chiral GC; $[\alpha]_D$ is consistent with that of lactone obtained from the biodesaturation carried out in the Erlenmeyer flask. Mass spectrometry data, ^{1}H and ^{13}C NMR spectra are consistent with those of racemic reference.

(*R*)-4-Methyl-5-pentylfuran-2(5*H*)-one (3e)

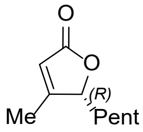

3e

Yield 51% (0.61 g, carried out on 7.8 mmol) as a pale-yellow oil; 99% purity by GC-MS; *ee* = 96% by chiral GC; $[\alpha]_D$ is consistent with that of lactone obtained from the biodesaturation carried out in the Erlenmeyer flask. Mass spectrometry data, ^{1}H and ^{13}C NMR spectra are consistent with those of racemic reference.

(*R*)-4-Methyl-5-pentylfuran-2(5*H*)-one (3q)

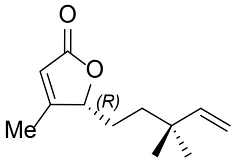

3q

Yield 33% (215 mg, carried out on 3.4 mmol) as a pale-yellow oil; 98% purity by GC-MS; *ee*>99% by chiral GC; $[\alpha]_D$ is consistent with that of lactone obtained from the biodesaturation carried out in the Erlenmeyer flask. Mass spectrometry data, ^{1}H and ^{13}C NMR spectra are consistent with those of racemic reference.

Hydrogenation of (5*R*)-3a and (5*R*)-3e for the determination of absolute stereochemical configuration

The chemical hydrogenations of (4*R*,5*R*)-**1a** and (4*R*,5*R*)-**1e** were carried out using Rh/Al_2O_3 catalyst, starting from the optically pure (5*R*)-**3a** and (5*R*)-**3e**, respectively (Scheme S7).

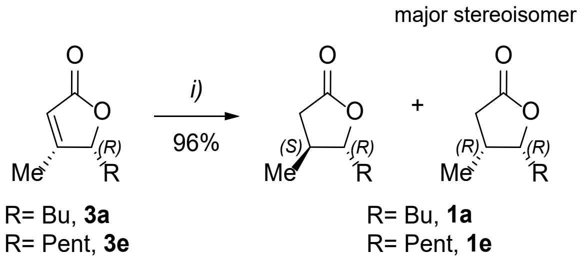

Scheme S7. Reaction conditions: *i*) H_2, Rh/Al_2O_3 (substrate/catalyst 5% w/w), EtOAc, rt.

General procedure

To a well stirred solution of (5*R*)-**3a** or (5*R*)-**3e** (0.64 mmol) in EtOAc (5 mL) was added Rh/Al_2O_3 (substrate/catalyst 5% w/w) and kept under an H_2 atmosphere until complete conversion of starting material (checked by GC-MS). Then, the mixture was filtered on a celite pad, which was washed with EtOAc (3 x 5 mL). The organic phase was dried over anhydrous Na_2SO_4 and concentrated under *vacuum*.

(+)-*cis*-Whisky lactone: (4*R*,5*R*)-5-Butyl-4-methyldihydrofuran-2(3*H*)-one ((4*R*,5*R*)-1a)

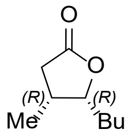

(4R,5R)-**1a**

Yield 96% (96 mg) as a pale-yellow oil; t_R=13.7 min (*trans*) and 14.4 min (*cis*), 98% purity by GC-MS; *trans*/*cis*=13:87 by GC-MS; $ee_{4R,5R}$=98% by chiral GC; $[\alpha]_D$= +65.0 ($CHCl_3$, *c*=1.1) *vs* lit. +72.6 (CCl_4, *c*=1.4).[30] Both NMR and mass spectrometry data were consistent with those of raceme mixture.

(+)-*cis*-Cognac lactone: (4*R*,5*R*)-5-Pentyl-4-methyldihydrofuran-2(3*H*)-one ((4*R*,5*R*)-1e)

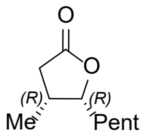

(4*R*,5*R*)-**1e**

Yield 98% (100 mg) as a pale-yellow oil; t_R=15.9 min (*trans*) and 16.7 min (*cis*), 98% purity by GC-MS; *trans*/*cis*=14:86 by GC-MS; $ee_{4R,5R}$=98% by chiral GC; $[\alpha]_D$= +46.2 ($CHCl_3$, *c*=1.5) *vs* lit. +63.3 (CCl_4, *c*=1.12).[19] Both NMR and mass spectrometry data were consistent with those of raceme mixture.

Monitoring enzymatic hydrolysis

The time course of enzymatic hydrolysis of **1a** was monitored by GC-MS using γ-decalactone as an internal standard for GC calibration. Among various possible lactones, γ-decalactone was selected because its retention time (t_R=17.68 min) does not overlap with that of **1a** and **3a**, while its solubility in water is expected to be similar. The GC-MS integrals of **1a** and **3a** were corrected using factors of 0.96 and 2.46, respectively, relative to the integral of γ-decalactone.
The biotransformation was carried out in a fermenter as described on page S29. A solution of γ-decalactone in H_2O/EtOH (98:2, 0.6 g/L, 40 mL) was added to each 20 mL aliquot of the reaction medium. Aliquots were withdrawn at time points specified in Table S2. Each aliquot was shaken at 120 rpm for 10 minutes and then divided into portions.
For one portion, the pH was adjusted to 2-3 using HCl (0.1 M). Each portion was centrifuged (6000 rpm) and then extracted with EtOAc (3 x 10 mL). The combined organic phases were concentrated under reduced pressure to approximately one-quarter of the initial volume, dried over Na_2SO_4, filtered directly into a 10 mL volumetric flask. The Na_2SO_4 was washed with EtOAc until the final volume reached 10 mL. Finally, the samples were analyzed by GC-MS. The integral areas were corrected and normalized relative to γ-decalactone. The concentrations and diastereomeric excess values are summarized in Table S2, and the plot concentration vs time is shown in Figure 5 of article.

Table S2. Monitoring hydrolysis by GC-MS.

	**Time** (hours)				
	0	14	20	24	238
**[1a]** at biotransformation pH (mg/L)	600	252	200	84	<10
**[1a]** after acidification (mg/L)	600	445	489	512	454
**[3a]** at biotransformation pH (mg/L)	-[a]	-[a]	-[a]	24	75
***de**$_{trans}$* (%)	8	32	42	64	>99
**pH**	7.1	7.7	8.0	8.1	8.3

[a] Not detected.

Derivatization of intermediate I as methylester (4a)

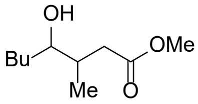

4a

After 24 hours of biotransformation of **1a** (procedure described at page S17), the pH of a sample (10 mL) was adjusted to 5.5-6.0 with HCl (0.05 M), extracted with Et_2O (3 x 10 mL) and concentrated under reduced pressure without heating. The concentrated solution was treated with CH_2N_2 (1 mL) and analyzed by GC, showing the presence of **4a** and **1a** not hydrolyzed.

Data: t_R=14.35 min (*anti*) + 13.72 min (*syn*), *syn/anti* 8:2 by GC-MS (this diastereomeric ratio is not correct, since **4a** partially cyclizes to **1a** already in the injector of GC; GC-MS: m/z (%) 157(M-31,2), 141(1), 99(100).

Synthesis of deuterated substrates

Synthesis of 2a-1,1,4-d_3 and 1a-5-*d*

The synthesis of the deuterated substrates **2a**-1,1,4-d_3 and **1a**-5-*d* is shown in Scheme S8. First, whisky lactone **1a** was hydrolyzed with KOH in MeOH and H_2O. Subsequently, the carboxylate intermediate was O-alkylated with ^{i}PrI in DMF, yielding the *i*-propyl hydroxyester, which was oxidized with the Dess-Martin periodinane (DMP), resulting in the formation of ketoester **7**. Then, $LiAlD_4$ (1.5 eq.) was used to reduce both carbonyl groups of **7a** to obtain **2a**-1,1,4-d_3, while a lesser amount of $LiAlD_4$ (0.5 eq.) was employed to reduce regioselectively the ketone carbonyl of **7a**. Finally, treatment of the deuterium-labeled hydroxyester with TFA gave lactone **1a**-5-*d*.

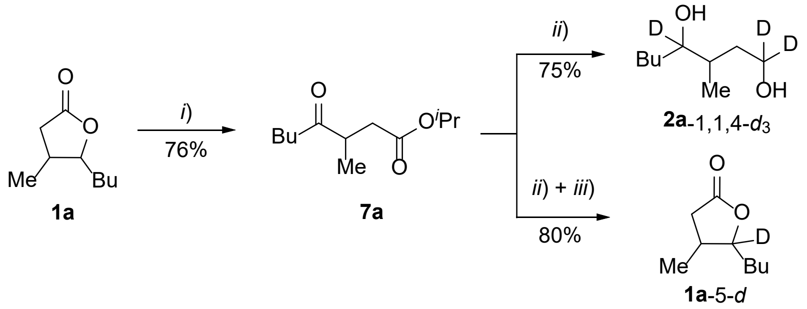

Scheme S8. Reaction conditions: *i*): a) KOH, H_2O, MeOH, rt; b) 2-iodopropano, DMF, 40 °C; c) Dess-Martin periodinane (DMP), CH_2Cl_2, 0 °C to rt. *ii*) $LiAlD_4$, Et_2O, 0 °C to rt. *iii*) TFA, CH_2Cl_2, 0 °C to rt.

Isopropyl 3-methyl-4-oxooctanoate (7a)

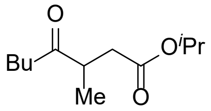

7a

To a well stirred solution of **1a** (3.0 g, 19.2 mmol) in MeOH (30 mL) a solution of KOH (1.0 g, 17.9 mmol) in H_2O (3 mL) was added. The heterogeneous mixture was stirred for 5 hours at room temperature and then concentrated under *vacuum* to give a viscous oil. The latter was treated with Et_2O (4 x 10 mL) and concentrated under reduced pressure. This procedure was repeated at least 4 times in such a way to eliminate all traces of H_2O and MeOH. The crude material was left under a high *vacuum* for 6 hours. Then, to a solution of the crude mixture in anhydrous DMF (20 mL) was added 2-iodopropane (3.5 g, 20.6 mmol) and left to stir at 40 °C under a N_2 atmosphere. After 14 hours, the reaction mixture was diluted with brine (sat., 70 mL) and then extracted with Et_2O (5 x 20 mL). The combined organic phases were extracted with brine (sat., 40 mL), dried over Na_2SO_4 and concentrated under reduced pressure to give the corresponding hydroxyester, which was of sufficient purity for the next step.[4]

To an ice-cooled solution of crude material in CH_2Cl_2 (30 mL) was added DMP (7.3 g, 17.2 mmol), then the reaction mixture was left to stir at room temperature. After 1 hour it was filtered on a celite pad, which was washed with CH_2Cl_2 (3 x 30 mL). The organic phase was dried over anhydrous Na_2SO_4 and concentrated under *vacuum* affording **7a** as a yellow oil. The ketone was purified by silica gel column chromatography using *n*-hexane/EtOAc (90:10) as eluent and then by bulb-to-bulb distillation (110 °C, 0.1-0.3 mbar).

Yield 76% (2.6 g) as a colorless oil; t_R=15.8 min, >99% purity by GC-MS; **^{1}H-NMR** ($CDCl_3$, 400 MHz): δ 5.43 (m, 1H), 2.97 (m, 1H), 2.69 (m, 1H), 2.53-2.44 (m, 2H), 2.22 (m, 1H), 1.59-1.47 (m, 2H), 1.34-1.23 (m, 2H), 1.17 (m, 3H+3H), 1.08 (d, J=7.2 Hz, 3H), 0.87 (t, J=7.3 Hz, 3H); **^{13}C{^{1}H}-NMR** ($CDCl_3$, 101 MHz): δ 213.0, 171.9, 67.9, 42.1, 41.0, 37.5, 25.8, 22.4, 21.9, 16.8, 13.9; GC-MS: m/z (%) 214(M^+,1), 155(75), 85(100), 57(74).

3-Methyloctane-1,1,4-d_3-1,4-diol (2a-1,1,4-d_3)

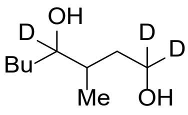

2a-1,1,4-d_3

To an ice-cooled and well stirred solution of **7** (1.4 g, 6.5 mmol) in Et_2O (20 mL) was portion wise added $LiAlD_4$ (410 mg, 9.8 mmol), usually over 5 minutes. Then, the mixture was left to reach room temperature and after 1 hour was ice-cooled, quenched with a Seignette solution (sat., 20 mL) and left to stir over 1 hour. Then, it was extracted with EtOAc (3 x 20 mL). The combined organic phase was dried over anhydrous Na_2SO_4 and concentrated under *vacuum* affording

2a-1,1,4-d_3 as a yellow liquid, which was purified by silica gel column chromatography using *n*-hexane/EtOAc (50:50) as eluent.

Yield 75% (800 mg) as a yellow liquid; t_R=14.6 min, t_R=14.7 min, 98% purity (*anti/syn*=51:49) by GC-MS; **^{1}H-NMR** ($CDCl_3$, 400 MHz): δ 3.35-2.62 (bs, OH, 1H+1H), 1.80-1.59 (m, 2H), 1.55-1.23 (m, 7H), 0.90 (m, 6H); **^{13}C{^{1}H}-NMR** ($CDCl_3$, 101 MHz): δ 75.2 (t, BuCDOH , J_{C-D}=22.0 Hz), 74.4 (t, BuCDOH, J_{C-D}=21.6 Hz), 59.4 (m+m, CD_2), 36.3, 35.1, 34.0, 33.2, 28.7, 28.1, 22.84, 22.82, 16.5, 14.1, 13.9; **^{2}H-NMR** ($CHCl_3$, 61.4 MHz): δ 3.70 (bs, 2D), 3.59 (bs, 3D), 3.38 (s, 1dD); GC-MS (first diastereoisomer): m/z (%) 163(M^+,1), 88(100), 70(45), 58(84); GC-MS (second diastereoisomer): m/z (%) 163(M^+, 2), 88(100), 70(34), 58(75).

5-Butyl-4-methyldihydrofuran-2(3*H*)-one-5-*d* (1a-5-*d*)

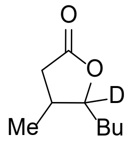

1a-5-*d*

To an ice-cooled and well stirred solution of **7** (400 mg, 1.9 mmol) in Et_2O (15 mL) was portion wise added $LiAlD_4$ (39 mg, 0.9 mmol), usually over 5 minutes. Then, the mixture was left to reach room temperature and after 1 hour the reaction was ice-cooled, quenched with a Seignette solution (sat., 15 mL) and left to stir. After 1 hour, it was extracted with EtOAc (3 x 15 mL), the combined organic phase was dried over anhydrous Na_2SO_4 and concentrated under *vacuum*.

Then crude material was ice-cooled, CH_2Cl_2 (1 mL), TFA (20 μL) was added, and left to stir at room temperature. After 12 hours, CH_2Cl_2 (10 mL) was added, it was ice-cooled, quenched with a solution of $NaHCO_3$ (sat., 10 mL) and left to stir at room temperature. After 30 minutes, the mixture was extracted with CH_2Cl_2 (3 x 10 mL), the combined organic phase was extracted with brine (sat., 10 mL), dried over anhydrous Na_2SO_4 and concentrated under *vacuum* affording **1a-**5-*d* as pale-yellow oil, which was purified by silica gel column chromatography using *n*-hexane/EtOAc (80:20) as eluent.

Yield 80% (150 mg) as a yellow liquid; t_R=13.6 min (*trans*) and 14.4 min (*cis*), 98% purity by GC-MS; *trans*/*cis*=54:46 by GC-MS and by ^{1}H-NMR; **^{1}H-NMR** ($CDCl_3$, 400 MHz): δ 2.68 (m, 1H), 2.59 (m, 0.54H), 2.22-2.13 (m, 1.46H), 1.69-1.59 (m, 1.54H), 1.55-1.46 (m, 1.46H), 1.35 (m, 1H+2H), 1.12 (d, J=6.5 Hz, 1.38H), 1.00 (d, J=7.0 Hz, 1.62H), 0.91 (m, 3H); **^{13}C{^{1}H}-NMR** ($CDCl_3$, 101 MHz) (*trans*): δ 176.6, 87.0 (t, BuCDO, J_{C-D}=22.8 Hz), 37.1, 36.0, 33.6, 27.8, 22.47, 17.5, 13.87; (*cis*): δ 176.9, 83.3 (t, BuCDO, J_{C-D}=22.8 Hz), 37.6, 32.9, 29.5, 28.0, 22.51, 13.90, 13.80; **^{2}H-NMR** ($CHCl_3$, 61.4 MHz): δ 4.41 (bs, *cis* diast.), δ 3.99 (bs, *trans* diast.); GC-MS (*trans*): m/z (%) 157(M^+, 2), 100(100), 88(20), 72(33); GC-MS (*cis*): m/z (%) 157(M^+,3), 100(100), 88(23), 70(36).

Synthesis of 5-butyl-4-methyldihydrofuran-2(3*H*)-one-3,4-d_2 (1a-3,4-d_2)

The synthesis of **1a**-3,4-d_2 is shown in Scheme 9. In a continuous-flow high-pressure hydrogenation apparatus,[31] feed with D_2 generated by electrolysis of D_2O, substrate **3a** was reduced to **1a** using Pd/C as catalyst.

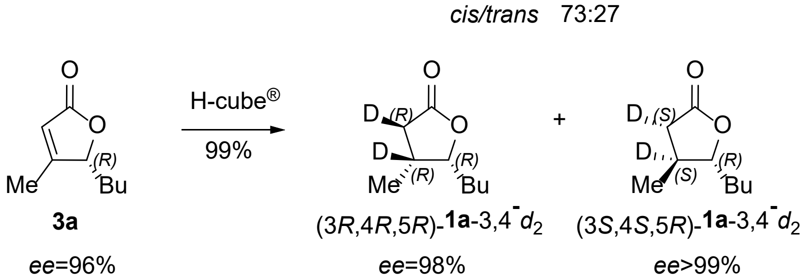

Scheme S9. Reaction conditions: substrate 0.046 M in EtOAc, T=25 °C, Pd/C cartridge, D_2 (40 bar), flow rate 0.7 mL min^{-1}.

A solution of (5*R*)-**3a** (250 mg, 1.6 mmol) in EtOAc (35 mL) was circulated into a continuous-flow high pressure hydrogenation reactor equipped with a cartridge filled with Pd/C (5% w/w loading) at a flow rate of 0.7 mL min^{-1}. The D_2 (40 bar) was *in situ* generated by means of D_2O electrolysis (65 mL, purity >99%). Before starting the reduction, the Pd/C cartridge was conditioned by flowing EtOAc (10 mL, for 10 minutes) under D_2 pressure and with a flow rate of 0.7 mL min^{-1}. After three hours, the collected solution was concentrated under reduced pressure affording the crude material, which was of sufficient purity to be used in biotransformation experiment. Yield 99% (250 mg) as a yellow liquid; t_R=13.7 min (*trans*) and 14.4 min (*cis*), > 99% purity by GC-MS, *cis/trans*=73:27 by GC-MS and by ^{1}H-NMR.

An aliquot of *cis/trans* mixture of **1a**-3,4-d_2 was submitted to silica gel column chromatography separation using *n*-hexane/EtOAc (80:20) eluent.

(3*S*,4*S*,5*R*)-5-Butyl-4-methyldihydrofuran-2(3*H*)-one-3,4-*d*2 (*trans*-1a-3,4-*d*2)

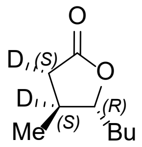

(3*S*,4*S*,5*R*)-**1a**-3,4-*d*2

Trans/*cis*=95:5 by GC-MS and by ^{1}H-NMR; *ee*>99% by chiral GC; **^{1}H-NMR** ($CDCl_3$, 400 MHz): δ 4.42 (m, 0.05H), 3.99 (m, 0.95H), 2.16 (m, 1H), 1.70-1.58 (m, 2H), 1.49 (m, 1H), 1.40-1.32 (m, 1H+2H), 1.12 (s, 2.85H), 0.99 (s, 0.15H), 0.91 (t, J=7.2 Hz, 3H); **^{13}C{^{1}H}-NMR** ($CDCl_3$, 101 MHz) (*trans*): δ 176.7, 87.5, 36.9 (t, J_{C-D}=20.9 Hz), 35.7 (t, J_{C-D}=20.2 Hz), 33.8, 27.9, 22.6, 17.5, 14.0; **^{2}H-NMR** ($CHCl_3$, 61.4 MHz): δ 2.65-2.61 (d, J_{D-H}=2.7 Hz, CDH, 1D), 2.17 (s, CDMe, 1D); GC-MS (*trans*): m/z (%) 158(M^+,2), 101(100), 73(36), 69(24).

(3*R*,4*R*,5*R*)-5-Butyl-4-methyldihydrofuran-2(3*H*)-one-3,4-*d*2 (*cis*-1a-3,4-*d*2)

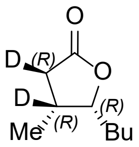

(3*R*,4*R*,5*R*)-**1a**-3,4-*d*2

Cis/trans=80:20 by GC-MS and ^{1}H-NMR; *ee* = 98% by chiral GC; **^{1}H-NMR** ($CDCl_3$, 400 MHz): δ 4.42 (m, 0.80), 3.98 (m, 0.20H), 2.12 (m, 1H), 1.61 (m, 1H), 1.55-1.38 (m, 2H), 1.37-1.25 (m, 1H+2H), 1.09 (s, 0.6H), 0.96 (s, 2.4H), 0.88 (m, 3H); **^{13}C{^{1}H}-NMR** ($CDCl_3$, 101 MHz) (*cis*): δ 176.9, 83.6, 37.1 (t, J_{C-D}=20.1 Hz), 32.6 (t, J_{C-D}=20.6 Hz), 29.6, 28.1, 22.5, 13.9, 13.7; **^{2}H-NMR** ($CHCl_3$, 61.4 MHz): δ 2.68-2.64 (d, J_{D-H}=2.7 Hz, CDH, 1D), 2.55 (s, CDMe(*cis*+*trans*), 0.8D), 2.17 (s, CDMe(*trans*), 0.2D); GC-MS (*cis*): m/z (%) 158(M^+,2), 101(100), 87(14), 69(16).

Biodesaturation of deuterium labeled substrates or of 3a in D_2O/water medium

The biotransformation conditions described in the general procedure at page S26 were applied, using a deuterium-labeled substrate or using **3a** in a medium with a specific H_2O/D_2O. The results are described in Table S3.

Table S3. Labeled dehydrogenated γ-lactones obtained from deuterium labeled substrates or using **3a** in medium with different D_2O/water ratios.

Exp.	Substrate	H_2O/D_2O	Time	Yield	3a [D/H][a]	
			[d]	[%]	C(3)	C(5)
**A**	**1a**-5-*d*	100	10	17	-	60:40
**B**	**2a**-1,1,4-d_3	100	9	16	-	54:44
**C**	**1a**	80:20	4	43	6:94	8:92
**D**	**1a**	68:32	4	40	8:92	10:90
**E**	*cis*-**1a**-3,4-d_2	100	10	18	56:44	-

[a] By ^{1}H-NMR.

A) (*R*)-3a+(*R*)-3a-5-*d* from 1a-5-*d*

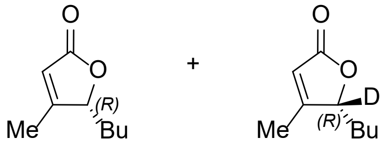

3a **3a**-5-*d*

Yield 17% (11 mg) as a yellow liquid; t_R=15.2 min, 96% purity by GC-MS; *ee* = 94% by chiral GC; **3a**/**3a**-5-*d* 40:60 by ^{1}HNMR; optical rotation value, ^{1}H and ^{13}C chemical shifts and integrals are consistent with those of **3a**, with the exception of the following NMR signals: **^{1}H-NMR** ($CDCl_3$, 400 MHz): δ 4.82 (m, 0.40H); **^{13}C{^{1}H}-NMR** ($CDCl_3$, 101 MHz) δ 84.4 (t, J_{C-D}=22.8 Hz); **^{2}H-NMR** ($CHCl_3$, 61.4 MHz): δ 4.82 (s); GC-MS (**3a**+**3a**-5-*d*): m/z (%) 155(M^+,15), 125(45), 98(100), 69(53).

B) (*R*)-3a+(*R*)-3a-5-*d* from 2-1,1,4-d_3

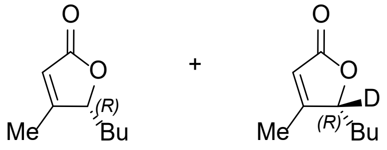

3a **3a**-5-*d*

Yield 16% (10 mg) as a yellow liquid; t_R=15.2 min, 98% purity by GC-MS; *ee* = 96% by chiral GC; **3a**/**3a**-5-*d* 30:70 by ^{1}HNMR; optical rotation value, ^{1}H and ^{13}C chemical shifts and integrals are consistent with those of the unlabeled **3a**, with the exception of following signals: **^{1}H-NMR** ($CDCl_3$, 400 MHz): δ 4.82 (m, 0.30H); **^{13}C{^{1}H}-NMR** ($CDCl_3$, 101 MHz) δ 84.4 (t, J_{C-D}=22.8 Hz); **^{2}H-NMR** ($CHCl_3$, 61.4 MHz): δ 4.83; GC-MS (**3a**+**3a**-5-*d*): m/z (%) 155(M^+,16), 125(26), 98(100), 70(28).

(*R*)-3a+(*R*)-3a-3,5-d_2 from 1a in medium with different D_2O/water ratio: C) 20:80; D) 32:68

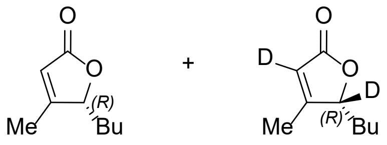

3a **3a**-3,5-d_2

Yield 43% for B) and 40% for C) (24-26 mg) as a yellow liquid; t_R=15.3 min, 98% purity by GC-MS either for B) and C) transformations; *ee* = 78% by chiral GC for both experiments C) and D); ^{1}H and ^{13}C chemical shifts and integrals are consistent with those of the unlabeled **3a**, with the exception of following NMR signals: transformation C) **^{1}H-NMR** ($CDCl_3$, 400 MHz): δ 5.76 (m, 0.94H), 4.80 (m, 0.92H); transformation D) **^{1}H-NMR** ($CDCl_3$, 400 MHz): δ 5.76 (m,

0.92H), 4.80 (m, 0.90H); **^{13}C{^{1}H}-NMR** (CDCl$_3$, 101 MHz) δ 173.4, 168.6, 117.0, 84.7, 31.7, 26.5, 22.5, 14.0, 13.9; **^{2}H-NMR** (CHCl$_3$, 61.4 MHz): δ 5.81 (s), 4.82 (s,); GC-MS (**3a**-3,5-*d*$_2$): m/z (%) 155(M$^+$,1), 125(46), 97(100), 69(89).

E) (*R*)-3a+(*R*)-3a-3-*d* from *cis*-1a-3,4-*d*$_2$

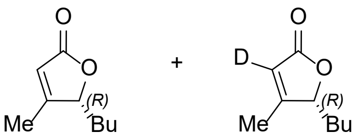

3a **3a**-3-*d*

Yield 18% (12 mg) as a yellow liquid; t$_R$=15.3 min, 98% purity by GC-MS; *ee* = 78% by chiral GC; ^{1}H and ^{13}C chemical shifts and integrals are consistent with the unlabeled **3a**, with the exception of **^{1}H-NMR** (CDCl$_3$, 400 MHz): δ 5.80 (m, 0.56H); **^{13}C{^{1}H}-NMR** (CDCl$_3$, 101 MHz): δ 116.8 (t, J$_{C-D}$=27.3 Hz); **^{2}H-NMR** (CHCl$_3$, 61.4 MHz): δ 5.83 (s); GC-MS (**3a**-3-*d*): m/z (%) 155(M$^+$, 14), 98(100), 85(35), 70(63).

Synthesis of intermediate 6a

The intermediate **6a** was obtained by oxidation of **5a** with MnO_2 (Scheme S10).

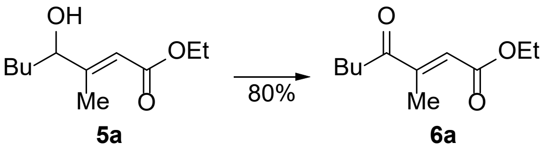

Scheme S10. Reaction conditions: MnO_2, refluxing CH_2Cl_2.

Ethyl (*E*)-3-methyl-4-oxooct-2-enoate (6a)

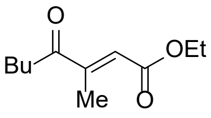

6a

To a refluxing and well stirred solution of **5a** (500 mg, 2.5 mmol) in CH_2Cl_2 (20 mL) was added MnO_2 (2.5 g). After 8 hours the reaction mixture was filtered on a celite pad, which was washed with CH_2Cl_2 (3 x 20 mL). The organic phase was dried over anhydrous Na_2SO_4 and concentrated under *vacuum* affording **6a** as a yellow liquid which was purified by silica gel column chromatography using *n*-hexane/EtOAc (90:10) as eluent.

Yield 80% (400 mg) as a yellow liquid; t_R=16.5 min, 96% purity by GC-MS; *E*>99% by ^{1}H-NMR; **^{1}H-NMR** ($CDCl_3$, 400 MHz): δ 6.49 (m, 1H), 4.28-4.13 (m, 2H), 2.73-2.59 (m, 2H), 2.18 (m, 3H), 1.64-1.49 (m, 2H), 1.33-1.25 (m, 2H+3H), 0.88 (m, 3H); **^{13}C{^{1}H}-NMR** ($CDCl_3$, 101 MHz): δ 202.5, 166.3, 150.7, 125.2, 60.8, 38.0, 26.4, 22.4, 14.3, 13.9, 13.4; GC-MS: m/z (%) 198(M^+,1), 141(60), 125(100), 110(94).[32] ESI-HRMS (m/z): calcd. for $C_{11}H_{18}O_3Na^+$ $[M+Na]^+$ 221.1148, found 221.1145.

Synthesis of enantiomerically enriched (4*R*,5*S*)-1a

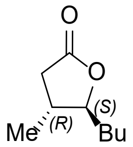

1a

The (4*R*,5*S*)-**1a** was prepared following the reaction conditions described by Boratyński,[33] but starting from whisky lactone instead from diol *anti*-**2a** carrying out the biotransformation into the bioreactor as described at page S28. However, the reaction was stopped after 24 hours, and the work-up was similar with the exception that reaction mixture was not acidified.

Yield 37% (0.44 g) as a pale-yellow oil; t_R=13.7 min (*trans*) and 14.4 min (*cis*), 95% purity by GC-MS; *trans*/*cis*=74:26 by GC-MS; $ee_{4R,5S}$=74% and $ee_{4R,5R}$=16% by chiral GC; $[\alpha]_D$= –51.0 ($CHCl_3$, *c*=1.1) *vs* lit. –89.0 (MeOH, *c*=1.0).[34] Both NMR spectroscopic and mass spectrometry data were consistent with those of raceme mixture.

Chemical hydrolysis of 1a and H/D exchange monitoring at α-position by ^{1}H-NMR

To a solution of Tris buffer (50 mM, pH=8.5) in D_2O (500 µL) was added a solution of **1a** (15 mg, 0.10 mmol) in DMSO-*d*$_6$ (45 µL) into an NMR tube (5 mm). The reaction was left to react at 26 °C for 5 days. Since after 1 day and 5 days no hydrolysis was observed (by ^{1}HNMR, Figure S1A and S1B), a solution of **1a** (15 mg, 0.10 mmol) and (10 mg) K_2CO_3 (10 mg) in D_2O/DMSO-*d*$_6$ (500 µL, 90:10) into an NMR tube (5 mm) was heated for 3 hours to 60 °C. The ^{1}H-NMR reaction monitoring shown that: i) **1a** is not hydrolysable at biotransformation conditions;, *ii*) chemical hydrolysis of **1a** to give **I** requires higher temperatures (T>60 °C) but it is not highly diastereoselective (44:56 *syn/anti*, by integration of *syn* and *anti* BuC*H*O signals, Figure S1C); *iii*) H/D exchange at α-position of both **1a** and **I** intermediate do not occur, since the integration of CH_2CO_2 signals do not change.

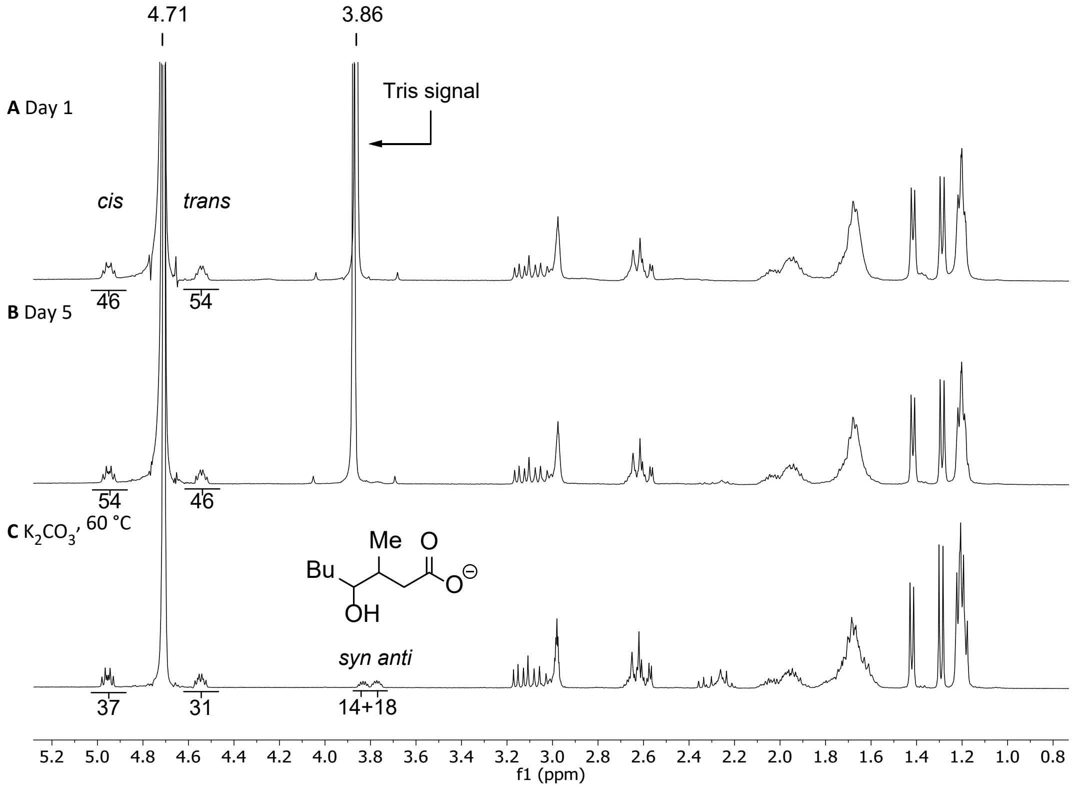

Figure S1. ^{1}H-NMR spectra (400 MHz) of **1a** in D_2O: A) and B) at pH 8.5 (tris buffer) and at 26 °C, after 1 day and 5 days, respectively; C) at pH>9 (K_2CO_3) at 60 °C, after 3 hours.

(*R*)-2,2-Dimethyl-4-(3-methyl-5-oxo-2,5-dihydrofuran-2-yl)butanal (8)

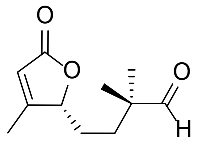

8

To a solution of **3q** (158 mg, 0.77 mmol) and NMO (50% w/w in H_2O, 0.2 mL, 0.97 mmol) in H_2O/acetone (1:1, 5 mL), and *t*-BuOH (1 mL) was added $K_2OsO_4 \cdot 2\ H_2O$ (2.5 mg, 7 µmol) at rt and under a N_2 atmosphere. The mixture was stirred until the starting lactone was no longer detectable (by TLC, usually after 8 hours). Hence, the reaction was quenched by addition of $Na_2S_2O_4$ (88 mg, 0.5 mmol) and H_2O (10 mL) and the mixture was vigorously stirred for 30 min., followed by filtration on a celite pad. The filtrate was acidified with a solution of HCl (0.1 M, pH around 4) and the acetone was evaporated under *vacuum*. The obtained solution was extracted with EtOAc (3 x 50 mL) and the combined organic phase was washed with brine (sat., 40 mL), dried over anhydrous Na_2SO_4 and concentrated under *vacuum*. To a solution of crude diol (160 mg) in THF (15 mL) under stirring was added a solution of $NaIO_4$ (200 mg, 0.94 mmol) in H_2O (5 mL). After 20 min. the reaction mixture was filtered, the filtrate was extracted with ethyl acetate (2 x 30 mL) and the combined organic phase was washed with brine (sat., 30 mL), dried over anhydrous Na_2SO_4 and concentrated under *vacuum*. The residue was purified through silica gel column chromatography (*n*-hexane/EtOAc, 7:3) to afford aldehyde **8**.

Yield 71% (108 mg) as a slightly yellow liquid; t_R=20.7 min, 92% purity by GC-MS; $[\alpha]_D$= –5.0 ($CHCl_3$, *c*=1.1); **^{1}H-NMR** ($CDCl_3$, 400 MHz): δ 9.40 (s, 1H), 5.79 (m, 1H), 4.80 (m, 1H), 2.03 (dd, 2H), J=0.8 and 1.6 Hz), 2.18 (m, 3H), 1.84 (m, 1H), 1.35-1.56 (m, 3H), 0.88 (s+s, 3H+3H); **^{13}C{^{1}H}-NMR** ($CDCl_3$, 101 MHz): δ 205.6, 173.1, 168.2, 117.4, 84.2, 45.3, 31.1, 26.7, 21.6, 21.5, 13.9; GC-MS: m/z (%) 196(M^+,1), 168(37), 149(5), 121(11), 111(100). The spectroscopic data were consistent with those reported in literature.[35]

Computational study

Density functional theory (DFT) and post Hartree-Fock (HF) ab initio calculation methods were performed with Gaussian 16.6.13. Molecular geometry optimizations were performed using the B3LYP functional,[36] using the 6-311+G(d,p) basis set[37] *in vacuum*. Solvation energy was calculated as single point using B3LYP/6311+G(d,p), and SCRF=SMD (solvent=H_20).[38] Thermodynamics were computed within the Rigid-Rotor-Harmonic-Oscillator (RRHO) approximation at 298.15K and grouped in a term G_{corr}=G – E(el) where G is the free energy of the species after all thermodynamics corrections and E_{el} is the electronic potential energy of the species. Electronic energies were computed using functional MP2/6311+G(d,p).[39] The free energy of solvation for each geometry was determined as $\Delta G_{solv}=E_{el,solv} - E_{el,vacuum}$. The final free energy of a species is therefore calculated as: $\Delta G=E_{el}$ (MP2) + G_{corr} (B3LYP) + ΔG_{solv} (B3LYP). 3D renderings of optimized structures were generated using GaussView 6.0.16 and CYLview[40] were used to generate the final structures. In Tables S4-S6 the computed energies are summarized.

Table S4. Energies obtained with B3LYP/6311+G(d,p) in *vacuum*.

	Hydroxyacids				***Ketoacids***			
**Species**	(*E*)-**II**	*anti*-**I**	*gauche*-**I**	(*Z*)-**II**	(*E*)-**V**	*anti*-**IV**	*gauche*-**IV**	(*Z*)-**V**
**E** (Hartree)	-577.893758	-579.1218	-579.13588	-577.909058	-576.700956	-577.91969	-577.921307	-576.699678
**ΔH** (Hartree)	-577.651335	-578.855792	-578.869851	-577.666814	-576.482364	-577.677537	-577.679285	-576.480795
**S**	123.536	124.91	120.158	117.897	122.243	124.376	121.809	117.897
**ΔG** (Hartree)	-577.71003	-578.91514	-578.926942	-577.722831	-576.540445	-577.736633	-577.73716	-576.537335
$\mathbf{E_{corr.}}$ (Hartree)	0.183728	0.20666	0.208938	0.186227	0.160511	0.183057	0.184147	0.162343
$\mathbf{\Delta H_{Norm.}}$ (Hartree)	0.00	0.00	-0.014059	-0.015479	0.00	0.00	-0.001748	0.001569
**ΔH** (kcal mol^{-1})	0.00	0.00	-8.82	-9.71	0.00	0.00	-1.10	0.98
$\mathbf{\Delta G_{Norm.}}$ (Hartree)	0.00	0.00	-0.011802	-0.012801	0.00	0.00	-0.000527	0.00311
**ΔG** (kcal mol^{-1})	0.00	0.00	**-7.41**	**-8.03**	0.00	0.00	**-0.33**	**1.95**

Table S5. Energies obtained with B3LYP/6311+G(d,p), SCRF=SMD (solvent=H_20).

	Hydroxyacids				***Ketoacids***			
**Species**	(*E*)-**II**	*anti*-**I**	*gauche*-**I**	(*Z*)-**II**	(*E*)-**V**	*anti*-**IV**	*gauche*-**IV**	(*Z*)-**V**
**E** (Hartree)	-577.998848	-579.228746	-579.233183	-578.007086	-576.799637	-578.024388	-578.024718	-576.7939
**ΔH** (Hartree)	-577.75872	-578.964735	-578.968193	-577.76566	-576.582288	-577.783394	-577.783922	-576.576206
**S**	114.539	115.757	113.61742	117.897	116.22	119.087	116.477	111.508
**ΔG** (Hartree)	-577.813141	-579.019735	-579.022176	-577.818619	-576.637508	-577.839976	-577.839264	-576.629187
$\mathbf{E_{corr.}}$ (Hartree)	0.185707	0.209011	0.211007	0.188467	0.162129	0.184412	0.185454	0.185454
$\mathbf{\Delta H_{Norm.}}$ (Hartree)	0.00	0.00	-0.003458	-0.00694	0.00	0.00	-0.000528	0.006082
**ΔH** (kcal mol^{-1})	0.00	0.00	-2.17	-4.35	0.00	0.00	-0.33	3.82
$\mathbf{\Delta G_{Norm.}}$ (Hartree)	0.00	0.00	-0.002441	-0.005478	0.00	0.00	0.000712	0.008321
**ΔG** (kcal mol^{-1})	0.00	0.00	**-1.53**	**-3.44**	0.00	0.00	**0.45**	**5.22**

Table S6. Energies obtained using MP2/6311+G(d,p) in *vacuum*.

	Hydroxyacids				*Ketoacids*			
**Species**	(*E*)-**II**	*anti*-**I**	*gauche*-**I**	(*Z*)-**II**	(*E*)-**V**	*anti*-**IV**	*gauche*-**IV**	(*Z*)-**V**
**E** (Hartree)	-576.247997	-577.463597	-577.477529	-576.264656	-575.066517	-576.274592	-576.277907	-575.068979
**ΔG_{calc.}** (Hartree)	-576.169359	-577.363883	-577.365894	-576.176457	-575.004687	-576.196233	-576.197171	-575.000858
**ΔG_{norm.}** (Hartree)	0	0	-0.002011	-0.007098	0	0	-0.000938	0.003829
**ΔG** (kcal mol^{-1})	0.00	0.00	**-1.26**	**-4.45**	0.00	0.00	**-0.59**	**2.40**

Coordinates of computed species

(*E*)-**II**

C	1.17650500	0.67472900	-0.19569000
C	-0.30113000	0.95012000	0.03156100
H	-0.60760600	1.76456400	-0.63787500
C	1.70724500	-0.54559100	-0.01153200
H	1.05142300	-1.36785900	0.26496500
C	3.18831600	-0.95162800	-0.01449900
O	3.94718700	-0.42461200	-0.86233700
O	3.45788300	-1.79651800	0.87533600
C	-1.26048300	-0.22446000	-0.19552900
H	-1.05084300	-1.01134700	0.53716100
H	-1.05641200	-0.65362700	-1.18347700
C	-2.73525500	0.18051600	-0.09725600
H	-2.95668100	0.93452300	-0.86568100
H	-2.90544400	0.66809400	0.86870400
C	-3.70380600	-0.99663500	-0.25830900
H	-3.49093900	-1.74547800	0.51454300
H	-3.51743200	-1.49338800	-1.21891900
O	-0.50644300	1.50456700	1.36401000
H	0.13411100	1.05984500	1.93143300
C	1.99353200	1.89523400	-0.55127400
H	1.88169200	2.67772000	0.20984800
H	3.04280500	1.62681700	-0.66576100
H	1.64140600	2.32459500	-1.49969300
C	-5.17785400	-0.58631300	-0.17577200
H	-5.84446100	-1.44737700	-0.28948500
H	-5.40210600	-0.11618600	0.78747800
H	-5.43095400	0.13577400	-0.95950700

anti-**I**

C	0.32062500	-0.97788700	0.12568900
H	0.65910100	-1.90054500	-0.36540100
C	-3.33795800	0.60811600	0.22736200
O	-3.93175000	-0.49617700	0.19982500
O	-3.81465800	1.76584700	0.24120100
C	1.31011900	0.14085200	-0.23089800
H	1.03822700	1.04242400	0.32977500
H	1.19999500	0.38918400	-1.29340300
C	2.76995900	-0.22535000	0.05764500
H	3.04693700	-1.10581100	-0.53929000
H	2.85832600	-0.52675700	1.10691900
C	3.75703000	0.90972300	-0.23707600
H	3.48591600	1.78663900	0.36372900
H	3.65490500	1.21908600	-1.28518500

O	0.40906500	-1.27174100	1.53672900
H	-0.28777400	-0.76806800	1.97241900
C	-1.30593800	-0.75476700	-1.82625100
H	-0.87070200	-1.66272500	-2.26191000
H	-2.36996800	-0.73618700	-2.06911200
H	-0.83477900	0.11068000	-2.30578900
C	5.21635300	0.53454400	0.04185100
H	5.89630000	1.36530000	-0.17343600
H	5.35634400	0.25262700	1.09067100
H	5.52861000	-0.31802400	-0.57083300
C	-1.76297800	0.54738600	0.29288100
H	-1.36895000	1.44782300	-0.18899300
H	-1.52064600	0.64653300	1.36291800
C	-1.14170500	-0.72618500	-0.29972500
H	-1.72614400	-1.56420100	0.09600900

gauche-**I**

C	-0.01167700	0.81104700	0.76405000
H	-0.46834200	1.71251700	1.20326300
C	2.53383600	-1.13779500	-0.23433400
O	3.34865700	-1.72880000	-0.96688100
O	2.04407900	-1.55441500	0.85987500
C	-0.98371500	0.28606000	-0.31121700
H	-0.60630600	-0.67457700	-0.67751100
H	-1.00251600	0.97230900	-1.16945500
C	-2.40682700	0.10022600	0.22413400
H	-2.80391300	1.07327100	0.54847700
H	-2.35667600	-0.52673100	1.12036900
C	-3.37247700	-0.52151400	-0.79071700
H	-2.98066900	-1.49605300	-1.10636000
H	-3.40366400	0.09961700	-1.69580500
O	0.10853400	-0.11542600	1.82645600
H	0.83996700	-0.74850100	1.55209000
C	1.27716100	2.63312000	-0.45765000
H	0.84062400	3.37445200	0.22178300
H	2.26815400	2.99211700	-0.75395600
H	0.65918300	2.60601600	-1.36256200
C	-4.79493900	-0.69787900	-0.24793900
H	-5.46053500	-1.14902500	-0.99164100
H	-4.79922100	-1.34190000	0.63757600
H	-5.22779900	0.26492500	0.04549200
C	2.06718100	0.25898300	-0.75674100
H	2.96292200	0.74007900	-1.16417100
H	1.42648000	0.06845200	-1.62727100
C	1.37354300	1.24624900	0.20554300
H	2.00962500	1.35118000	1.09331000

(*Z*)-**II**

C	1.40776700	1.18447800	-0.04580100
C	0.07883900	0.86459600	0.64409500
H	-0.32119100	1.81692500	1.02390400
C	2.37552300	0.30647700	-0.35860300
H	3.25416000	0.69427000	-0.87150900
C	2.44541200	-1.20758500	-0.17954200
O	1.60720300	-1.76800900	0.59610600
O	3.34211400	-1.76698700	-0.84266700
C	-0.94194200	0.31545000	-0.38206500
H	-0.57580700	-0.66017500	-0.71758100
H	-0.96254700	0.97328800	-1.26238600
C	-2.35348700	0.17847600	0.19517700
H	-2.72484200	1.17000300	0.49393500

H	-2.29431600	-0.41714100	1.11191300
C	-3.35366100	-0.46046300	-0.77473000
H	-2.98442800	-1.45101500	-1.06602900
H	-3.39699400	0.13053900	-1.69927400
O	0.19286800	0.02409500	1.77099800
H	0.73300500	-0.76493600	1.44740500
C	1.56375500	2.64439300	-0.41189300
H	1.56964300	3.27515800	0.48691400
H	2.48810100	2.82588500	-0.96587400
H	0.72425600	2.99229600	-1.02939900
C	-4.76510000	-0.59478600	-0.19252500
H	-5.45568200	-1.05890200	-0.90486300
H	-4.75807200	-1.20943800	0.71358700
H	-5.17540300	0.38423200	0.07909000

(*E*)-**V**

C	-1.18977400	0.69032400	-0.01681500
C	0.26436700	0.92679900	-0.07747500
C	-1.69927500	-0.55778400	0.10334000
H	-1.02208000	-1.40537700	0.16421500
C	-3.16681000	-0.96473700	0.06528400
O	-3.90055900	-0.58194100	1.00047200
O	-3.43347100	-1.67498700	-0.93311100
C	1.23787200	-0.26202700	-0.01149800
H	1.04776400	-0.91075300	-0.87472600
H	1.00102800	-0.86772800	0.87000600
C	2.70871400	0.15353100	0.01844900
H	2.88517600	0.80163000	0.88401300
H	2.92861400	0.77073300	-0.85869700
C	3.66631900	-1.04256000	0.06565800
H	3.48692100	-1.68763900	-0.80383500
H	3.44184000	-1.65645000	0.94722800
O	0.72189200	2.06343900	-0.18849500
C	-2.06514500	1.91650700	-0.11751500
H	-1.97289400	2.38485000	-1.10384800
H	-3.10586000	1.64991200	0.06260800
H	-1.75833900	2.67034500	0.61379700
C	5.14253600	-0.63259800	0.09645700
H	5.80379100	-1.50459300	0.12913700
H	5.40683300	-0.04664200	-0.79005500
H	5.36148400	-0.01510800	0.97393000

anti-**IV**

C	-0.29279700	0.89612400	0.39903300
C	3.34165400	-0.68350700	0.16774800
O	3.96223400	0.39781000	0.04449500
O	3.78017500	-1.85507300	0.20399600
C	-1.26836000	-0.19365800	-0.05512000
H	-1.01451700	-1.09764500	0.51315200
H	-1.05650200	-0.44753000	-1.09965200
C	-2.74321300	0.15846300	0.13618700
H	-2.97948300	1.05869800	-0.44383000
H	-2.91388300	0.42976400	1.18267200
C	-3.69020100	-0.97627000	-0.27007800
H	-3.45286300	-1.87348500	0.31522400
H	-3.51135900	-1.24498000	-1.31892100
O	-0.69057800	1.81632200	1.09480000
C	1.30546700	1.08218400	-1.53856400

H	0.86397700	2.05030600	-1.80005400
H	2.37171700	1.10859200	-1.76806100
H	0.83618100	0.30978500	-2.15817500
C	-5.16957700	-0.62407800	-0.08219800
H	-5.82229700	-1.45183300	-0.37743400
H	-5.38730600	-0.38435800	0.96375800
H	-5.44631000	0.24887400	-0.68270700
C	1.77405800	-0.58831900	0.32780300
H	1.33490200	-1.39246200	-0.27222400
H	1.56020900	-0.82892100	1.37705800
C	1.15884900	0.77812700	-0.03315100
H	1.70759900	1.54832700	0.51290100

gauche-**IV**

C	0.14773200	0.97228100	0.51025500
C	2.22909900	-1.41300100	0.13468600
O	2.99370800	-2.30020400	-0.30244500
O	1.46726400	-1.44120800	1.13598200
C	-0.81038200	0.10274300	-0.29189400
H	-0.42769800	-0.91909800	-0.21883900
H	-0.72928000	0.39006500	-1.35022900
C	-2.26156700	0.17443900	0.17866600
H	-2.61706300	1.21096300	0.13451000
H	-2.30182500	-0.10866000	1.23540600
C	-3.19680200	-0.72777600	-0.63415900
H	-2.83454100	-1.76141900	-0.58325100
H	-3.15157400	-0.44298800	-1.69371200
O	-0.24488600	1.63369600	1.45596700
C	1.65753900	2.39559300	-0.85475400
H	1.27999800	3.26751400	-0.31234200
H	2.69194000	2.59529600	-1.15147500
H	1.07022300	2.28156400	-1.77343500
C	-4.65244900	-0.67430400	-0.15791800
H	-5.29791800	-1.32784700	-0.75422600
H	-4.73448000	-0.98910000	0.88756500
H	-5.05342400	0.34293000	-0.22608300
C	2.18908500	-0.08986900	-0.70255500
H	3.21244200	0.14426000	-1.01494300
H	1.64091400	-0.30280000	-1.63024800
C	1.59033500	1.12201700	0.01873600
H	2.16805300	1.30831800	0.92889600

(*Z*)-**V**

C	1.87656800	0.29363000	0.25213900
C	0.93931300	1.25114800	-0.37987600
C	1.67932400	-1.04148700	0.35752700
H	2.47489200	-1.60295000	0.85029500
C	0.54576600	-1.96692800	-0.07154000
O	-0.32490100	-2.16559400	0.80569900
O	0.66430100	-2.46488800	-1.21197500
C	-0.40998100	0.80505400	-0.91974900
H	-0.34652300	-0.19122900	-1.36004200
H	-0.67511200	1.51638800	-1.70875700
C	-1.49495600	0.82349500	0.17579600
H	-1.57784600	1.84095000	0.58045200
H	-1.18961400	0.15801100	0.98780300
C	-2.85842400	0.36007800	-0.34678700
H	-2.75172600	-0.65745500	-0.73667200
H	-3.16614600	0.99366000	-1.19000900

O	1.24358500	2.44399500	-0.44617000
C	3.14199600	0.92848300	0.79431700
H	2.91461600	1.68836900	1.54939500
H	3.78902800	0.17124800	1.24409700
H	3.69949100	1.44171400	0.00395400
C	-3.94637600	0.37798900	0.73181100
H	-4.91068300	0.04003400	0.33692700
H	-3.67601500	-0.28176900	1.56172900
H	-4.08762800	1.38615300	1.13856800

Copies of Chiral GC chromatograms
1a and 3a

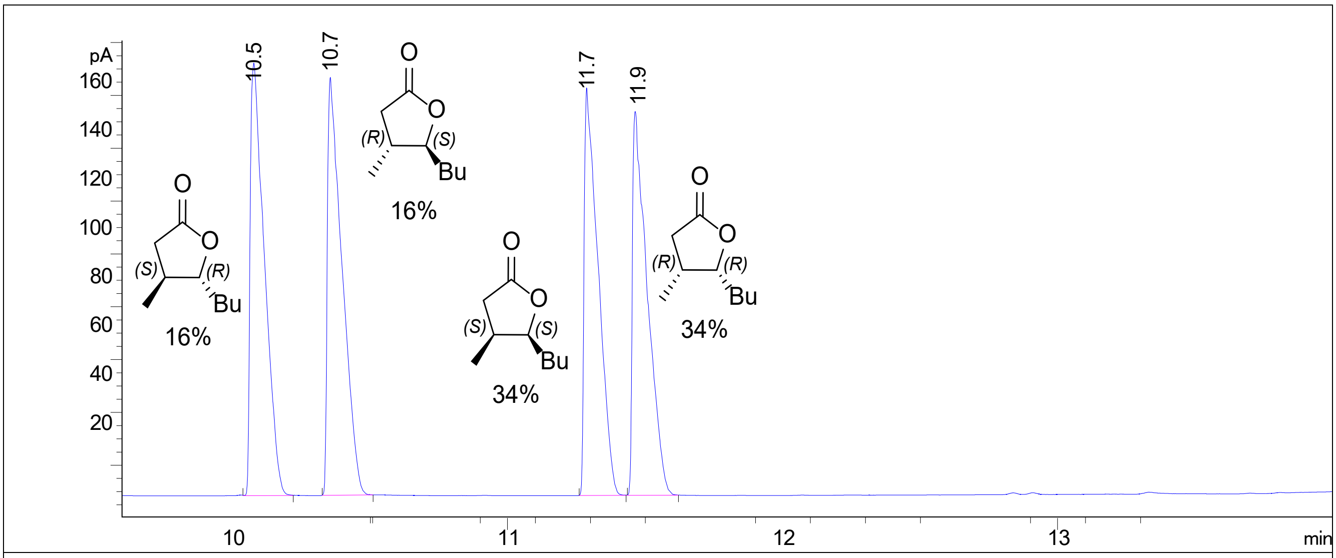

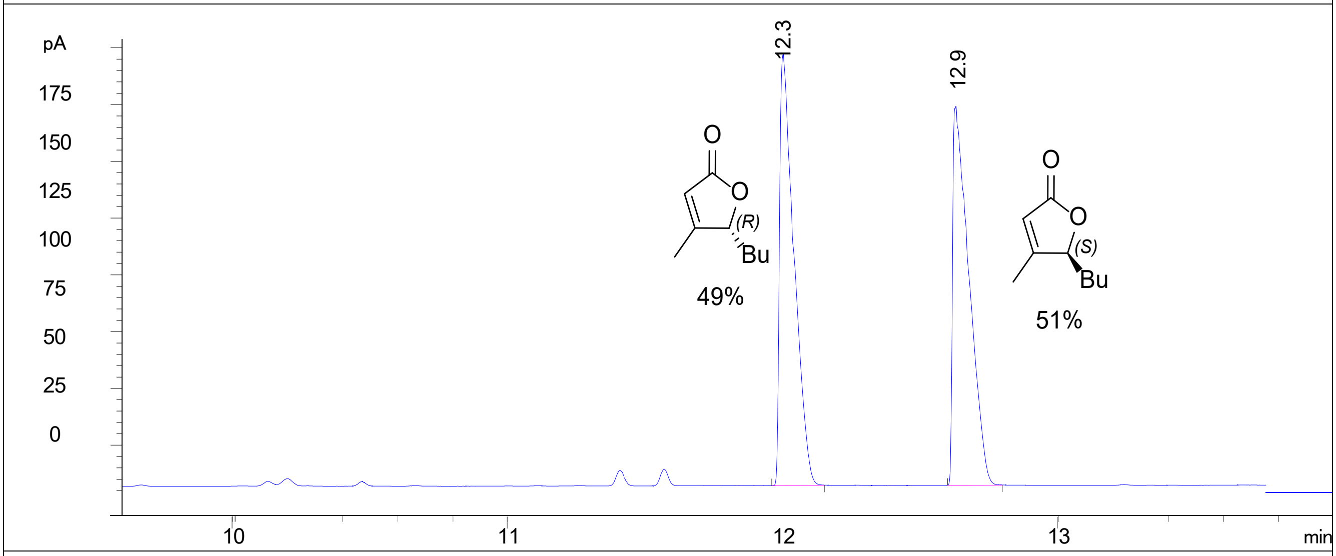

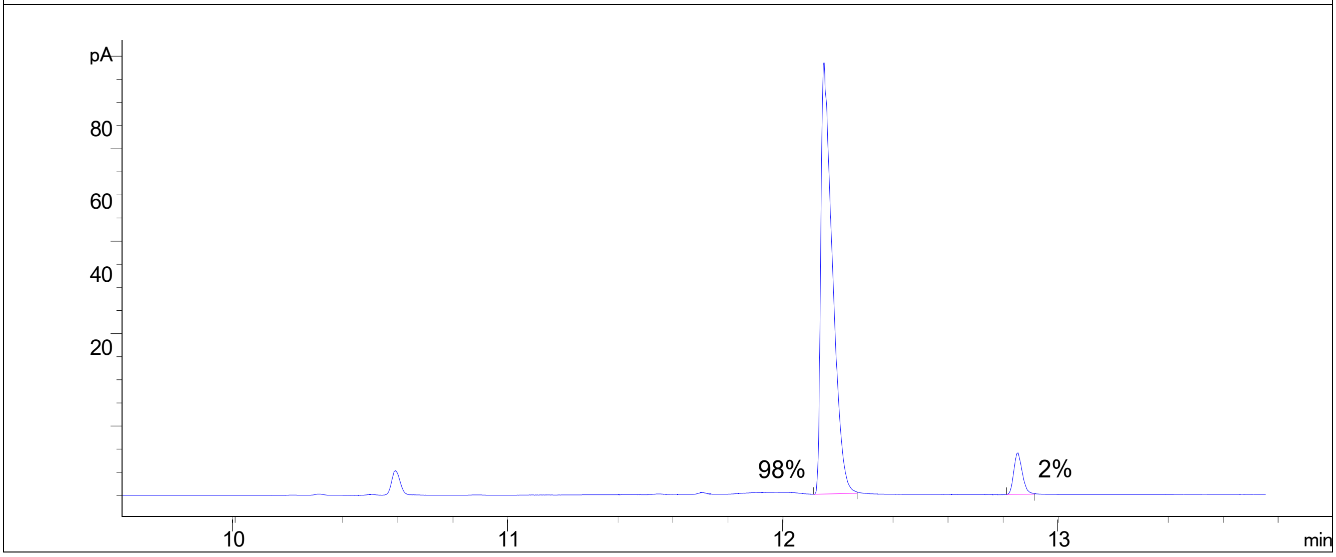

1b and 3b

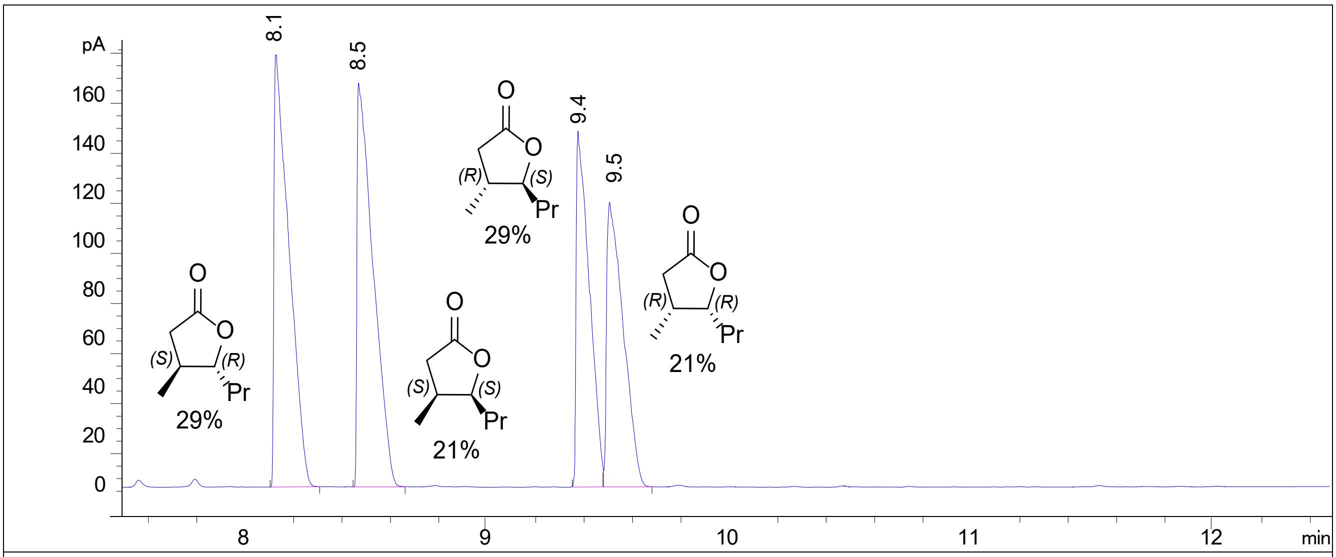
pA
160
140
120
100
80
60
40
20
0
8.1
8.5
9.4
9.5
(S)
(R)
Pr
29%
(S)
(S)
Pr
21%
(R)
(S)
Pr
29%
(R)
(R)
Pr
21%
8
9
10
11
12
min

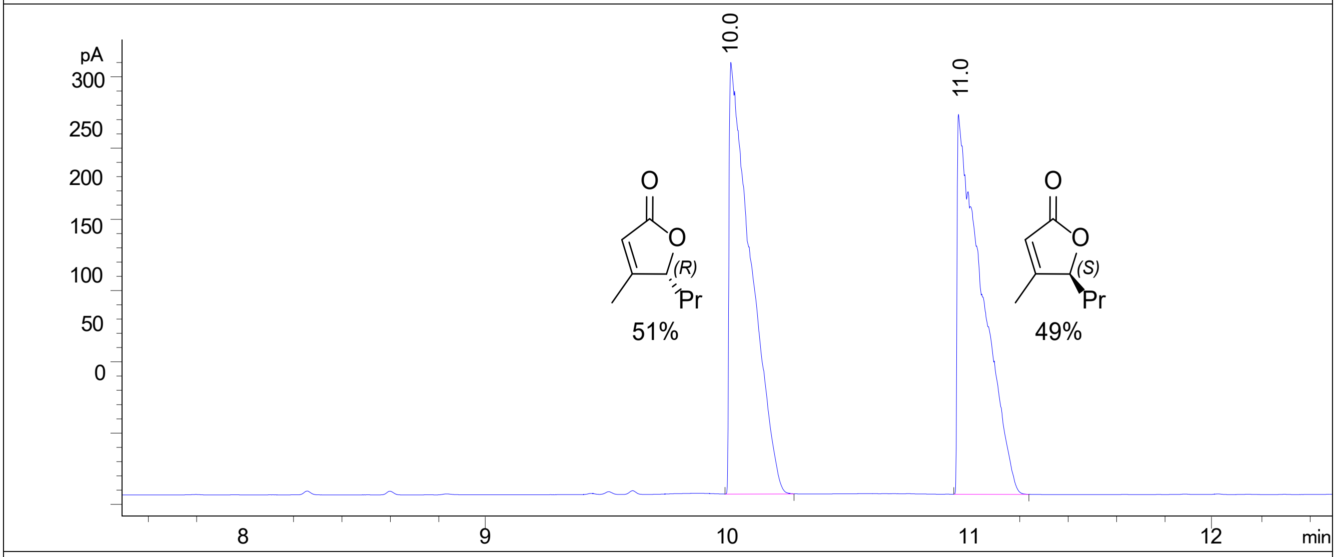
pA
300
250
200
150
100
50
0
10.0
11.0
(R)
Pr
51%
(S)
Pr
49%
8
9
10
11
12
min

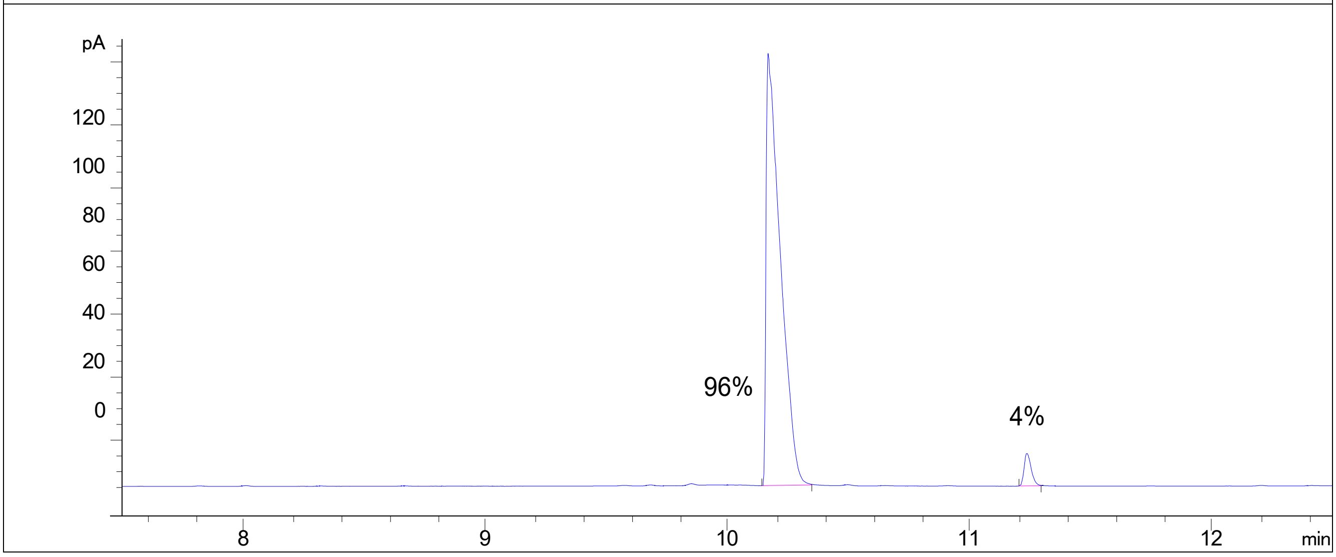
pA
120
100
80
60
40
20
0
96%
4%
8
9
10
11
12
min

1d and 3d

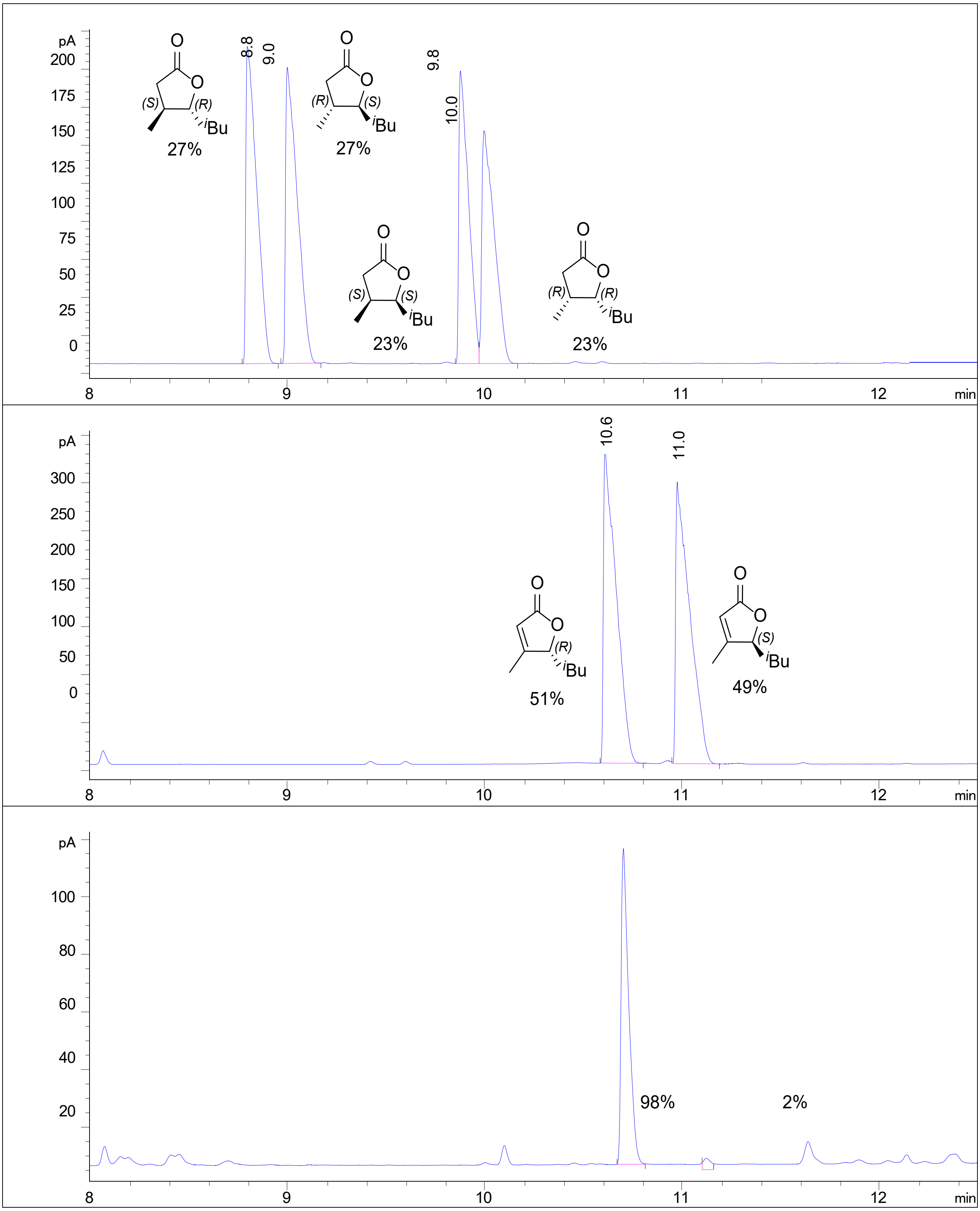
pA
200
175
150
125
100
75
50
25
0
8.8
9.0
9.8
10.0
(S)
(R)
iBu
27%
(R)
(S)
iBu
27%
(S)
(S)
iBu
23%
(R)
(R)
iBu
23%
8
9
10
11
12
min
pA
300
250
200
150
100
50
0
10.6
11.0
(R)
iBu
51%
(S)
iBu
49%
8
9
10
11
12
min
pA
100
80
60
40
20
98%
2%
8
9
10
11
12
min

1e and 3e

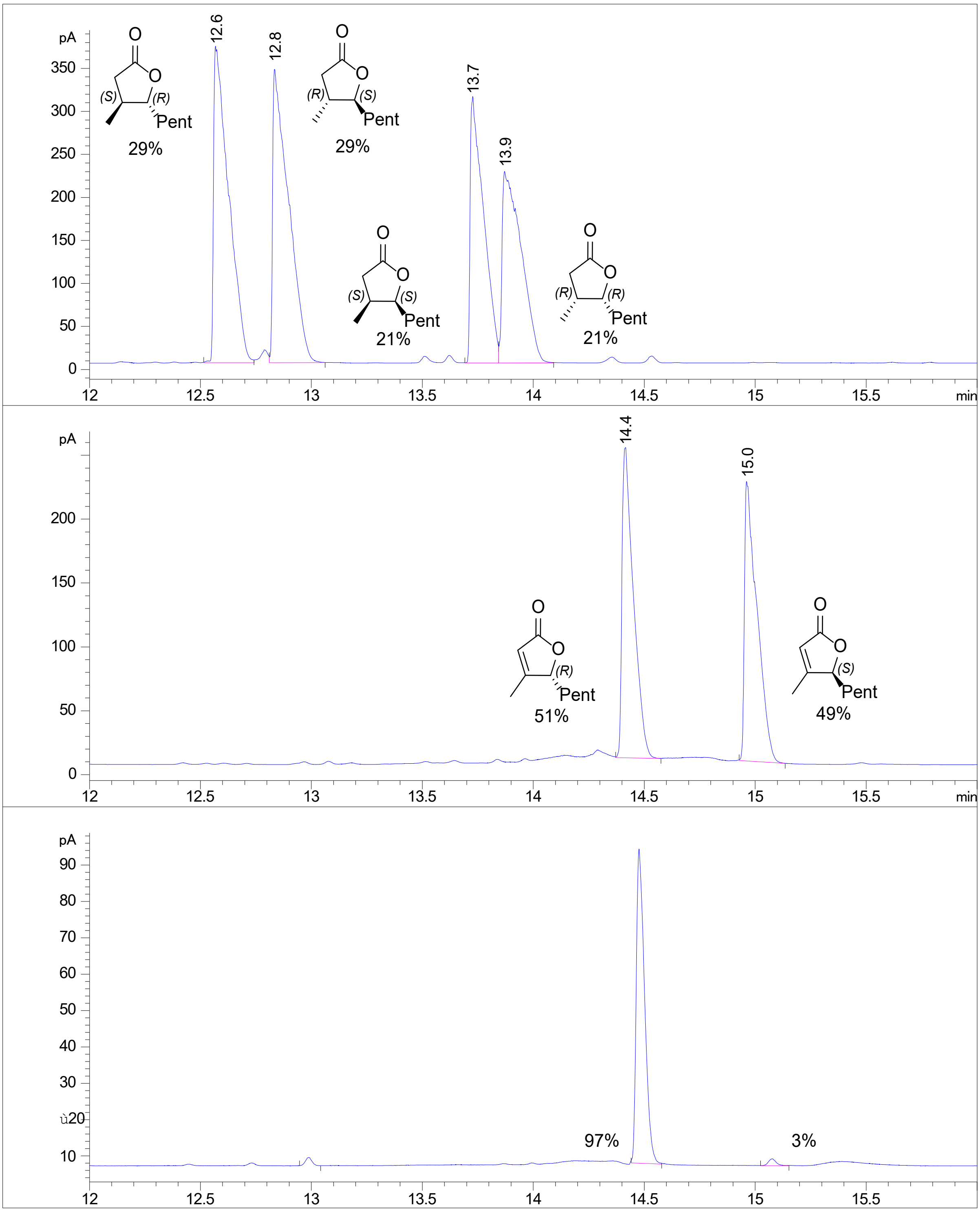
pA
12.6
12.8
13.7
13.9
(S)
(R)
Pent
29%
(R)
(S)
Pent
29%
(S)
(S)
Pent
21%
(R)
(R)
Pent
21%
min
14.4
15.0
(R)
Pent
51%
(S)
Pent
49%
97%
3%

1f and 3f

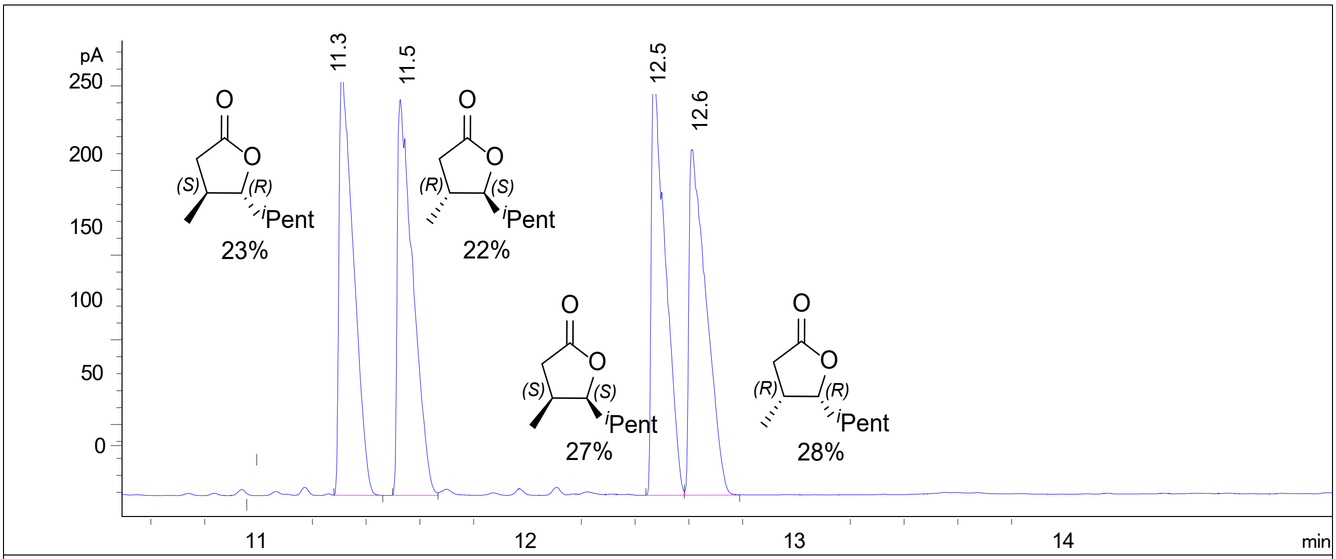
pA
250
200
150
100
50
0
11.3
11.5
12.5
12.6
(S)
(R)
^{i}Pent
23%
(R)
(S)
^{i}Pent
22%
(S)
(S)
^{i}Pent
27%
(R)
(R)
^{i}Pent
28%
11
12
13
14
min

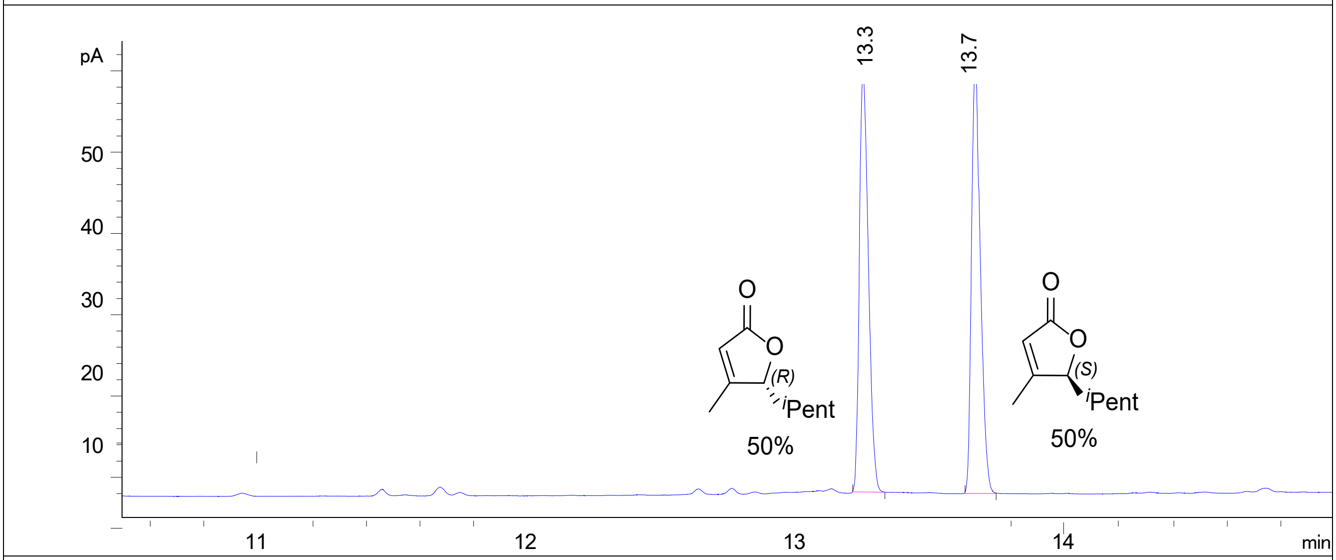
pA
50
40
30
20
10
13.3
13.7
(R)
^{i}Pent
50%
(S)
^{i}Pent
50%
11
12
13
14
min

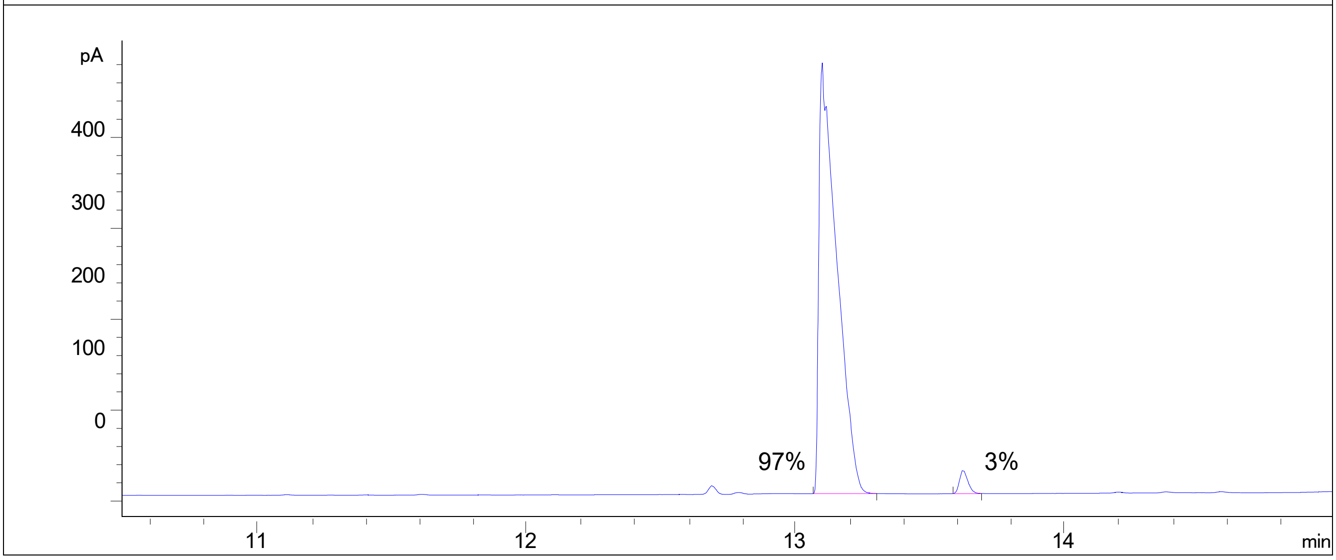
pA
400
300
200
100
0
97%
3%
11
12
13
14
min

1g and 3g

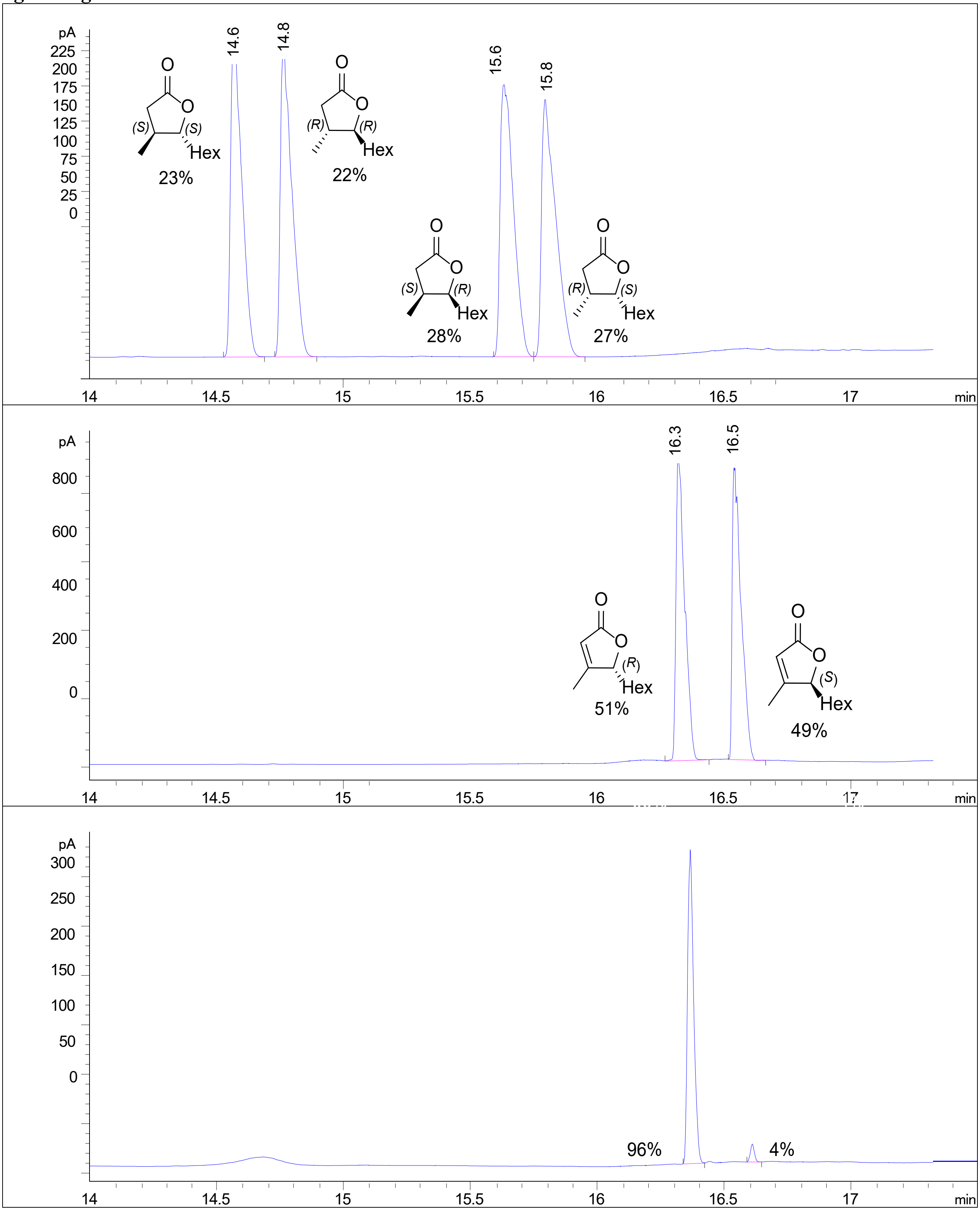

pA
225
200
175
150
125
100
75
50
25
0
14.6
14.8
15.6
15.8
(S)
(S)
Hex
23%
(R)
(R)
Hex
22%
(S)
(R)
Hex
28%
(R)
(S)
Hex
27%
14
14.5
15
15.5
16
16.5
17
min
pA
800
600
400
200
0
16.3
16.5
(R)
Hex
51%
(S)
Hex
49%
pA
300
250
200
150
100
50
0
96%
4%

11 and 31

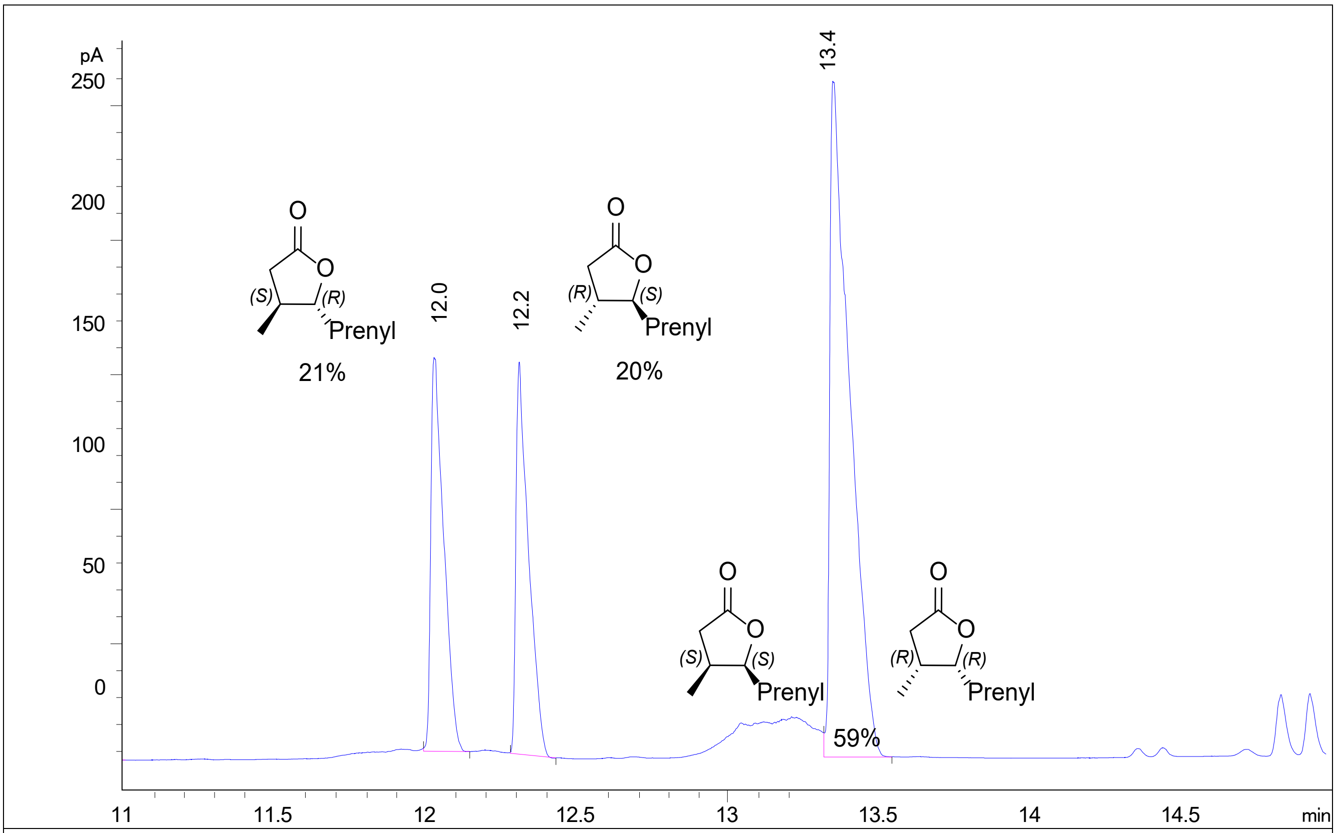
pA
250
200
150
100
50
0
12.0
12.2
13.4
(S)
(R)
Prenyl
21%
(R)
(S)
Prenyl
20%
(S)
(S)
Prenyl
(R)
(R)
Prenyl
59%
11
11.5
12
12.5
13
13.5
14
14.5
min

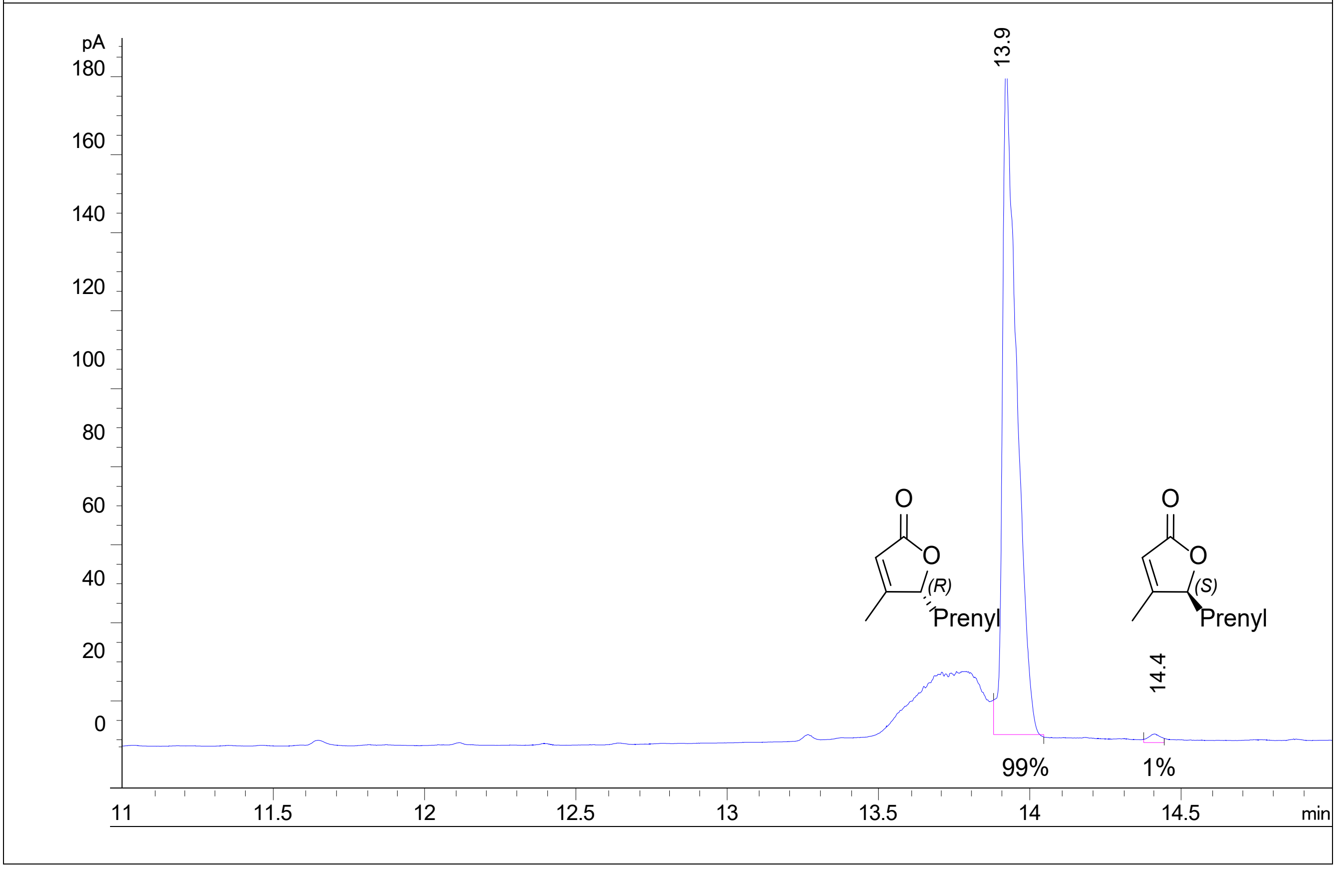
pA
180
160
140
120
100
80
60
40
20
0
13.9
14.4
(R)
Prenyl
(S)
Prenyl
99%
1%
11
11.5
12
12.5
13
13.5
14
14.5
min

1q and 3q

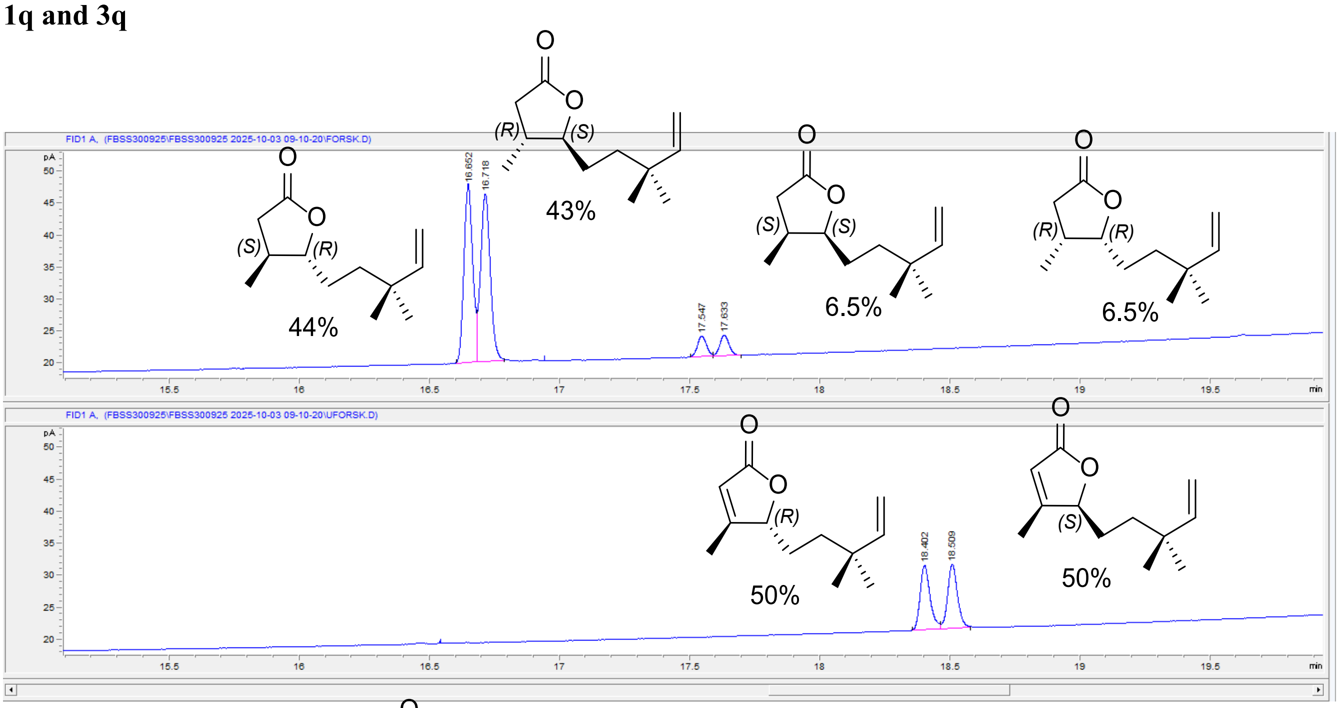
FID1 A, (FBSS300925\FBSS300925 2025-10-03 09-10-20\FORSK.D)
(S)
(R)
44%
(R)
(S)
43%
16.652
16.718
17.547
17.633
(S)
(S)
6.5%
(R)
(R)
6.5%
FID1 A, (FBSS300925\FBSS300925 2025-10-03 09-10-20\UFORSK.D)
(R)
50%
18.402
18.509
(S)
50%
pA
min

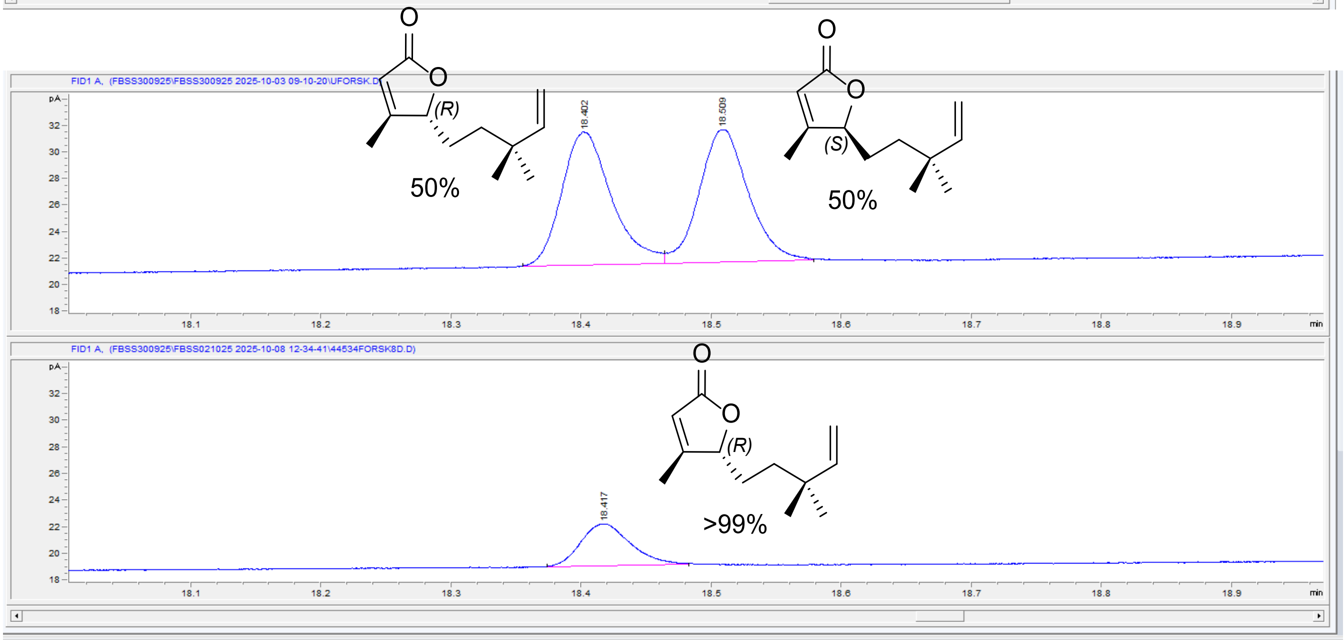
FID1 A, (FBSS300925\FBSS300925 2025-10-03 09-10-20\UFORSK.D)
(R)
50%
18.402
18.509
(S)
50%
FID1 A, (FBSS300925\FBSS021025 2025-10-08 12-34-41\44534FORSK8D.D)
(R)
18.417
>99%
pA
min

(+)-Whisky lactone from hydrogenation of 3a (*cis*-1a + *trans*-1a)

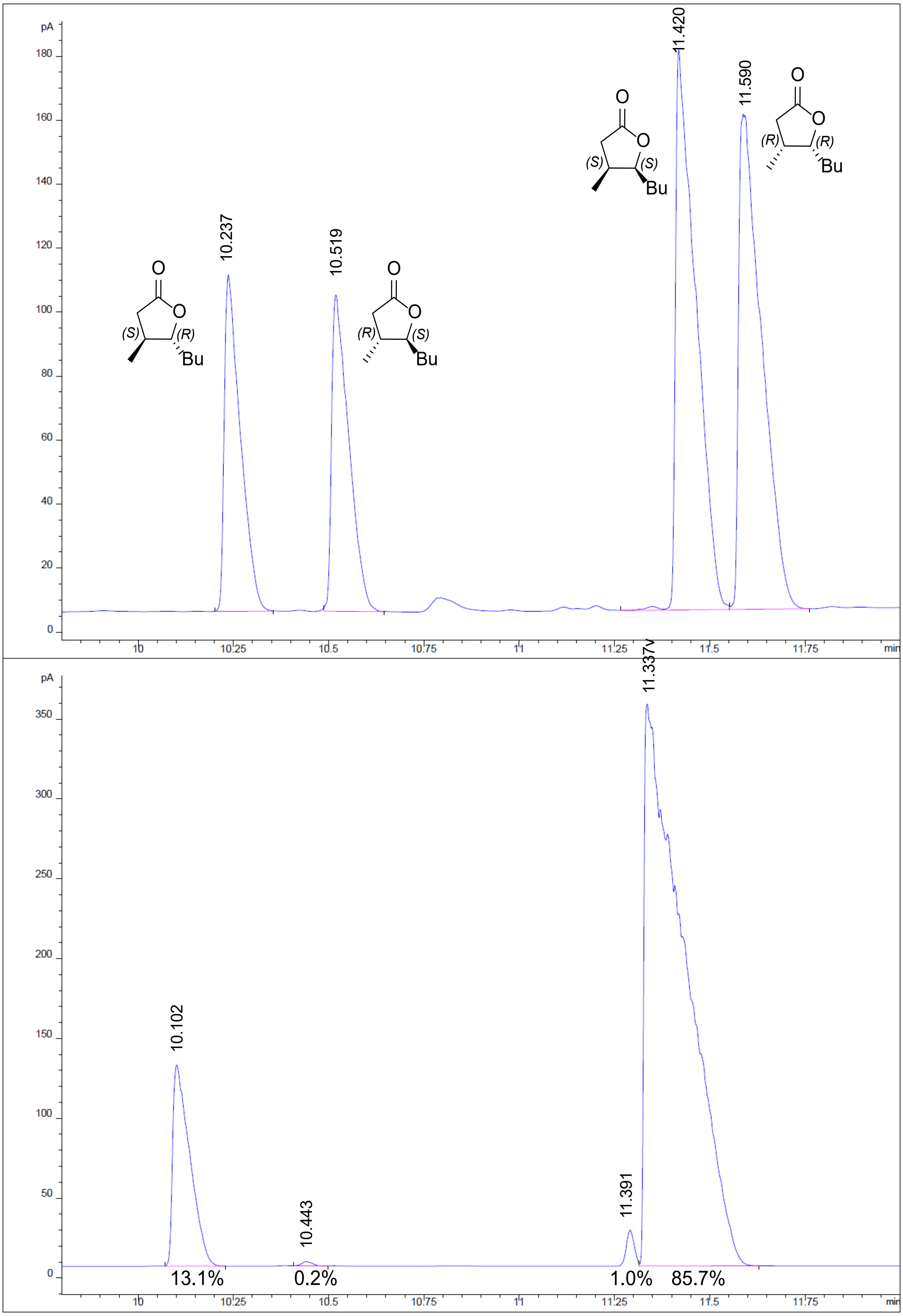

(+)-Cognac lactone from hydrogenation of 3a (*cis*-1e + *trans*-1e)

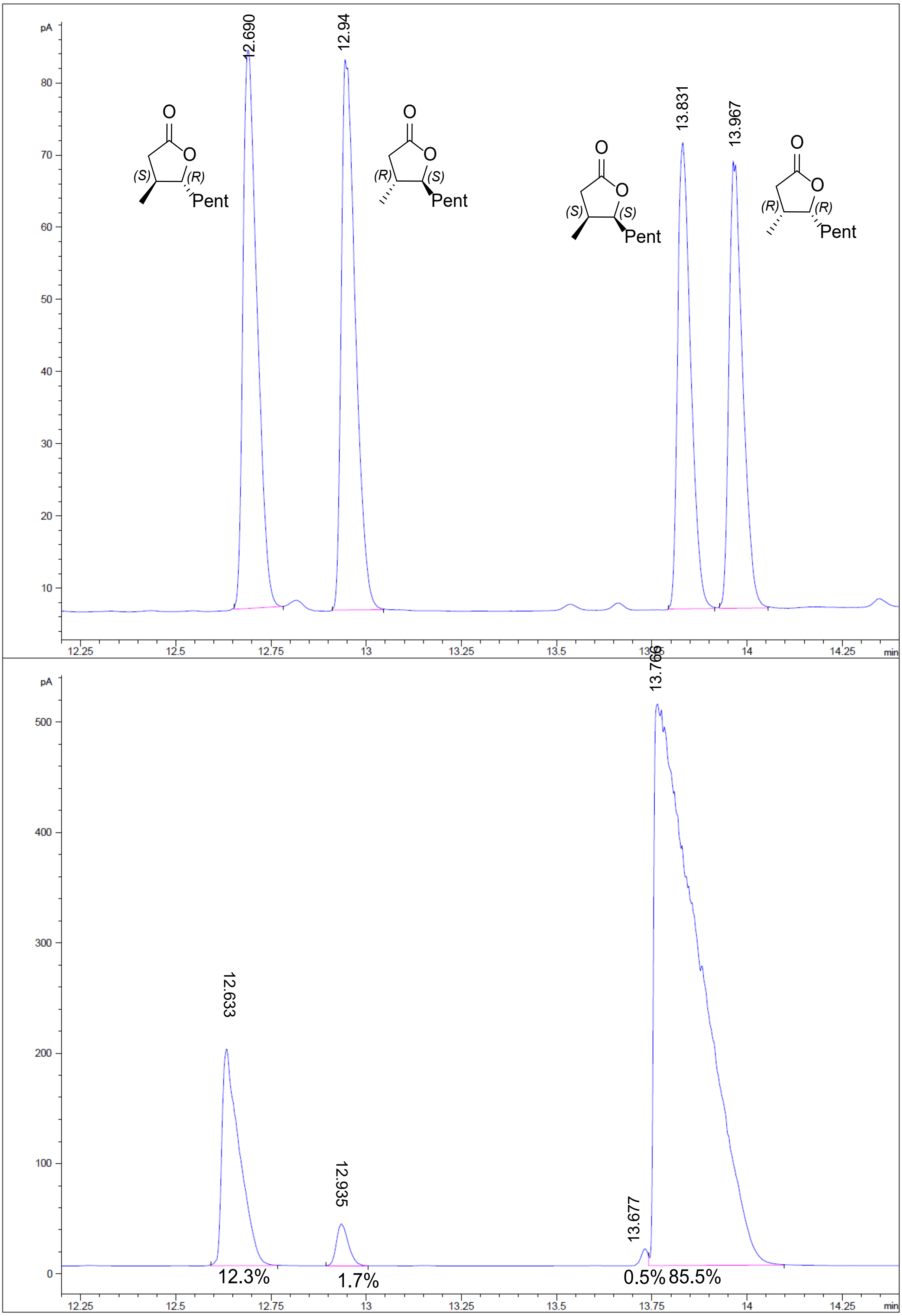

(+)-*trans*-1a-3,4-d_2 + (+)-*cis*-1a-3,4-d_2 from (*R*)-3a by H-cube reduction

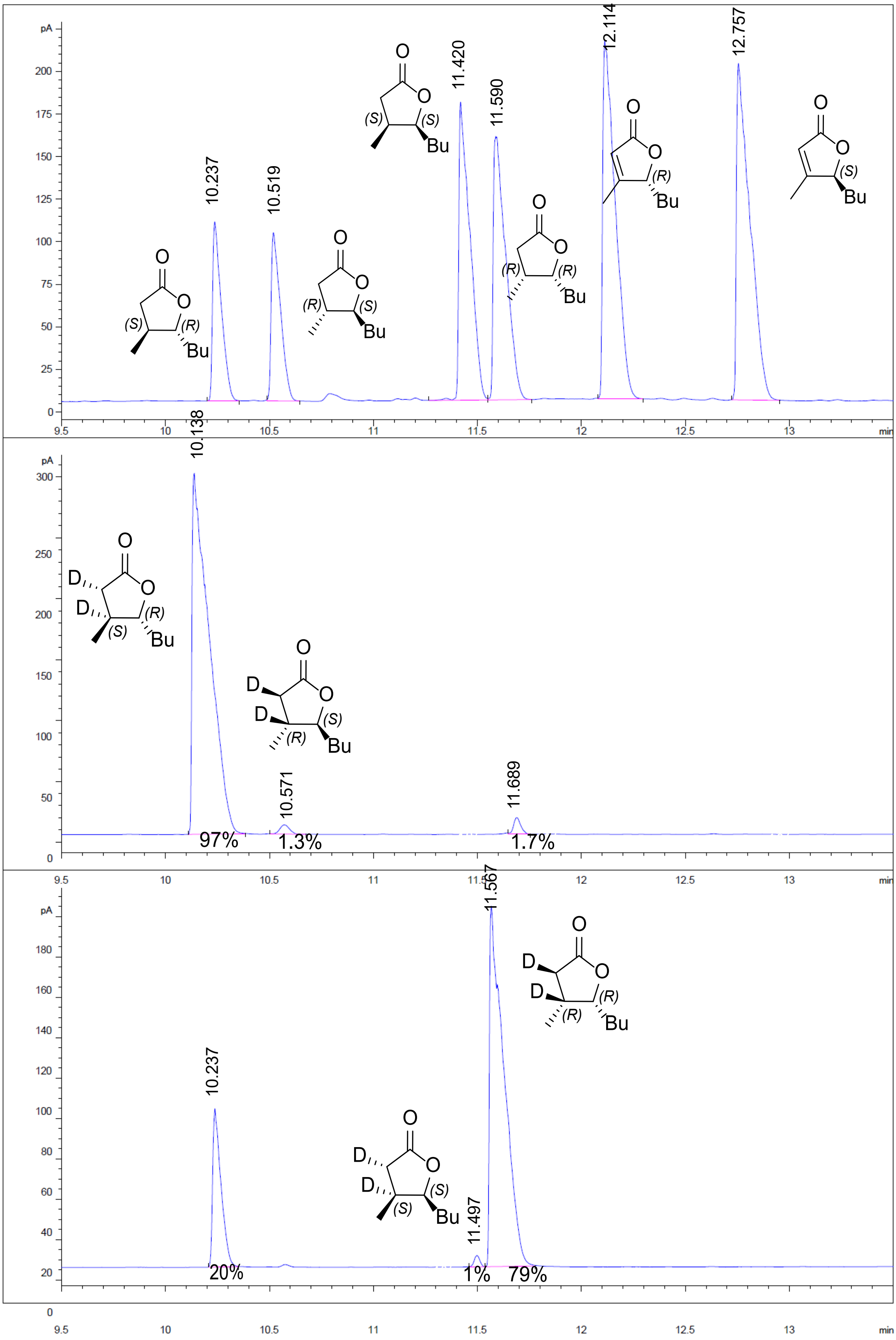

Exp. A: (*R*)-3a from 1a-5*d*

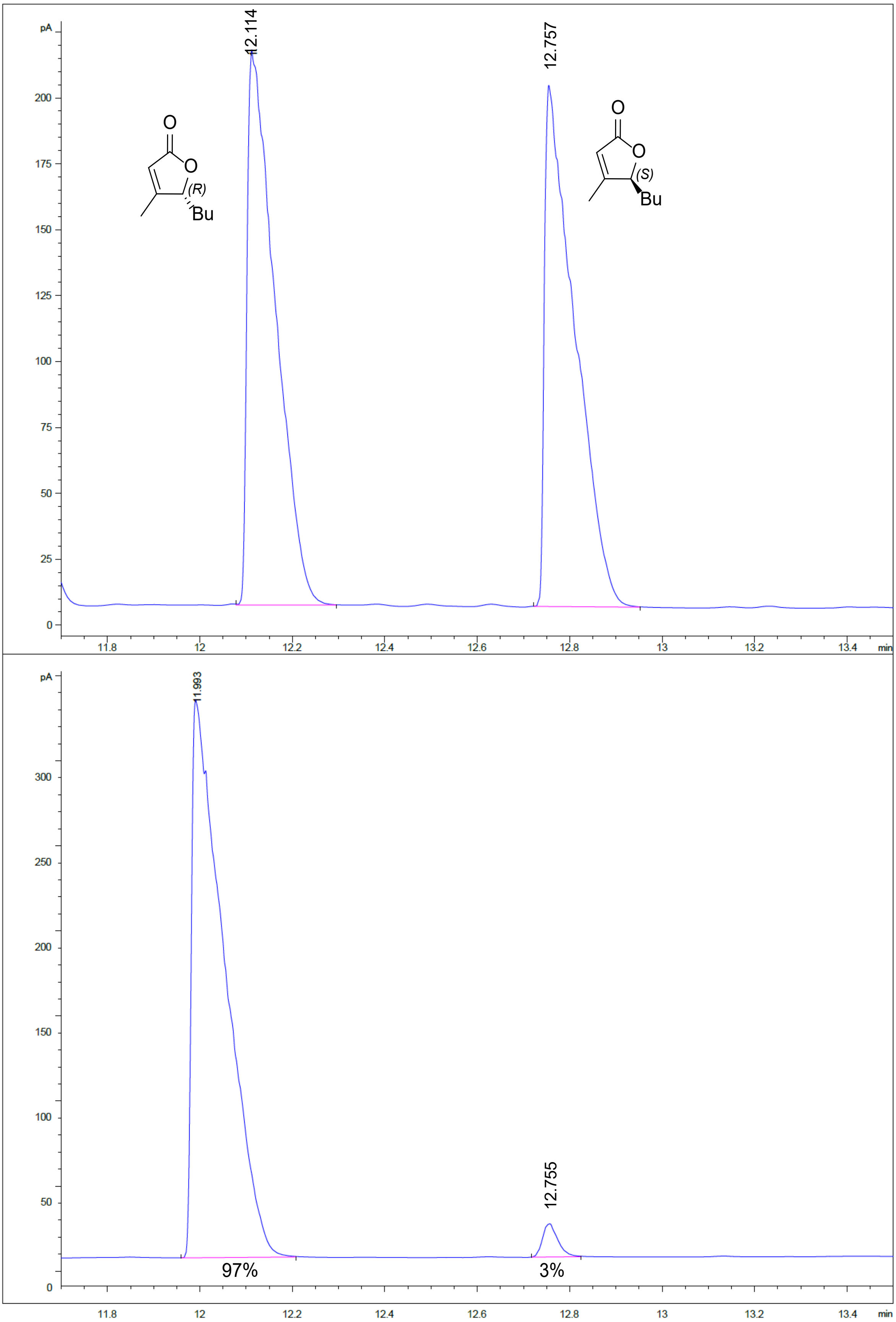

Exp. B: (*R*)-3a from 2a-1,1,4-d_3

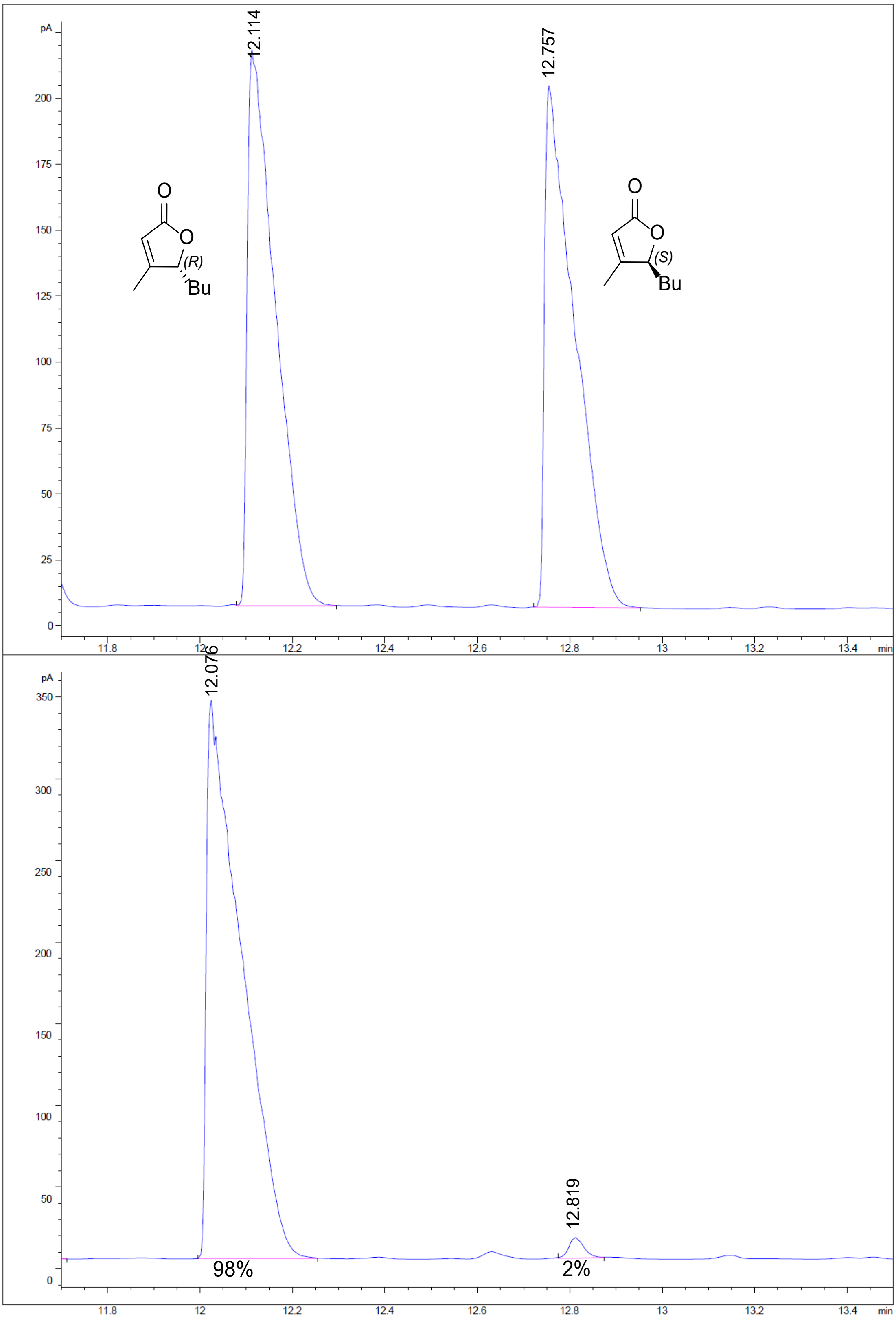

Exp. C+D: (*R*)-3a from 1a in different H_2O/D_2O ratio

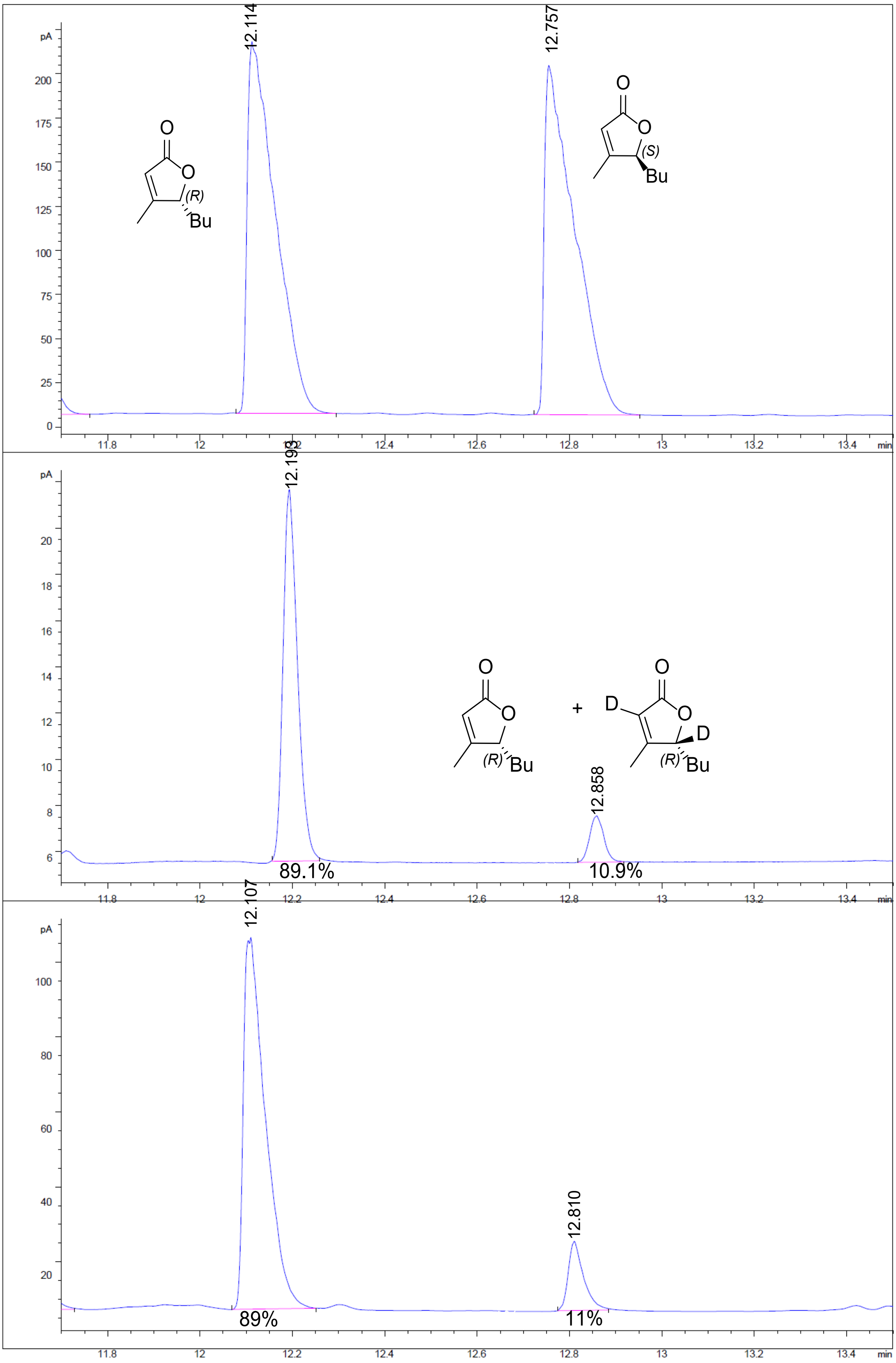

Exp. E: (*R*)-3a from *cis*-1a-3,4-d_2

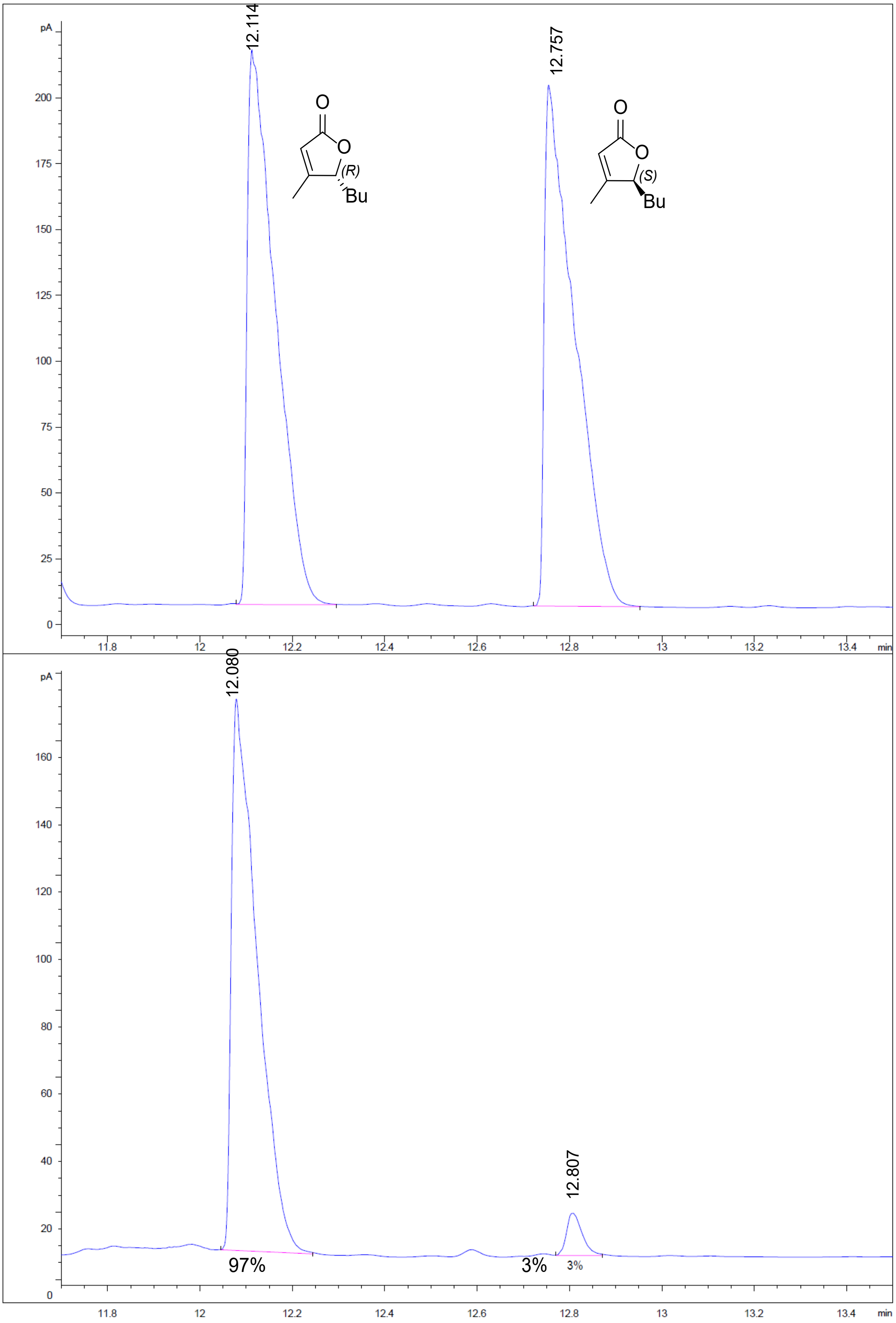

Chiral GC chromatograms of biotransformation of (5*S*)-1a enantiomerically enriched stereoisomers

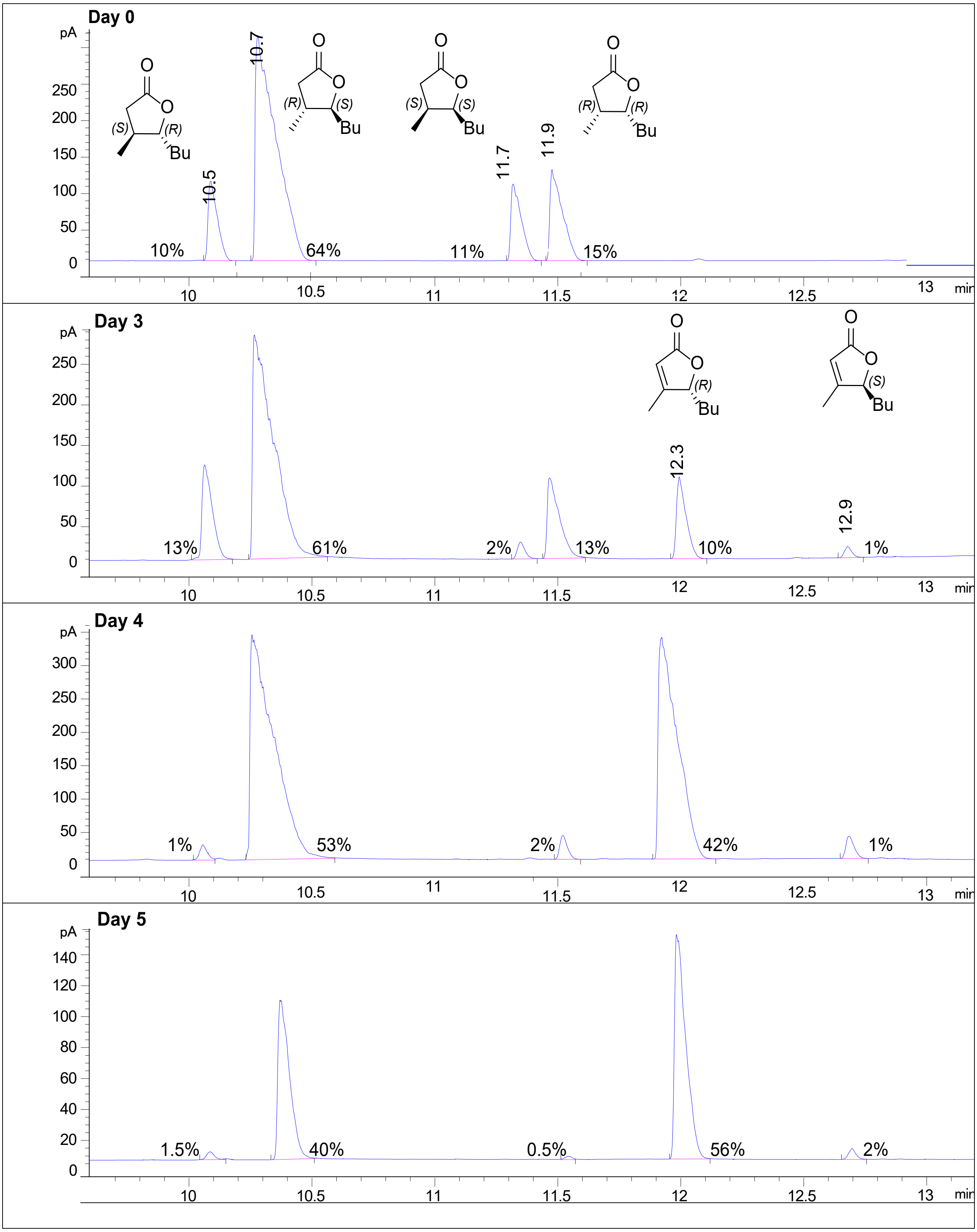

Copies of ^{1}H and ^{13}C NMR spectra

^{1}H-NMR (CDCl$_3$, 400 MHz)

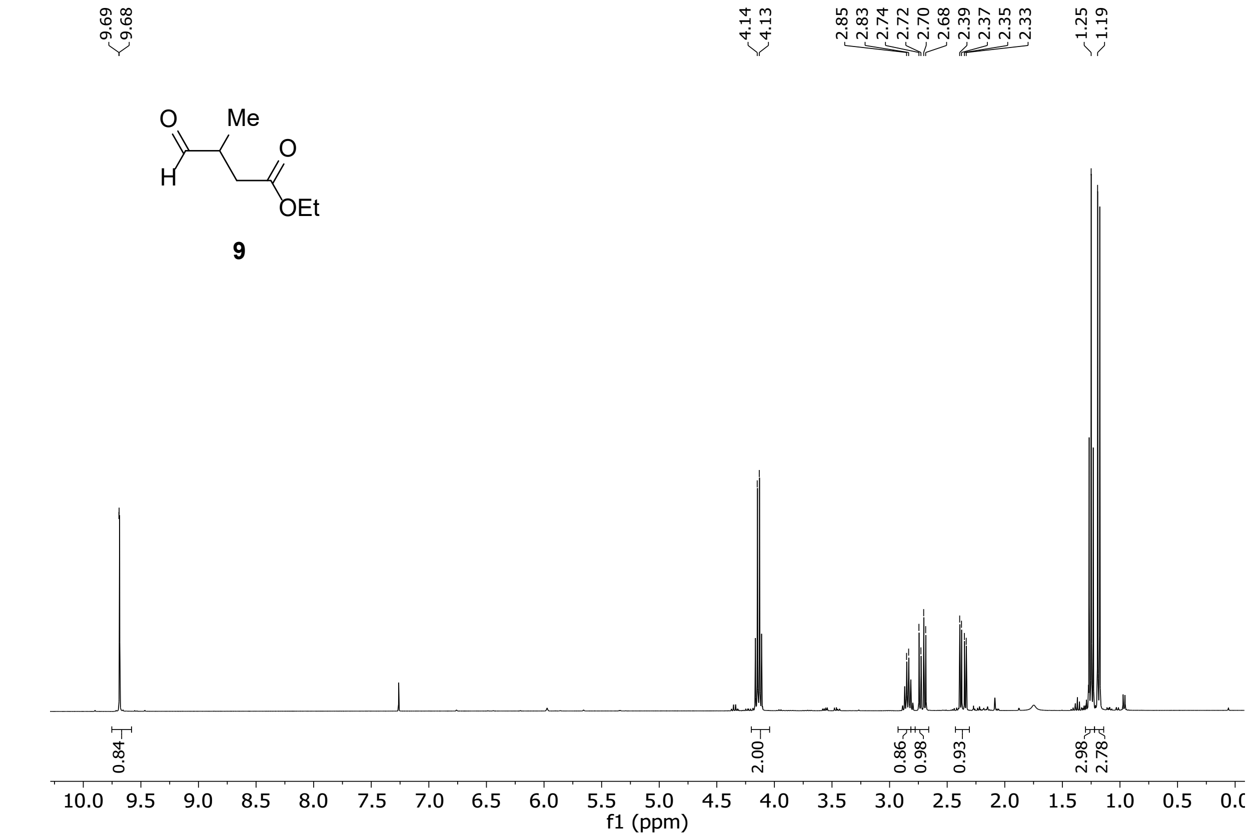

^{13}C{^{1}H}-NMR (CDCl$_3$, 101 MHz)

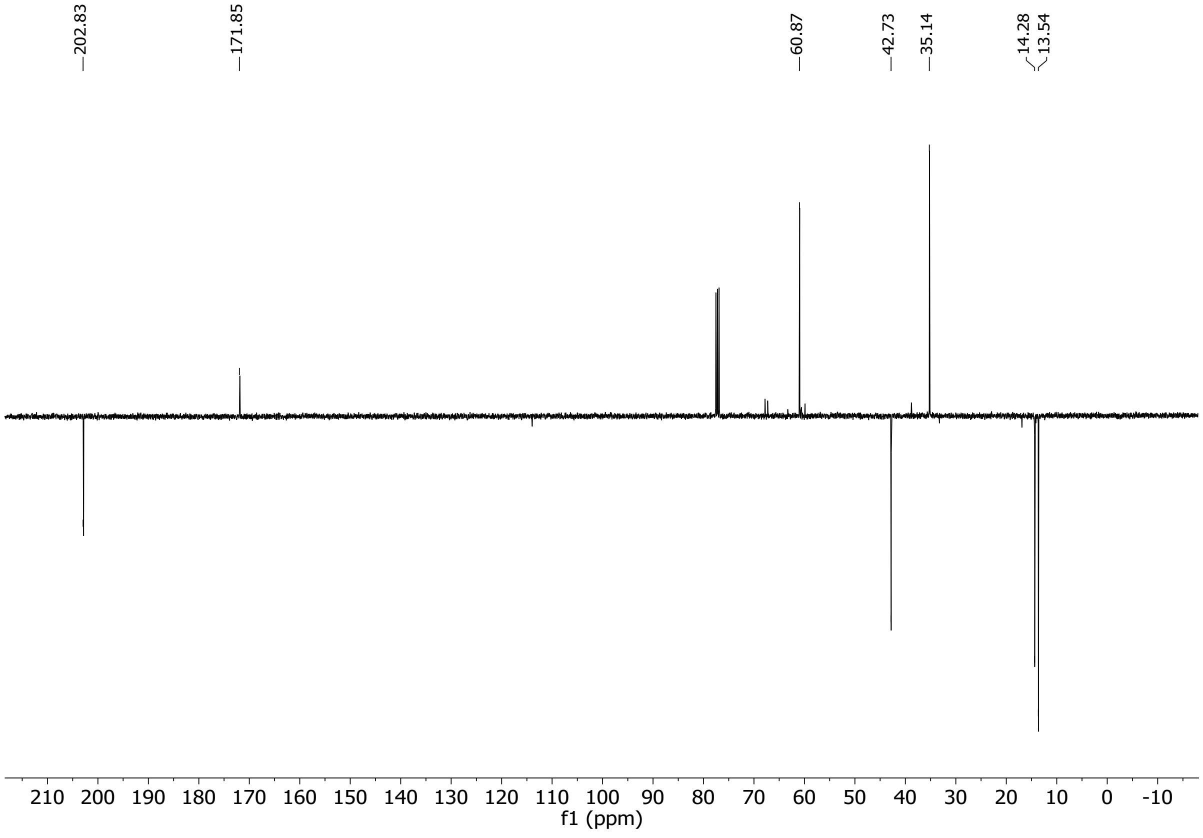

^{1}H-NMR ($CDCl_3$, 400 MHz)

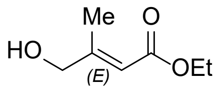

5l

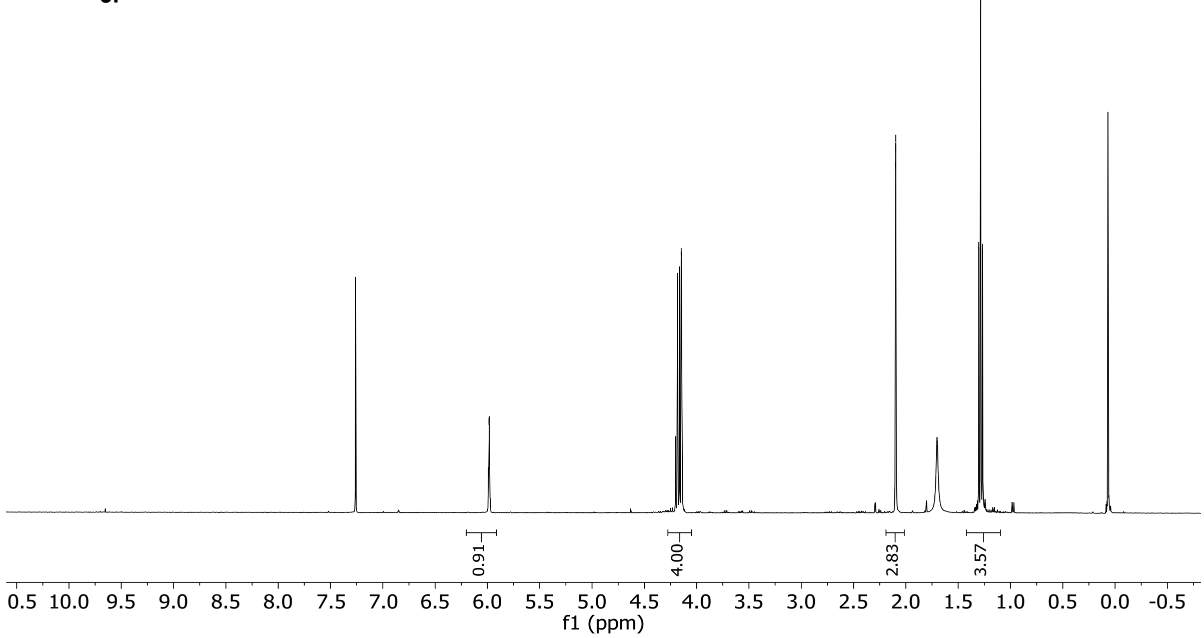

^{13}C{^{1}H}-NMR ($CDCl_3$, 101 MHz)

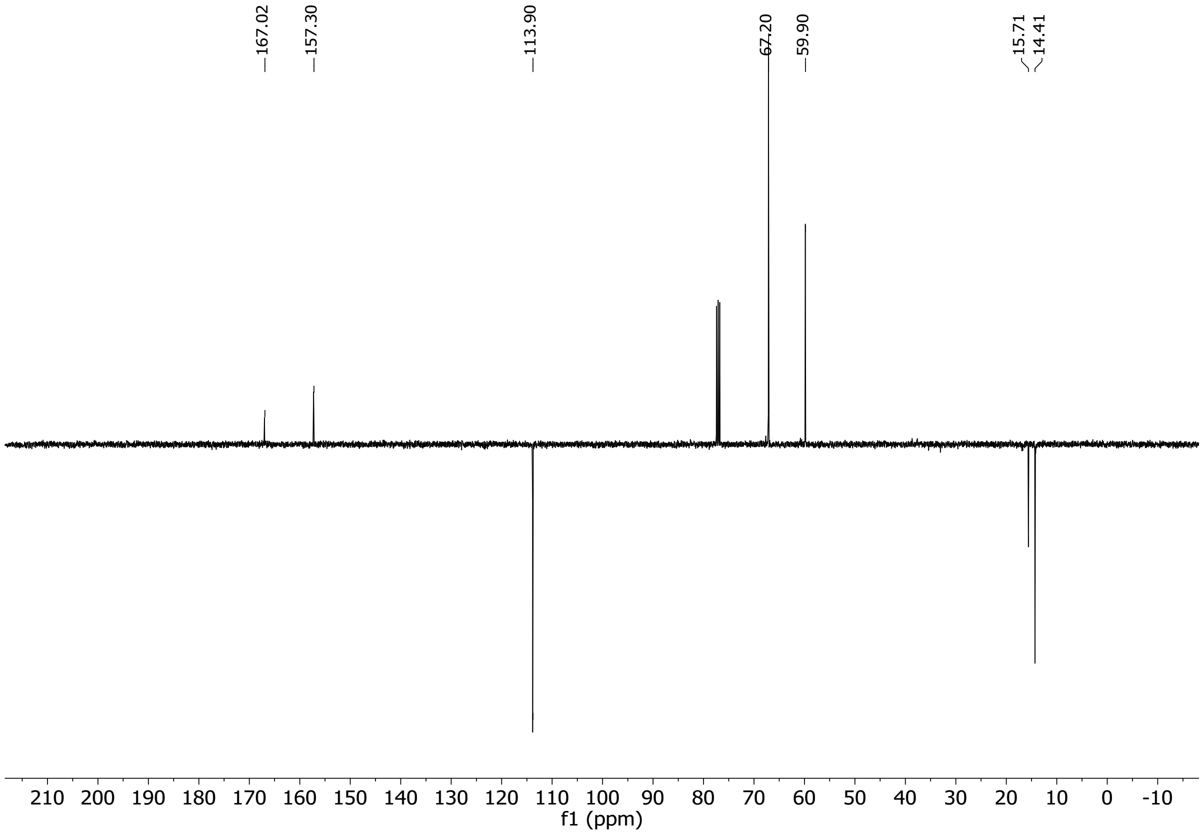

^{1}H-NMR ($CDCl_3$, 400 MHz)

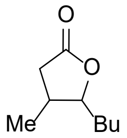

1a

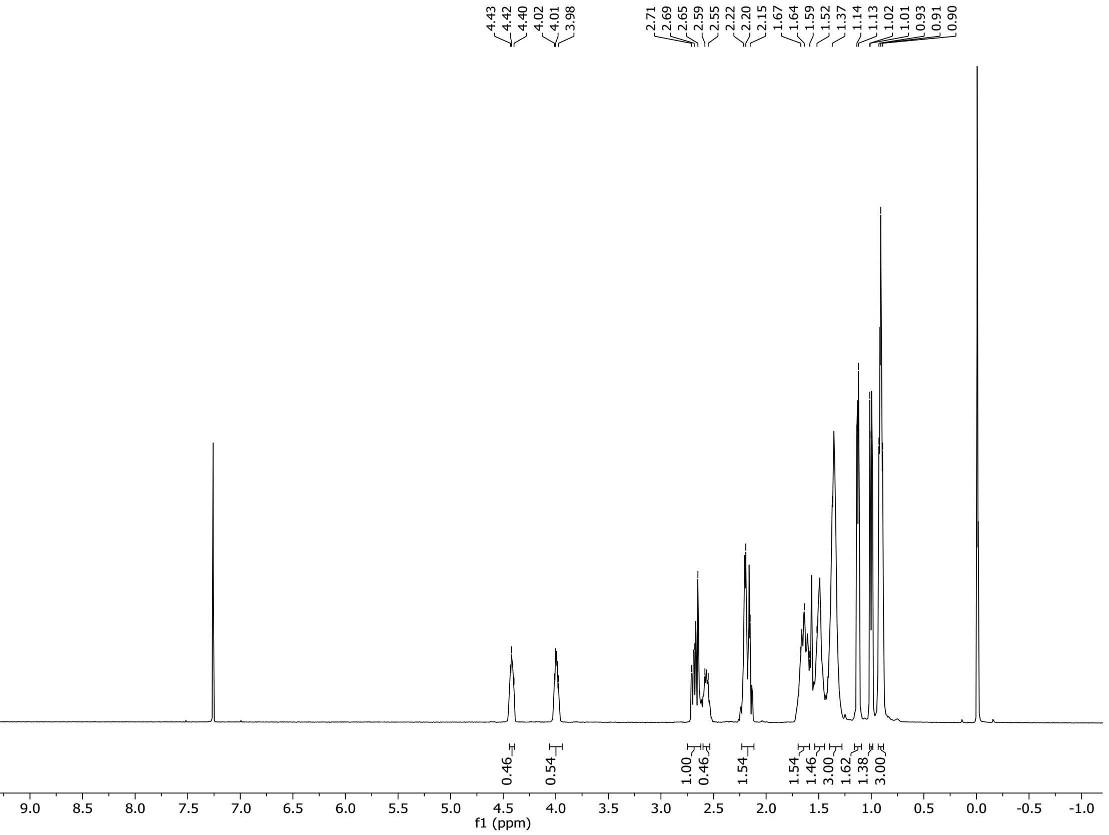

^{13}C{^{1}H}-NMR ($CDCl_3$, 101 MHz)

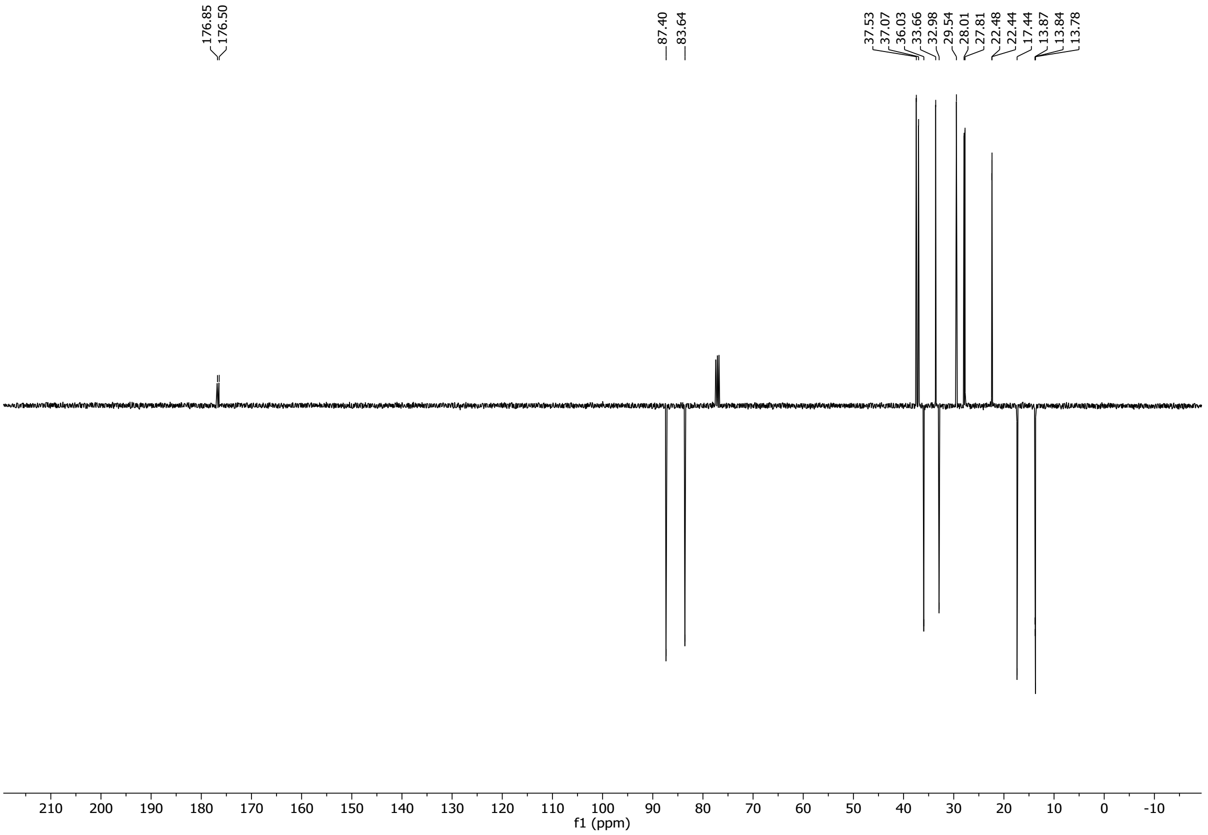

^{1}H-NMR (CDCl$_3$, 400 MHz)

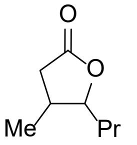

1b

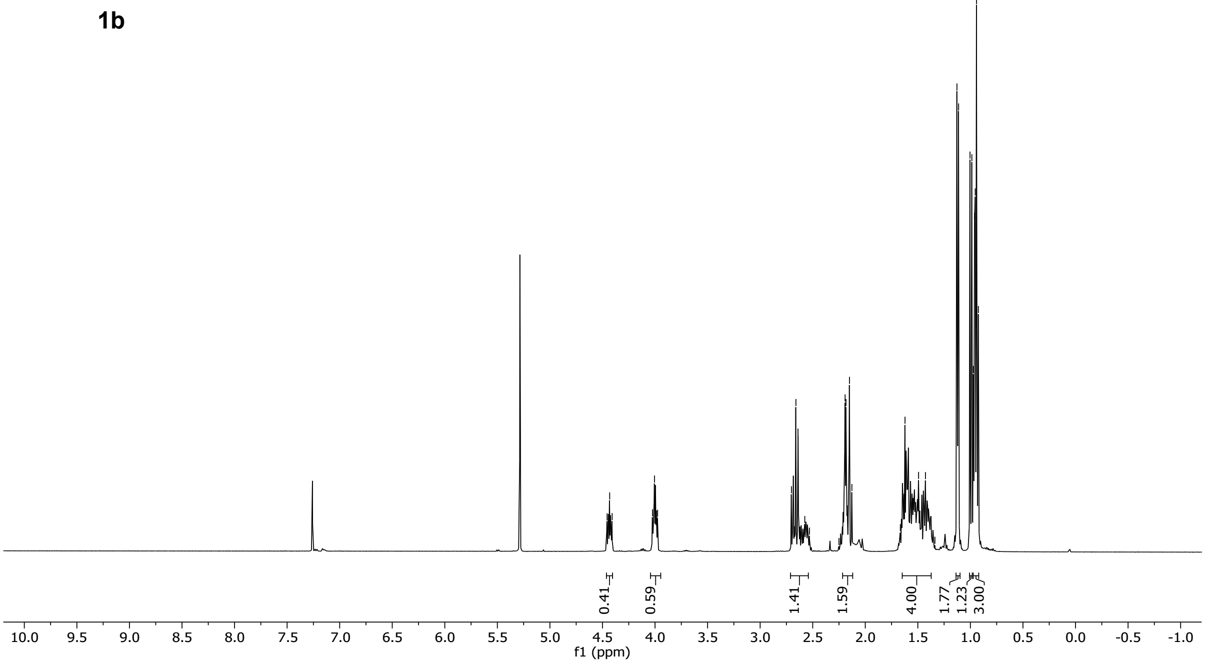

^{13}C{^{1}H}-NMR (CDCl$_3$, 101 MHz)

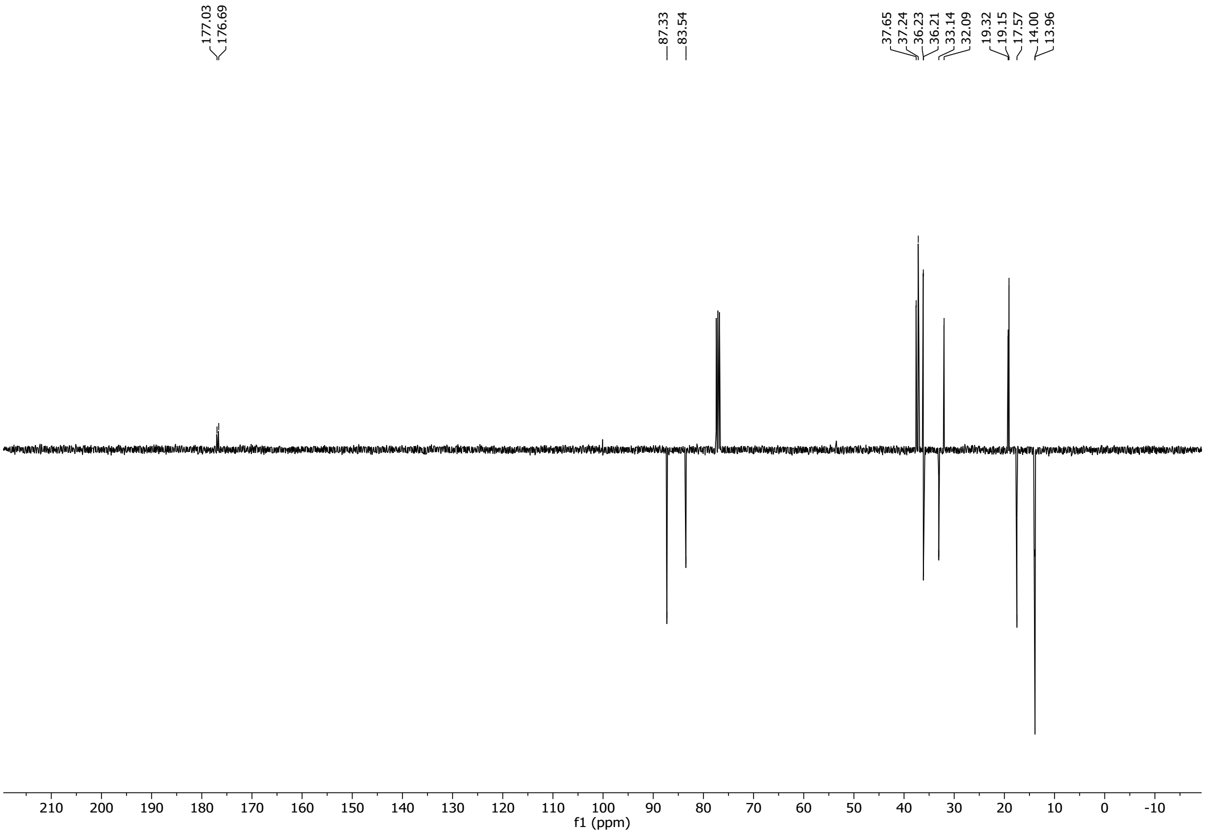

^{1}H-NMR (CDCl$_3$, 400 MHz)

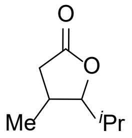

1c

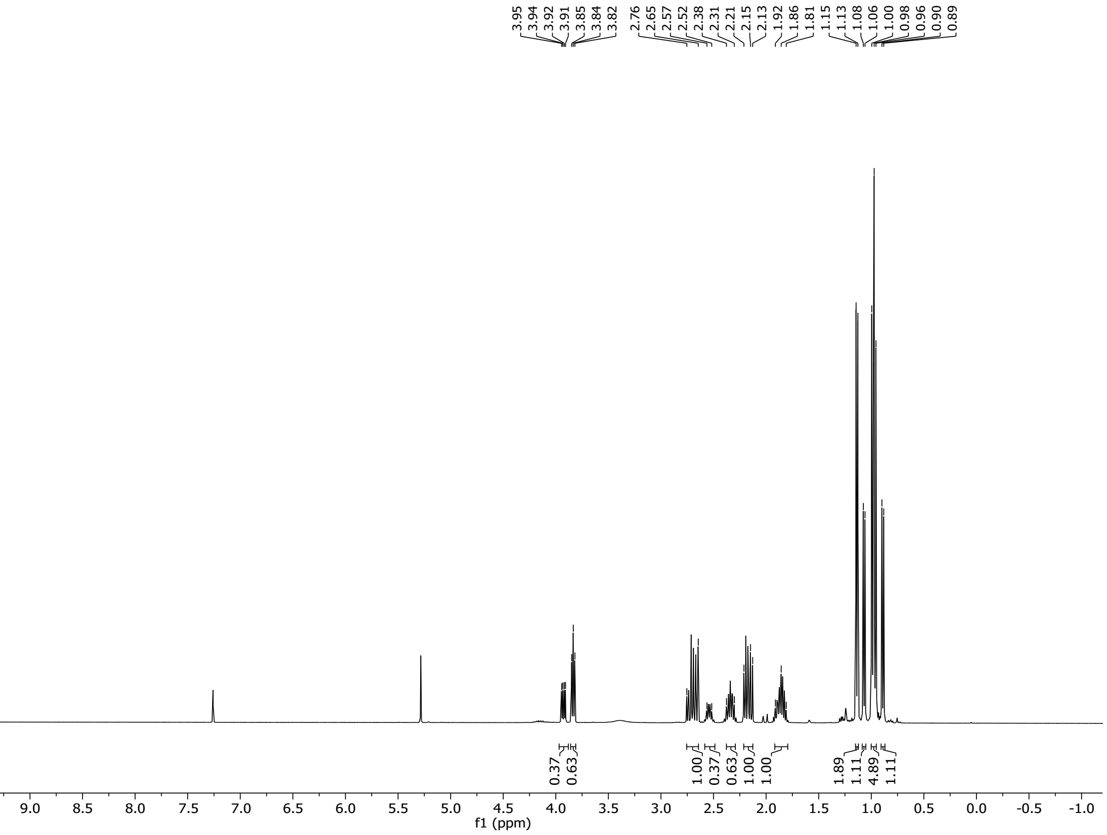

^{13}C{^{1}H}-NMR (CDCl$_3$, 101 MHz)

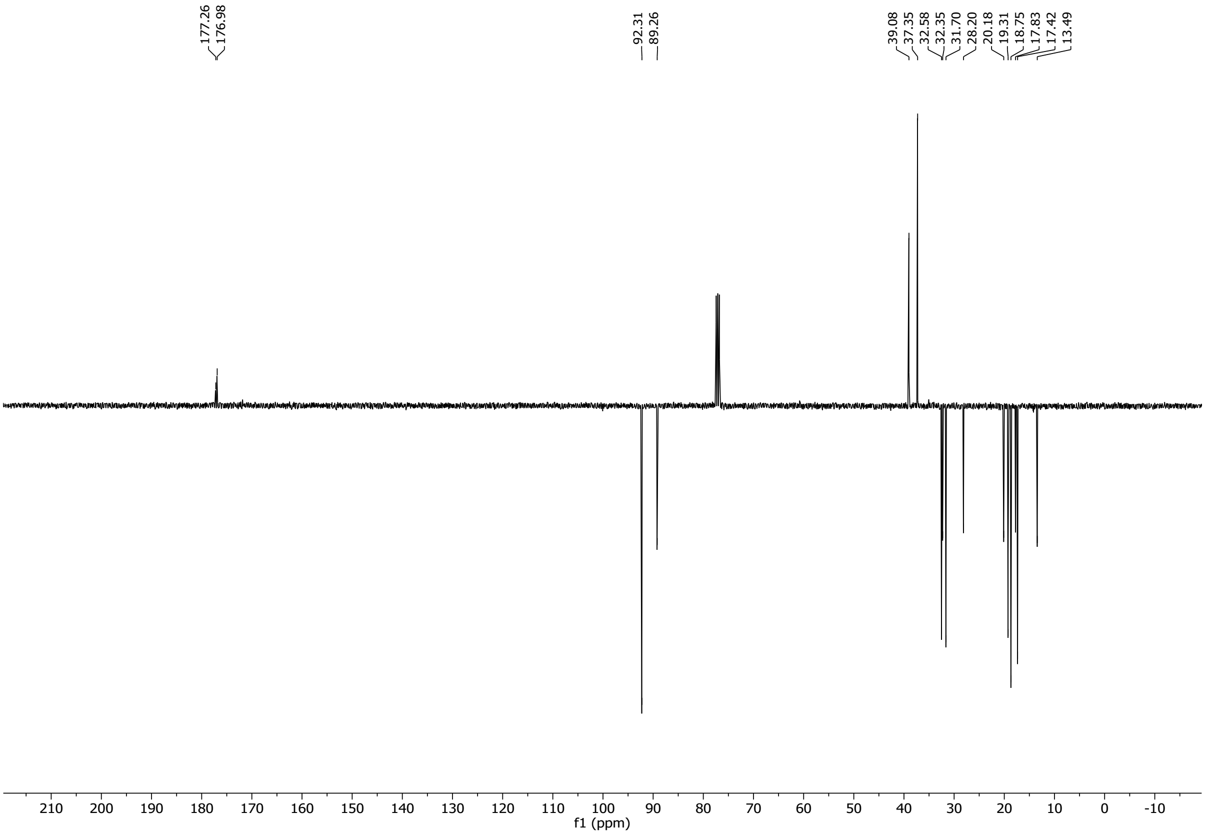

^{1}H-NMR ($CDCl_3$, 400 MHz)

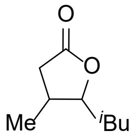

1d

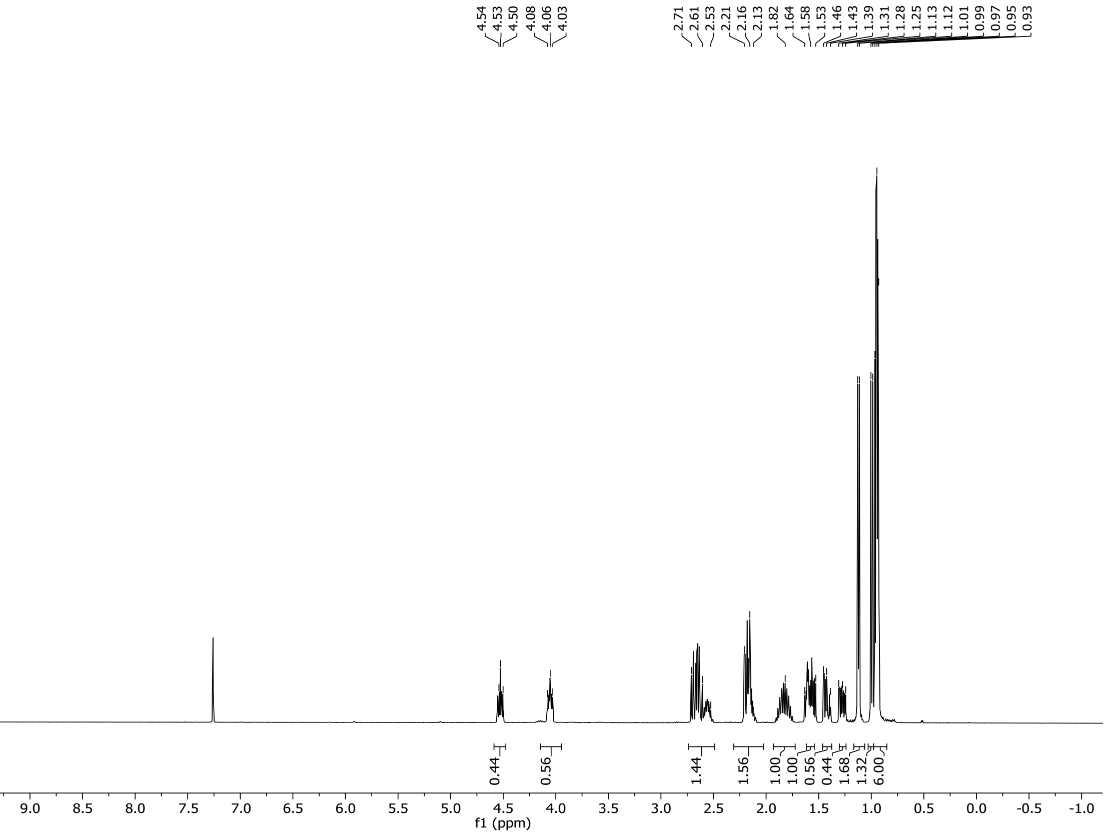

^{13}C{^{1}H}-NMR ($CDCl_3$, 101 MHz)

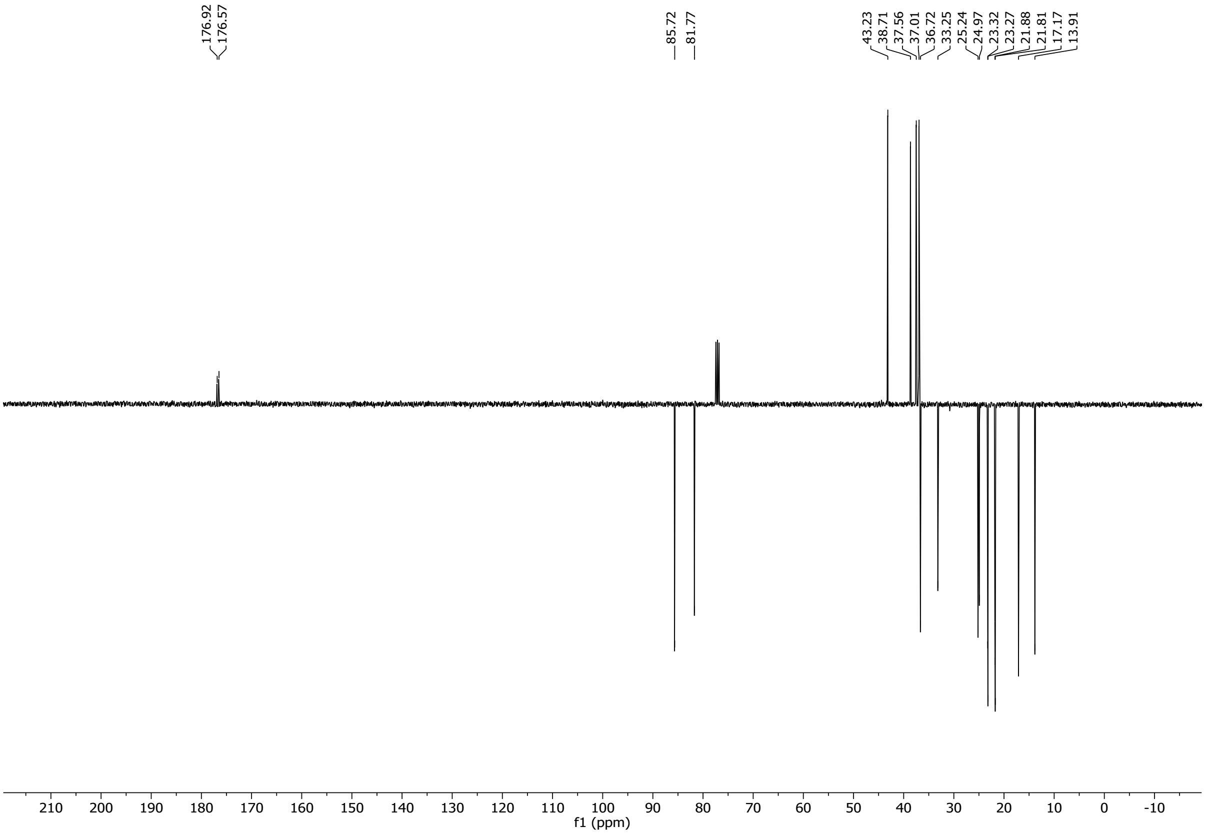

^{1}H-NMR ($CDCl_3$, 400 MHz)

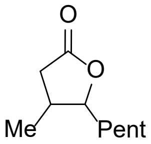

1e

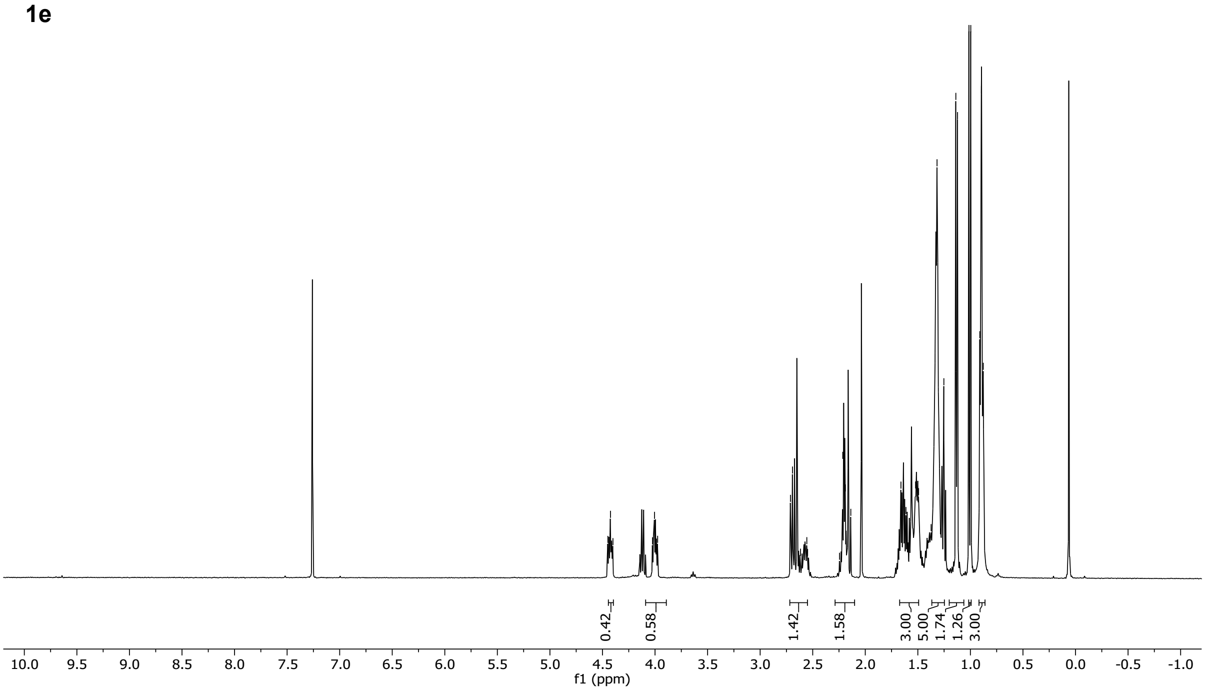

^{13}C{^{1}H}-NMR ($CDCl_3$, 101 MHz)

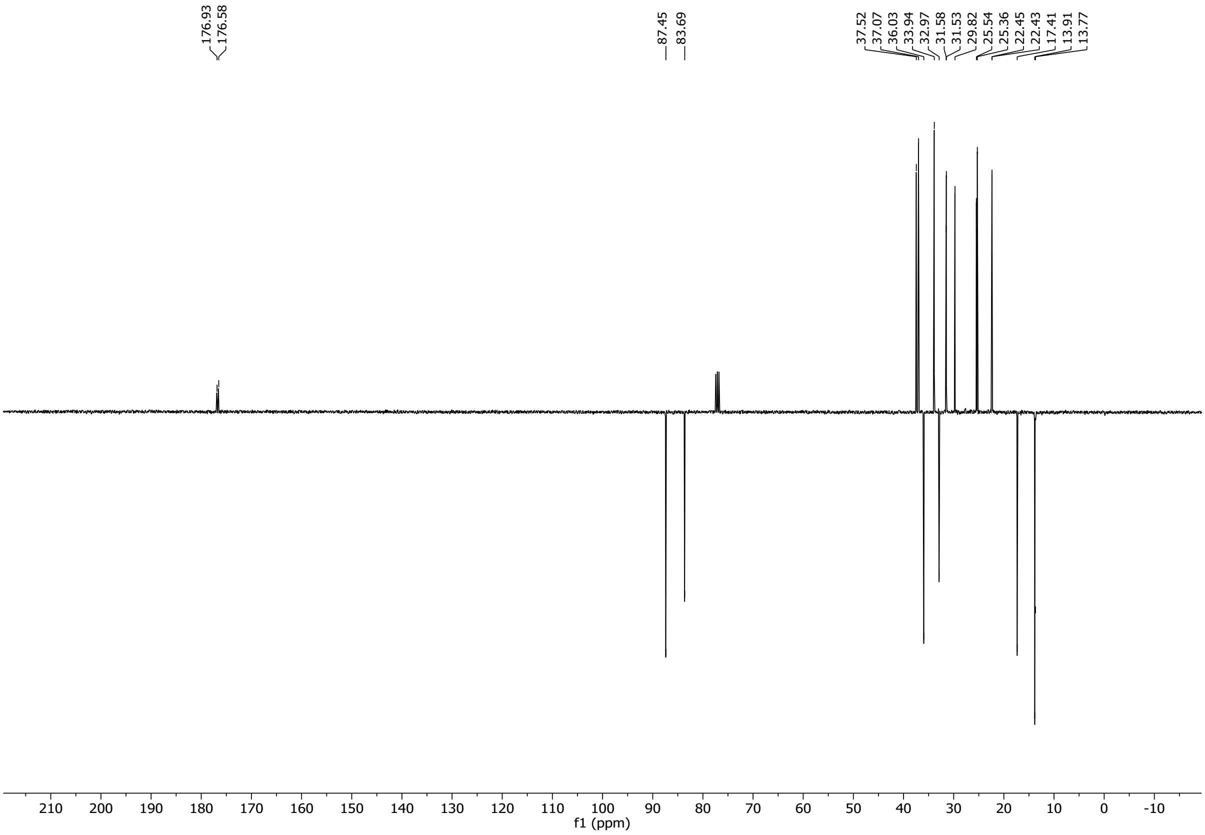

^{1}H-NMR ($CDCl_3$, 400 MHz)

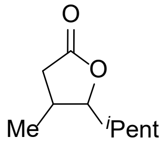

1f

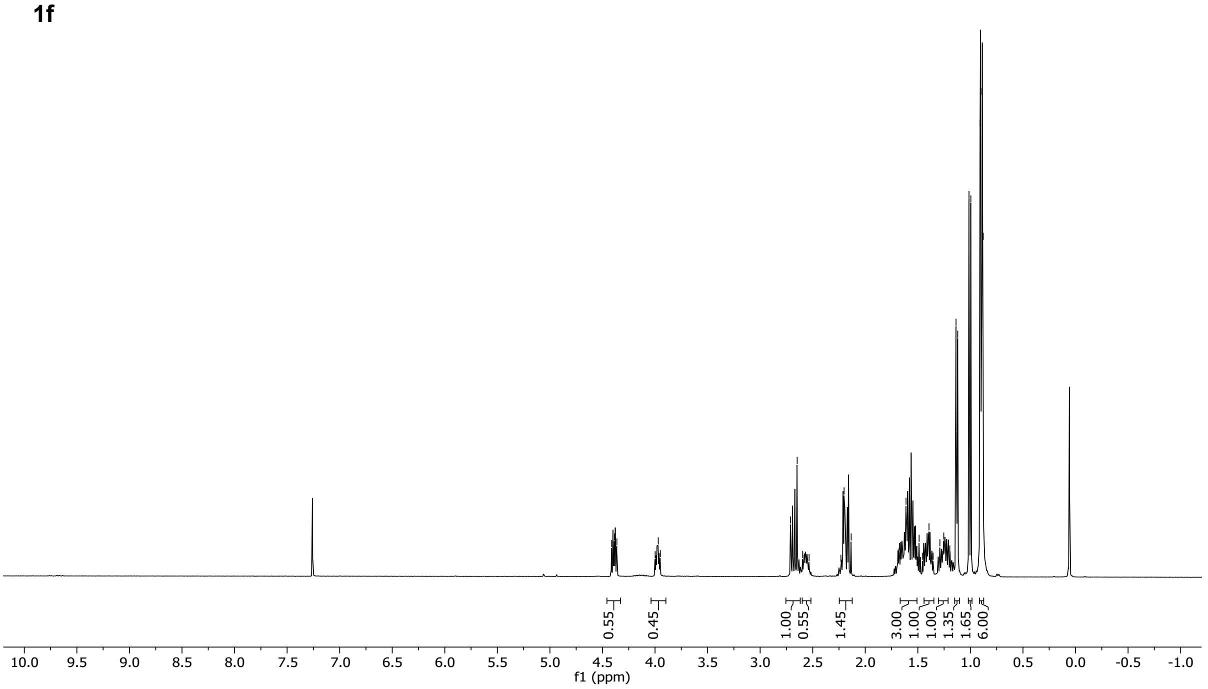

^{13}C{^{1}H}-NMR ($CDCl_3$, 101 MHz)

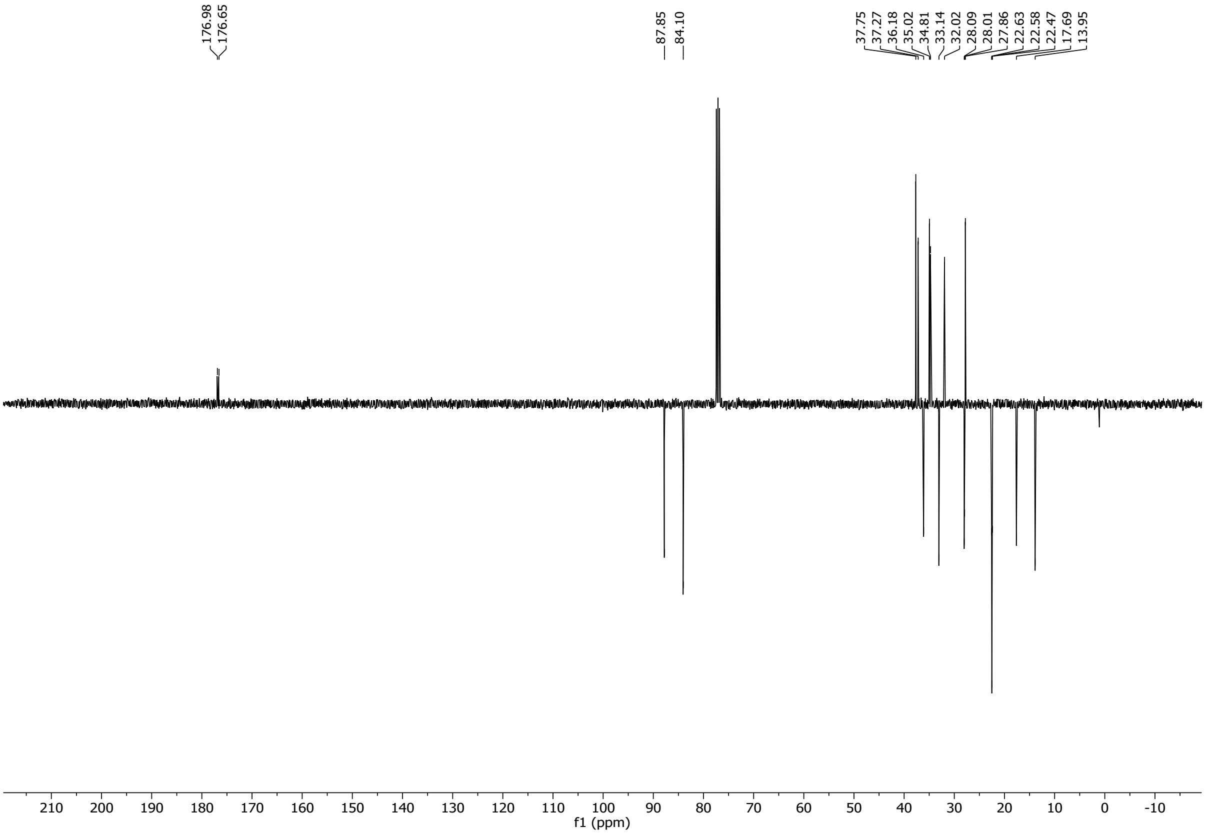

^{1}H-NMR ($CDCl_3$, 400 MHz)

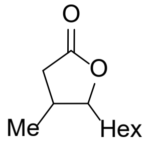

1g

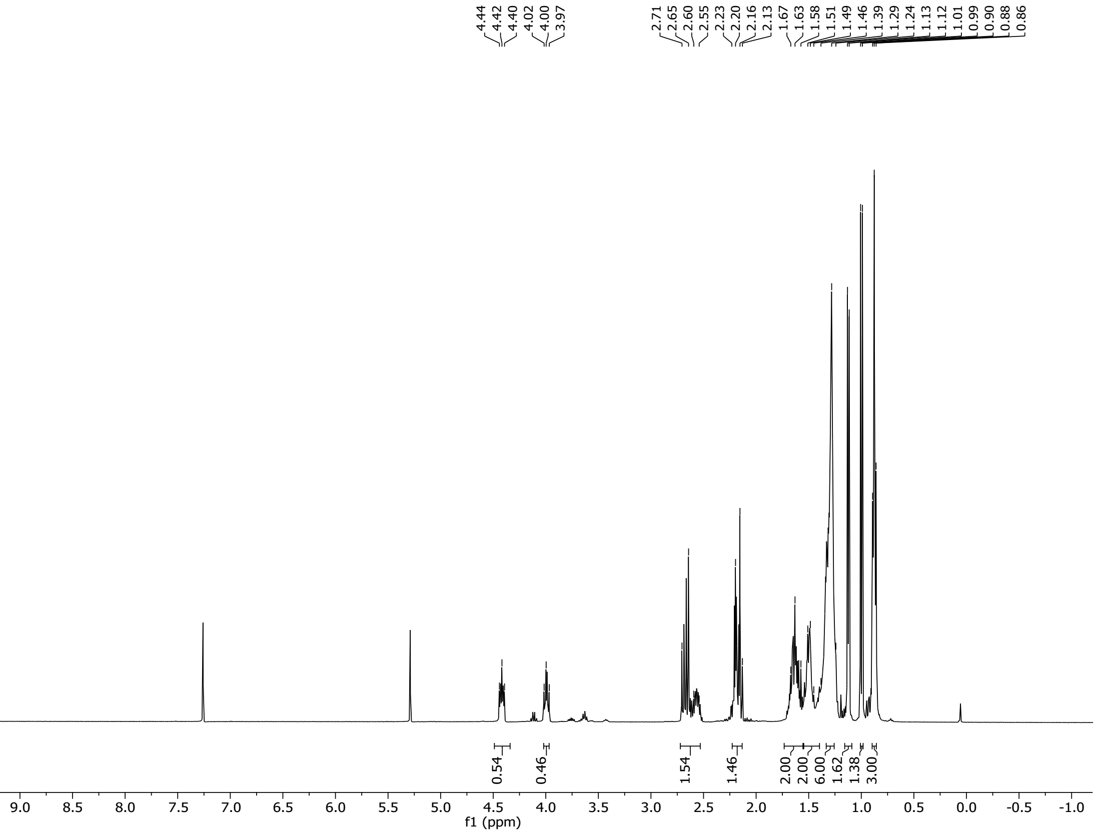

^{13}C{^{1}H}-NMR ($CDCl_3$, 101 MHz)

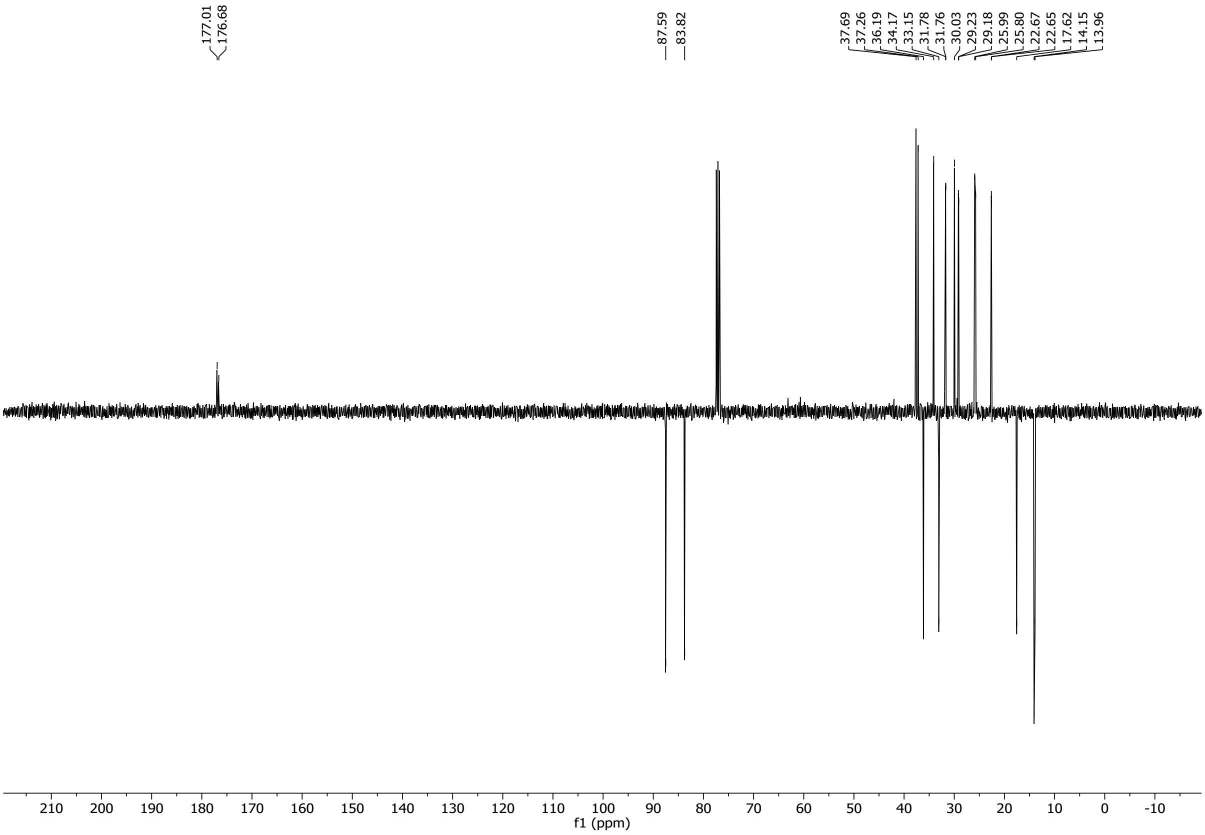

^{1}H-NMR ($CDCl_3$, 400 MHz)

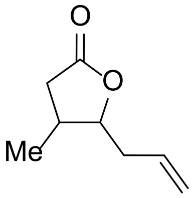

1h

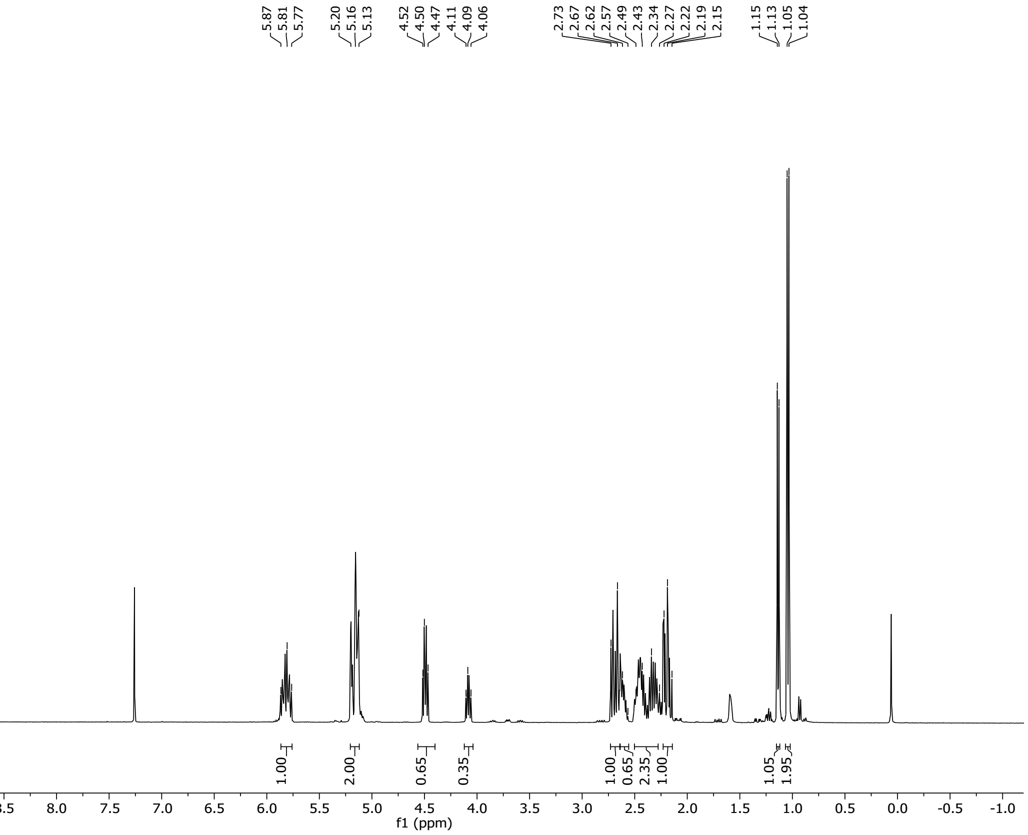

^{13}C{^{1}H}-NMR ($CDCl_3$, 101 MHz)

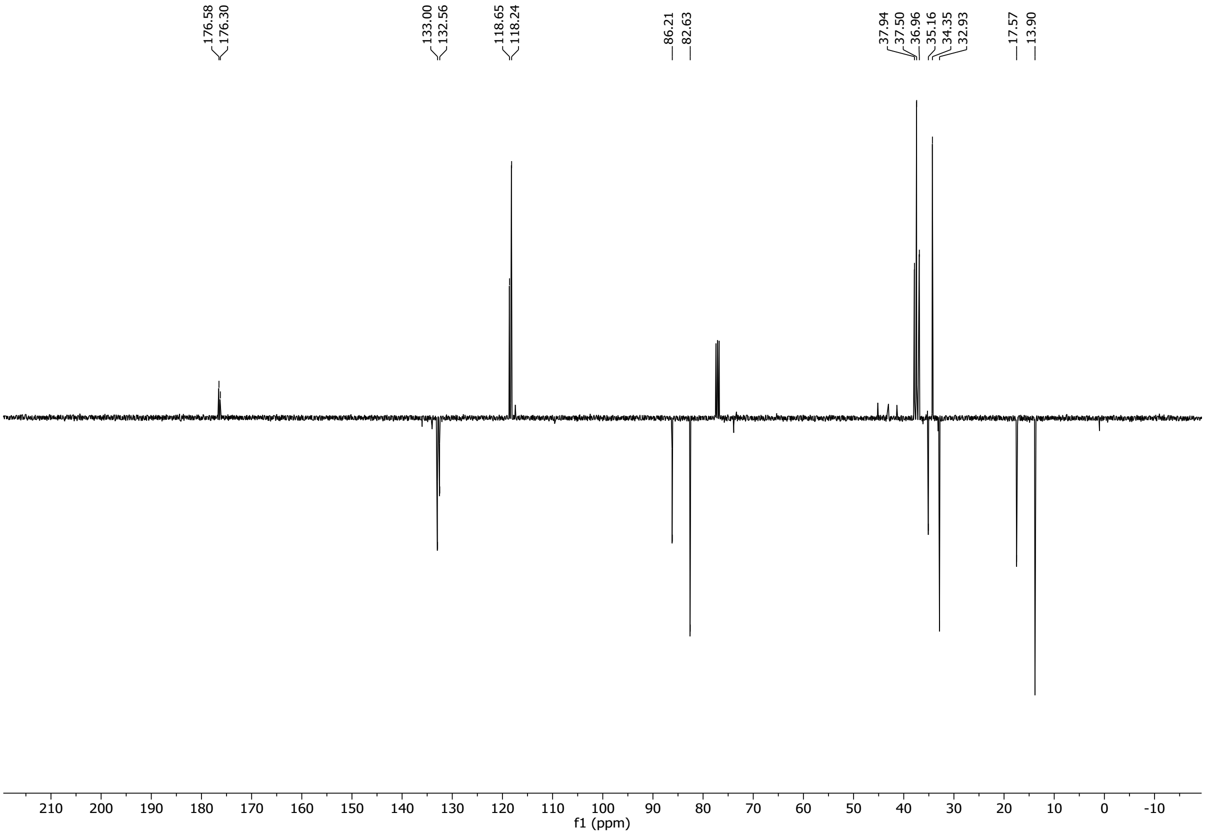

^{1}H-NMR ($CDCl_3$, 400 MHz)

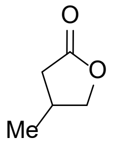

1l

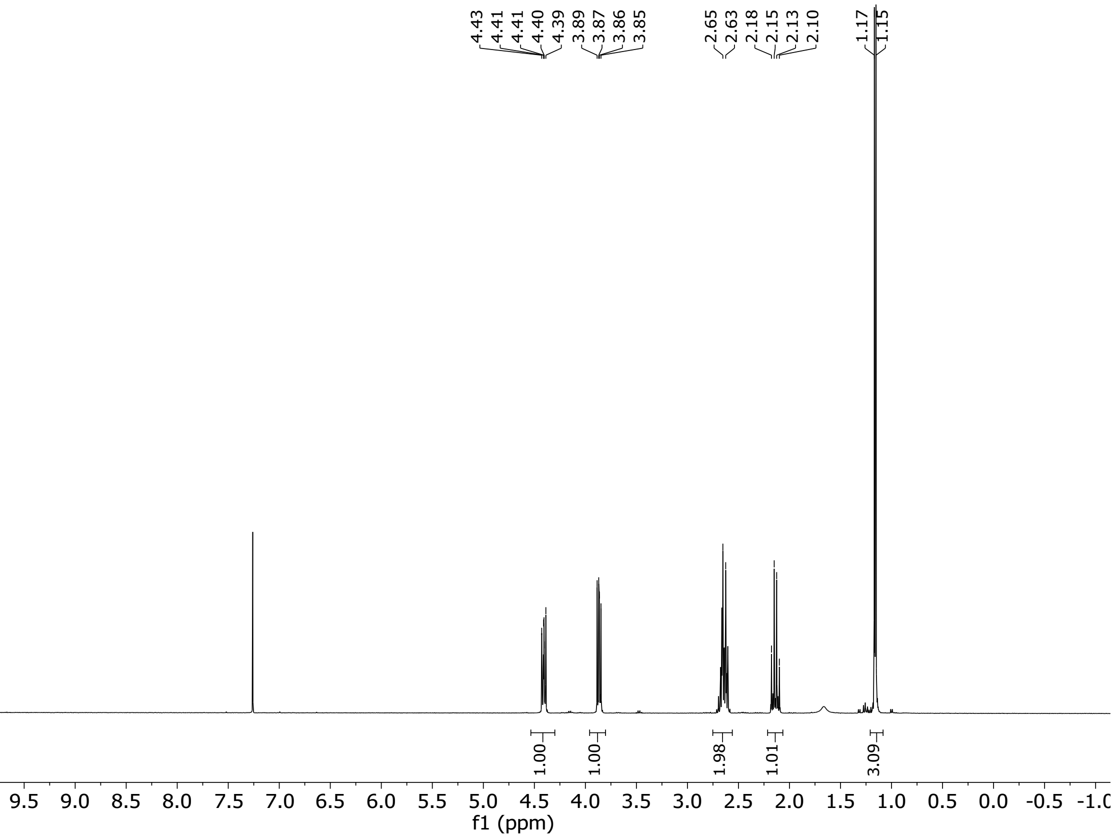

^{13}C{^{1}H}-NMR ($CDCl_3$, 101 MHz)

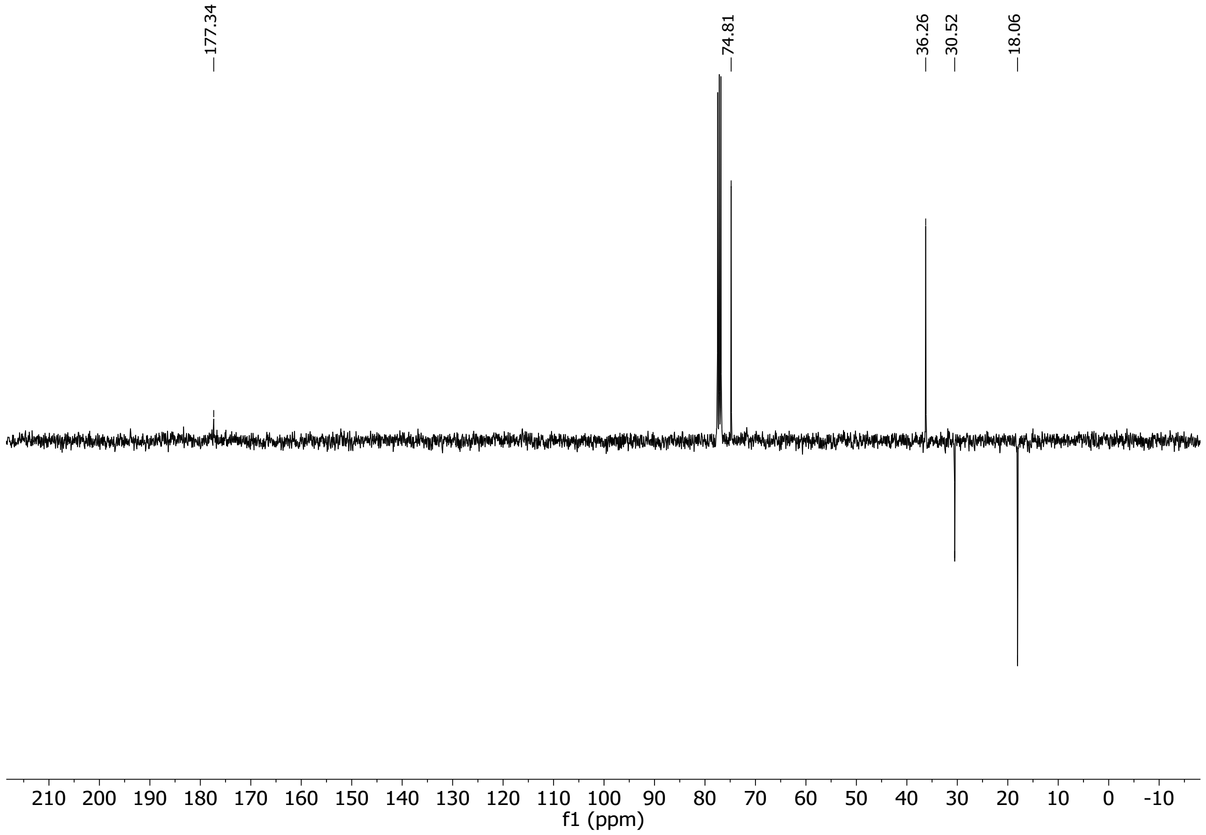

^{1}H-NMR ($CDCl_3$, 400 MHz)

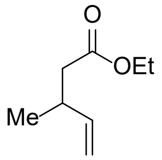

10

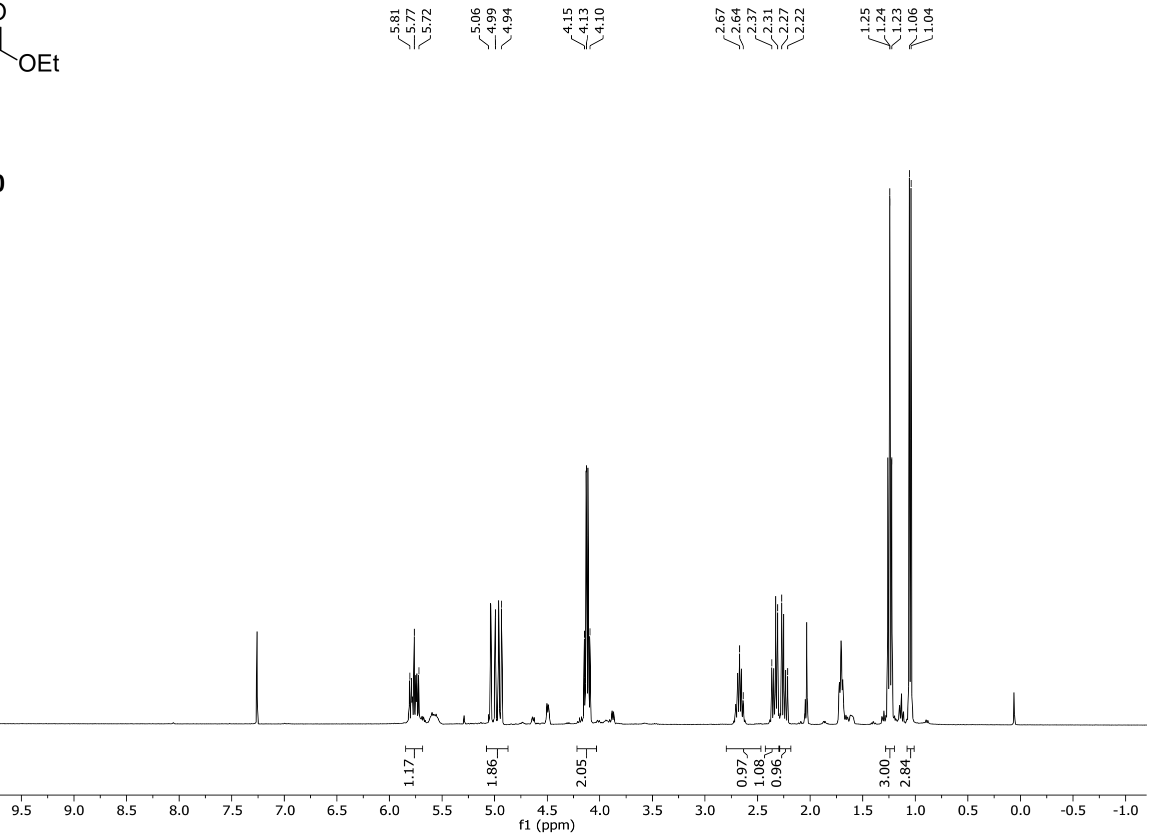

^{13}C{^{1}H}-NMR ($CDCl_3$, 101 MHz)

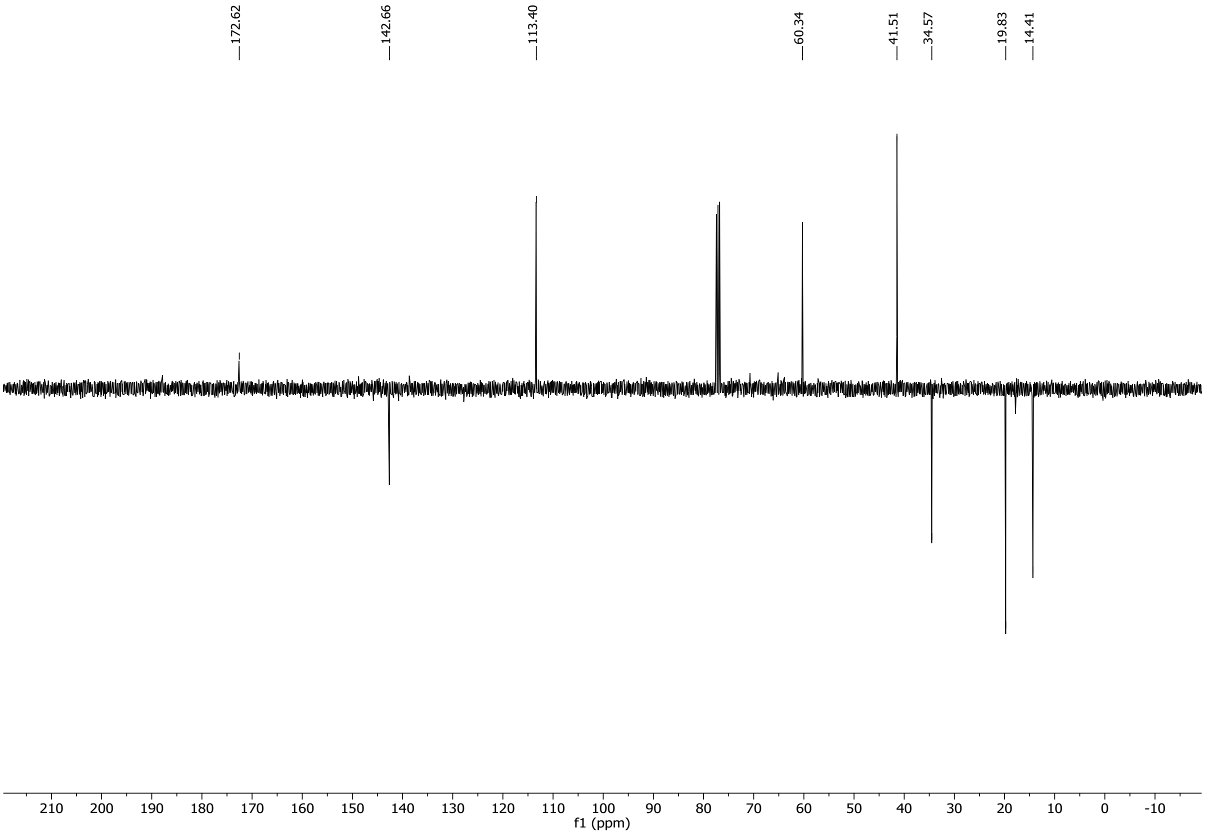

^{1}H-NMR ($CDCl_3$, 400 MHz)

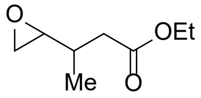

11

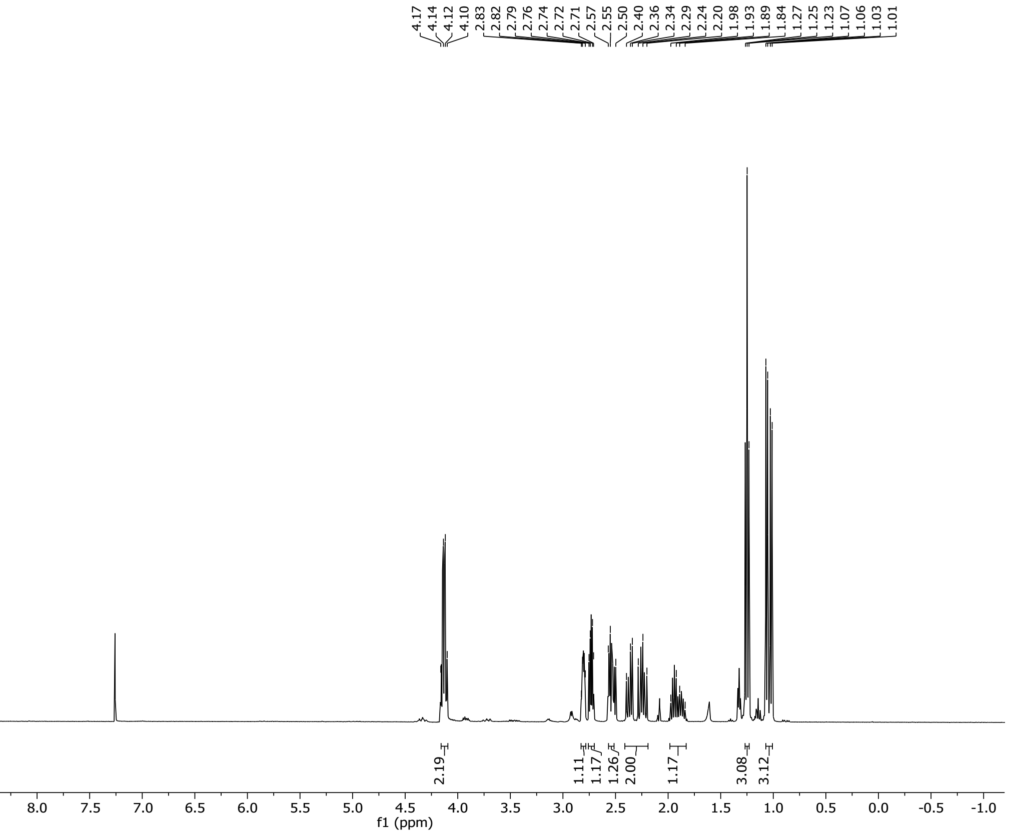

^{13}C{^{1}H}-NMR ($CDCl_3$, 101 MHz)

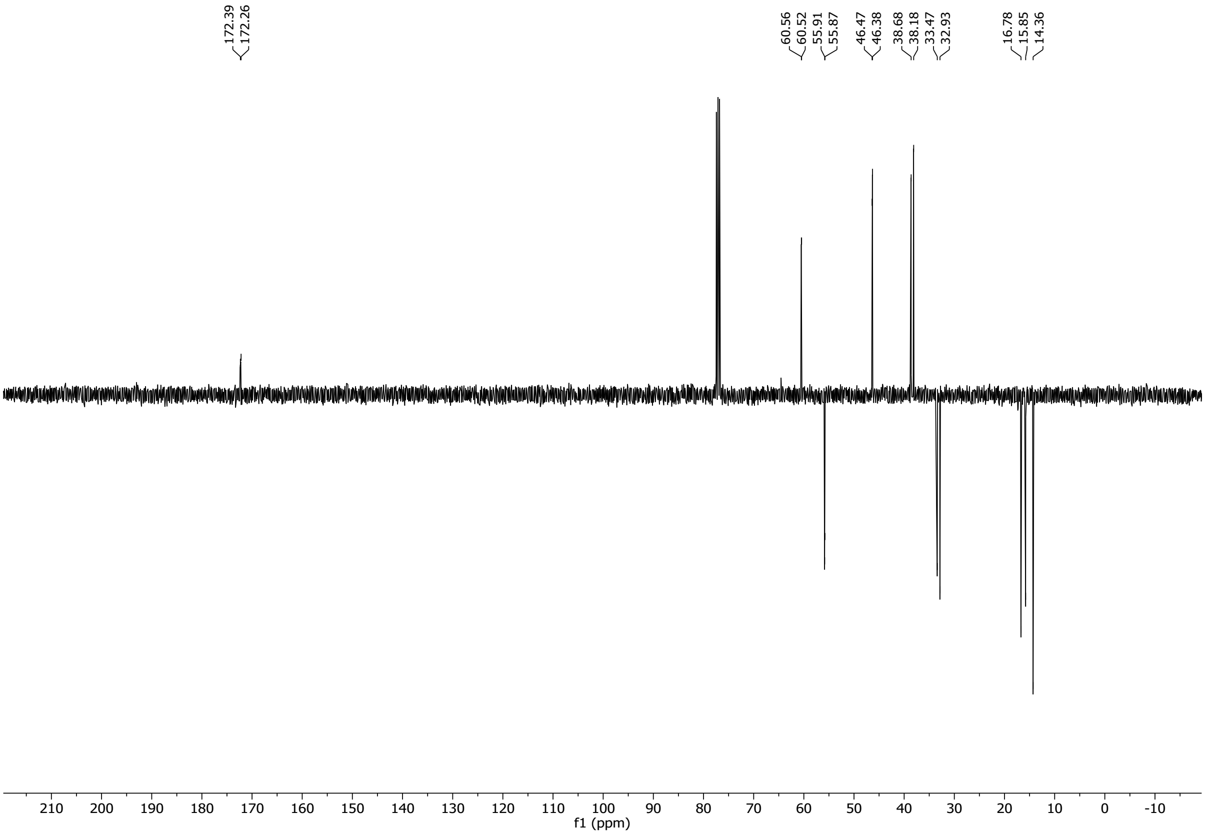

^{1}H-NMR ($CDCl_3$, 400 MHz)

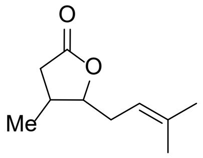

1i

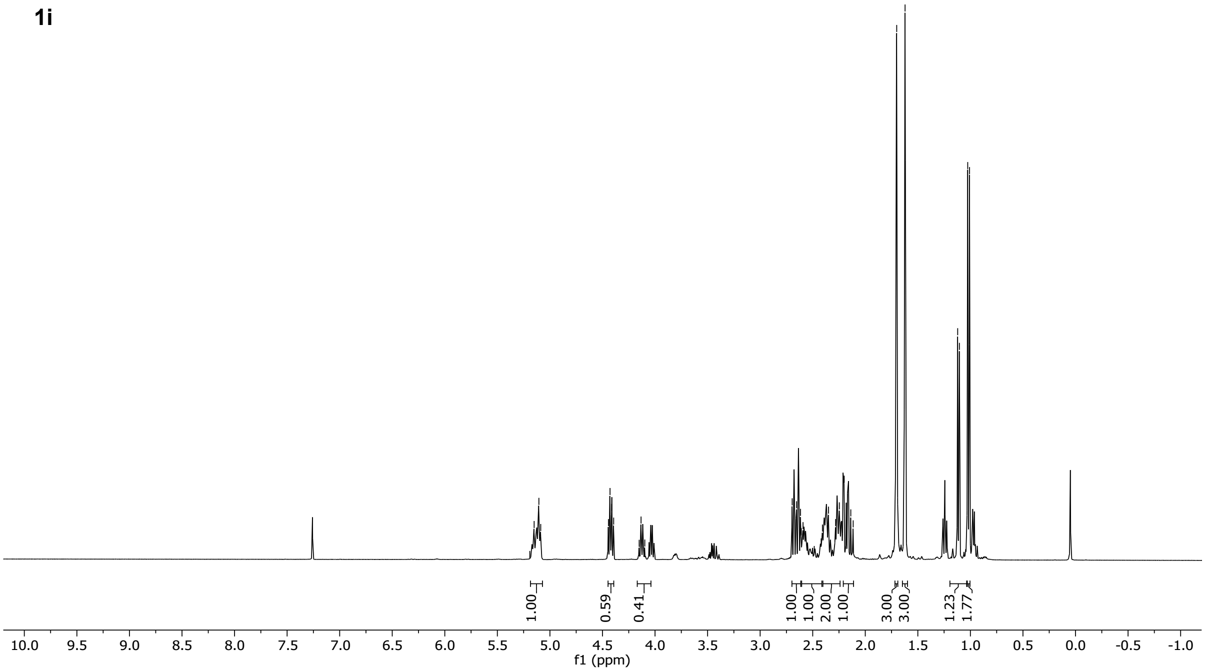

^{13}C{^{1}H}-NMR ($CDCl_3$, 101 MHz)

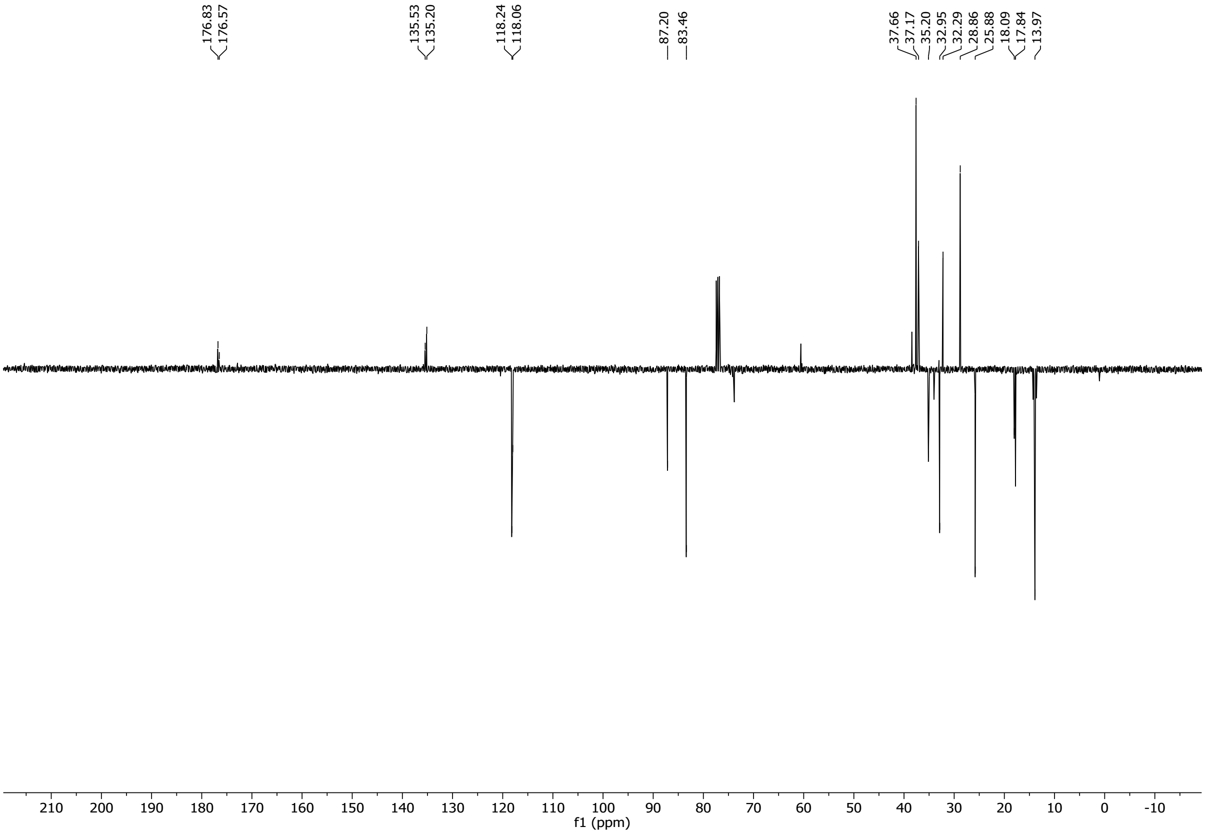

^{1}H-NMR ($CDCl_3$, 400 MHz)

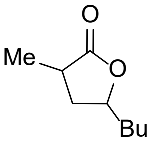

1m

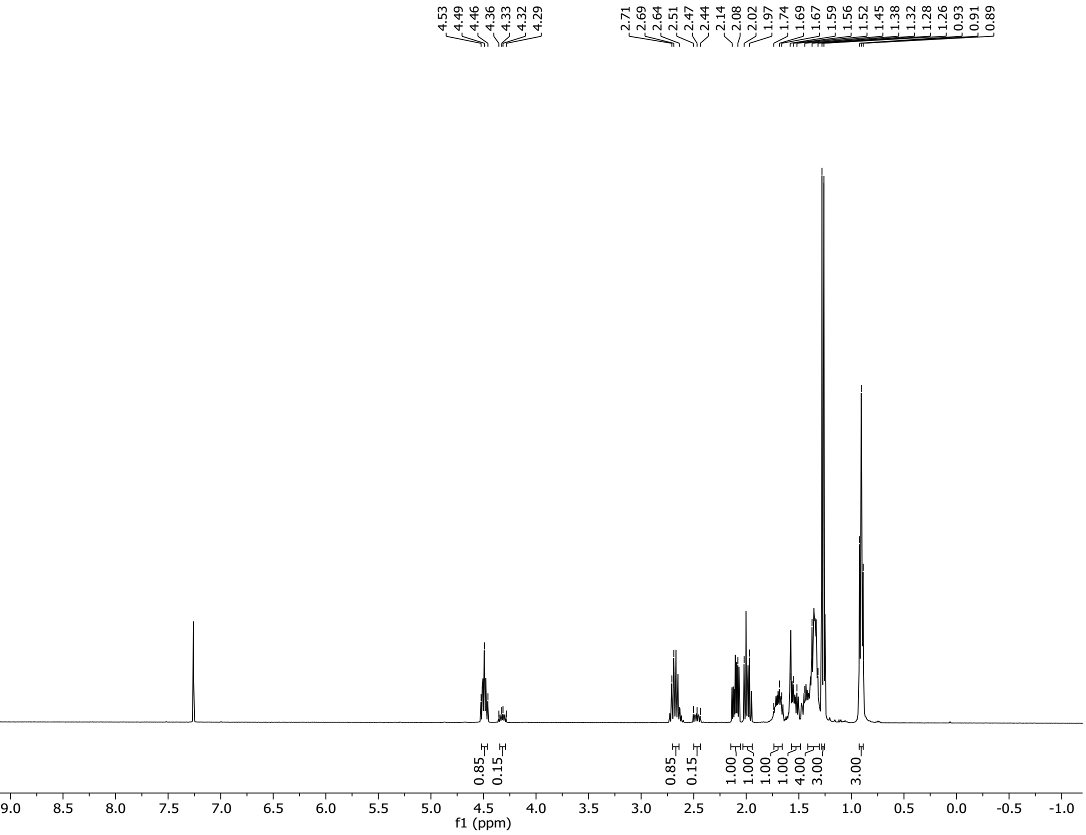

^{13}C{^{1}H}-NMR ($CDCl_3$, 101 MHz)

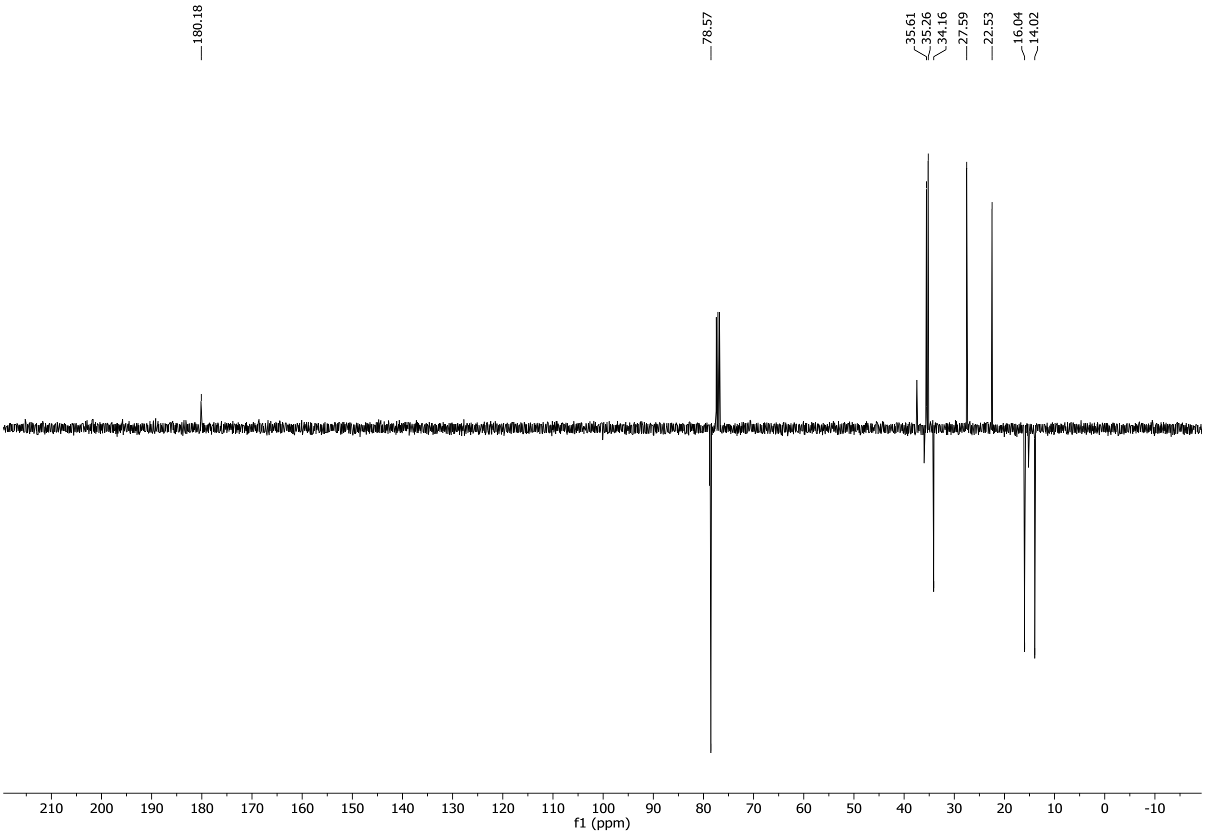

^{1}H-NMR (CDCl$_3$, 400 MHz)

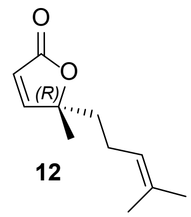

12

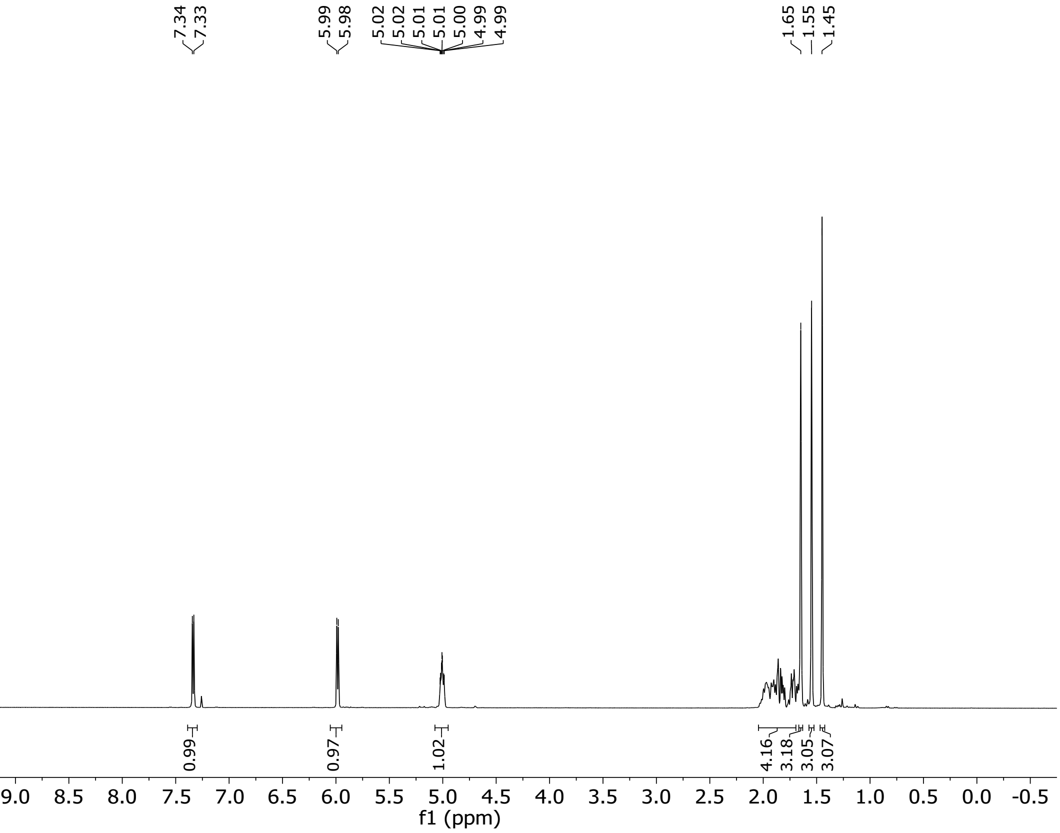

^{13}C{^{1}H}-NMR (CDCl$_3$, 101 MHz)

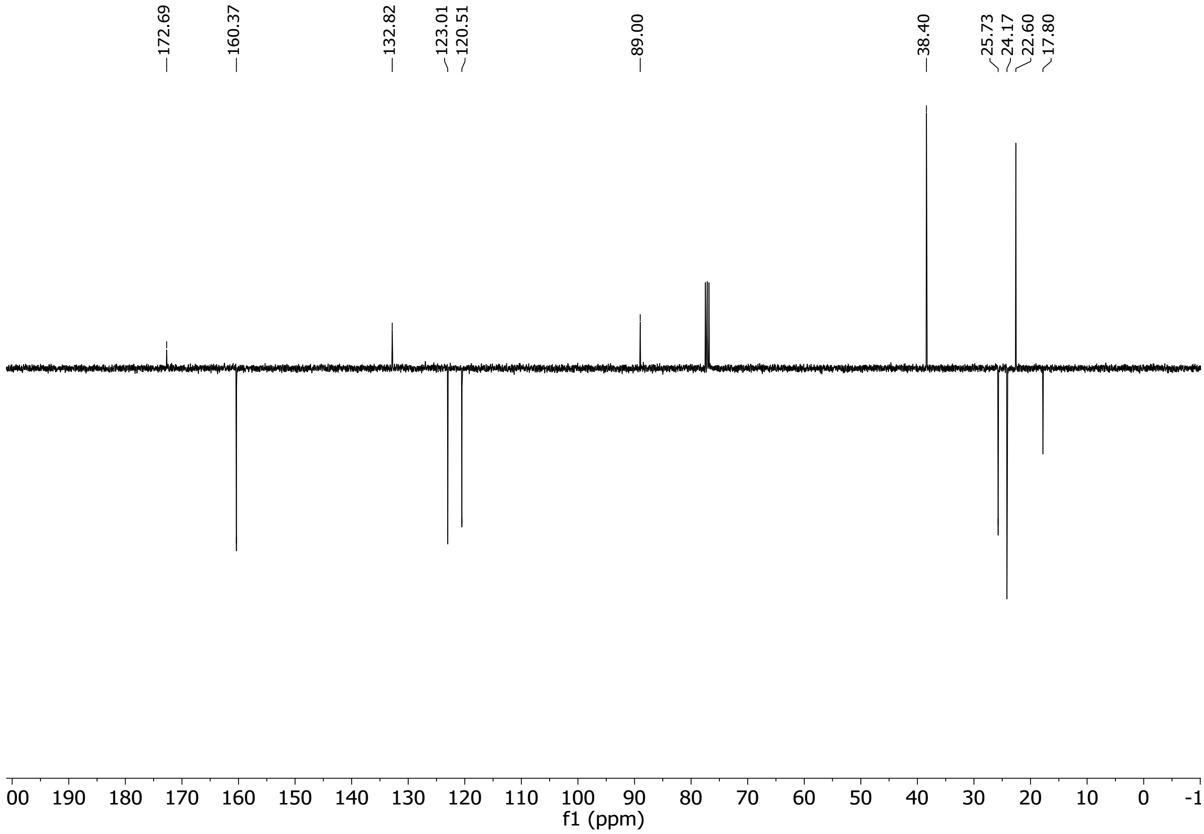

^{1}H-NMR ($CDCl_3$, 400 MHz)

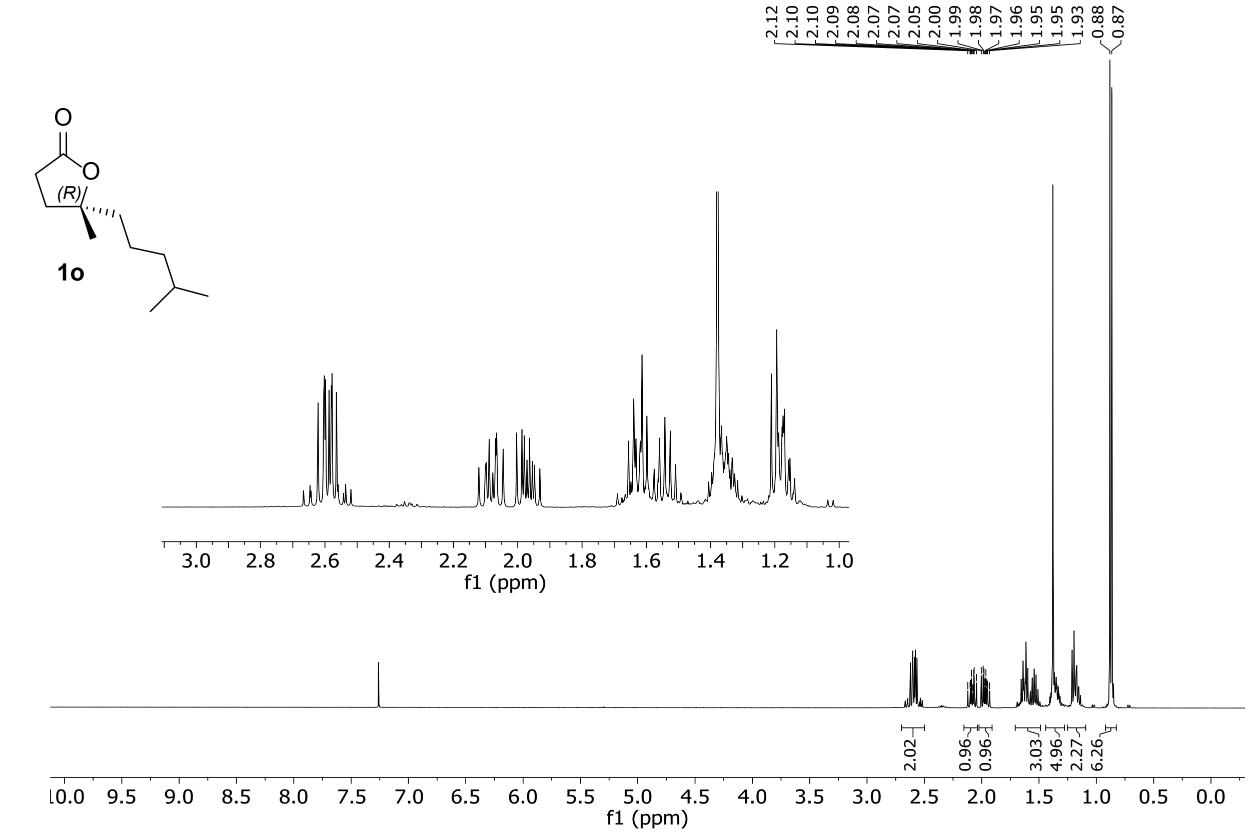

^{13}C{^{1}H}-NMR ($CDCl_3$, 101 MHz)

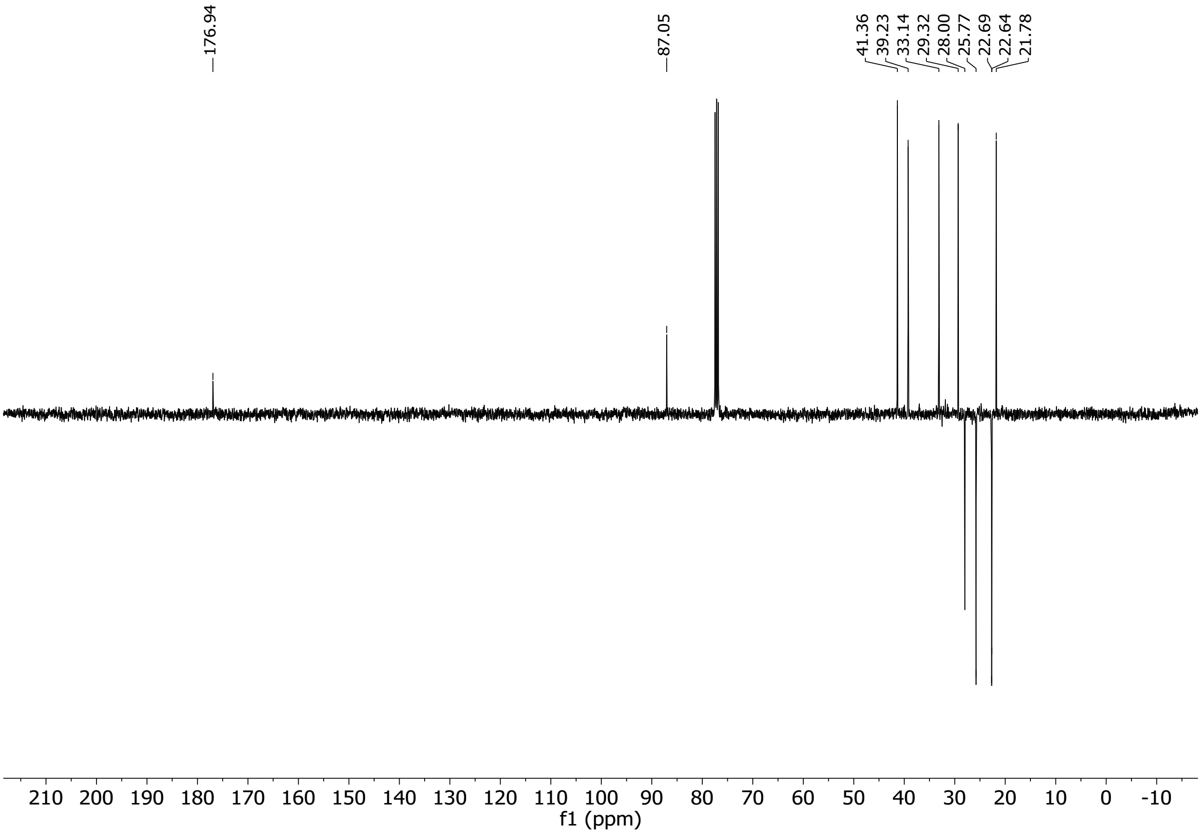

^{1}H-NMR ($CDCl_3$, 400 MHz)

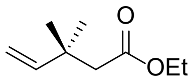

13

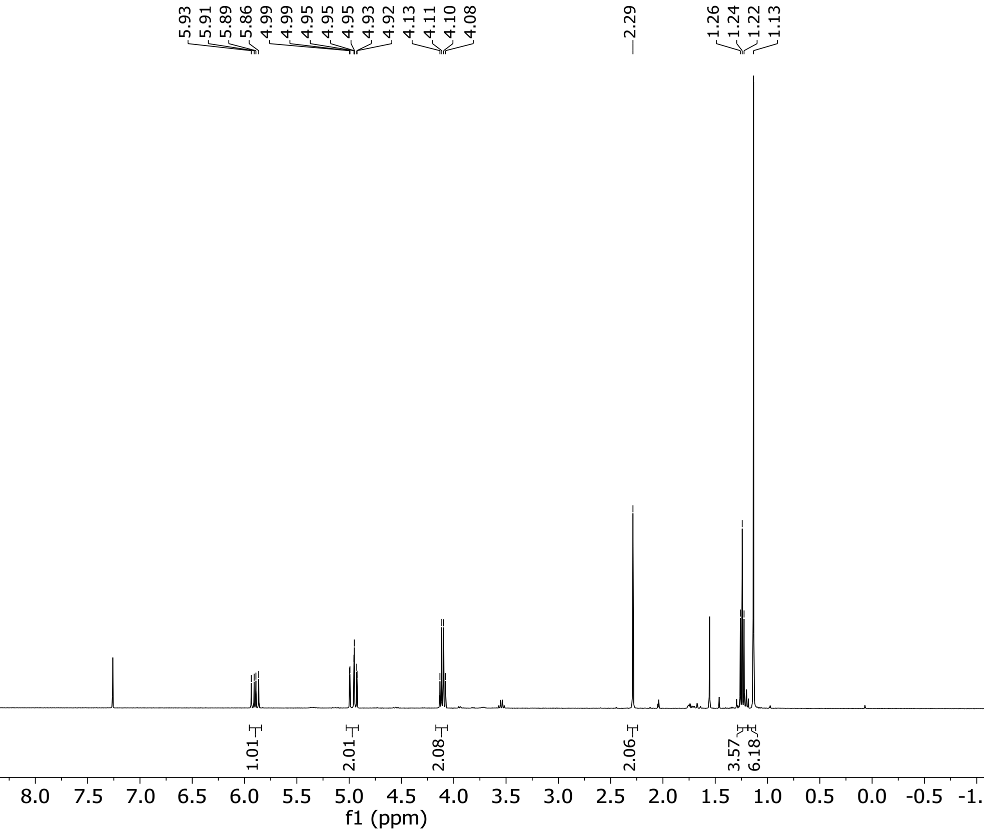

^{13}C{^{1}H}-NMR ($CDCl_3$, 101 MHz)

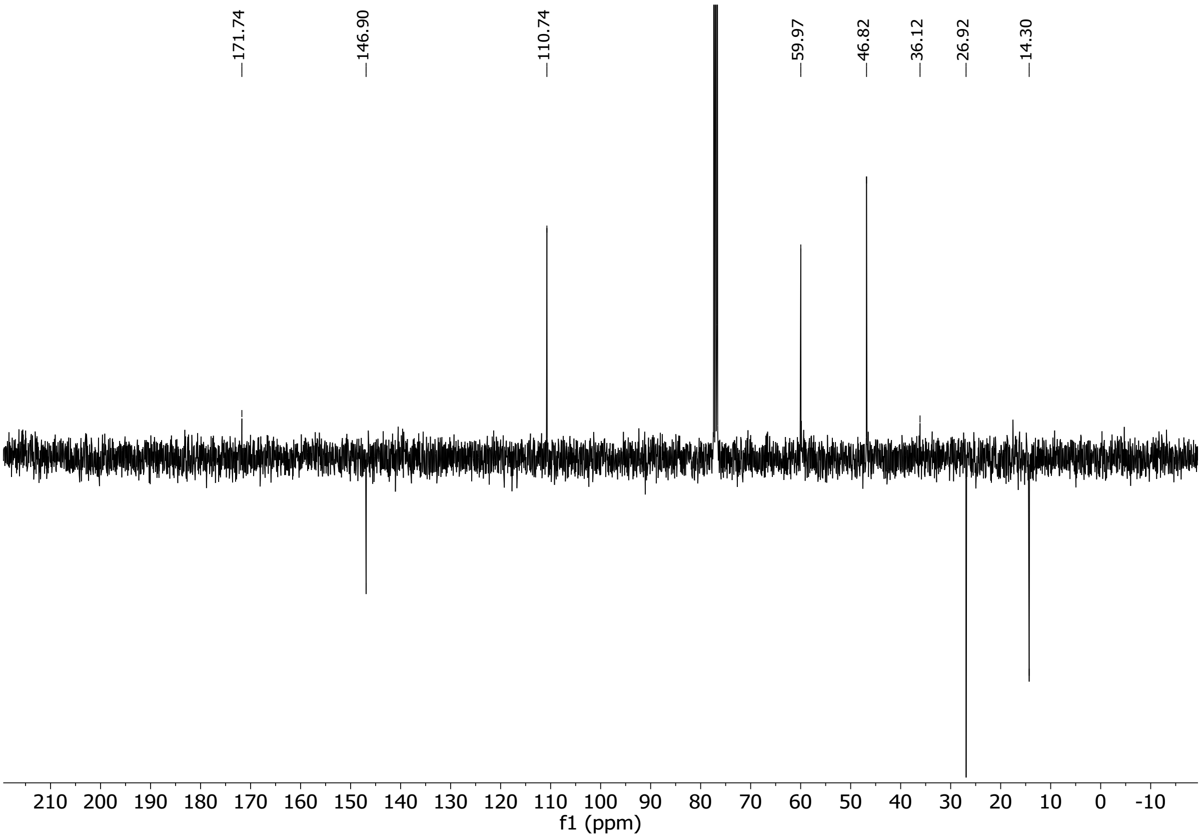

^{1}H-NMR ($CDCl_3$, 400 MHz)

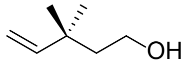

14

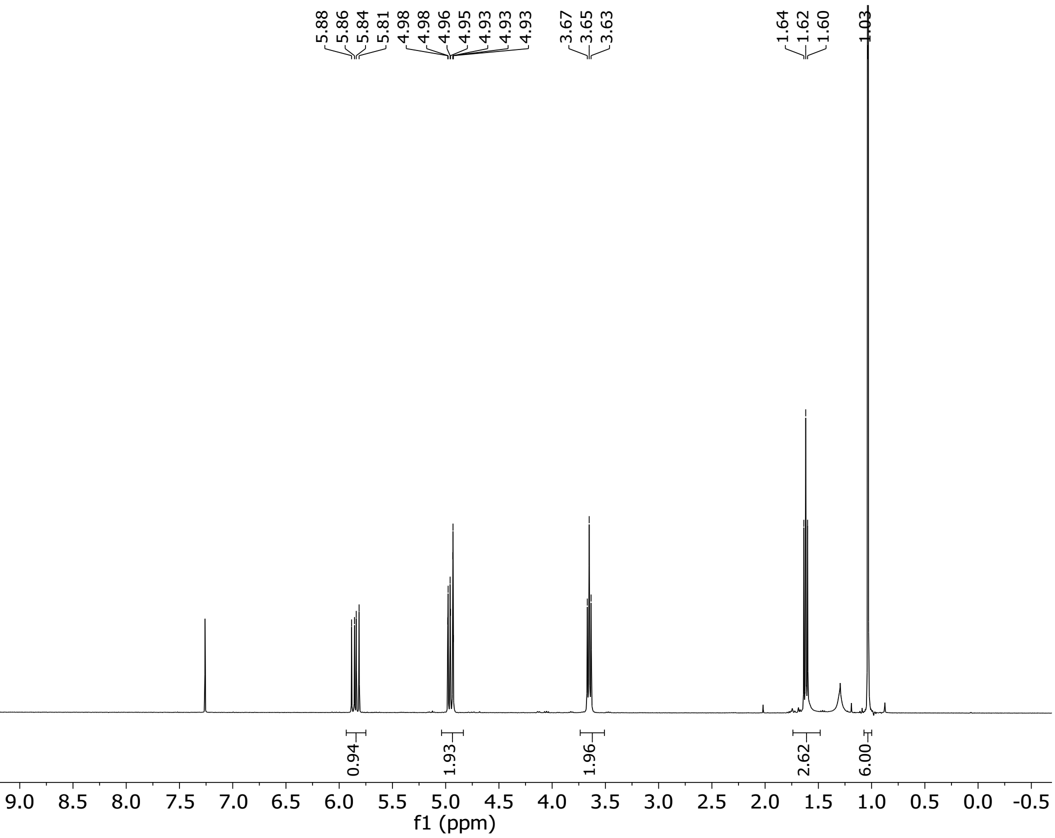

^{13}C{^{1}H}-NMR ($CDCl_3$, 101 MHz)

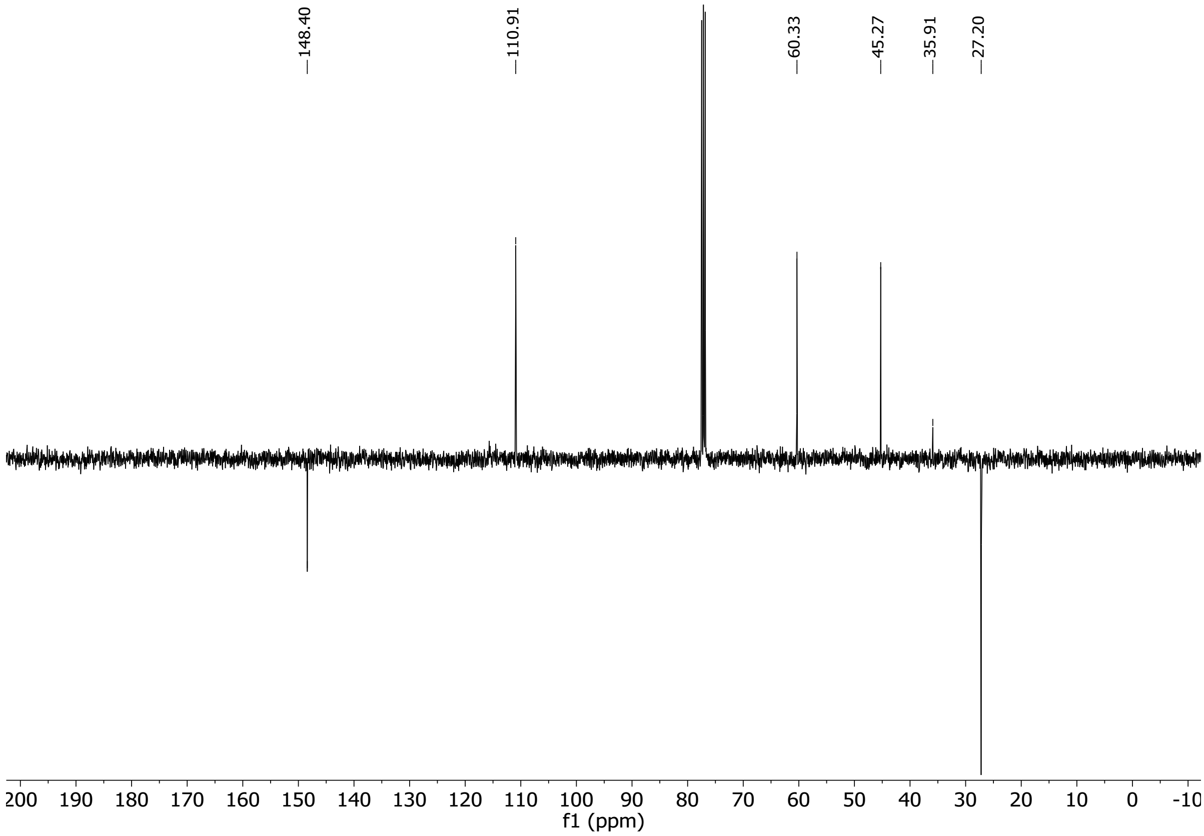

^{1}H-NMR ($CDCl_3$, 400 MHz)

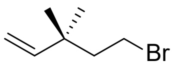

15

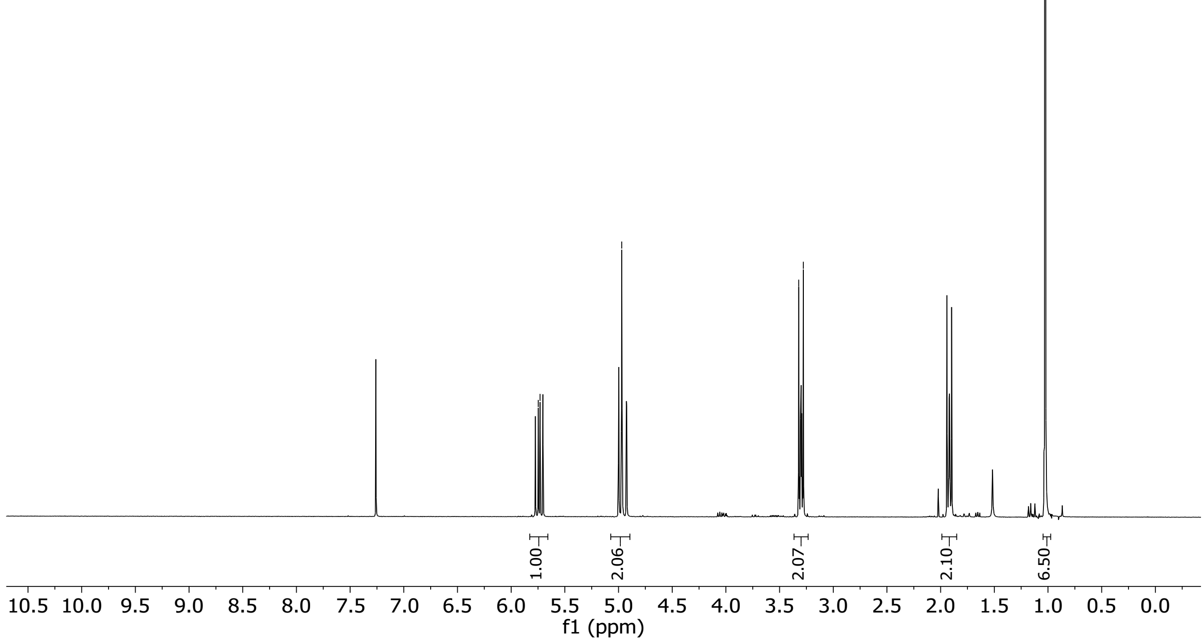

^{13}C{^{1}H}-NMR ($CDCl_3$, 101 MHz)

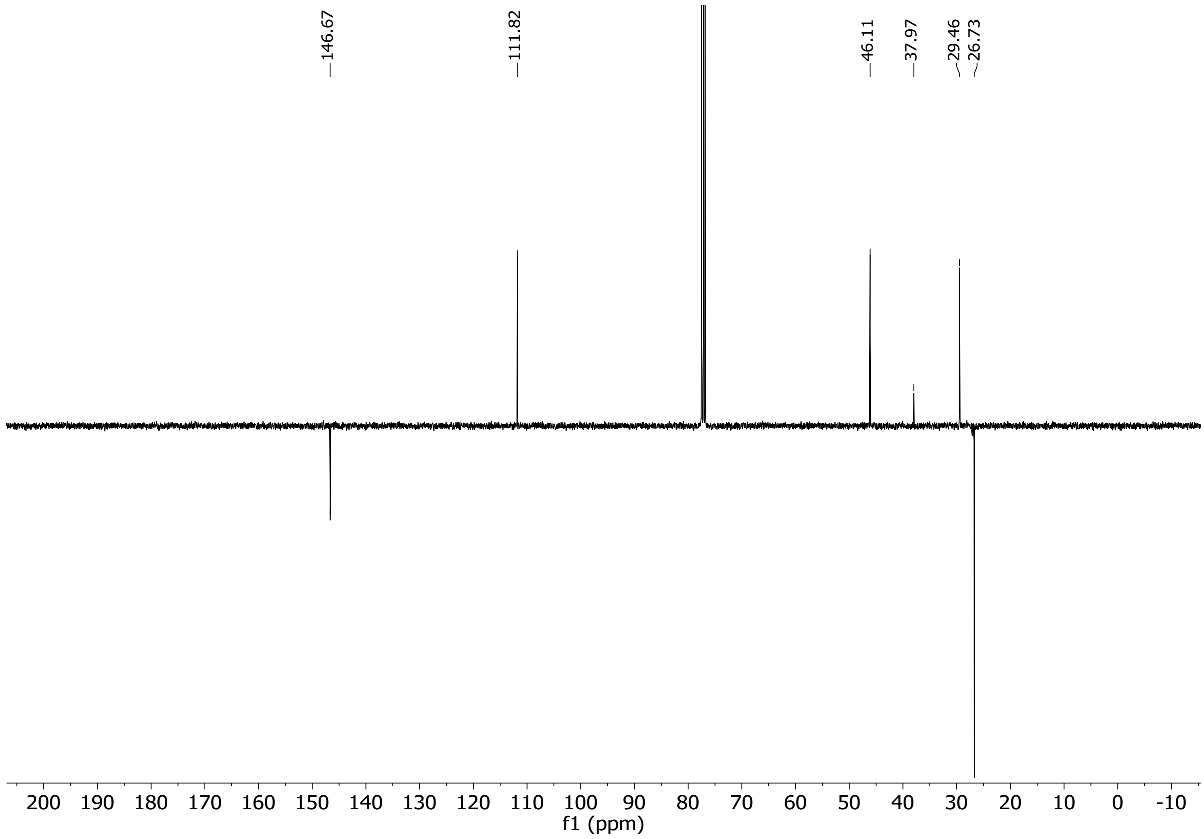

^{1}H-NMR ($CDCl_3$, 400 MHz)

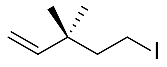

17

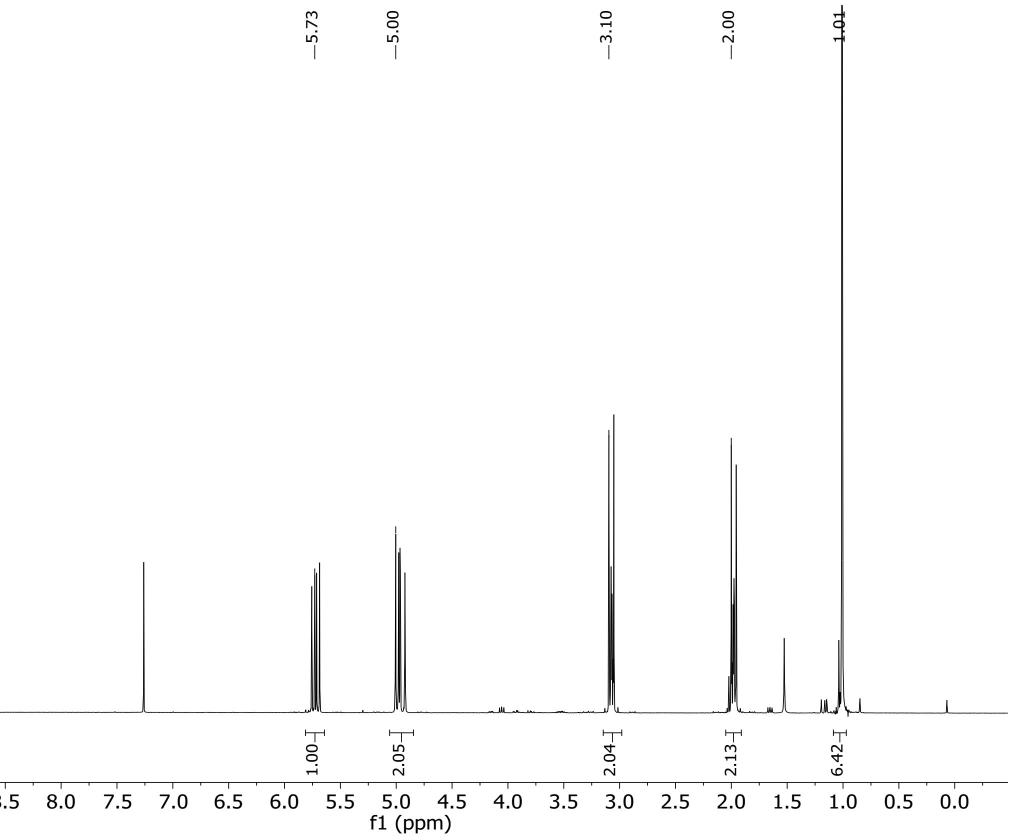

^{13}C{^{1}H}-NMR ($CDCl_3$, 101 MHz)

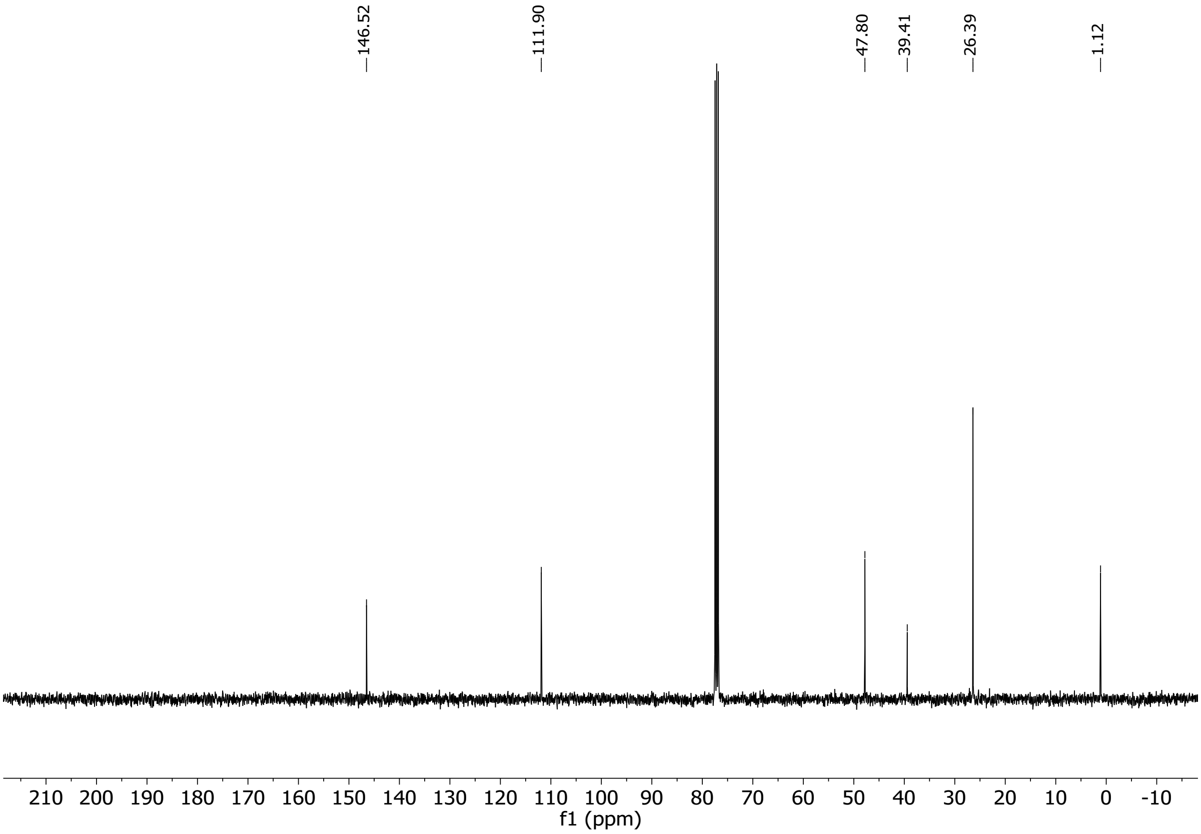

^{1}H-NMR ($CDCl_3$, 400 MHz)

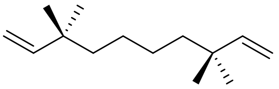

16

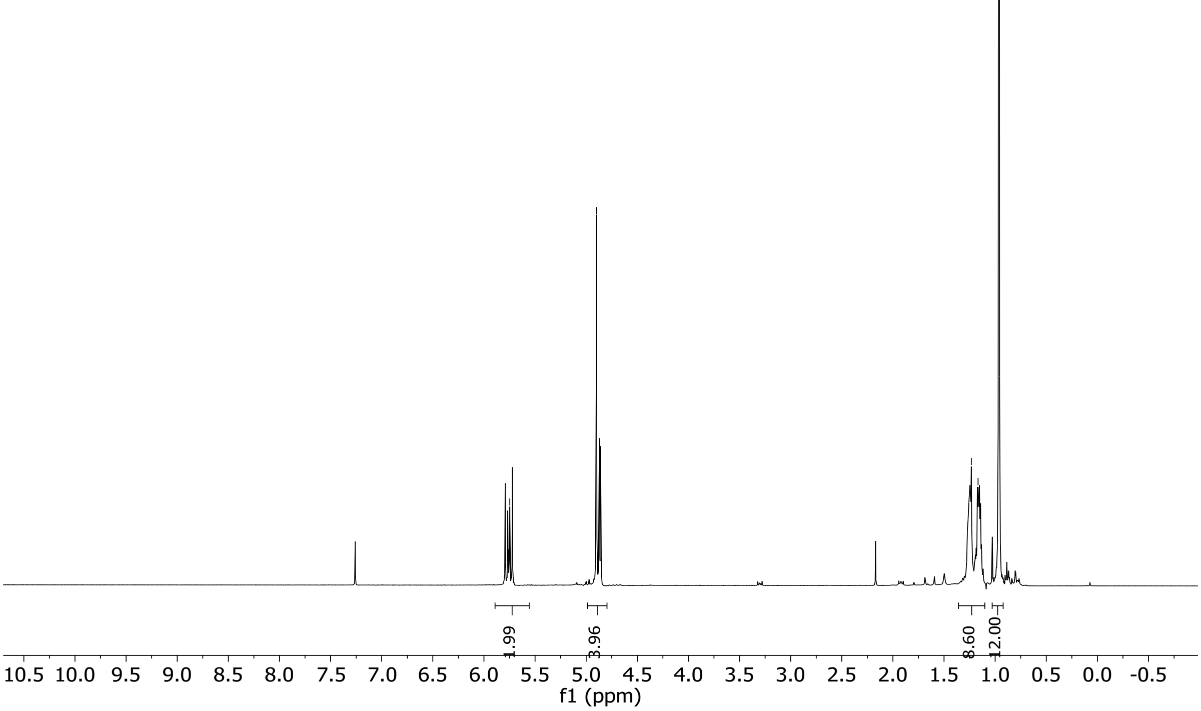

$^{13}C\{^1H\}$-NMR ($CDCl_3$, 101 MHz)

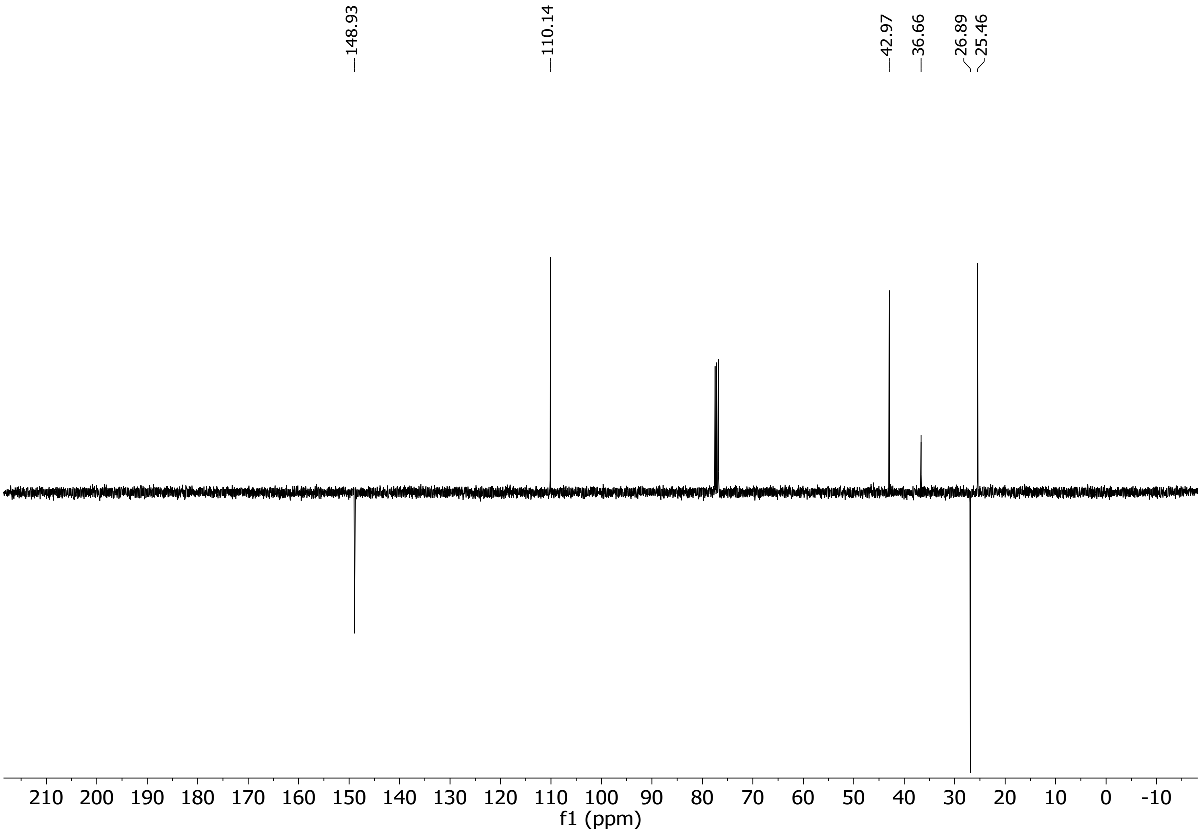

^{1}H-NMR ($CDCl_3$, 400 MHz)

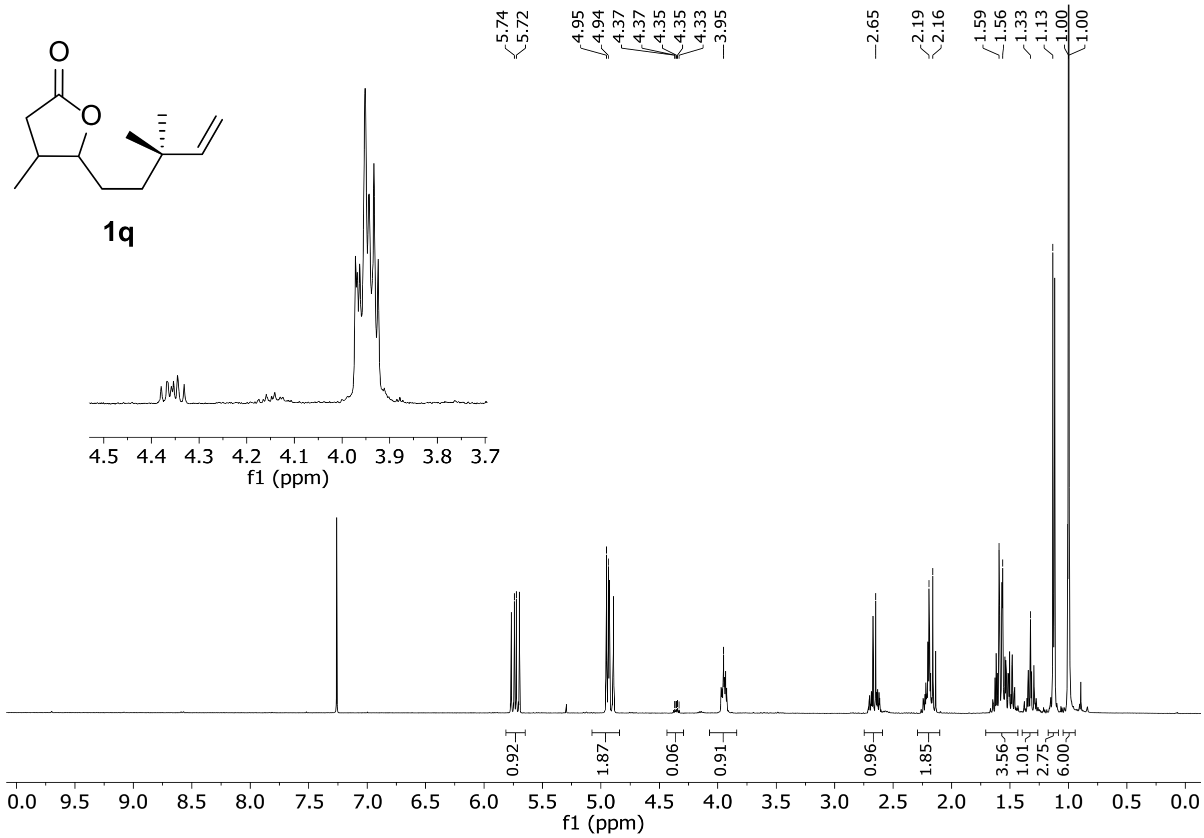

1q

^{13}C{^{1}H}-NMR ($CDCl_3$, 101 MHz)

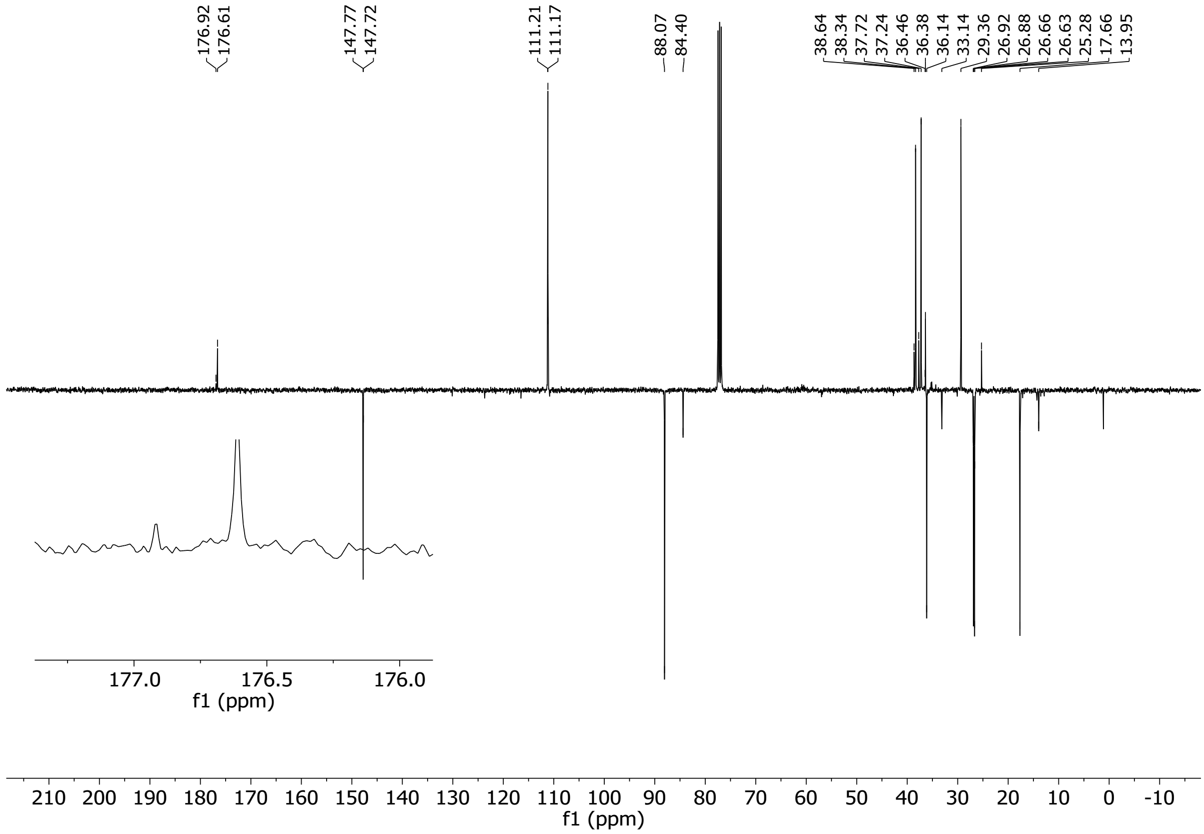

^{1}H-NMR ($CDCl_3$, 400 MHz)

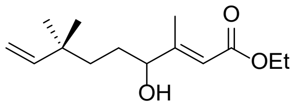

5q

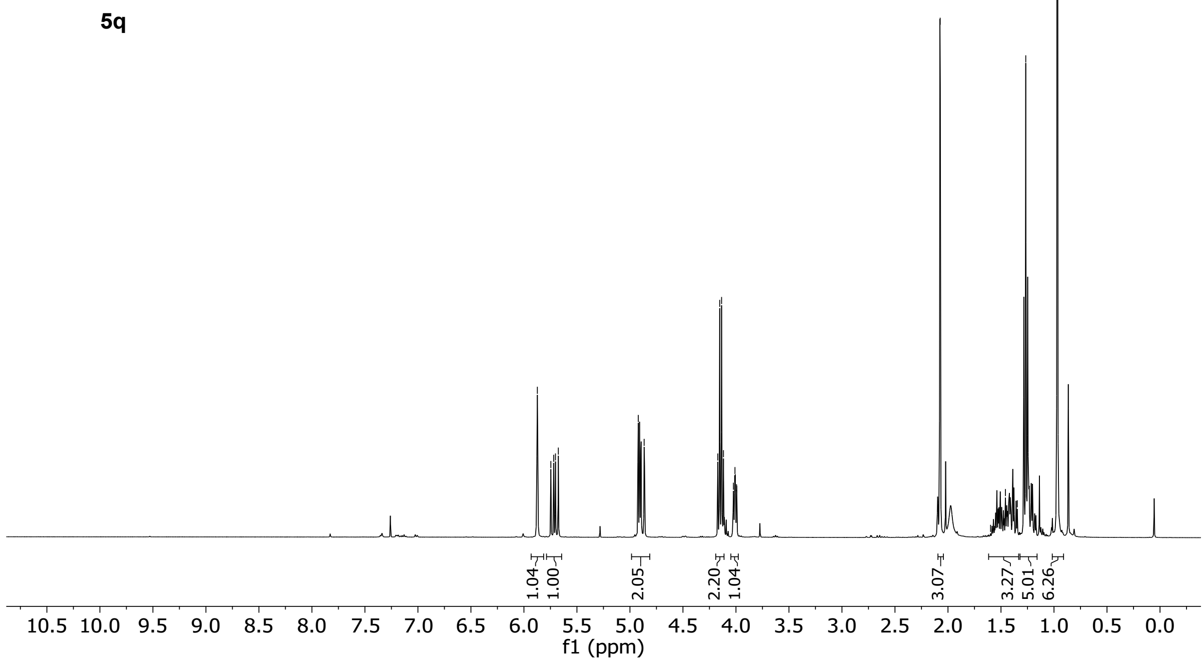

^{13}C{^{1}H}-NMR ($CDCl_3$, 101 MHz)

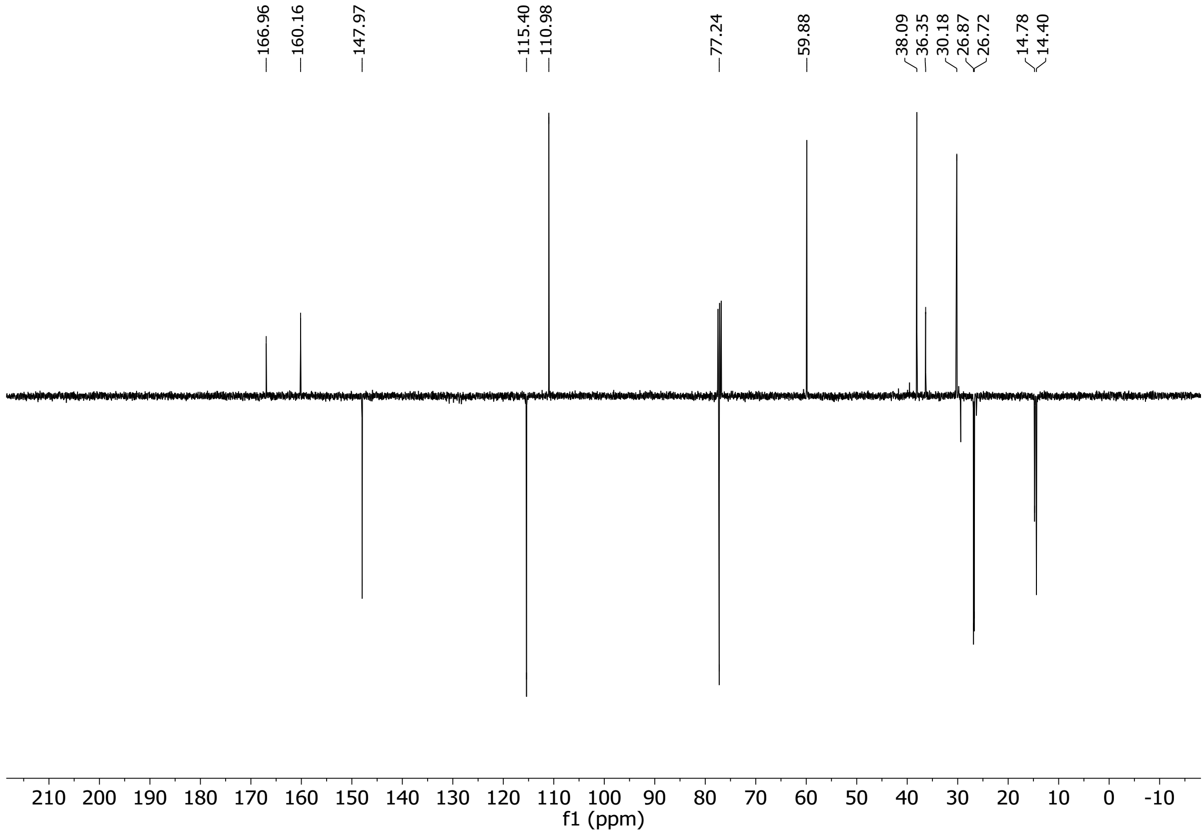

^{1}H-NMR ($CDCl_3$, 400 MHz)

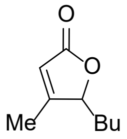

3a

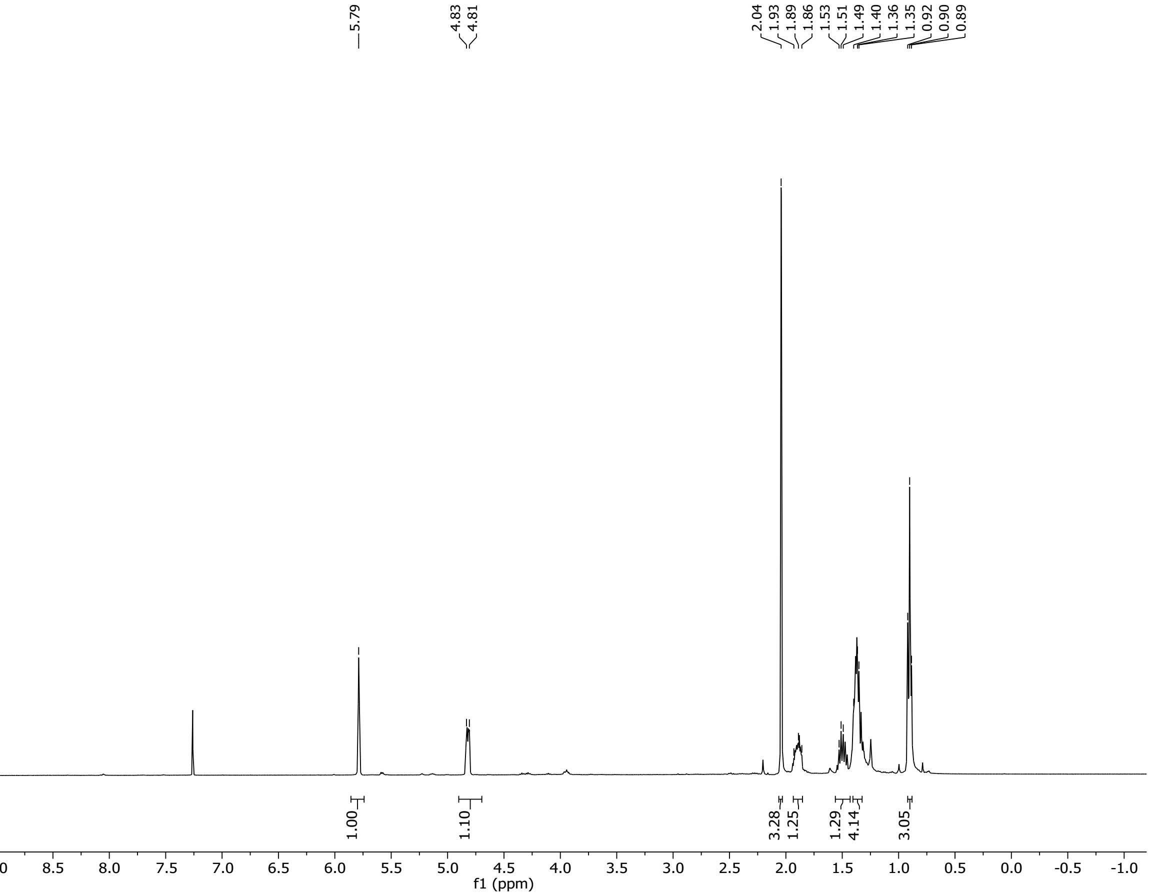

^{13}C{^{1}H}-NMR ($CDCl_3$, 101 MHz)

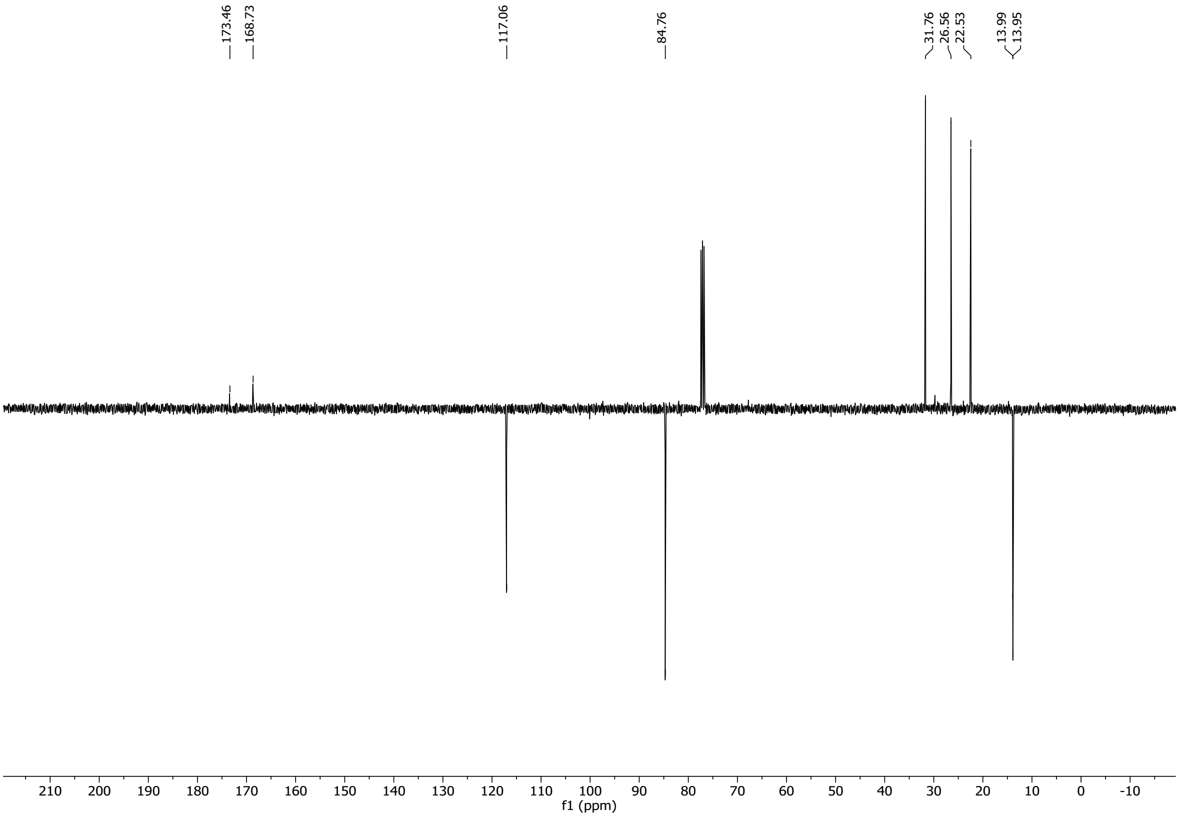

^{1}H-NMR ($CDCl_3$, 400 MHz)

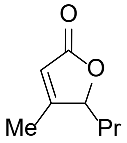

3b

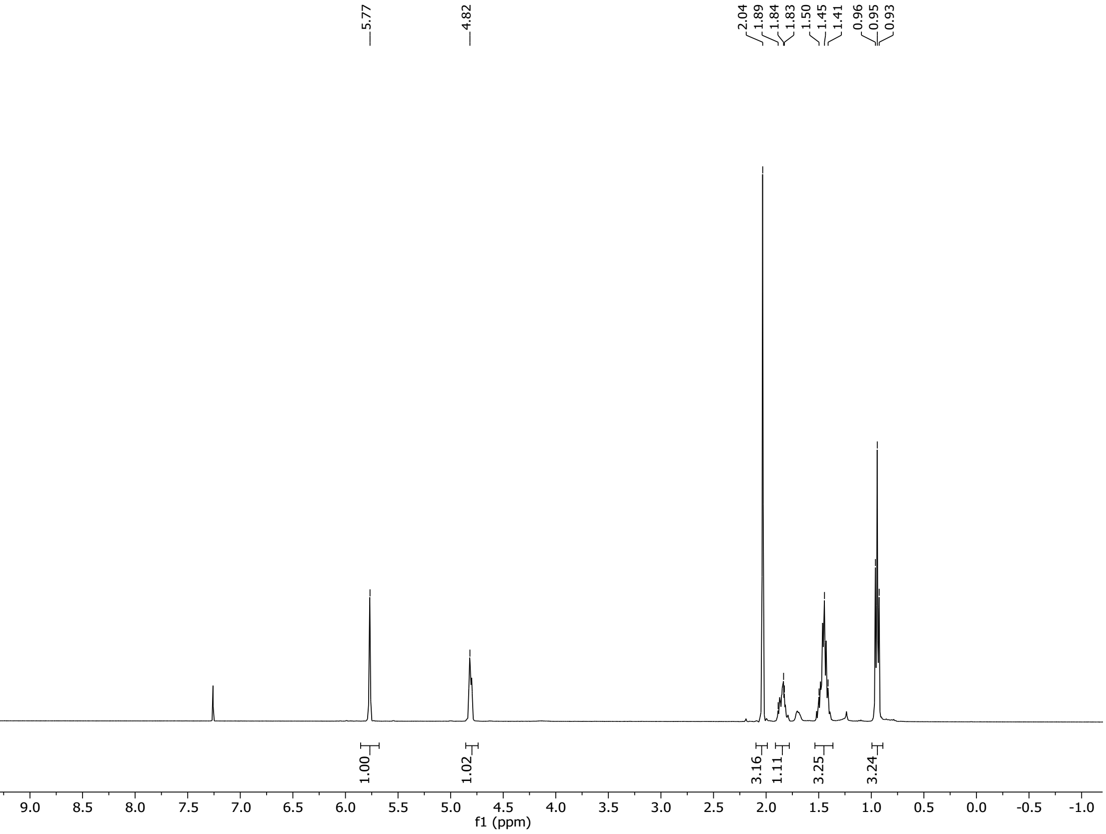

^{13}C{^{1}H}-NMR ($CDCl_3$, 101 MHz)

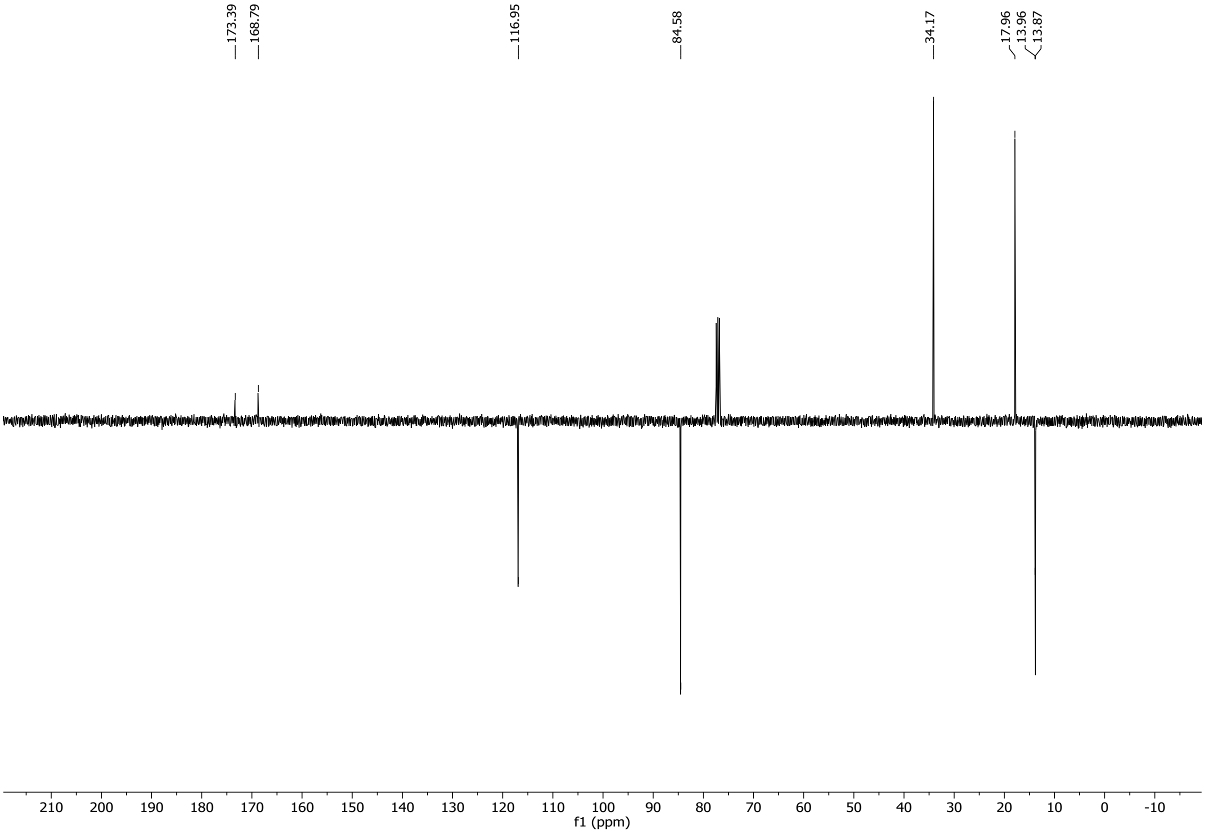

^{1}H-NMR ($CDCl_3$, 400 MHz)

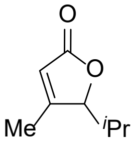

3c

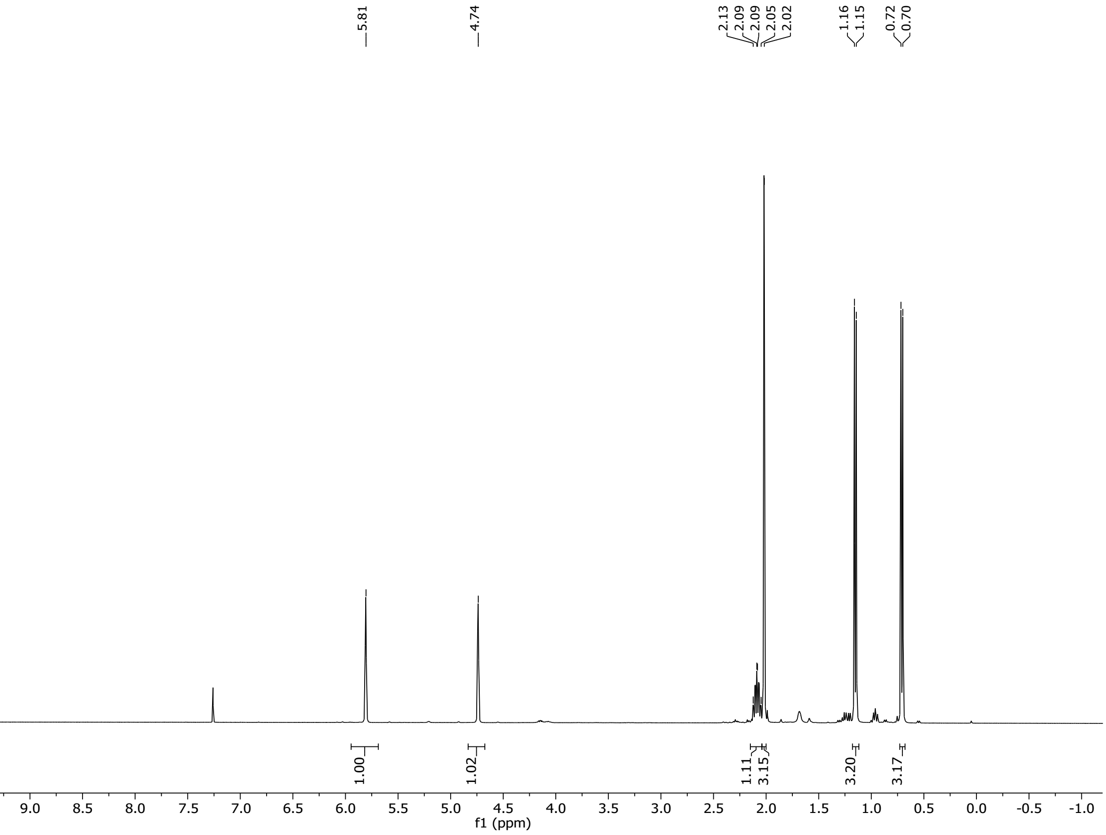

$^{13}C\{^{1}H\}$-NMR ($CDCl_3$, 101 MHz)

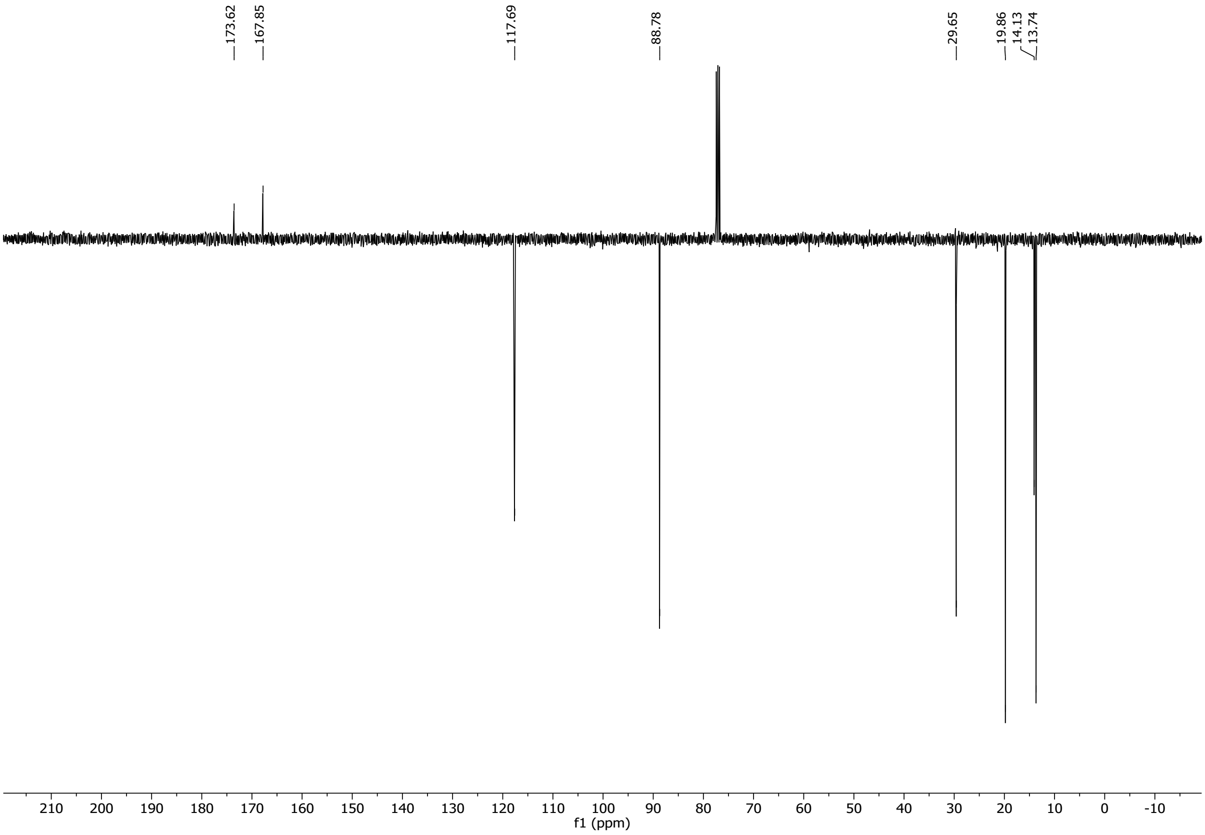

^{1}H-NMR ($CDCl_3$, 400 MHz)

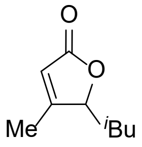

3d

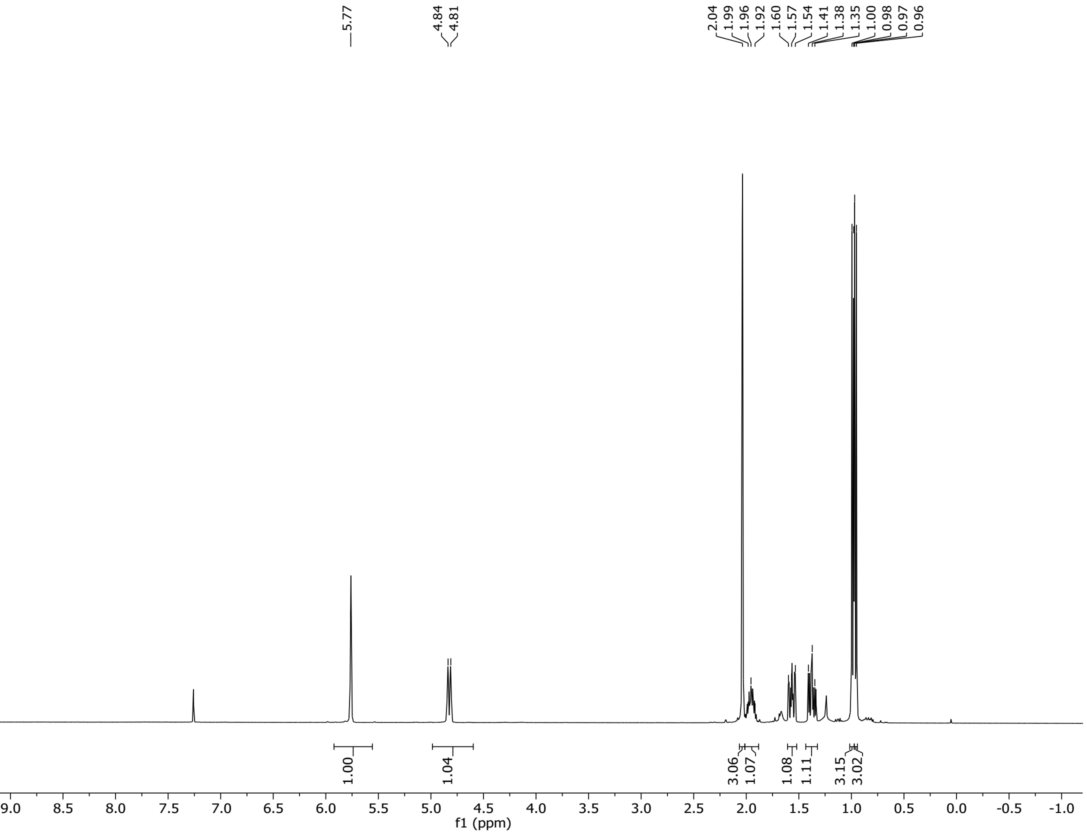

^{13}C{^{1}H}-NMR ($CDCl_3$, 101 MHz)

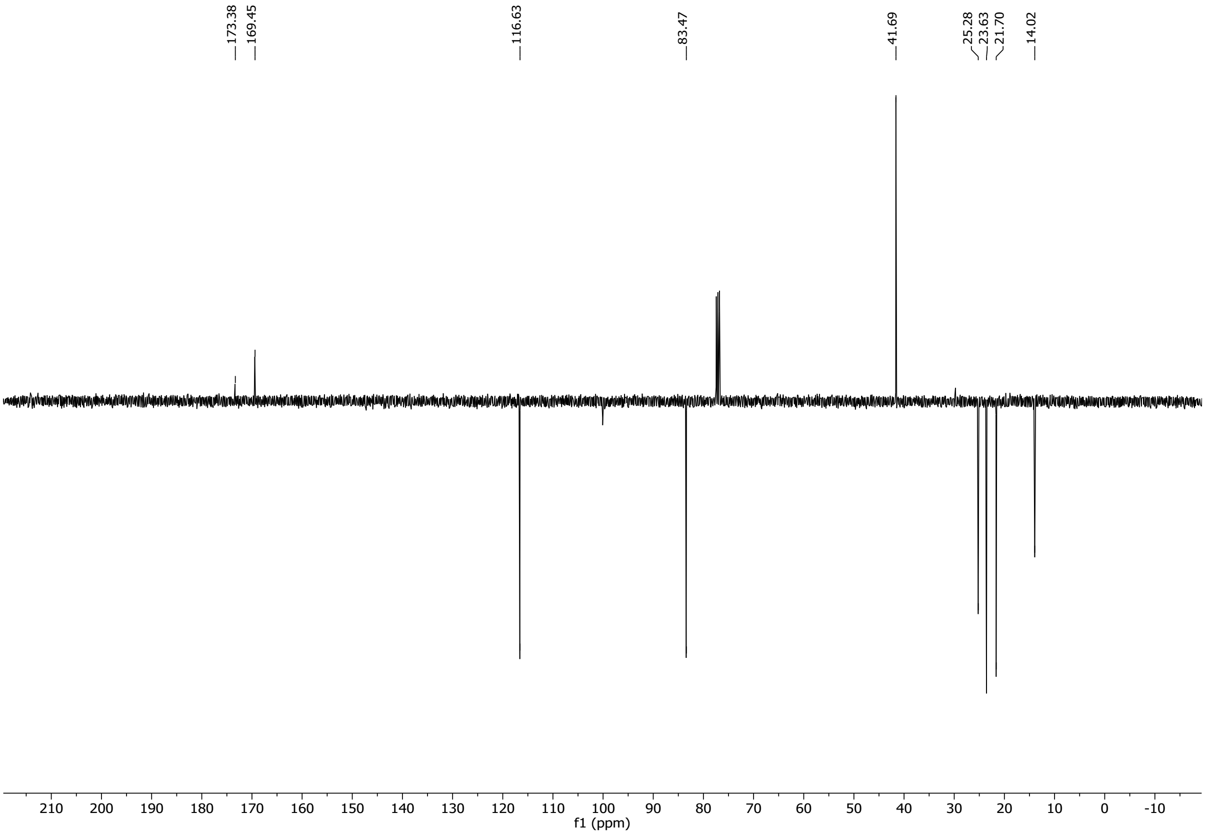

^{1}H-NMR ($CDCl_3$, 400 MHz)

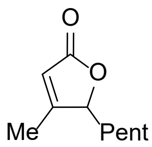

3e

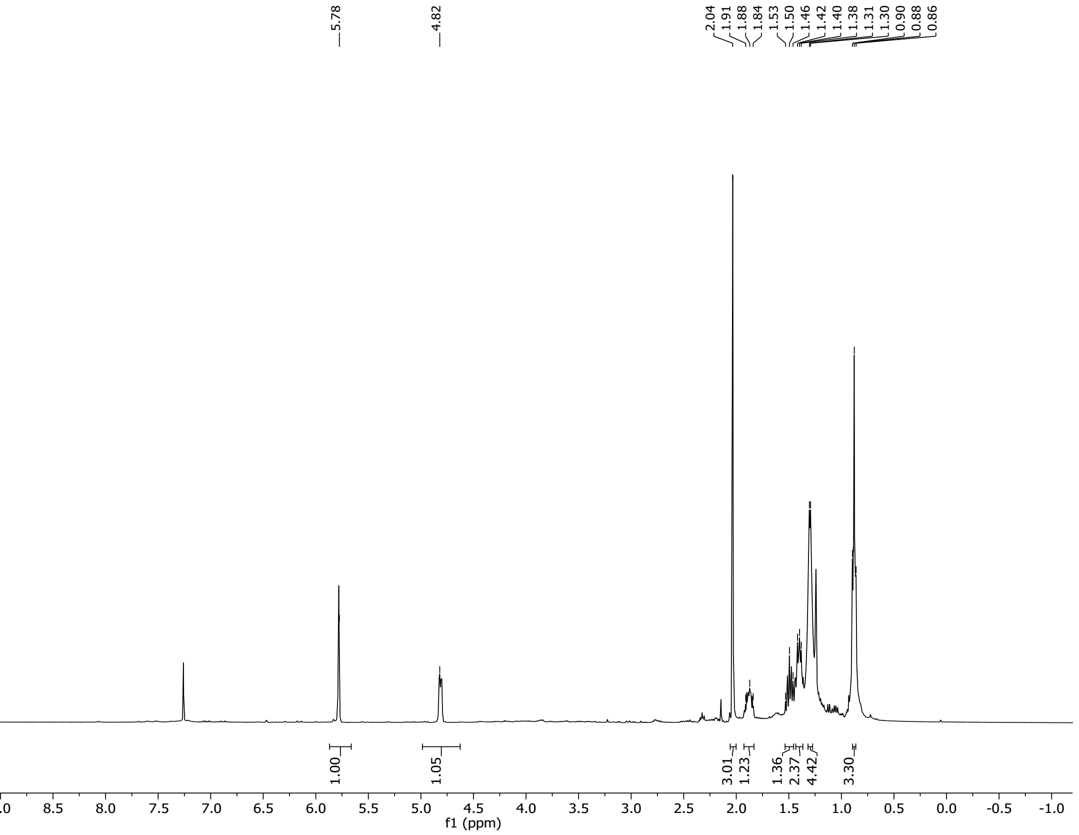

$^{13}C\{^1H\}$-NMR ($CDCl_3$, 101 MHz)

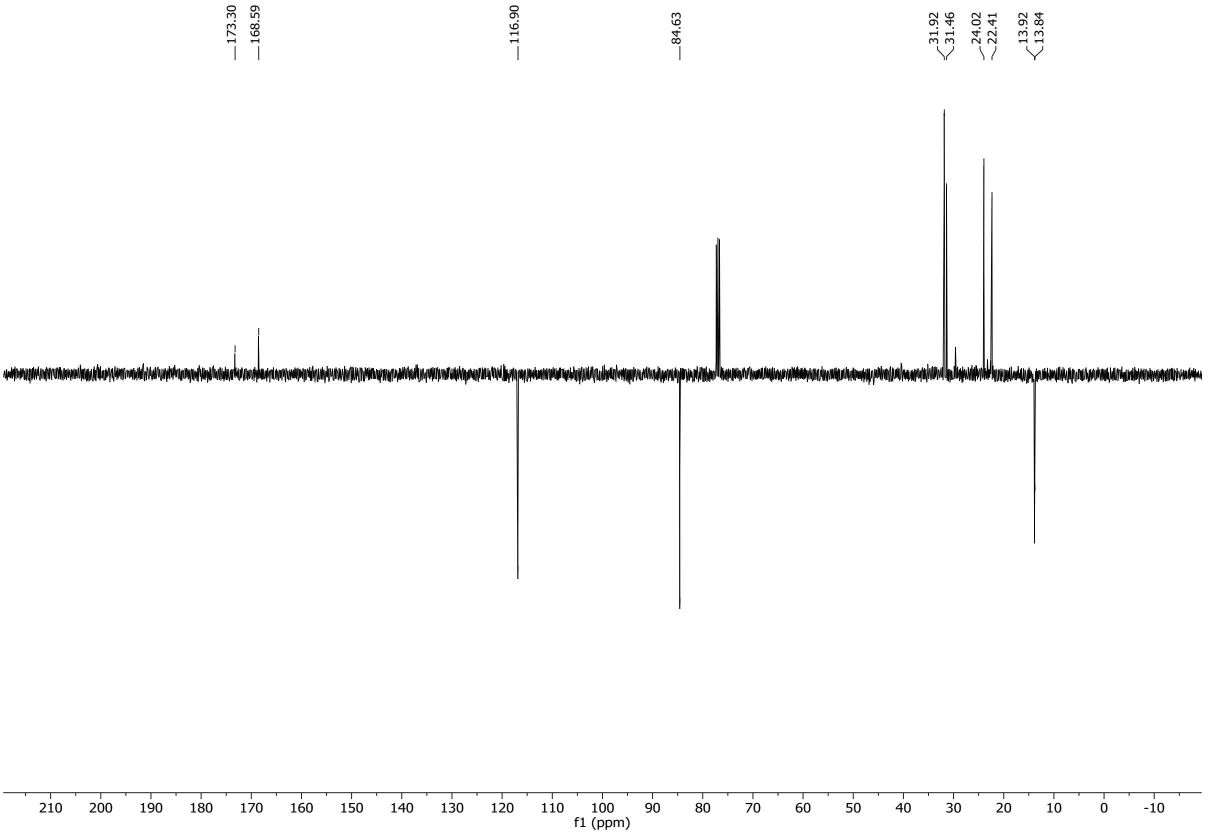

^{1}H-NMR (CDCl$_3$, 400 MHz)

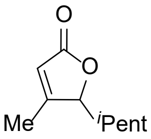

3f

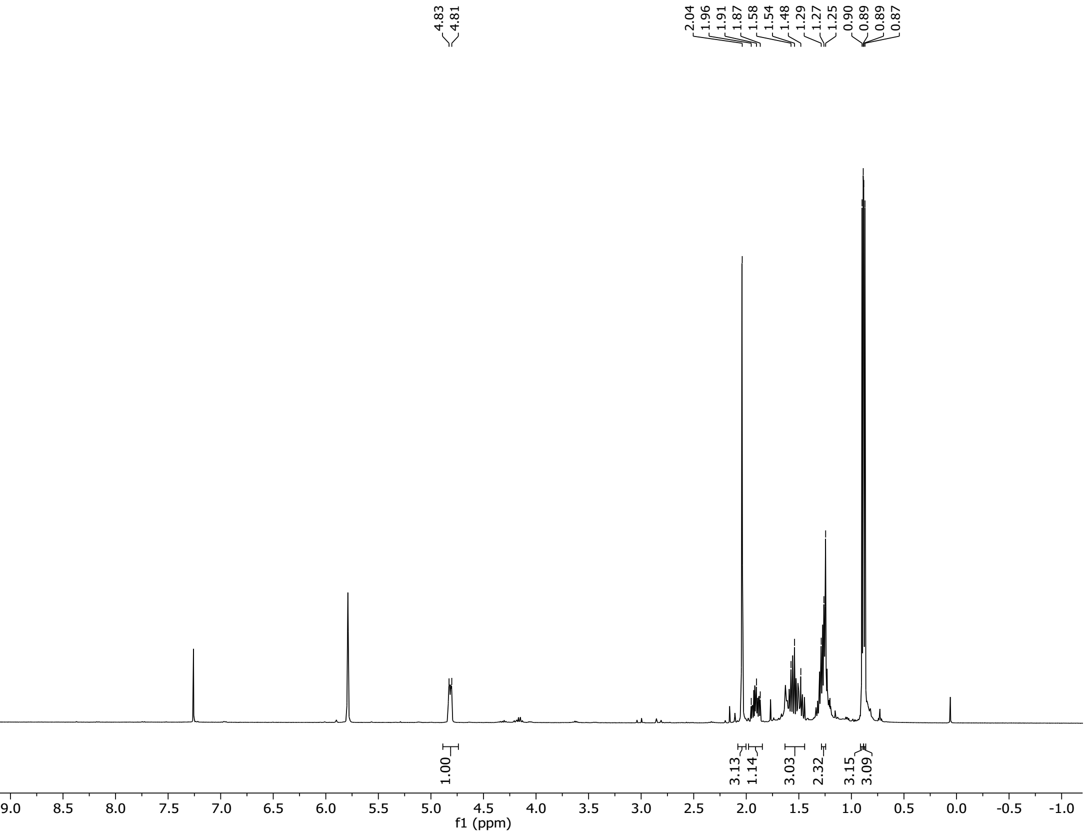

^{13}C{^{1}H}-NMR (CDCl$_3$, 101 MHz)

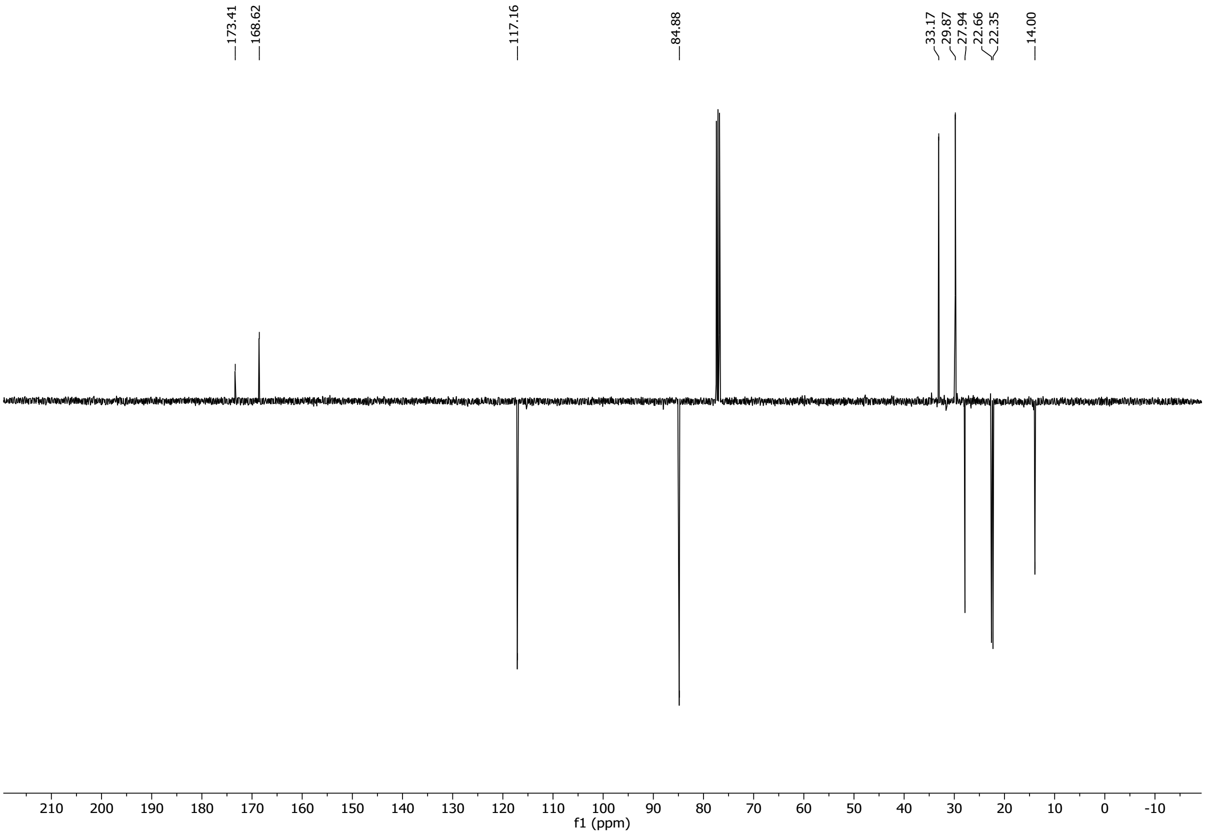

^{1}H-NMR ($CDCl_3$, 400 MHz)

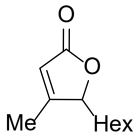

3g

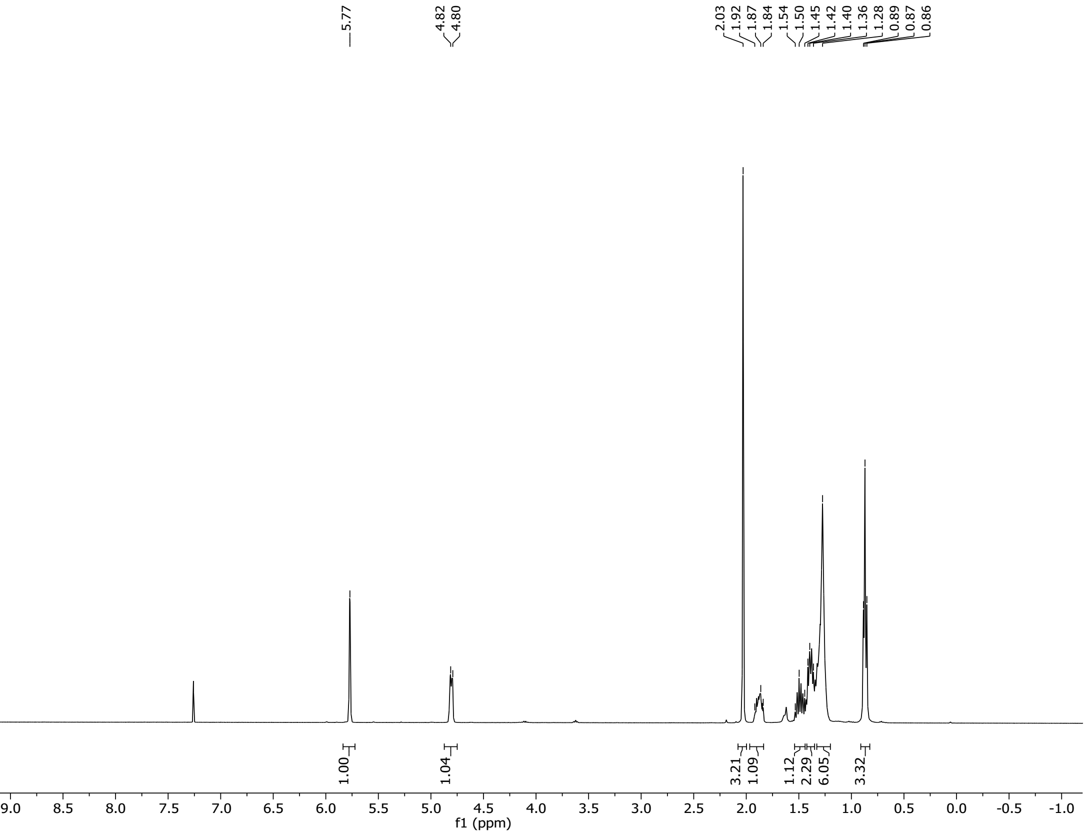

^{13}C{^{1}H}-NMR ($CDCl_3$, 101 MHz)

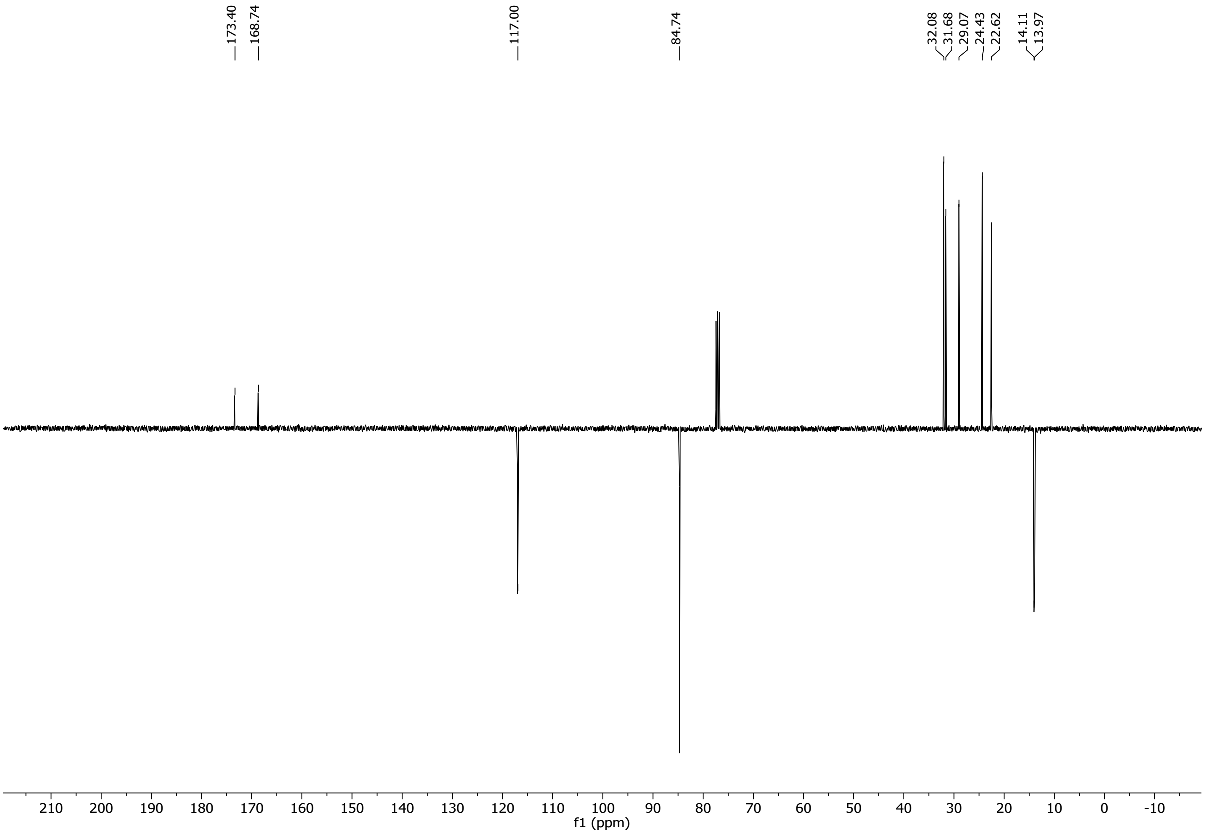

^{1}H-NMR ($CDCl_3$, 400 MHz)

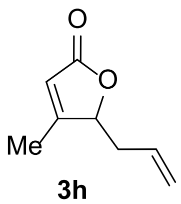

3h

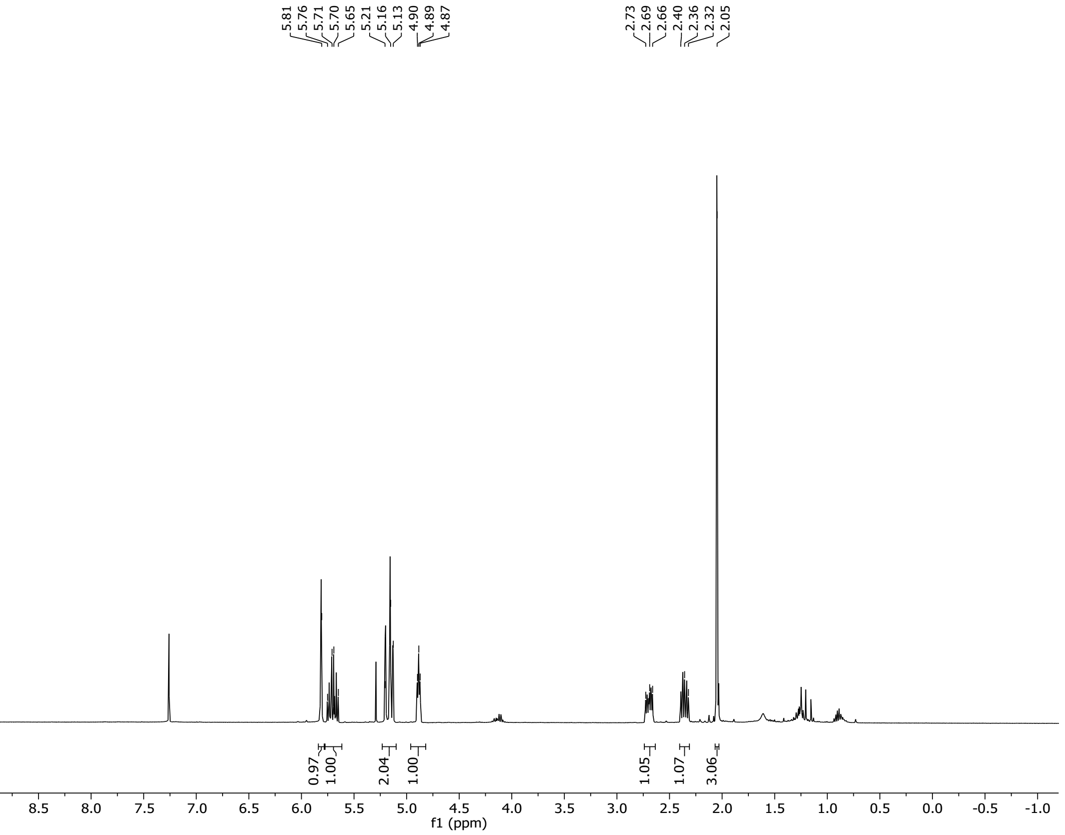

^{13}C{^{1}H}-NMR ($CDCl_3$, 101 MHz)

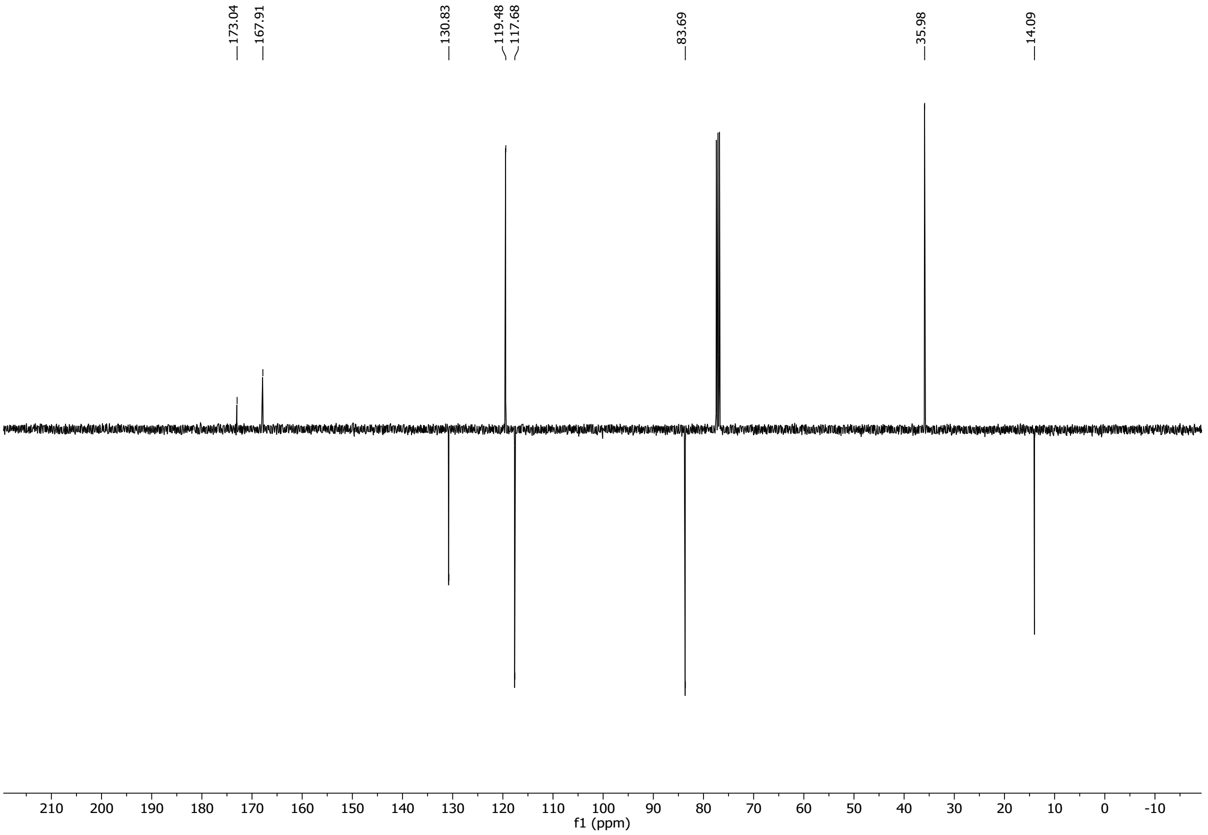

^{1}H-NMR ($CDCl_3$, 400 MHz)

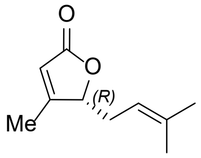

3i

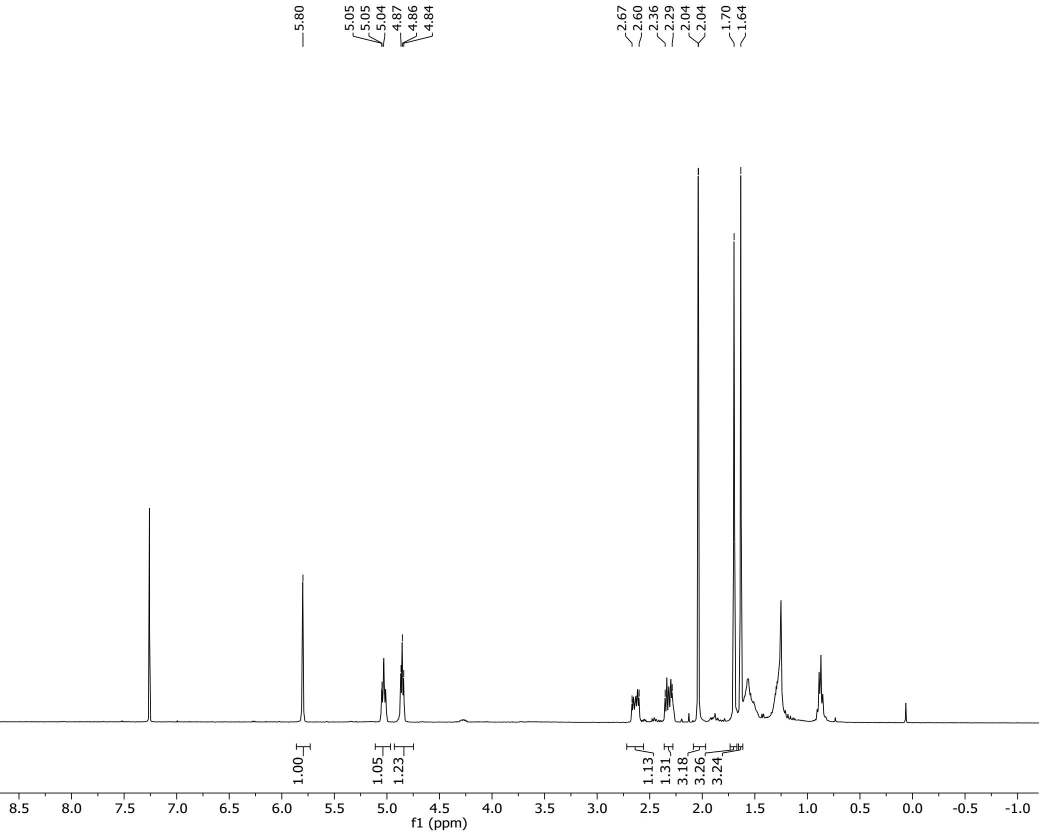

^{13}C{^{1}H}-NMR ($CDCl_3$, 101 MHz)

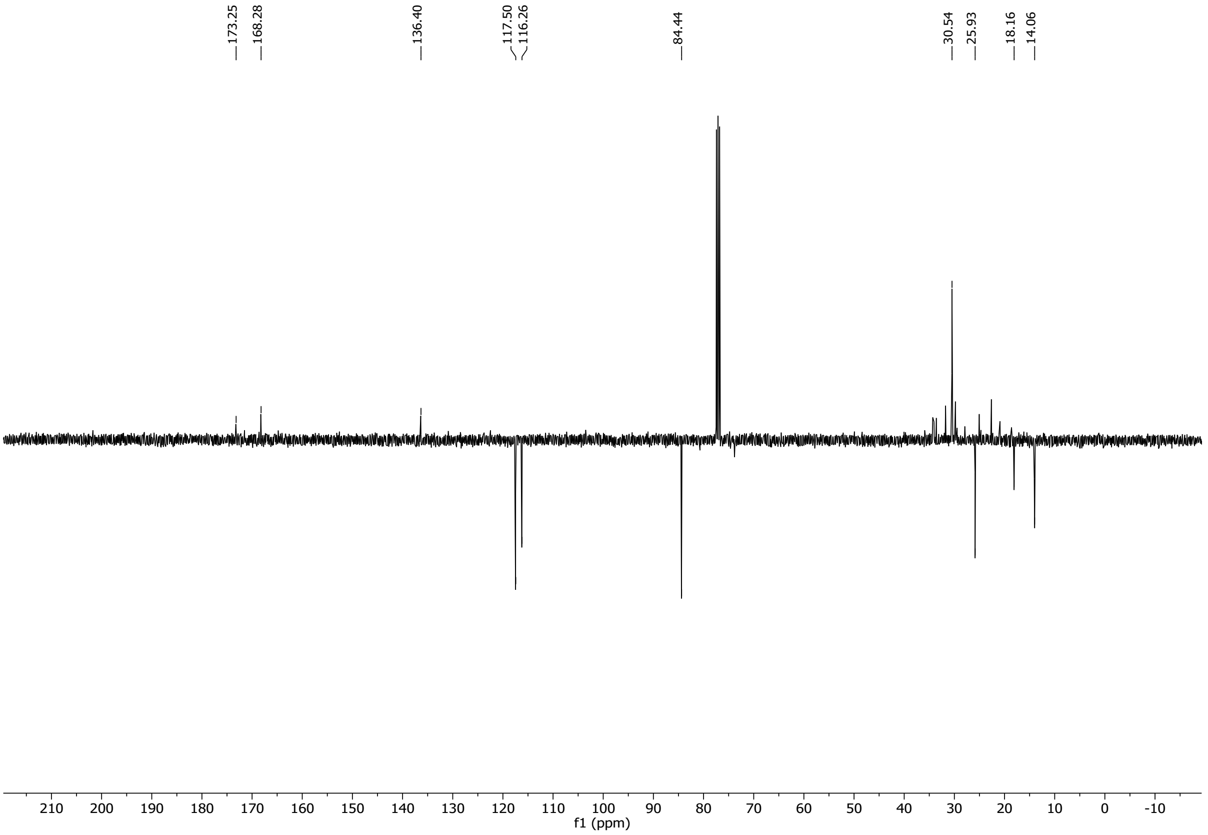

^{1}H-NMR (CDCl$_3$, 400 MHz)

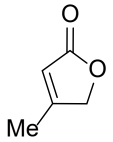

3l

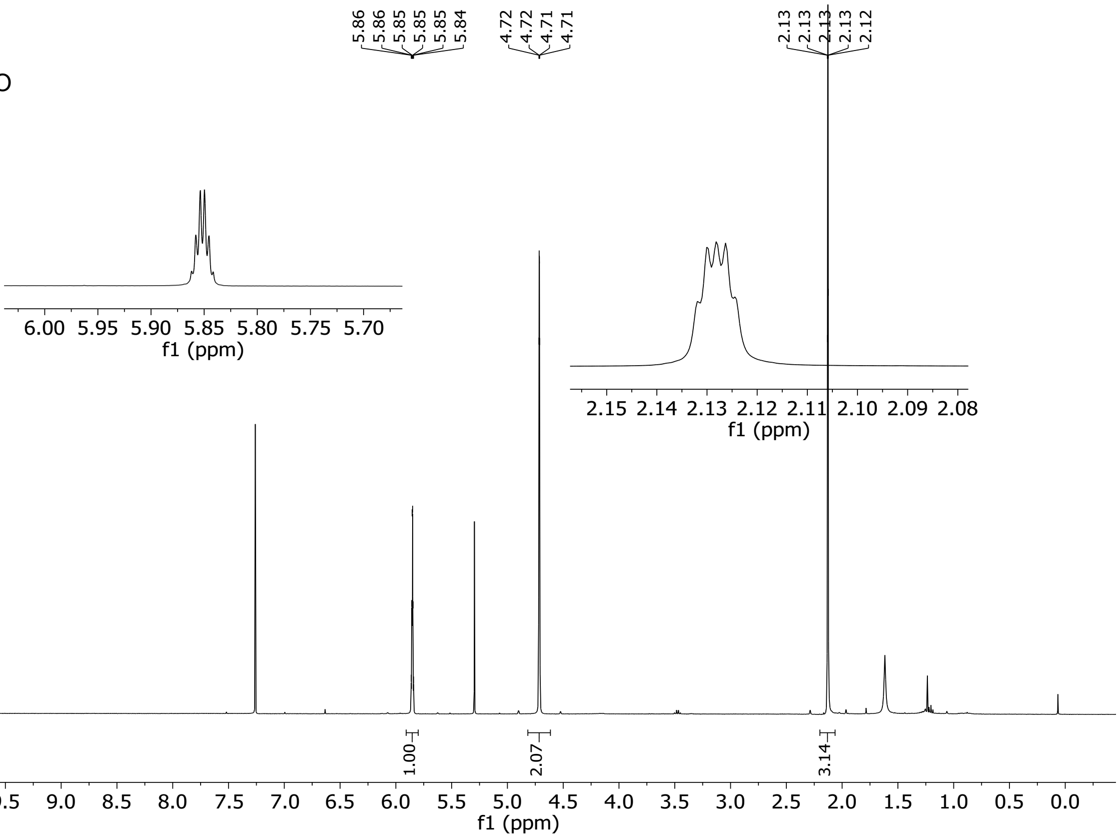

^{13}C{^{1}H}-NMR (CDCl$_3$, 101 MHz)

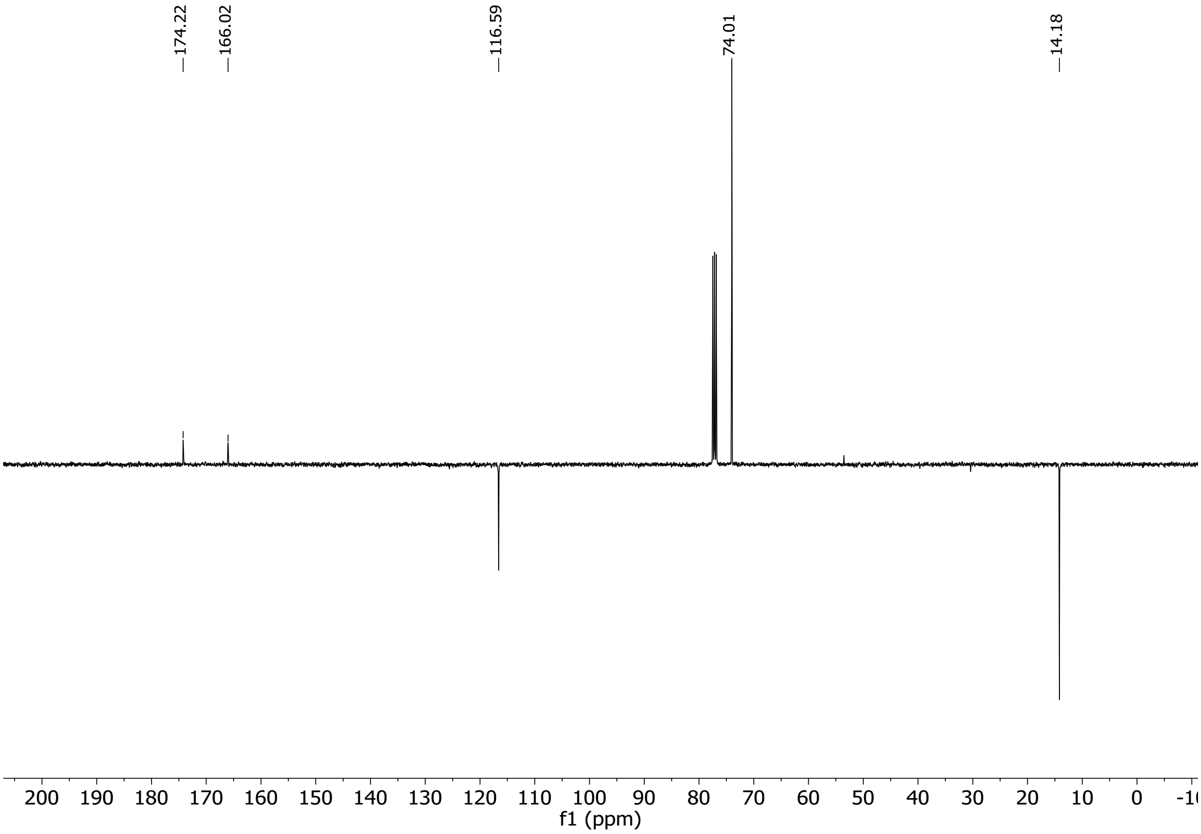

^{1}H-NMR (CDCl$_3$, 400 MHz)

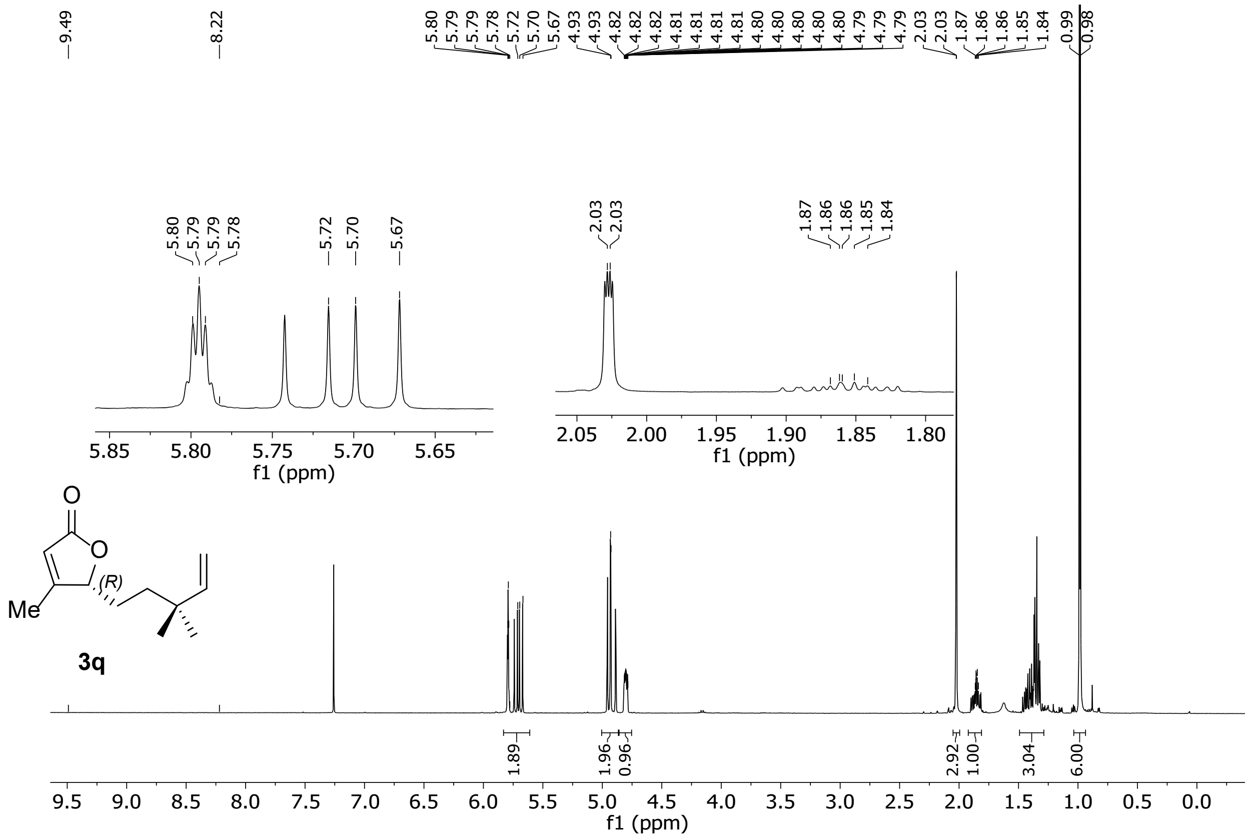

3q

^{13}C{^{1}H}-NMR (CDCl$_3$, 101 MHz)

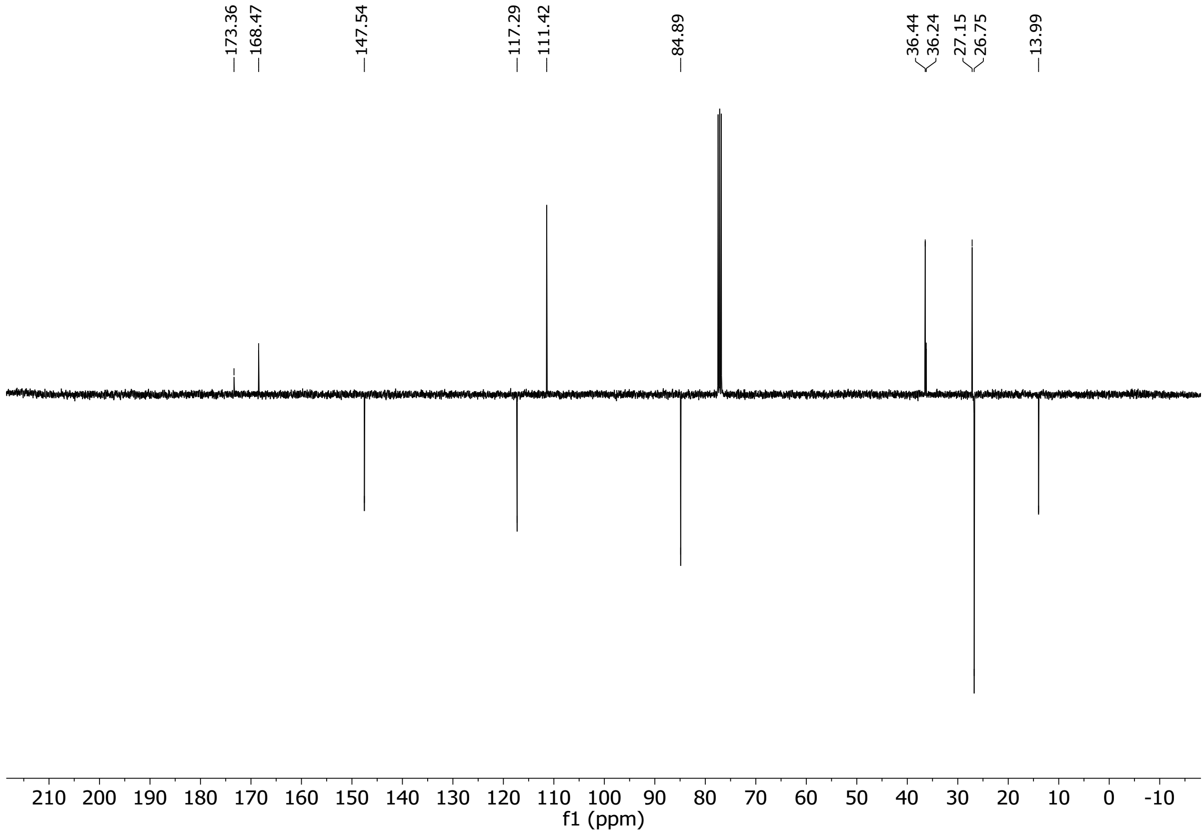

^{1}H-NMR (CDCl$_3$, 400 MHz)

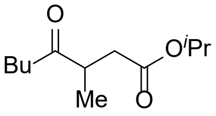

7a

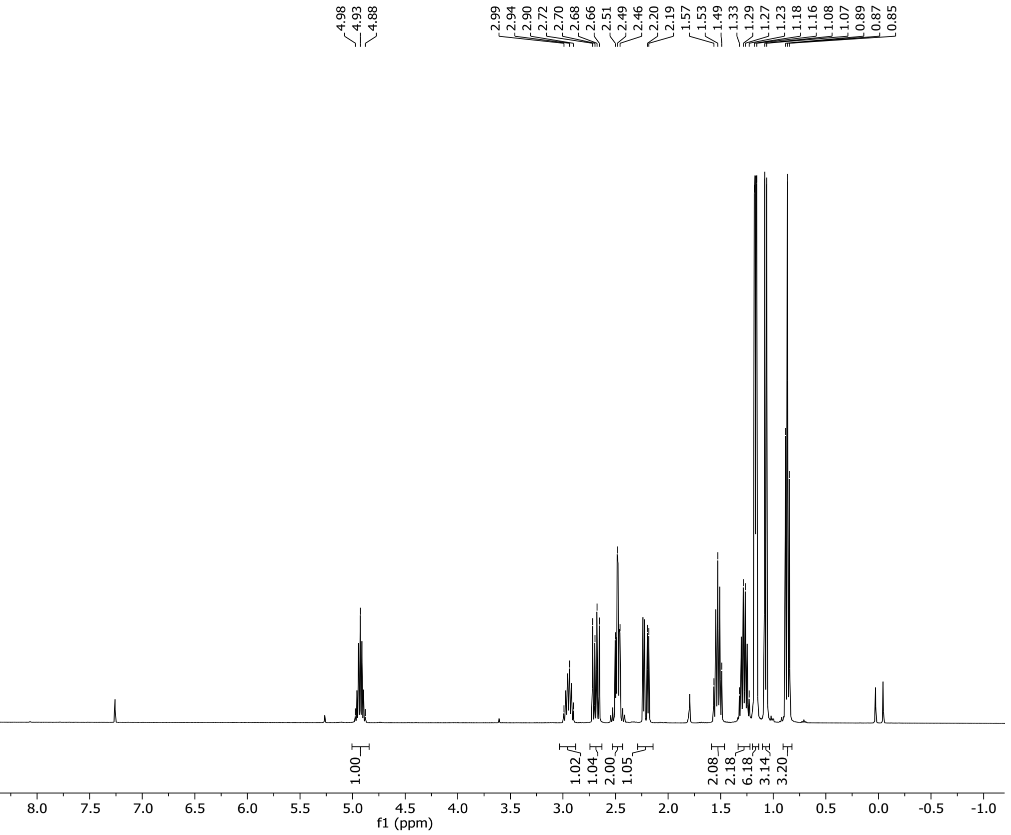

^{13}C{^{1}H}-NMR (CDCl$_3$, 101 MHz)

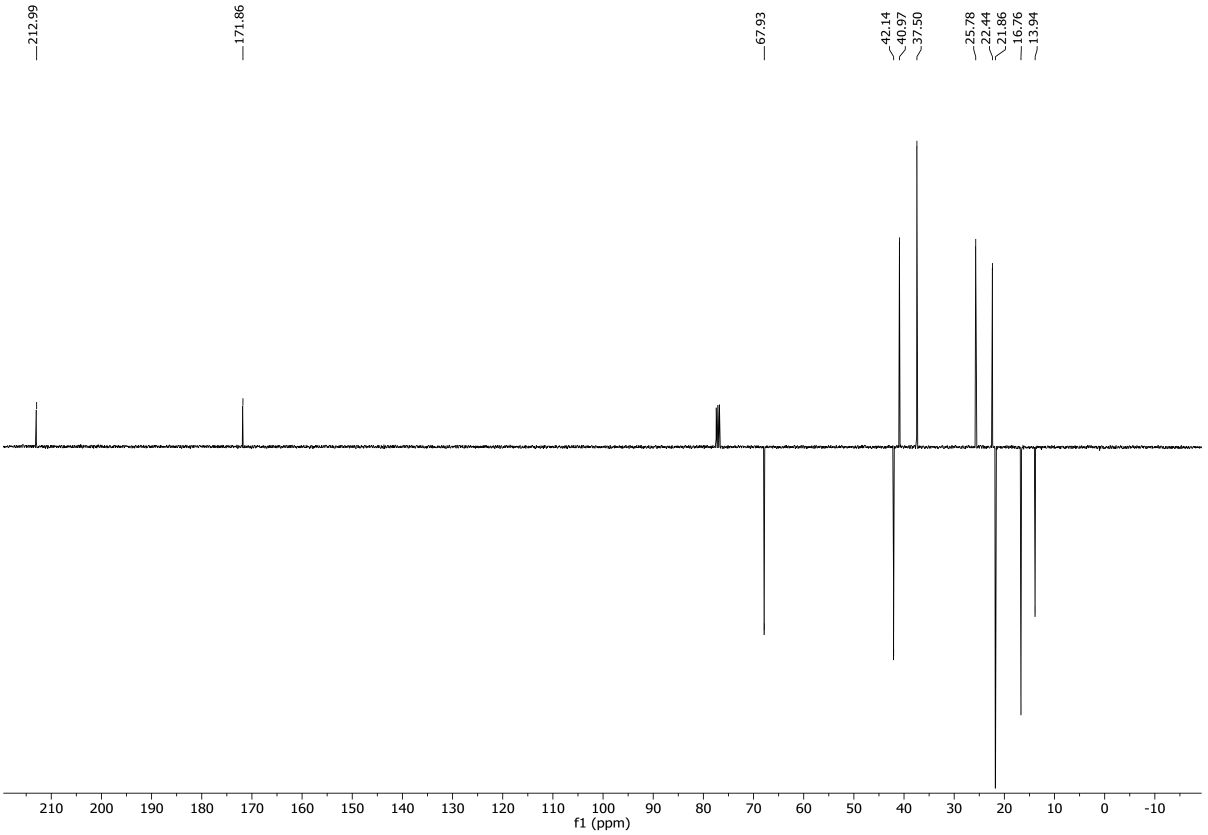

^{1}H-NMR ($CDCl_3$, 400 MHz)

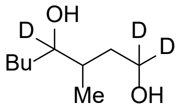

2a-1,1,4-d_3

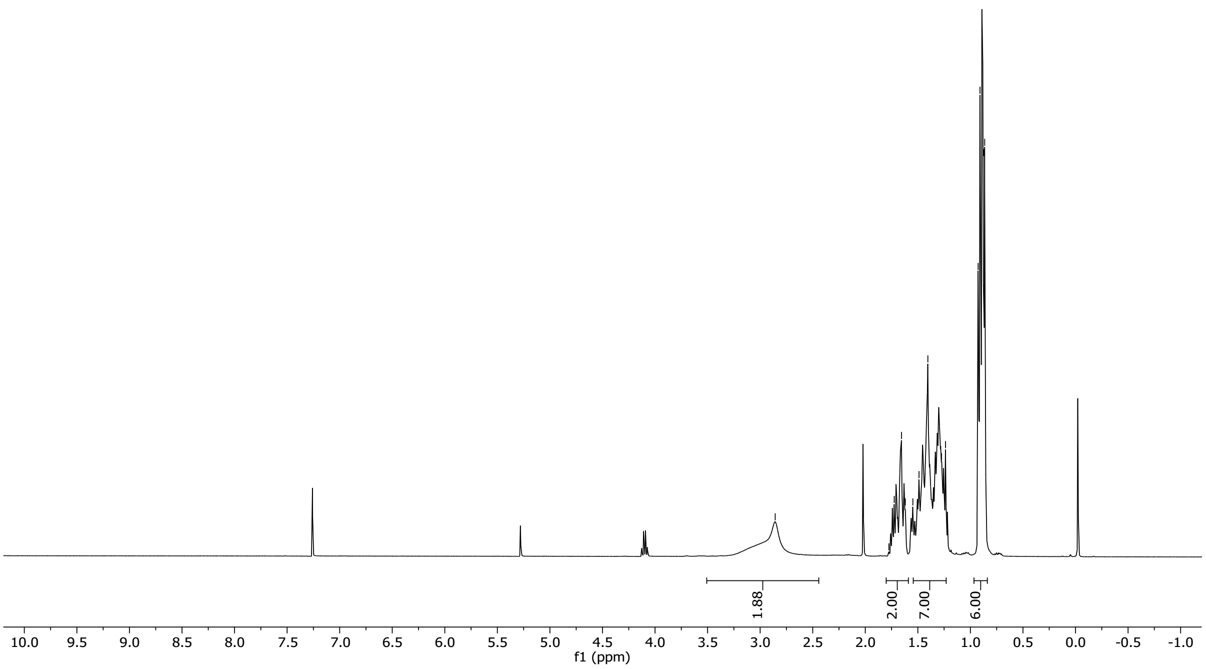

$^{13}C\{^{1}H\}$-NMR ($CDCl_3$, 101 MHz)

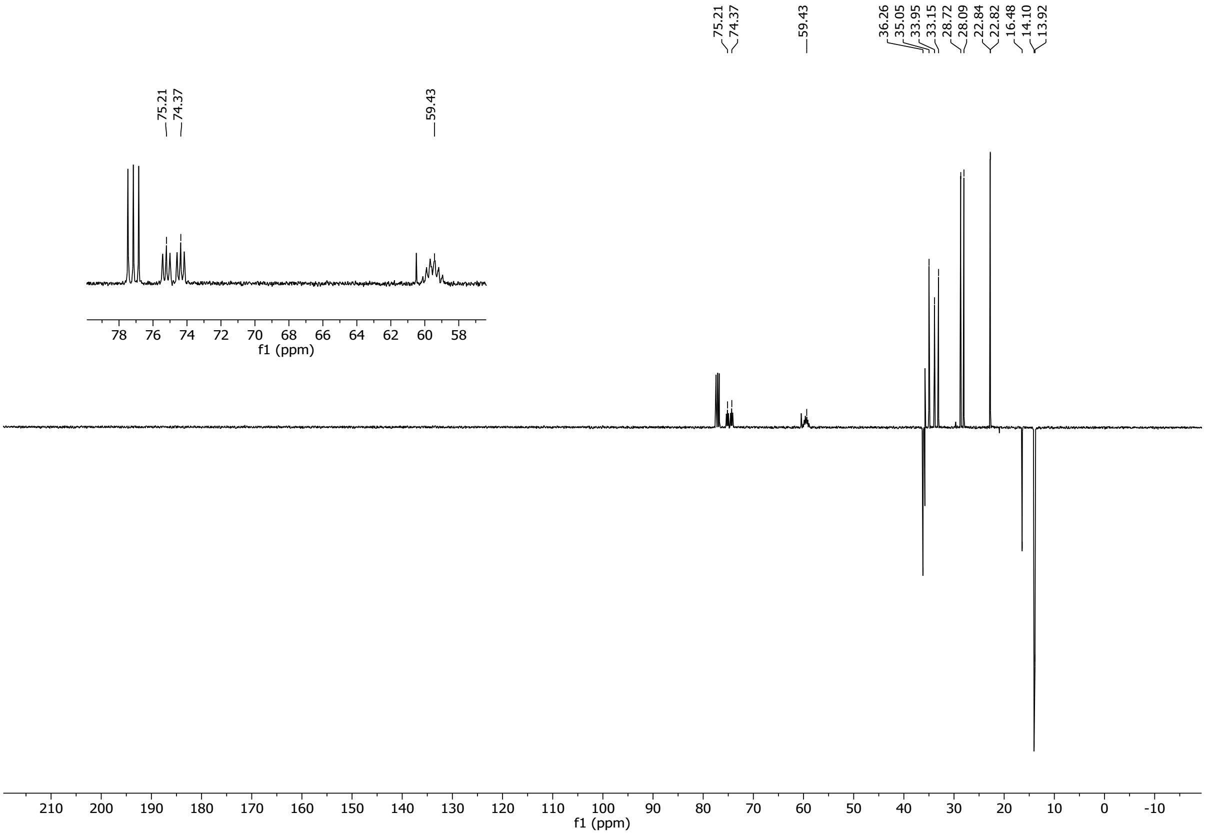

^{2}H-NMR ($CHCl_3$, 61.4 MHz)

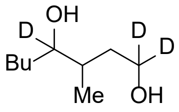

2a-1,1,4-d_3

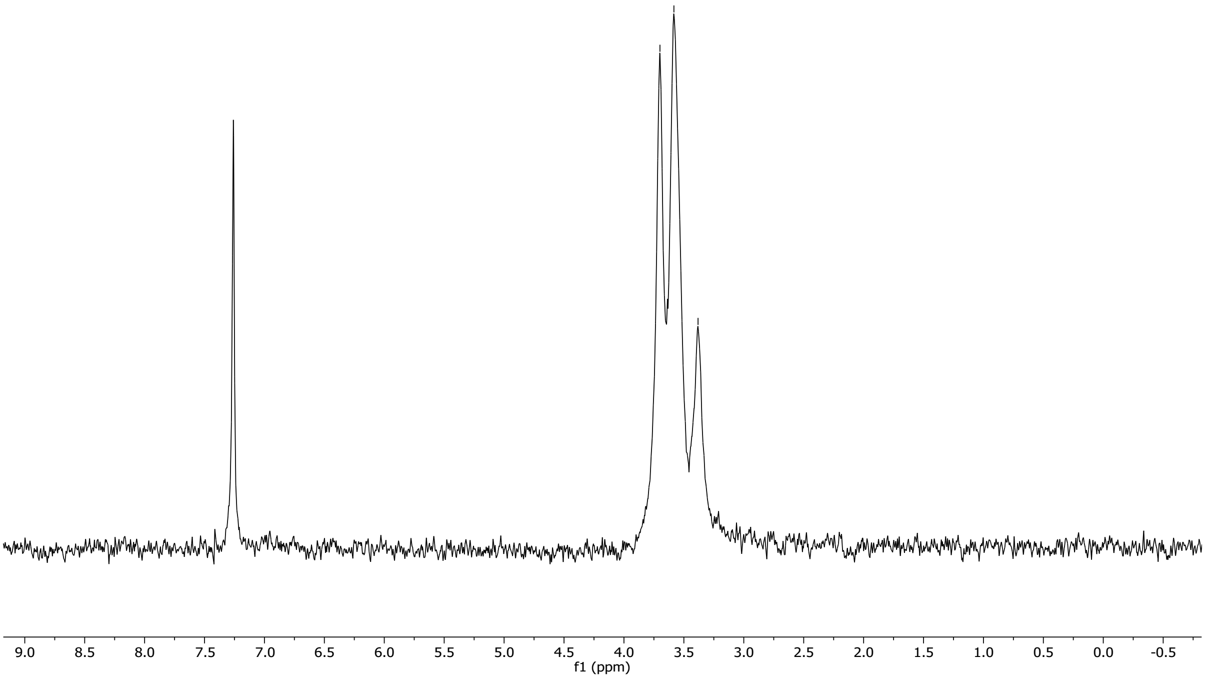

^{1}H-NMR (CDCl$_3$, 400 MHz)

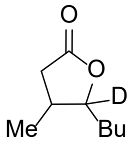

1a-5-*d*

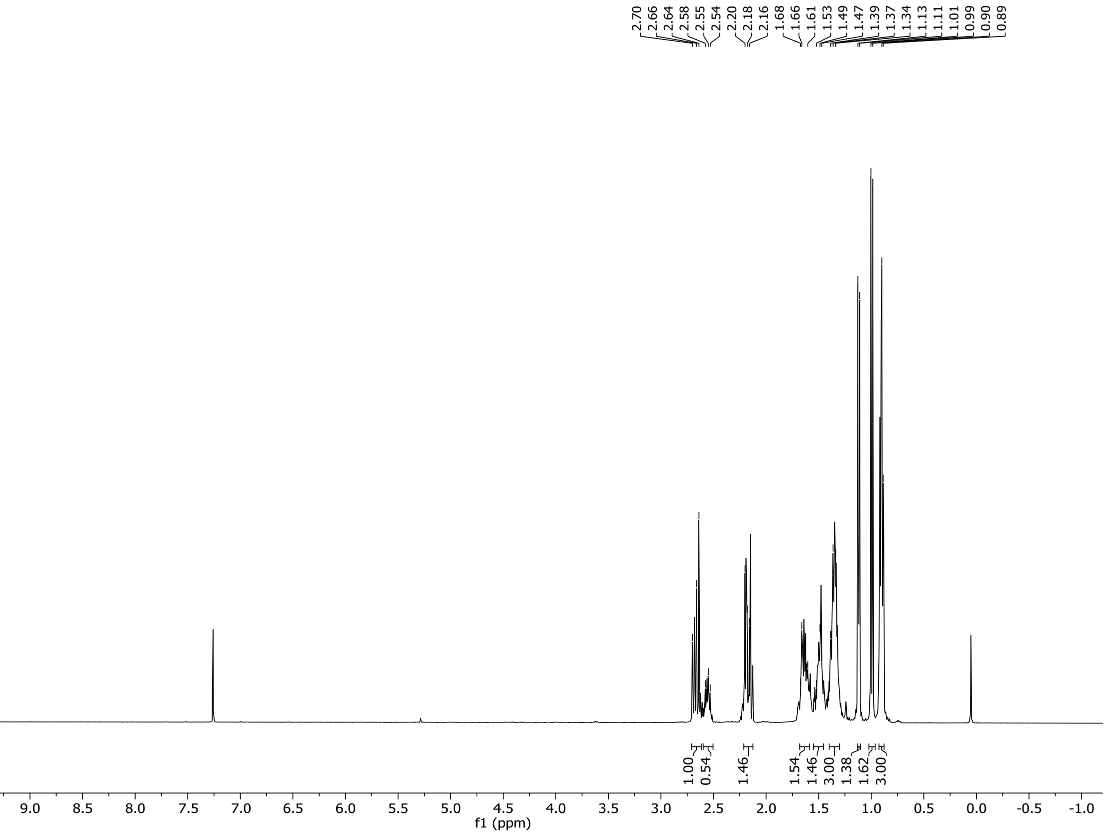

^{13}C{^{1}H}-NMR (CDCl$_3$, 101 MHz)

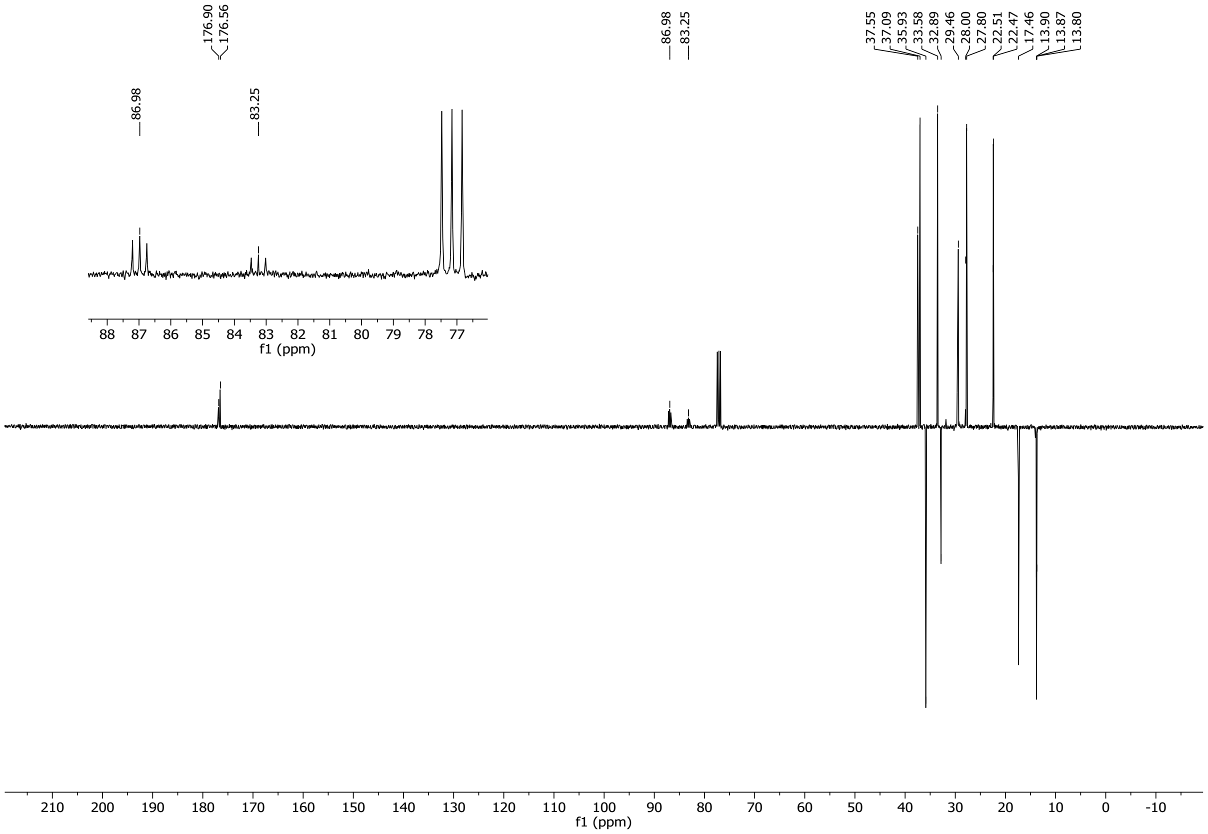

^{2}H-NMR ($CHCl_3$, 61.4 MHz)

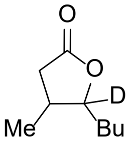

1a-5-*d*

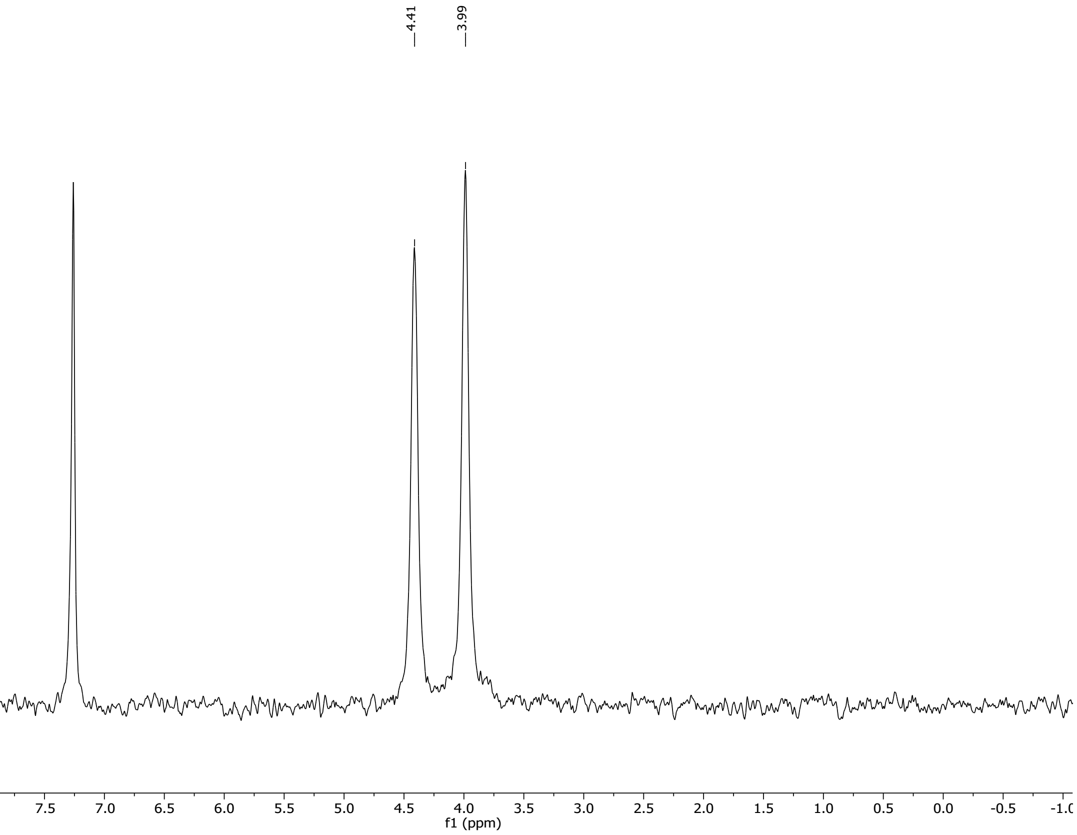

^{1}H-NMR ($CDCl_3$, 400 MHz)

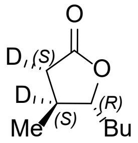

trans-**2a**-3,4-d_2

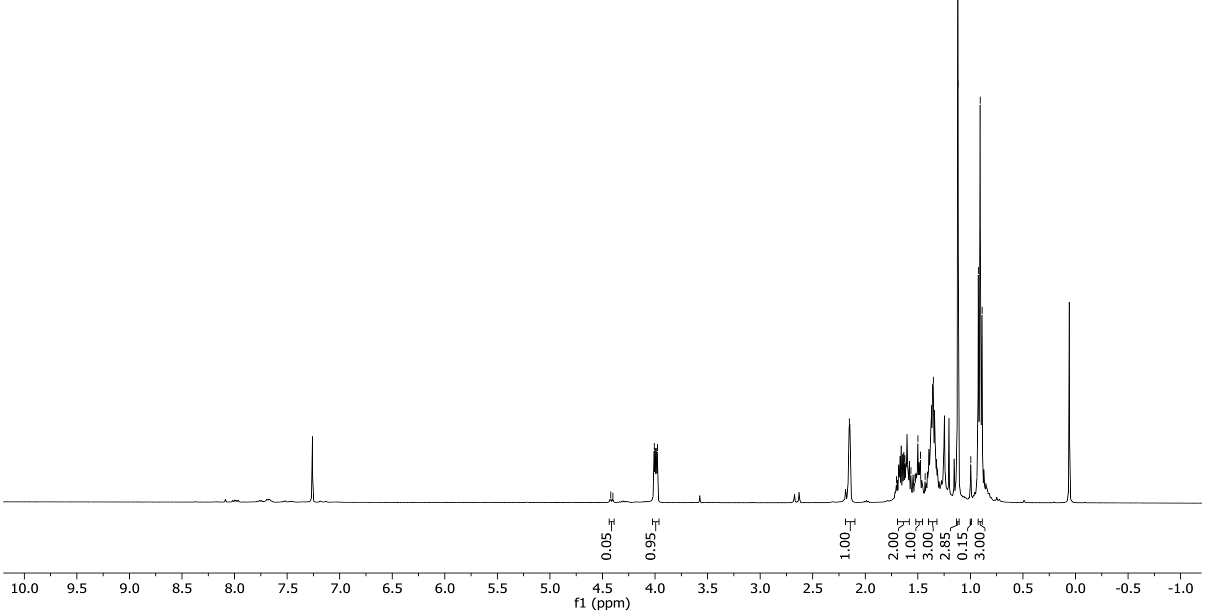

^{13}C{^{1}H}-NMR ($CDCl_3$, 101 MHz)

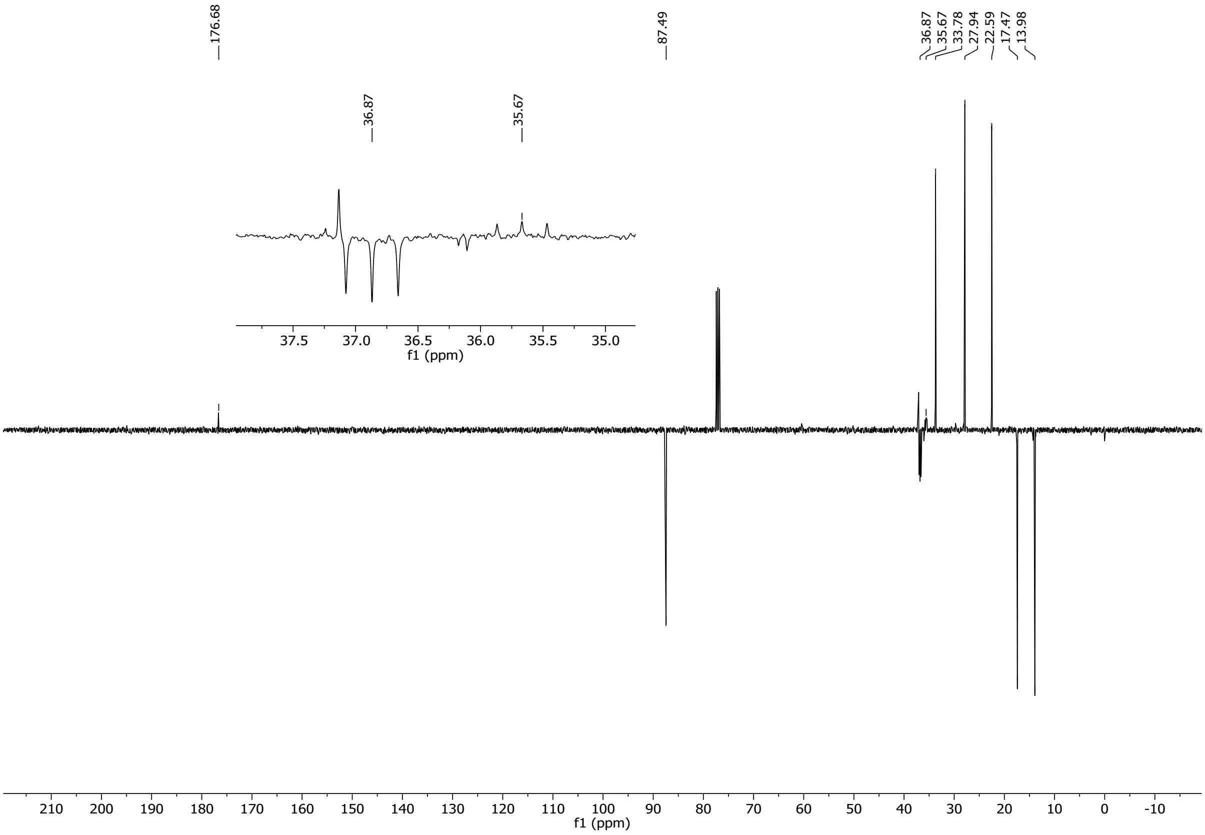

^{2}H-NMR ($CHCl_3$, 61.4 MHz)

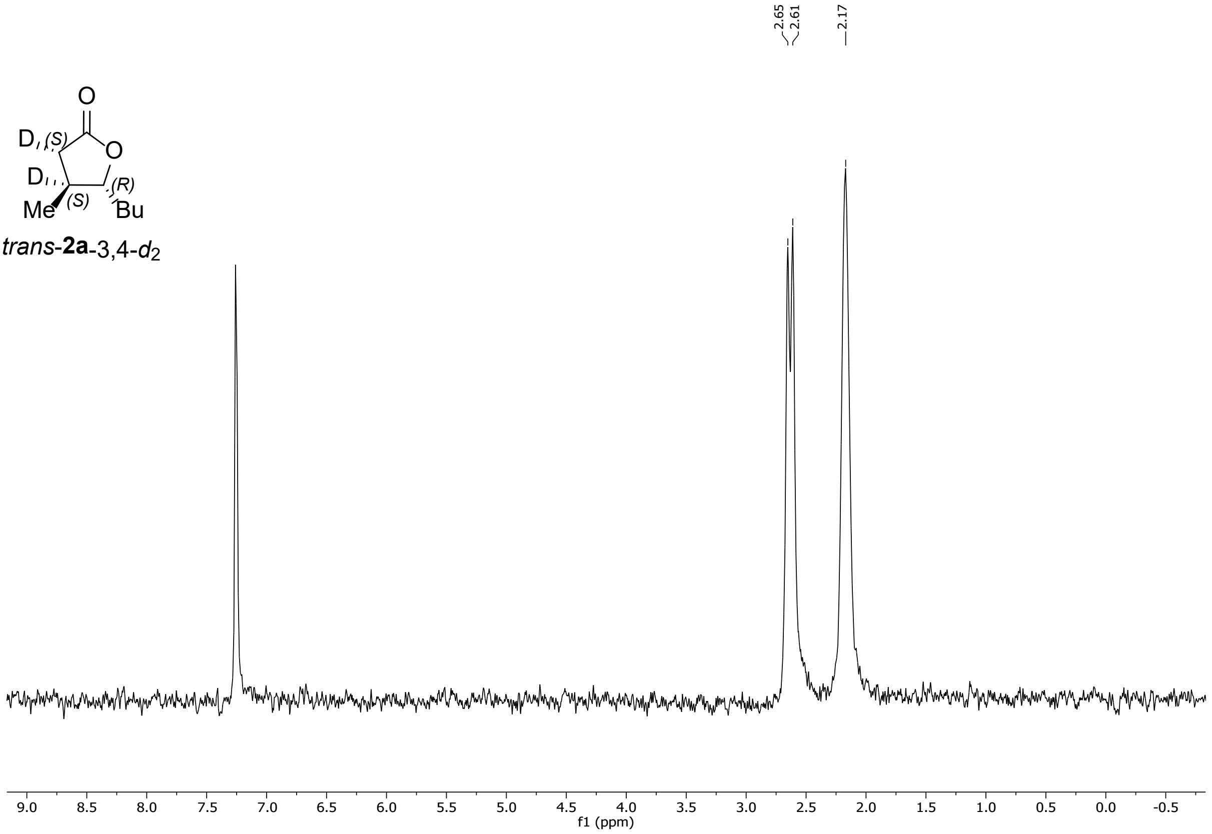

trans-**2a**-3,4-d_2

^{1}H-NMR ($CDCl_3$, 400 MHz)

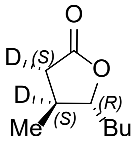

cis-**2a**-3,4-d_2

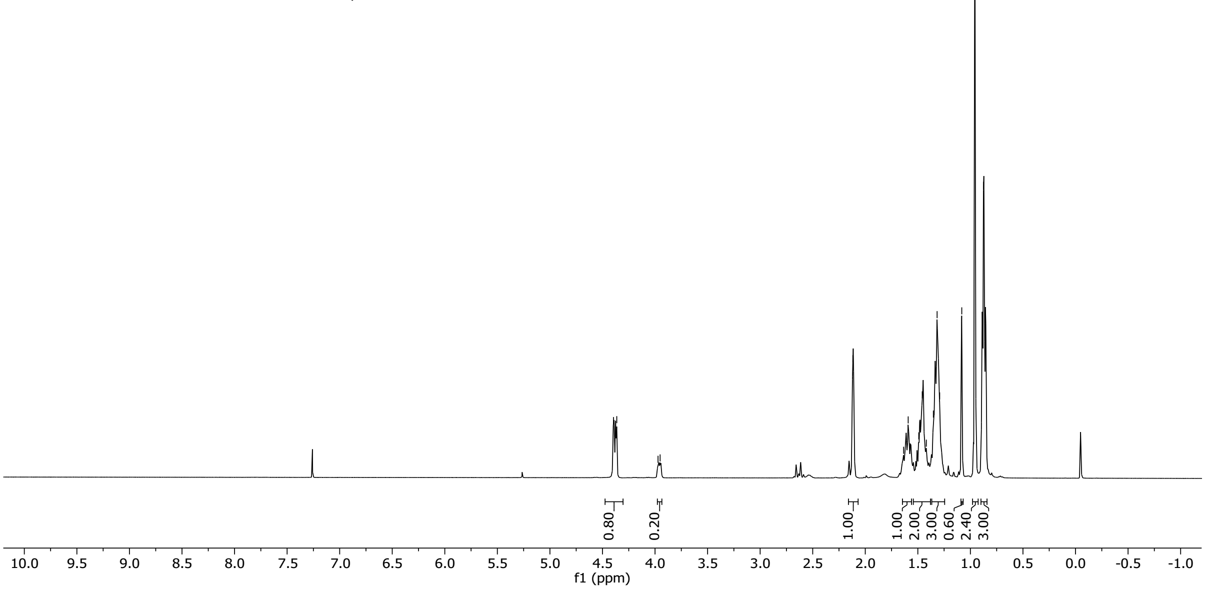

^{13}C{^{1}H}-NMR ($CDCl_3$, 101 MHz)

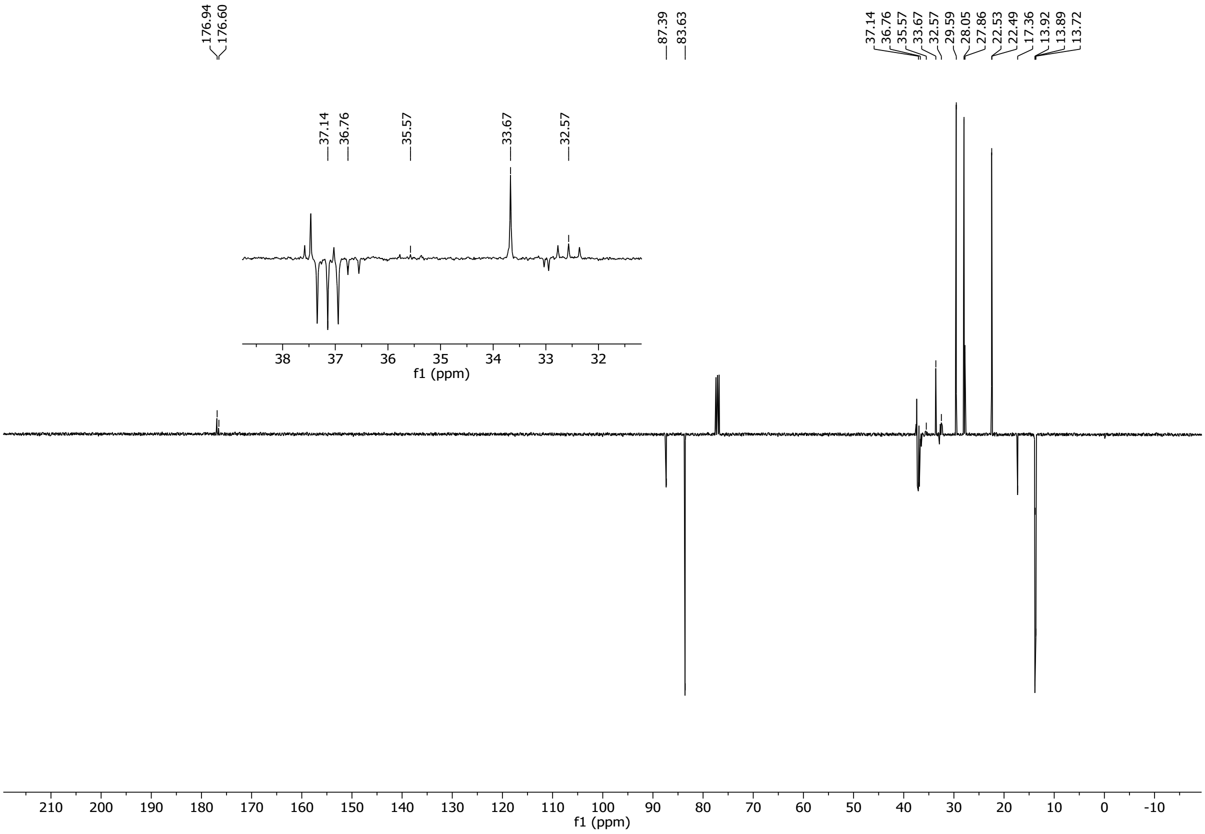

^2H-NMR ($CHCl_3$, 61.4 MHz)

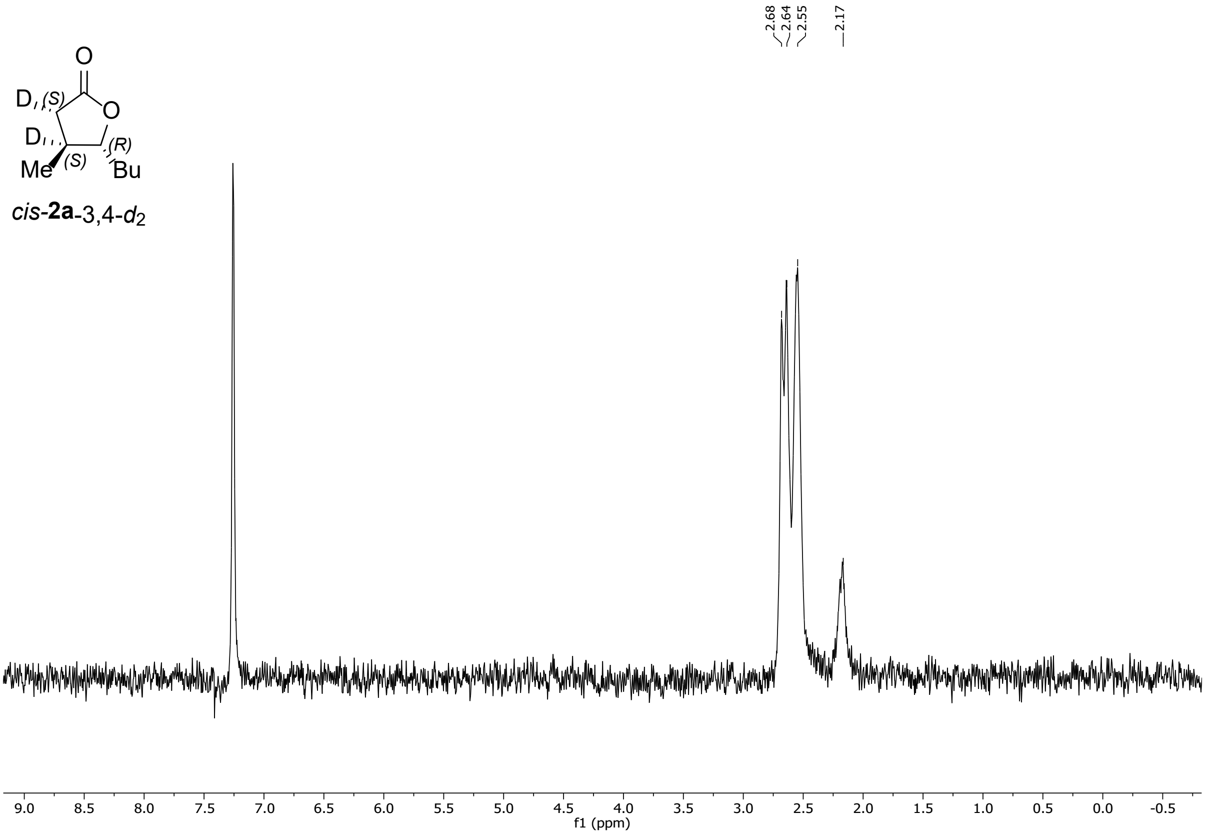

^{1}H-NMR ($CDCl_3$, 400 MHz)

Exp. A)

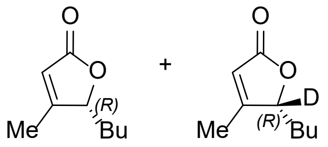

3a **3a**-5-*d*

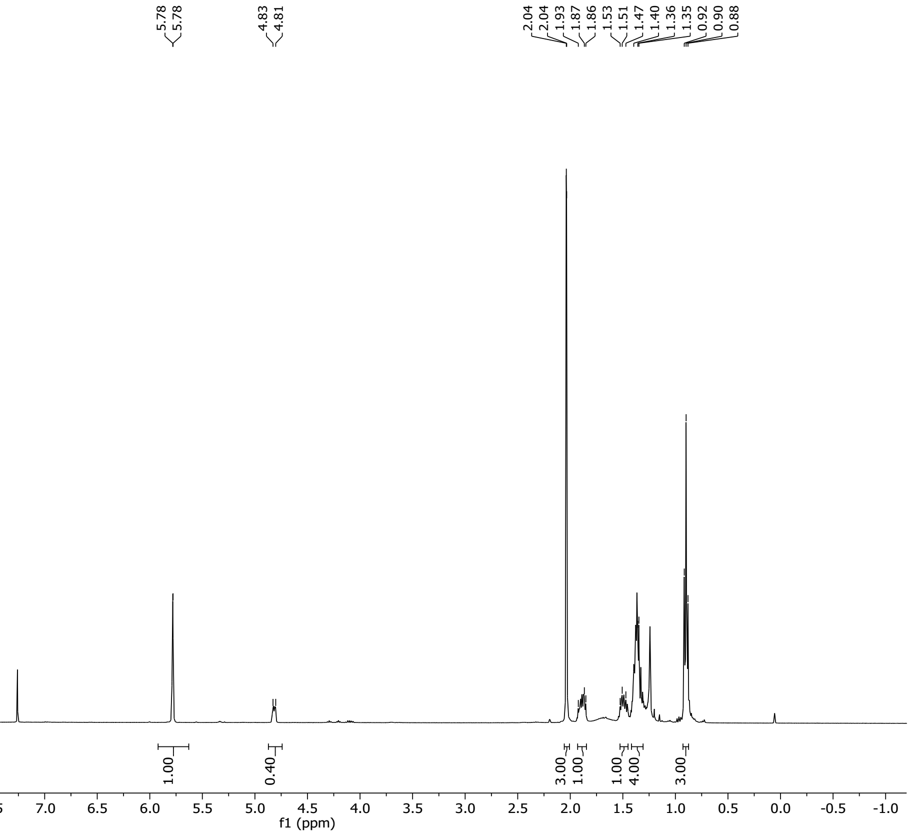

^{13}C{^{1}H}-NMR ($CDCl_3$, 101 MHz)

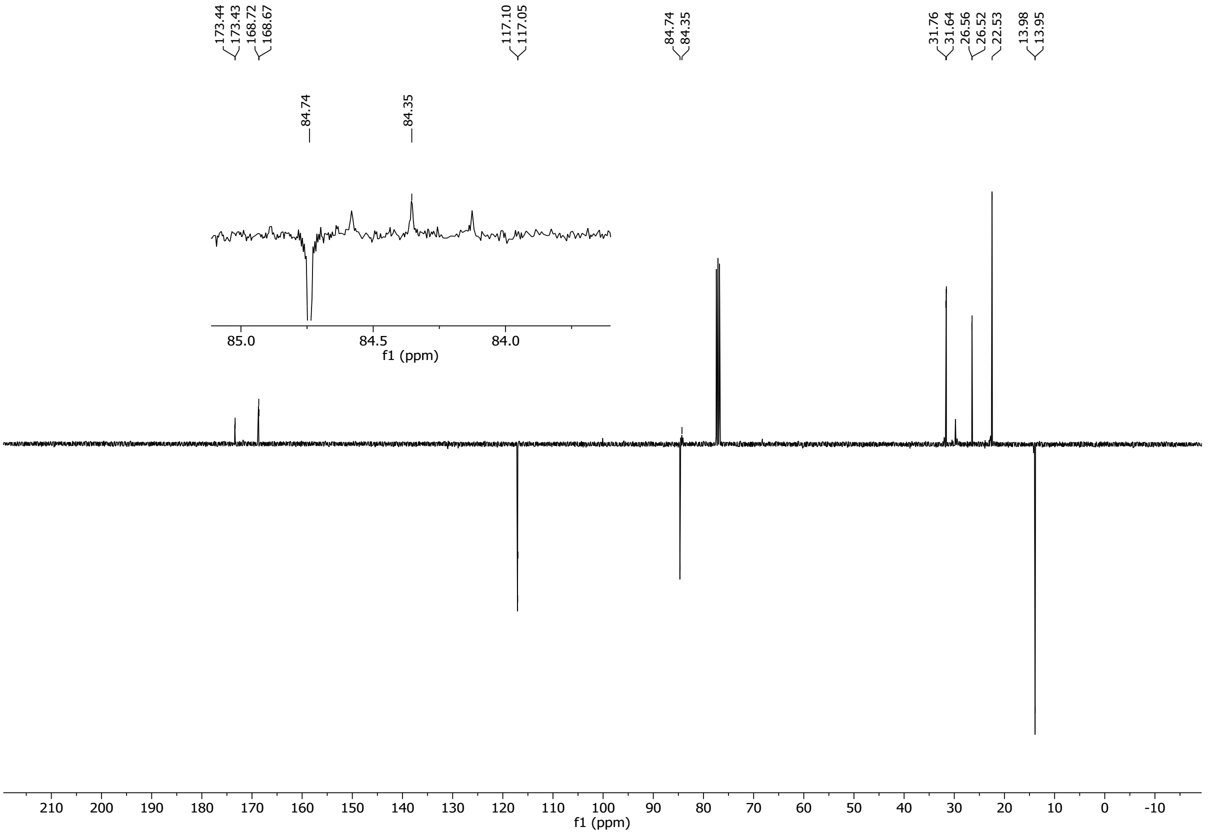

^{2}H-NMR ($CHCl_3$, 61.4 MHz)

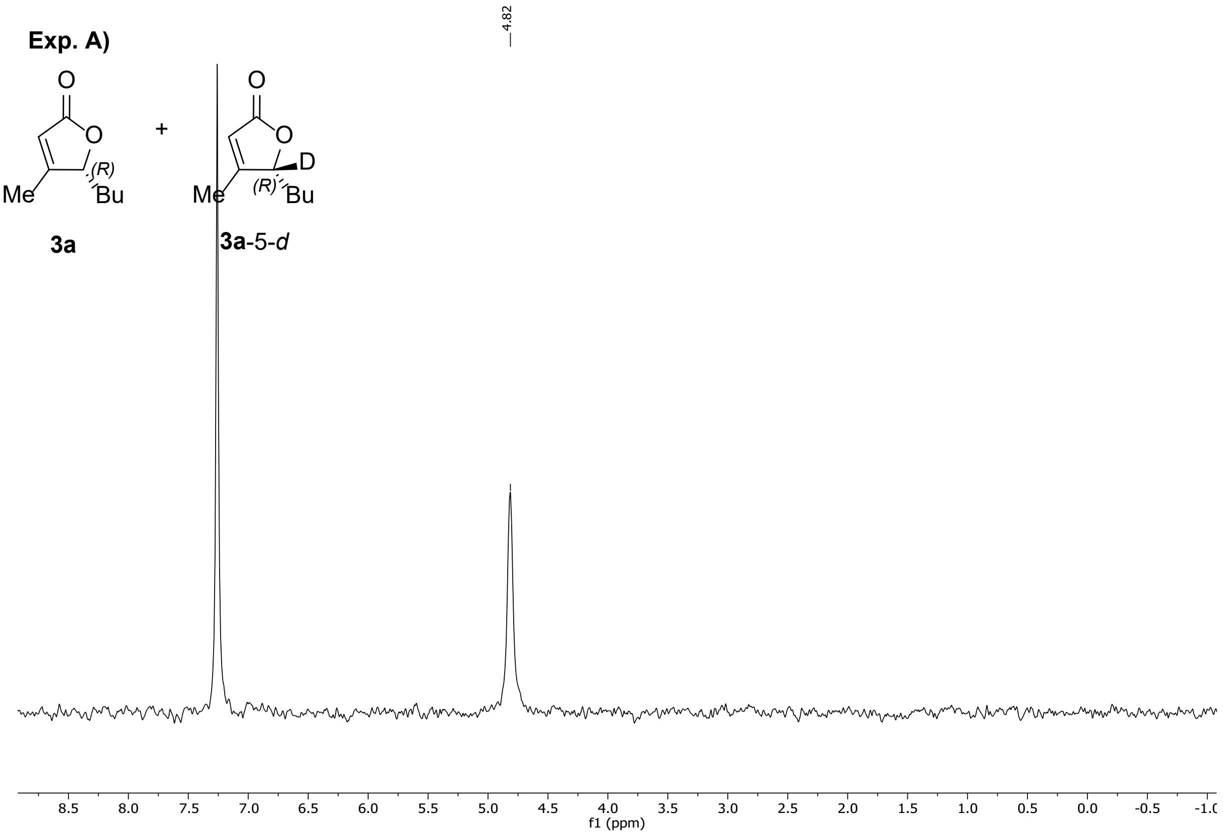

^{1}H-NMR ($CDCl_3$, 400 MHz)

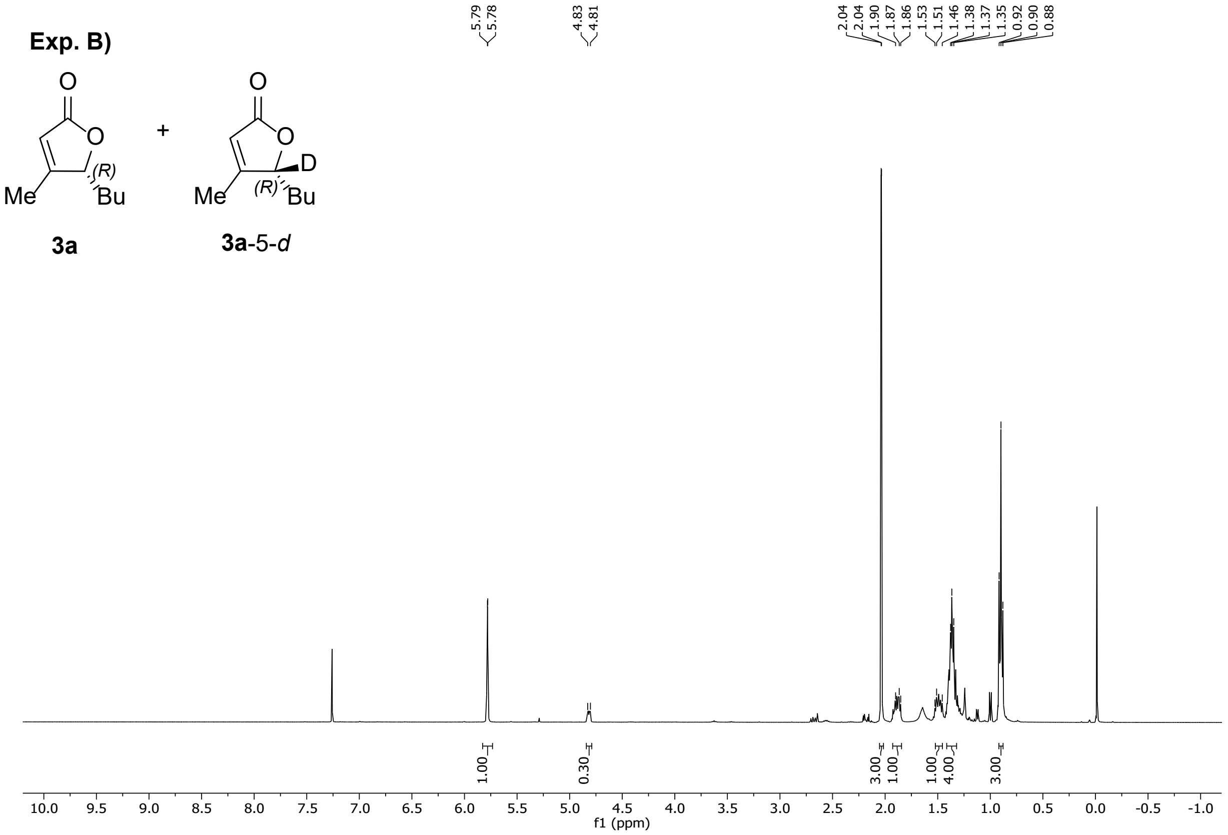

$^{13}C\{^{1}H\}$-NMR ($CDCl_3$, 101 MHz)

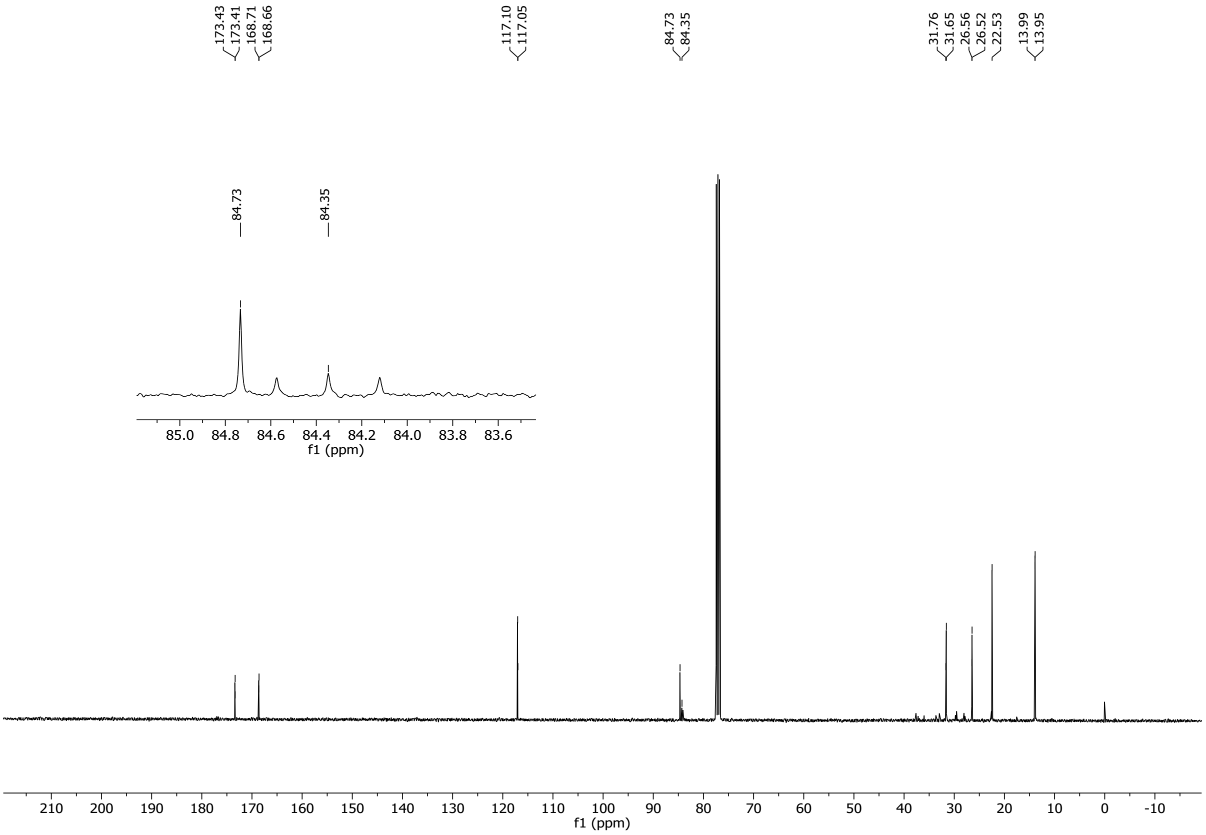

^{2}H-NMR ($CHCl_3$, 61.4 MHz)

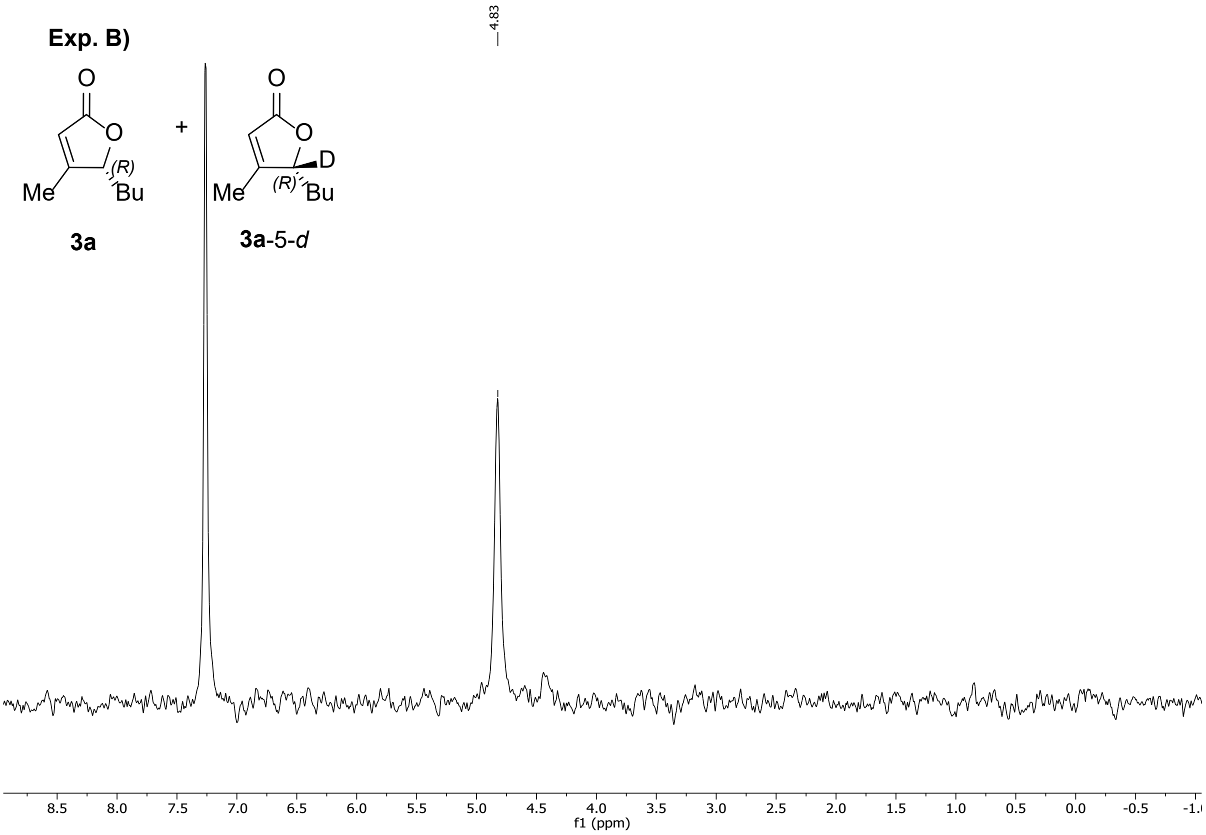

^{1}H-NMR ($CDCl_3$, 400 MHz)

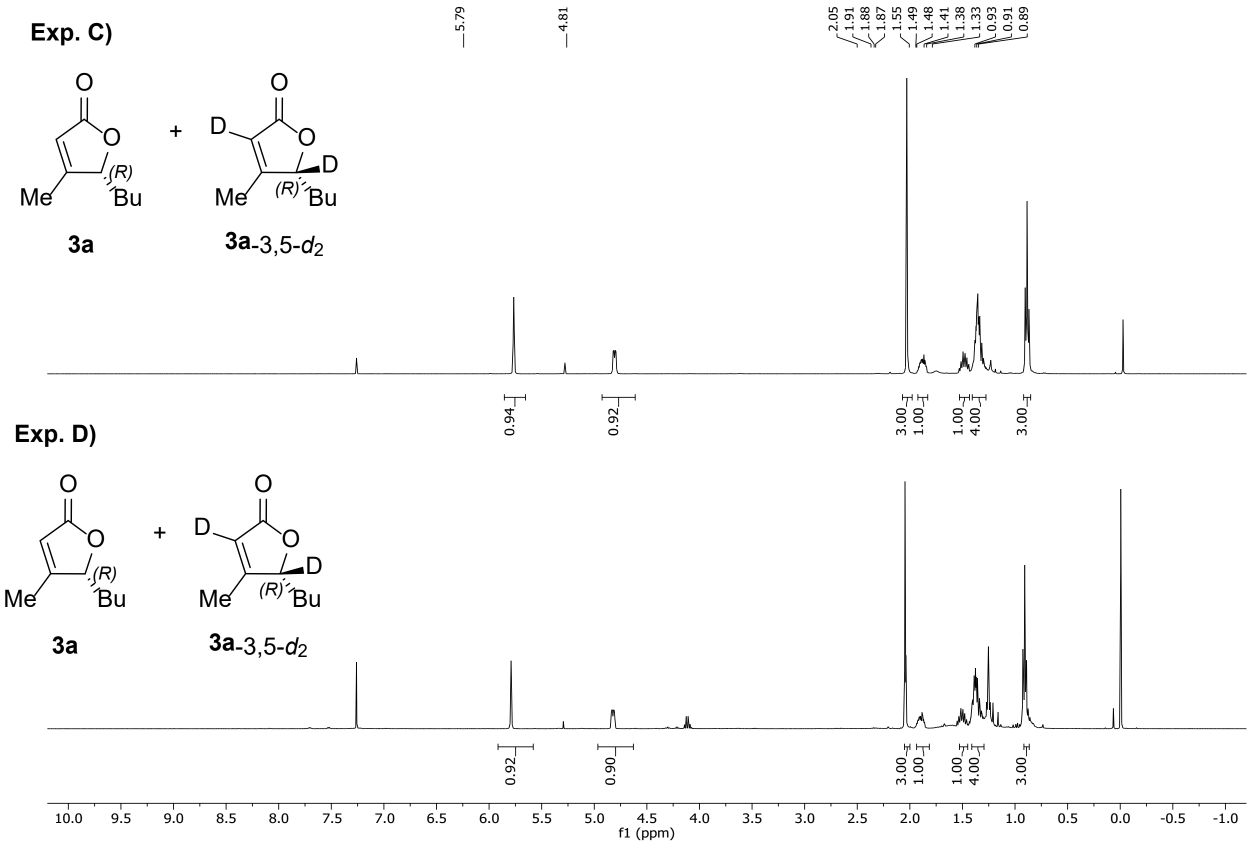

$^{13}C\{^{1}H\}$-NMR ($CDCl_3$, 101 MHz)

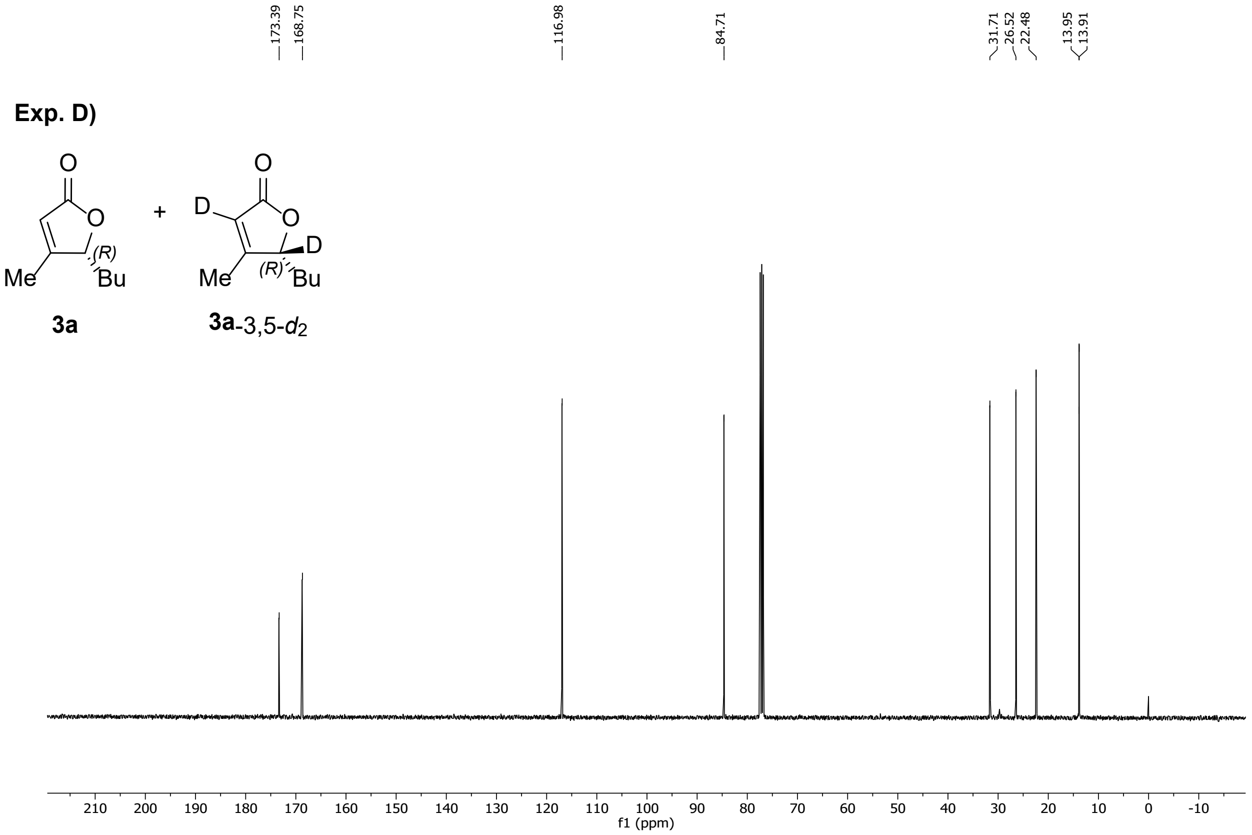

^{2}H-NMR ($CHCl_3$, 61.4 MHz)

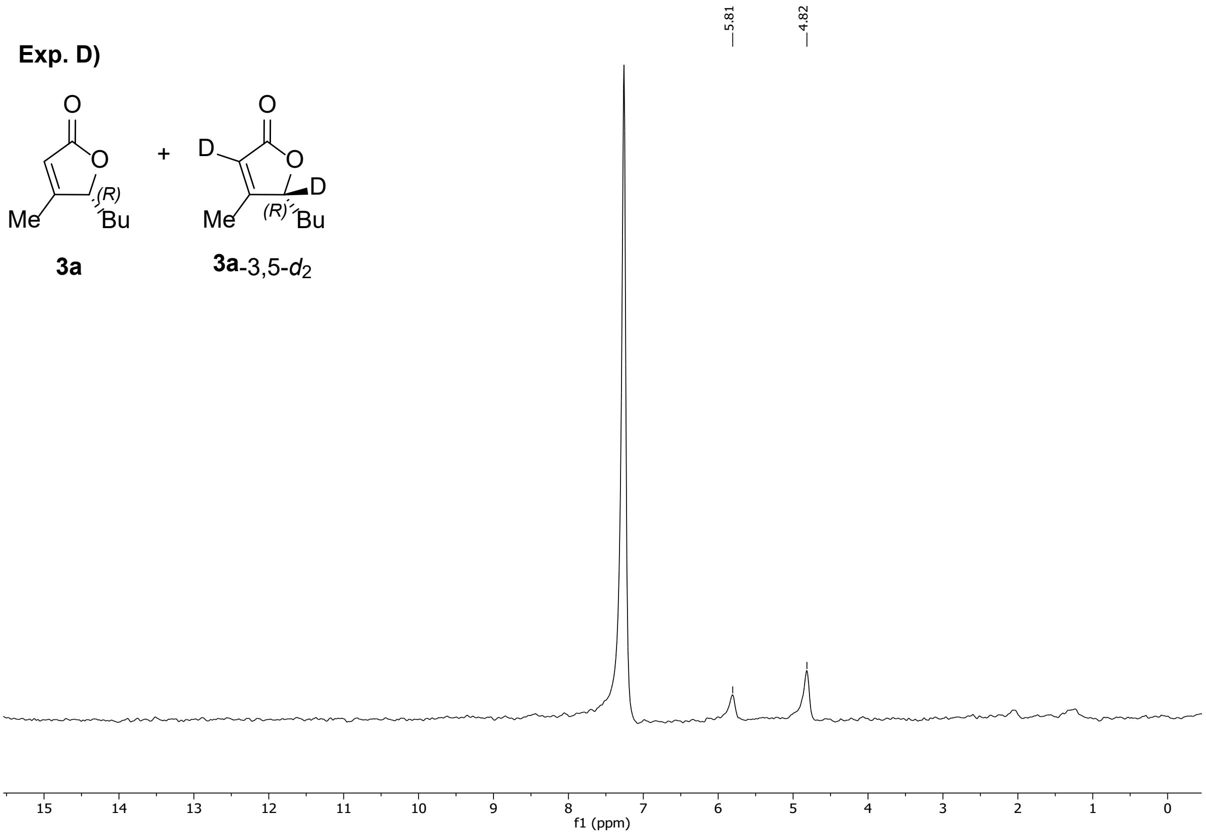

^{1}H-NMR ($CDCl_3$, 400 MHz)

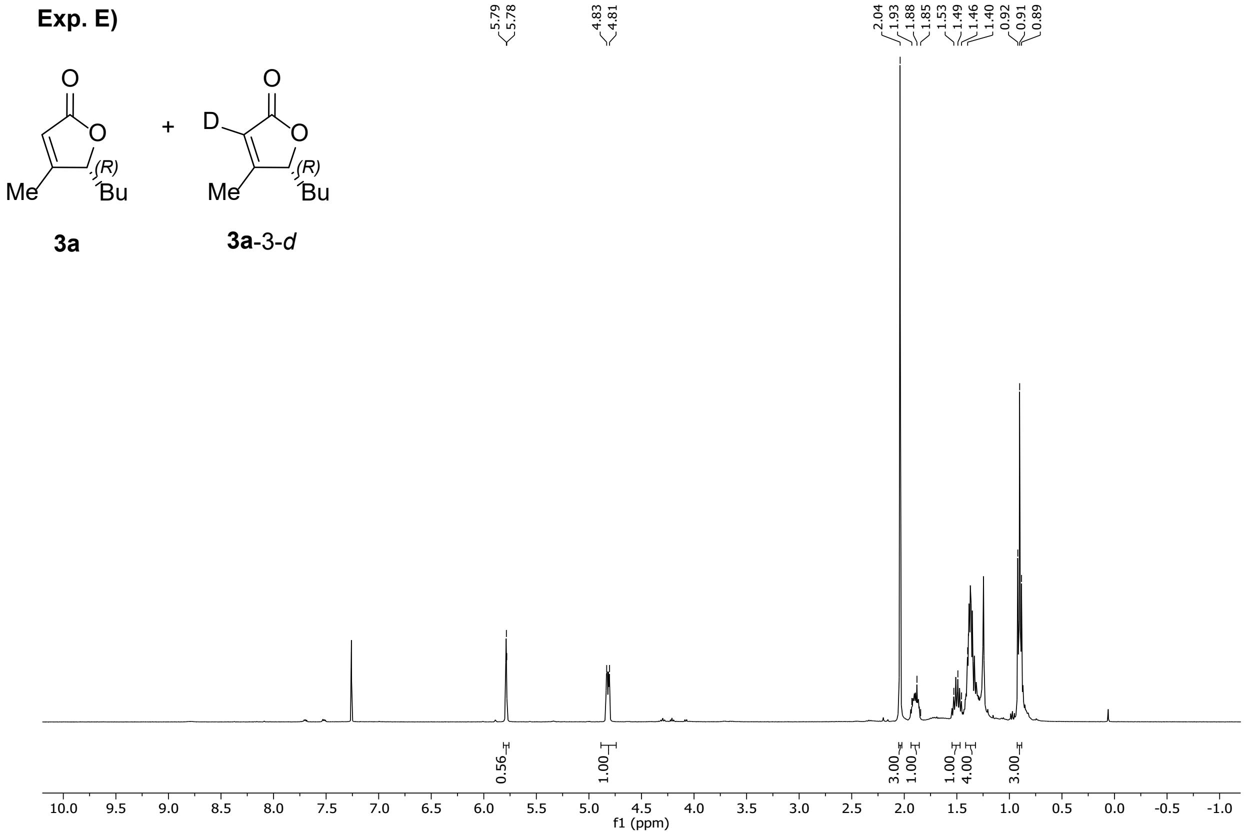

$^{13}C\{^{1}H\}$-NMR ($CDCl_3$, 101 MHz)

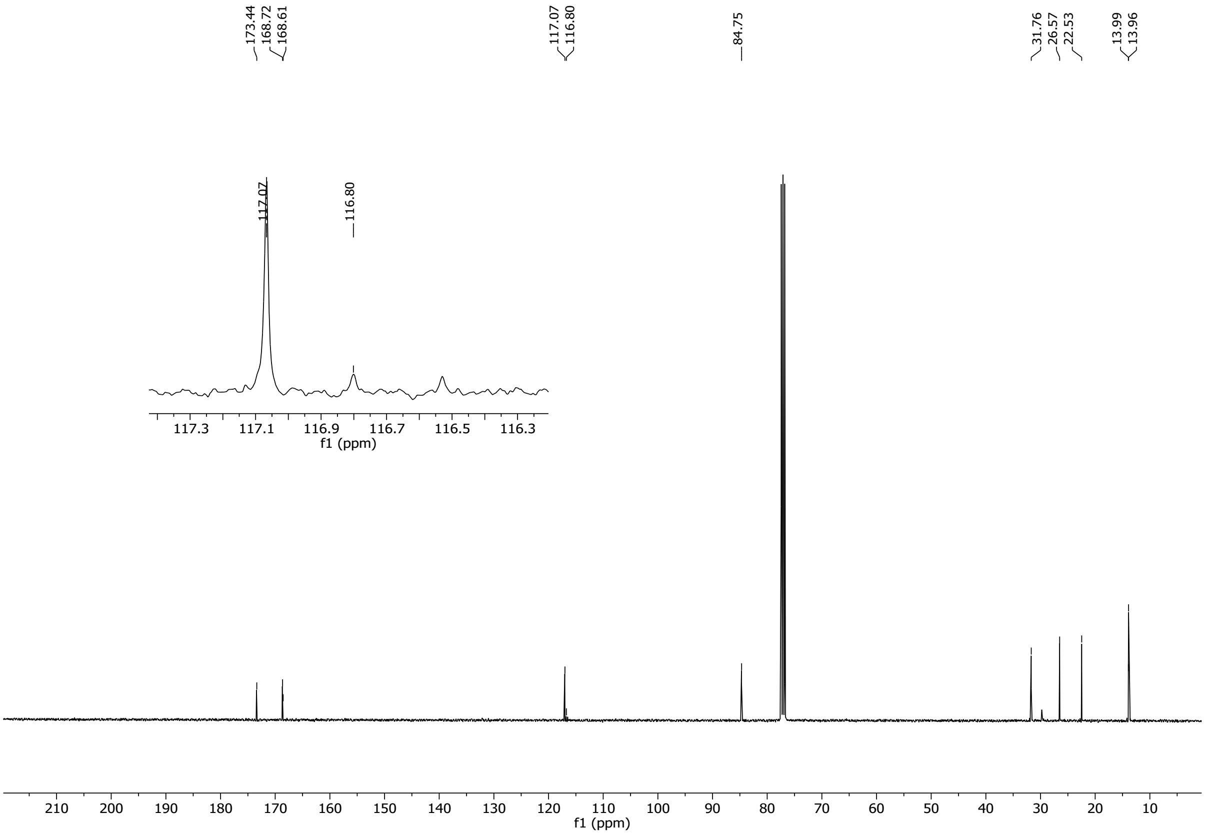

^{2}H-NMR ($CHCl_3$, 61.4 MHz)

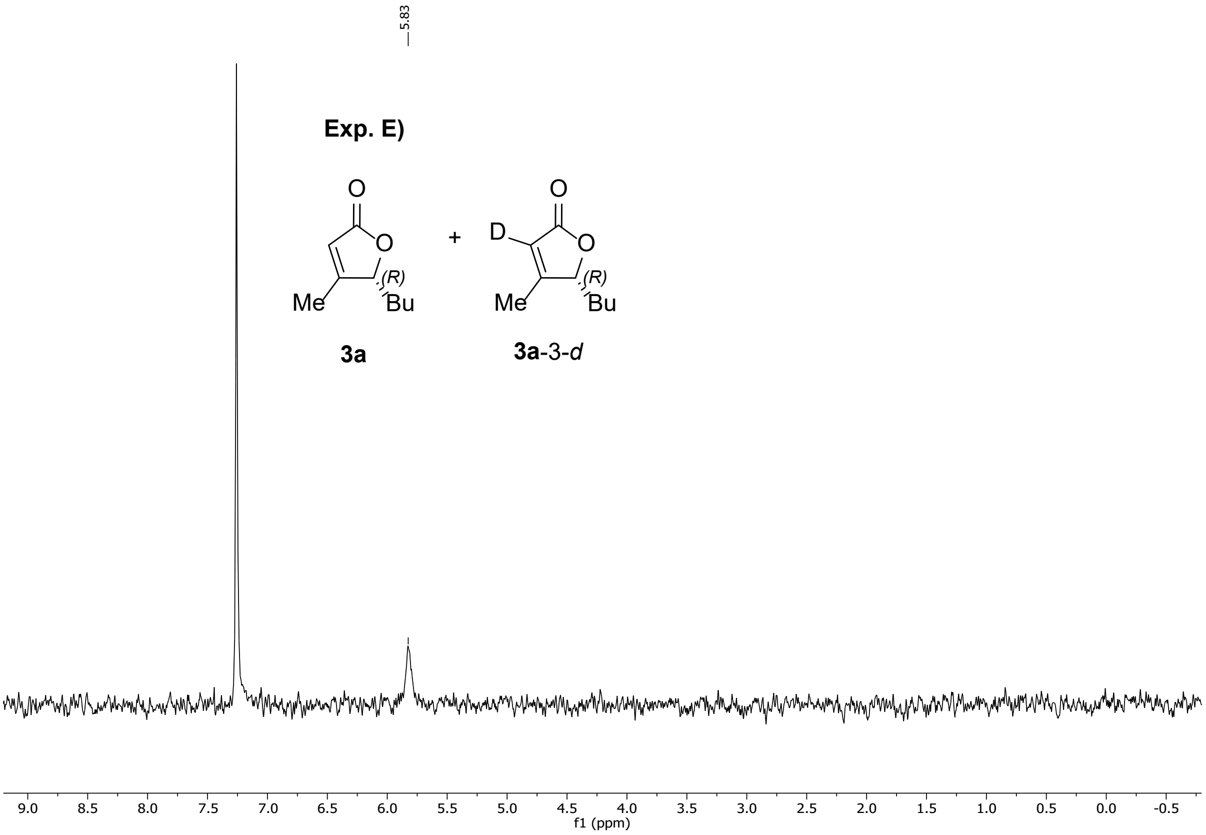

^{1}H-NMR ($CDCl_3$, 400 MHz)

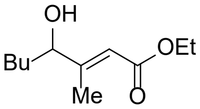

5a

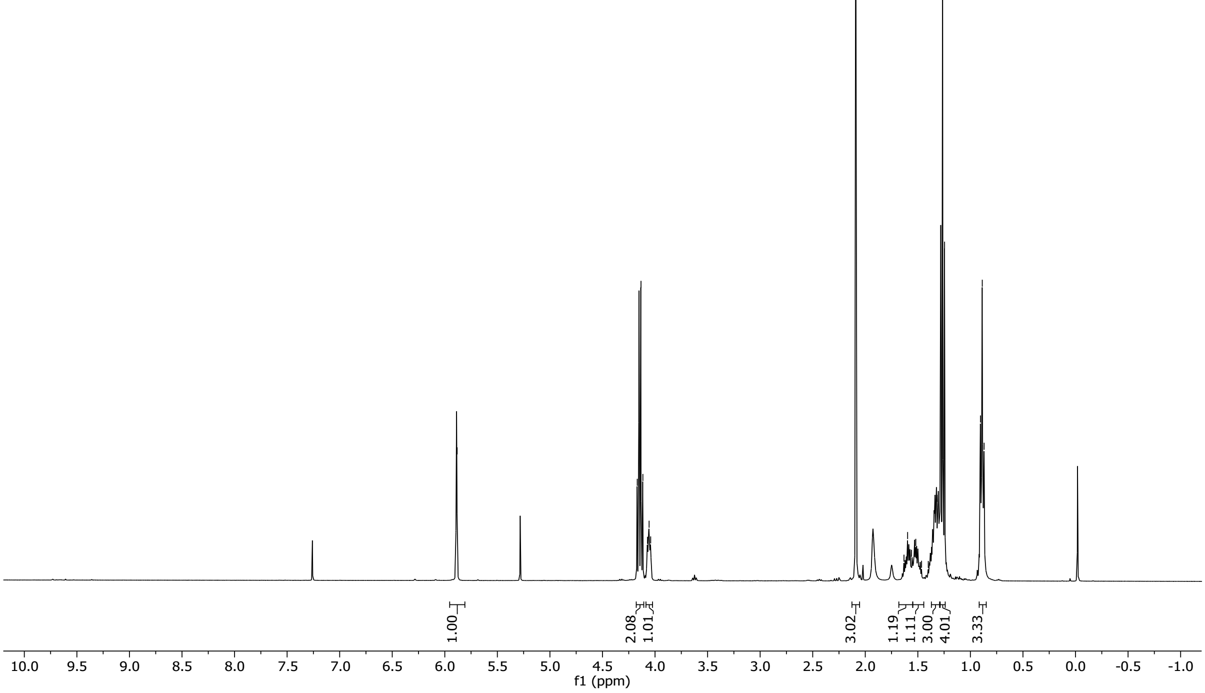

^{13}C{^{1}H}-NMR ($CDCl_3$, 101 MHz)

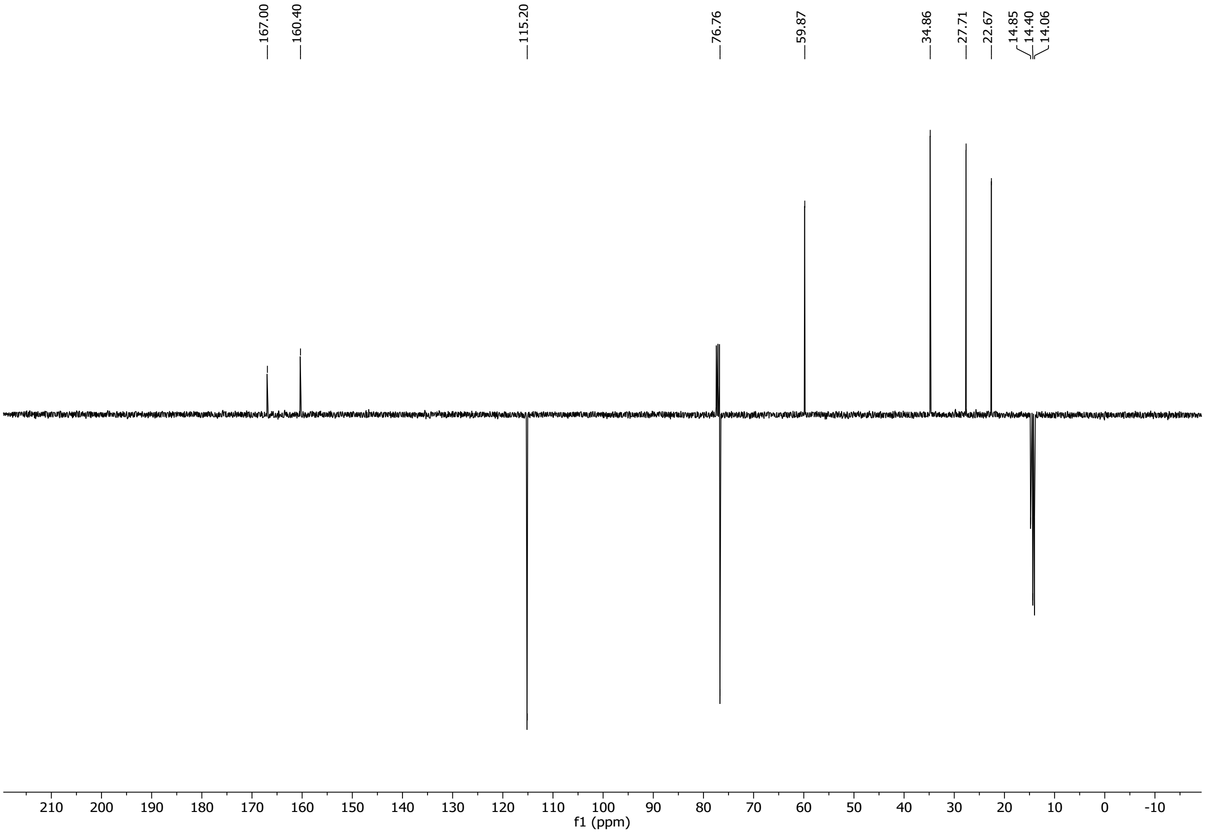

^{1}H-NMR ($CDCl_3$, 400 MHz)

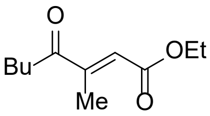

6a

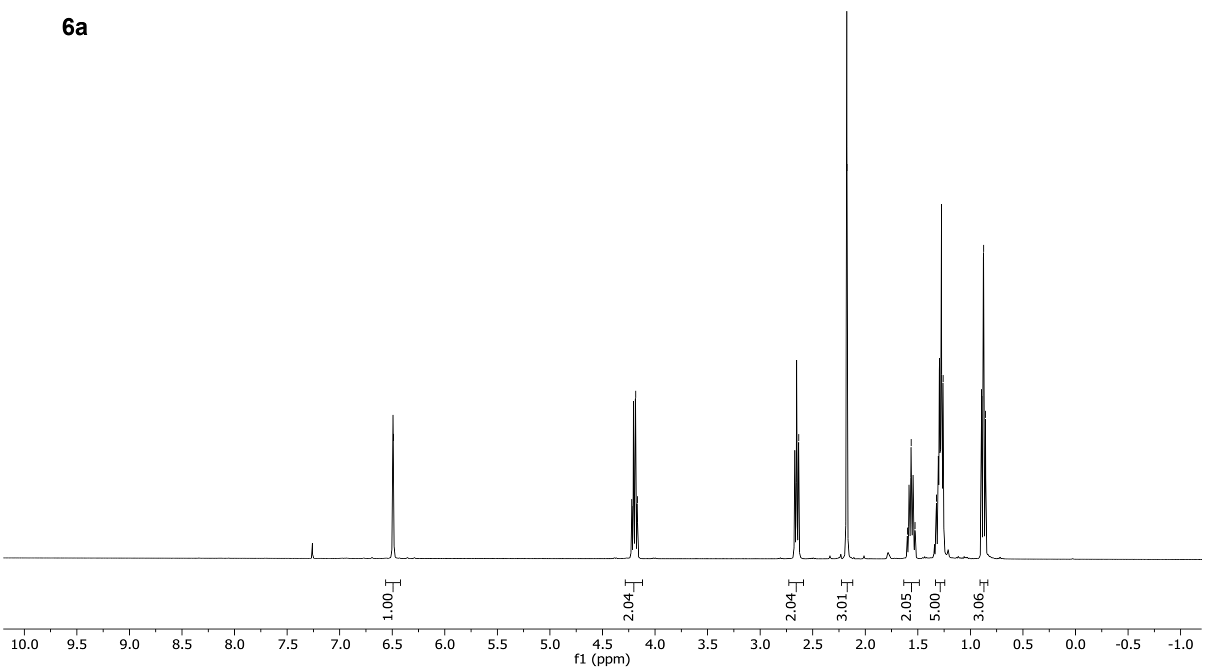

^{13}C{^{1}H}-NMR ($CDCl_3$, 101 MHz)

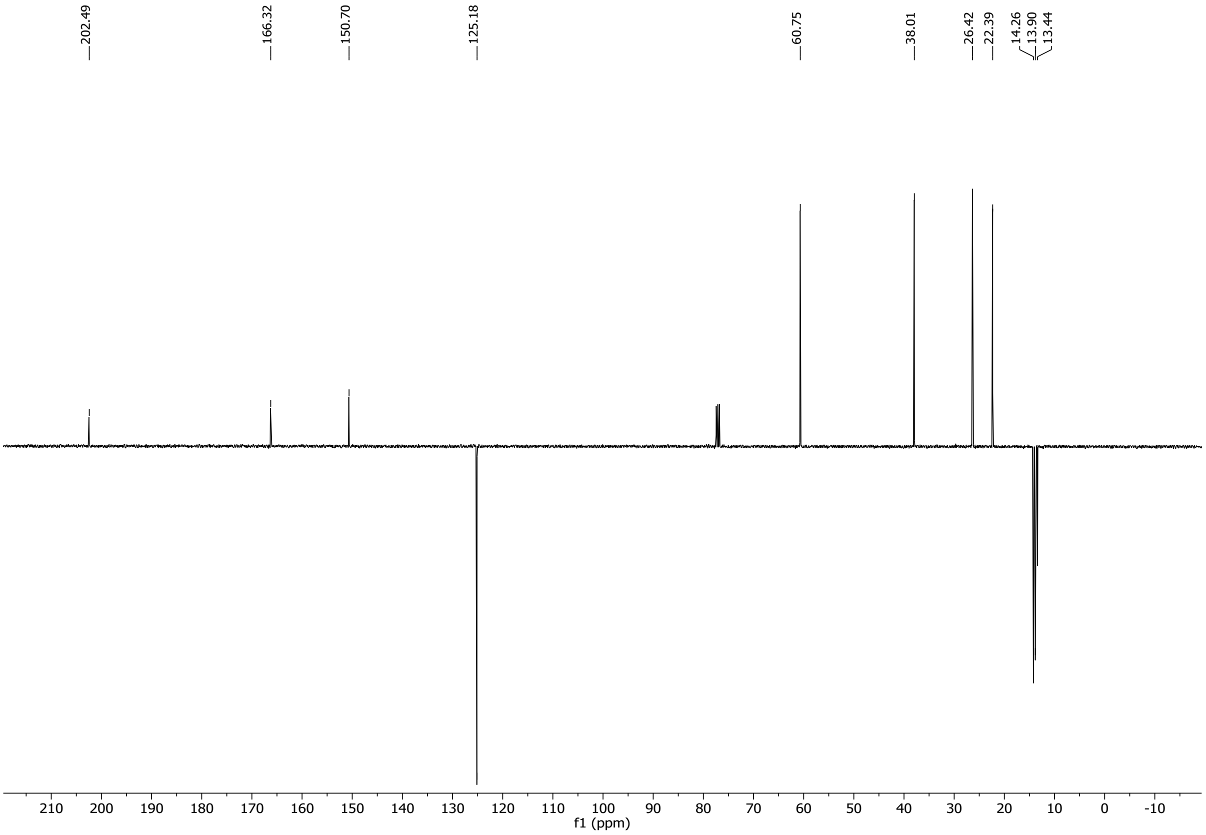

^{1}H-NMR ($CDCl_3$, 400 MHz)

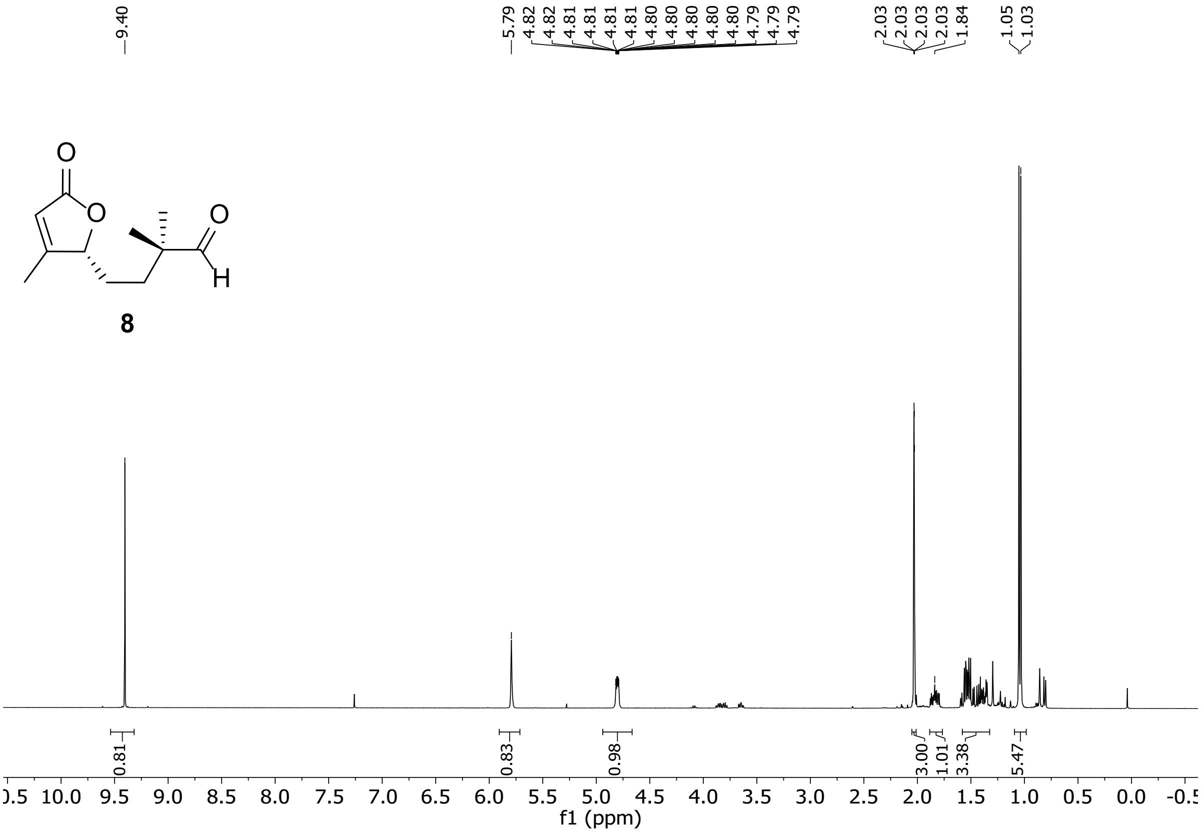

^{13}C{^{1}H}-NMR ($CDCl_3$, 101 MHz)

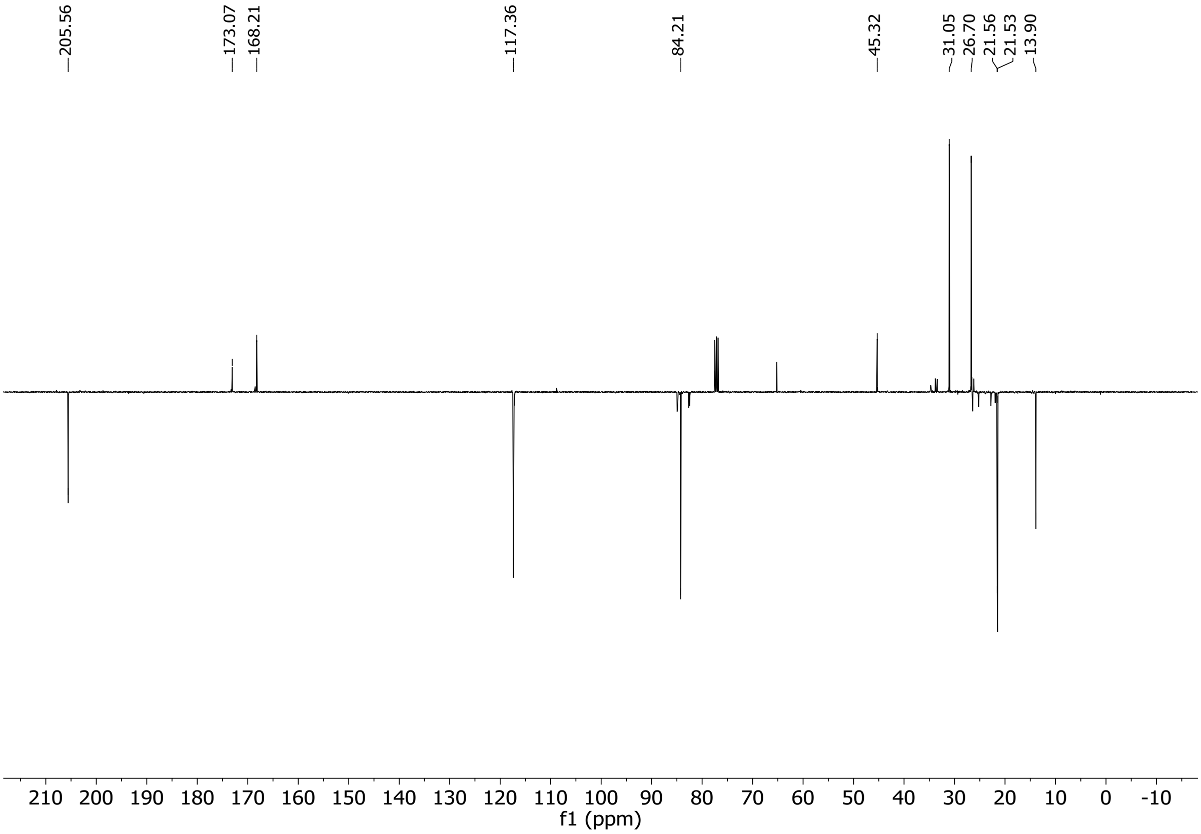

References

[1] Carman, L.; Kwart, L. D.; Hudlicky, T. Claisen/Ozonolysis Alternative to Alkylation of Enolate Anions. Synthesis of 1,4-Dicarbonyl Compounds. *Synt. Commun.* **1986**, *16*, 169-182.
[2] Comito, R. J.; Finelli, F. G.; MacMillan, D. W. C. Enantioselective Intramolecular Aldehyde α-Alkylation with Simple Olefins: Direct Access to Homo-Ene Products. *J. Am. Chem. Soc.* **2013**, *135*, 9358-9361.
[3] Mori, K.; Otsuka, T; Oda, M. Synthesis of all the four possible stereoisomers of pestalotin, a gibberellin synergist isolated from *Pestalotia cryptomeriaecola*. *Tetrahedron* **1984**, *40*, 2929-2934.
[4] Brenna, E.; Dalla Santa, F.; Gatti, F. G.; Gatti, G.; Tessaro, D. Exploiting the vicinal disubstituent effect on the diastereoselective synthesis of γ and δ lactones. *Org. Biomol. Chem.* **2019**, *17*, 813-821.
[5] Fang, J. M.; Liao, L. F.; Hong, B. C. Regio- and diastereoselective reactions of dithiol-substituted crotyllithium and aldehydes. *J. Org. Chem.* **1986**, *51*, 2625-2858.
[6] Vogel, C. J.; Butler, R.; Procter, D. J. An asymmetric, SmI_2-mediated approach to γ-butyrolactones using a new, fluorous-tagged auxiliary. *Tetrahedron* **2008**, *64*, 11876-11883.
[7] Leśniak, A.; Smuga, M.; Białońska, A.; Kula, J.; Wawrzeńczyk, C. Lactones 44. Microbial lactonization of γ-ketoacids. *J. Mol. Catal. B: Enzym.* **2014**, *106*, 32-39.
[8] Zhang, X.; Fu, C.; Yu, Y.; Ma, S. Stereoselective Iodolactonization of 4-Allenoic Acids with Efficient Chirality Transfer: Development of a New Electrophilic Iodination Reagent. *Chem. -Eur. J.* **2012**, *18*, 13229-13540.
[9] Tang, J.; Shinokubo, H.; Oshima, K. A New Strategy for the Preparation of an Active Mn(0) and its Use for Radical Cyclization Reactions. *Tetrahedron* **1999**, *55*, 1893-1904.
[10] Xie, X.; Stahl. Efficient and Selective Cu/Nitroxyl-Catalyzed Methods for Aerobic Oxidative Lactonization of Diols. *J. Am. Chem. Soc.* **2015**, *137*, 3767-3770.
[11] Kunesch, G.; Zagatti, P.; Lallemand, J. Y.; Debal, A.; Vigneron, J. P. Structure and synthesis of the wing gland pheromone of the male African sugar-cane borer. *Tetrahedron Lett.* **1981**, *22*, 5271-5271.
[12] Umemura, T; Mori, K. Stereocontrolled Synthesis of (2*R*,3*R*,5*R*,13*S*,14*R*)-(+)-Aplisiasphingosine, a Marine Terpenoid. *Agric. Biol. Chem*. **1987**, *51*, 1973-1982.
[13] Egami, H.; Tamaoki, S.; Abe, M.; Ohneda, N.; Yoshimura, T.; Okamoto, T.; Odajima, H.; Mase, N.; Takeda, K.; Hamashima, Y. Scalable Microwave-Assisted Johnson-Claisen Rearrangement with a Continuous Flow Microwave System. *Org. Process. Res. Dev.* **2018**, *22*, 1029-1033.
[14] Devalankar, D. A.; Karabal, P. U.; Sudalai, A. Optically pure γ-butyrolactones and epoxy esters via two stereocentered HKR of 3-substituted epoxy esters: a formal synthesis of (−)-paroxetine, Ro 67-8867 and (+)-eldanolide. *Org. Biomol. Chem*. **2013**, *11*, 1280-1285.
[15] Reissig, H.-U.; Angert, H. Ester Groups as Effective Ligands in Chelate-Controlled Additions of Cuprates and Grignard Reagents to Chiral β-Formyl Esters. *J. Org. Chem.* **1993**, *58*, 6280-6285.
[16] Cuvigny, T.; Julia, M.; Rolando, C. Stereoselective γ-*cis*-Vinylic Metalation of Tertiary Allylic Alcohols. *J. Chem. Soc., Chem. Commun.* **1984**, 8.
[17] Leisering, S.; Mavroskoufis, A.; Voßnacker, P.; Zimmer, R.; Christmann, M. Synthesis of Plakortolides E and I Enabled by Base Metal Catalysis. *Org. Lett.* **2021**, *23*, 4731-4735.
[18] Russel, C. A.; Sutherland, M. D. The Absolute Configuration of (-)-Ngaione, (-)-Epingaione and Related Substances. *Aust. J. Chem.* **1982**,*35,* 1881-94.
[19] Wang, D.; Jana, K.; Studer, A. Intramolecular Hydrogen Atom Transfer Induced 1,2-Migration of Boronate Complexes. *Org. Lett.* **2021**, *23*, 5876-5879.
[20] Meier, R.; Trauner, D. A Synthesis of (±)-Aplydactone. *Angew. Chem. Int. Ed.* **2016**, *55*, 11251-11255.
[21] Coulomb, J. $AlCl_3$-Promoted Conia-Ene-Related Cyclization of α,ω-Diethylenic Ketones and 1,2- or 1,3-Hydroalkenylation of Unactivated Cyclopropanes. *Adv. Synth. Catal.* **2023**, *365*, 2865-2870.
[22] Kang, Y.; Oh, C.H. Various synthetic approaches for [6,7,m]-tricyclic compounds containing an oxygen-bridged skeleton. *Bull. Korean Chem. Soc.* **2023**, *44*, 163-167.
[23] Reich, H. J.; Wollowitz, S. Preparation of *α,β*-Unsaturated Carbonyl Compounds and Nitriles by Selenoxide Elimination. *Org. React.* **1993**, *44*, 1-296.
[24] Sutar, R. L.; Sen, S.; Eivgi, O.; Segalovich, G.; Schapiro, I.; Reany, O.; Lemcoff, N. G. Guiding a divergent reaction by photochemical control: bichromatic selective access to levulinates and butenolides. *Chem. Sci*. **2018**, *9*, 1368-1374.
[25] Jefford, C. W.; Sledeski, A. W.; Boukouvalas, J. Synthesis of *cis* and *trans* Whisky and Cognac Lactones by the Regiocontrolled Alkylation of 2-(Trimethylsi1oxy)furan. *Helv. Chim. Acta.* **1989**, *72*, 1362-1370.
[26] Morikawa, T.; Nishiwaki, T.; Iitaka, Y.; Kobayashi, Y. Radical cyclization to fluorinated double bonds: 5-exo ring closure of bromoacetals derived from fluoroallyl alcohols. *Tetrahedron Lett*. **1987**, *28*, 671-674.
[27] Sharma, V.; Kelly, g. T.; Watanabe, C. M. H. Exploration of the Molecular Origin of the Azinomycin Epoxide: Timing of the Biosynthesis Revealed. *Org. Lett.* **2008**, *10*, 4815-4818.
[28] Pisani, L; Superchi, S.; D'Elia, A.; Scafato, P.; Rosini, C. Synthetic approach toward *cis*-disubstituted γ- and δ-lactones through enantioselective dialkylzinc addition to aldehydes: application to the synthesis of optically active flavors and fragrances. *Tetrahedron* **2012**, *68*, 5779-5784.

[29] Bonete, P.: Náiera Lithium 3-Lithio-3-tosylalkanoates: β-Acylvinyl Anion Equivalents of β-Lithiated α,β-unsaturated Carboxylic Acids. *J. Org. Chem.* **1994**, *59*, 3202-3209.
[30] Manna, A.; Chakraborty, I; Chatterjeem, S.; Bhaumik, T. A general and concise stereodivergent chiral pool approach toward *trans*-(4*S*,5*R*)- and *cis*-(4*R*,5*R*)-5-alkyl-4-methyl-γ-butyrolactones: Syntheses of (+)-*trans*- and (+)-*cis*-whisky and cognac lactones from D-(+)-mannitol. *Carbohydr. Res*. **2021**, *510*, 108452.
[31] Mándity, I. M.; Martinek, T. A.; Darvas, F.; Fülöp, F. A simple, efficient, and selective deuteration via a flow chemistry approach. *Tetrahedron Lett*. **2009**, *50*, 4372-4374. b) Irfan, M.; Petricci, E.; Glasnov, T.; Taddei, M.; Kappe, O. Continuous Flow Hydrogenation of Functionalized Pyridines. *Eur J. Org. Chem.* **2009**, *9*, 1327-1334.
[32] Chu, L.; Zhang, X.; Li, J.; Deng, X.; Wu, M.; Cheng, Y.; Zhu, W.; Qian, X.; Bai, Y. Continuous-flow synthesis of polysubstituted γ-butyrolactones *via* enzymatic cascade catalysis. *Chin. Chem. Lett*. **2024**, *35*, 108896.
[33] Hernik, D.; Gatti, F.; Brenna, E.; Szczepańska, E.; Olejniczak, T.; Boratyński, F. Stereoselective synthesis of whisky lactone isomers catalyzed by bacteria in the genus *Rhodococcus*. *Front. Microbiol.* **2023**, 14:1117835.
[34] Kumru, C.; Classen, T.; Pietruszka, J. T. Enantioselective, Catalytic One-Pot Synthesis of γ-Butyrolactone-Based Fragrances *ChemCatChem* **2018**, *10*, 4917-4926.
[35] Haschimoto, S.-I.; Sakata, S.; Sonegawa, M.; Ikegami, S. A Total Synthesisof (±)-Forskolin *J. Am. Chem. Soc.* **1988**, *110*, 3670-3672.
[36] Gaussian 16, Revision C.01, M. J. Frisch, G. W. Trucks, H. B. Schlegel, G. E. Scuseria, M. A. Robb, J. R. Cheeseman, G. Scalmani, V. Barone, G. A. Petersson, H. Nakatsuji, X. Li, M. Caricato, A. V. Marenich, J. Bloino, B. G. Janesko, R. Gomperts, B. Mennucci, H. P. Hratchian, J. V. Ortiz, A. F. Izmaylov, J. L. Sonnenberg, D. Williams-Young, F. Ding, F. Lipparini, F. Egidi, J. Goings, B. Peng, A. Petrone, T. Henderson, D. Ranasinghe, V. G. Zakrzewski, J. Gao, N. Rega, G. Zheng, W. Liang, M. Hada, M. Ehara, K. Toyota, R. Fukuda, J. Hasegawa, M. Ishida, T. Nakajima, Y. Honda, O. Kitao, H. Nakai, T. Vreven, K. Throssell, J. A., Jr. Montgomery, J. E. Peralta, F. Ogliaro, M. J. Bearpark, J. J. Heyd, E. N. Brothers, K. N. Kudin, V. N. Staroverov, T. A. Keith, R. Kobayashi, J. Normand, K. Raghavachari, A. P. Rendell, J. C. Burant, S. S. Iyengar, J. Tomasi, M. Cossi, J. M. Millam, M. Klene, C. Adamo, R. Cammi, J. W. Ochterski, R. L. Martin, K. Morokuma, O. Farkas, J. B. Foresman and D. J. Fox, Gaussian, Inc., Wallingford CT, 2016.
[37] Head-Gordon, M.; People, J. A.; Frisch, M. J. *Chem. Phys. Lett.* **1988**, *153*, 503-506.
[38] Marenich, A. V.; Cramer, C. J.; Truhlar, D. G. Universal solvation model based on solute electron density and a continuum model of the solvent defined by the bulk dielectric constant and atomic surface tensions. *J. Phys. Chem. B* **2009**, *113*, 6378-6396.
[39] Head-Gordon, M.; Head-Gordon, T. Analytic MP2 Frequencies Without Fifth Order Storage: Theory and Application to Bifurcated Hydrogen Bonds in the Water Hexamer. *Chem. Phys. Lett.* **1994**, *220*, 122-28.
[40] CYLview, 1.0b; Legault, C. Y., Université de Sherbrooke, **2009** (http://www.cylview.org).
